# Gold-Catalyzed Carbonyl Release and its Adaptation for Prodrug Therapy Using Multivalent Lectin-Directed Artificial Metalloenzymes

**DOI:** 10.1021/jacsau.5c01331

**Published:** 2026-01-12

**Authors:** Jing Huang, Yiling Liu, Yufei Li, Jianghui Du, Xiao Han, Kenward Vong

**Affiliations:** Department of Chemistry, 58207 The Hong Kong University of Science and Technology, Clear Water Bay, Kowloon, Hong Kong, China 999077

**Keywords:** bioorthogonal chemistry, gold catalysis, prodrug therapy, artificial metalloenzyme, cancer targeting

## Abstract

In the framework of developing artificial metalloenzyme (ArM) prodrug therapies, two main factors need to be considered; the cancer targeting capabilities of the ArM biocatalyst and the bioorthogonal prodrug activation mechanism. In this study, both these aspects were investigated to develop an example of an anticancer ArM prodrug strategy. To address targeting, the concept of multivalent lectin-directed artificial metalloenzymes was established using a Halotag-PduU-ACG lectin fusion protein (HtPA) functionalized with a gold catalyst. Acting through multivalent binding of hexameric lectin complexes (caused by PduU oligomerization), selective binding to sialic acid-rich cancer cells was proven. To address prodrug activation, the propargylbenzoxime (PBO) group was developed to undergo gold-catalyzed hydroamination, followed by spontaneous N–O bond cleavage to release carbonyl functional groups under mild and physiological conditions. Further adaptation of the PBO group was also explored so that carbonyl release could elicit the synthesis of indole-containing molecules. HtPA-based artificial metalloenzymes were then subsequently applied in cell assays for the activation of a PBO-based prodrug to highlight this alternative approach of an ArM prodrug therapy.

## Graphical abstract

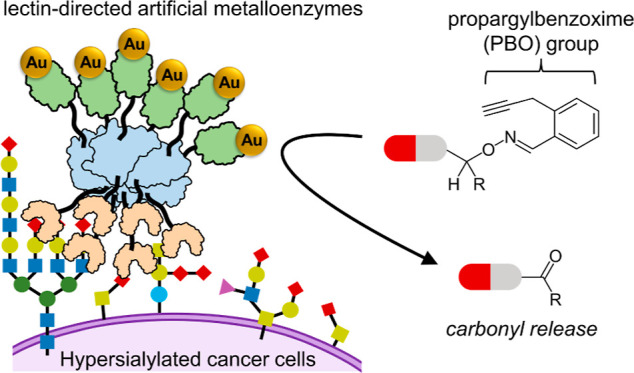

## Introduction

The proof that abiotic transition metal catalysts (i.e., Pd, Au) could be used within biological systems (i.e., cultured cells,^1^ zebrafish,^2^ mice^3^) galvanized an entirely new field dedicated to adopting in vivo transition metal catalysis for biotechnological or therapeutic applications. One popular approach to do this has been with artificial metalloenzymes (ArMs), which embeds relevant metal catalysts within a protein scaffold to influence factors related to stereoselectivity or biocompatibility.^4^ Recent examples of protein scaffolds used for ArM development include streptavidin,^5–11^ myoglobin,^12–17^ carbonic anhydrase,^18–21^ LmrR,^22–28^ nitrobindin,^29–34^ HaloTag protein,^35,36^ as well as from de novo proteins.^37^

One underexplored application of ArMs has been their modification with targeting capabilities to create ArM prodrug therapies for cell-specific drug release. Albumin-based ArMs have by far been the most studied protein scaffold for this approach, with cancer targeting being achieved through functionalization with complex *N*-glycans,^38–40^ and RGD-based peptides.^41,42^ And although currently limited, researchers have also explored developing ArMs from monoclonal antibodies like Herceptin.^43,44^

Another important consideration for any ArM prodrug therapy relates to the activation mechanism. From current metal-catalyzed bioorthogonal reactions,^4,45–48^ dissociative reactions have proven to be the most versatile for transforming prodrugs into their bioactive drug counterparts. In the literature, a majority of these dissociative reactions have largely focused on the release of amine- or alcohol-containing molecules through depropargylation,^2,43,49–72^ deallylation,^1,71–84^ deallenylation,^85^ and various cyclizations that utilize 2-alkynylbenzamide,^86^ pentynoyl amide,^87^ and pentenoic amide substrates.^88^ Notable exceptions include the release of aromatic groups via ring-closing metathesis,^89^ and a recent breakthrough with β-lapachone release proceeding through C–C bond cleavage.^90^ Thus, far, there are no known abiotic metal-catalyzed reactions that can release aldehyde- or ketone-containing molecules in a bioorthogonal manner.

In this study, both a cancer-targeting ArM and a gold-catalyzed carbonyl-releasing reaction were developed concurrently to create a working example of an anticancer ArM prodrug therapy (Figure 1). In the design of the ArM, three key components contribute to its functionality. First, lectins are used to elicit cancer targeting through binding to the hypersialylated environment of cancer cell surfaces. This is based on the well-known observation that aberrant glycosylation during carcinogenesis often stimulates an increase of sialic acids by 40–60% on cell surfaces,^91^ thereby promoting metastasis through enhanced immune evasion and migration,^91,92^ given the naturally weak monovalent binding of lectin monomers (Figure 1A), it was reasoned that lectin multivalency (via oligomeric lectins) could be exploited for stronger binding to cancer cells.

**Figure 1:**
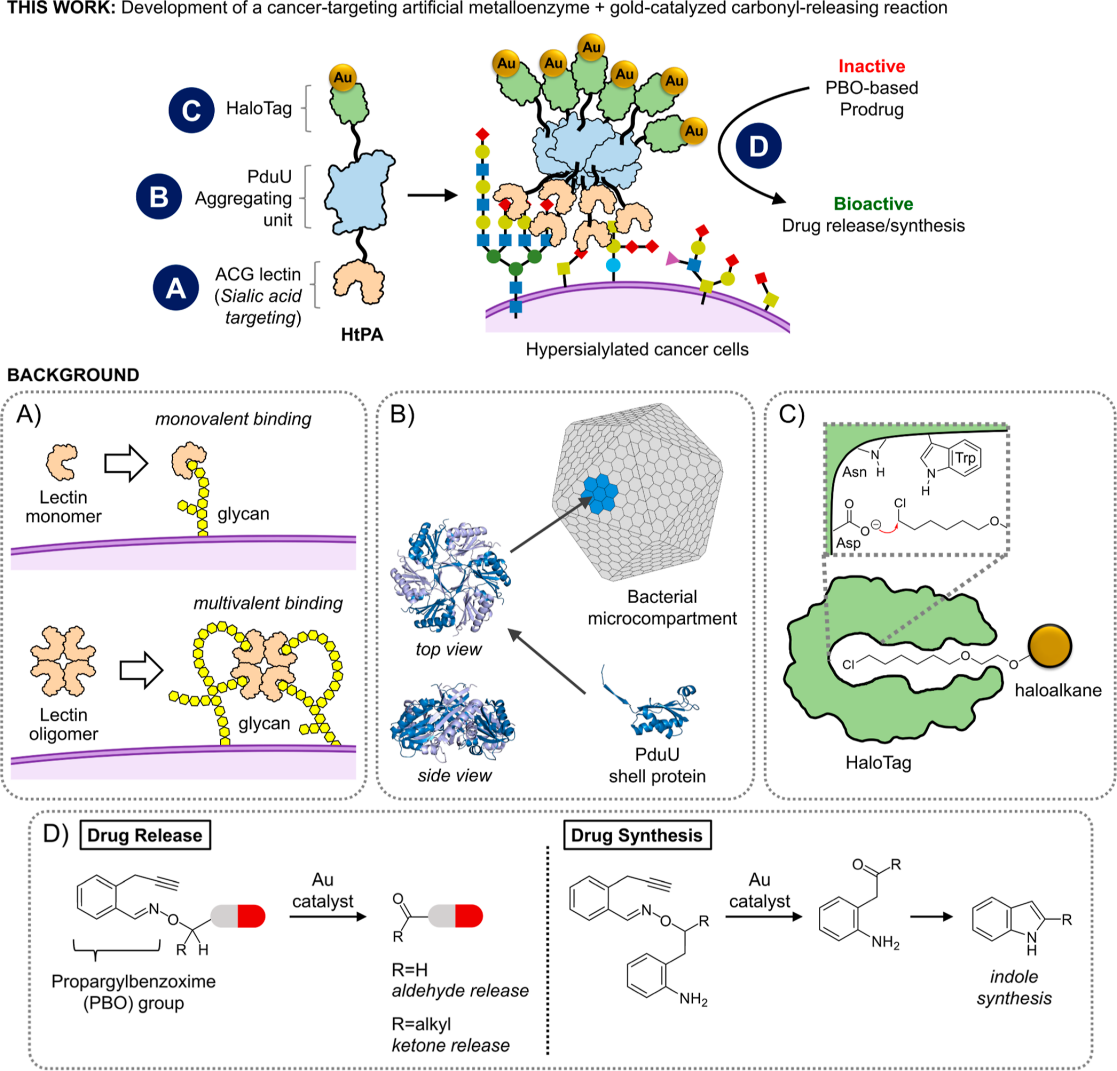
A cancer-targeting artificial metalloenzyme and a gold-catalyzed carbonyl-releasing reaction were developed concurrently to create a working example of an anticancer ArM prodrug therapy. To develop the artificial metalloenzyme, three key literature principles were considered. (A) Lectin monomers typically have weak monovalent binding to glycan ligands, but experience stronger binding through multivalent binding. (B) PduU is a monomeric shell protein from *S. enterica* found to naturally self-assemble into bacterial microcompartments. Thus, it is reasoned that fusion proteins containing the PduU monomer should also naturally self-assemble into hexameric complexes. (C) HaloTag is a self-labeling protein capable of covalently bonding with haloalkane substrates, thereby opening up the possibility to covalently attach gold-complexes linked to a haloalkane. (D) To develop the gold-catalyzed carbonyl releasing reaction, the dissociative mechanism of the propargylbenzoxime (PBO) group was designed and tested for the release of carbonyl functional groups following gold exposure under physiological conditions. Adaptation of the PBO group can be made to either release carbonyl-containing drugs, or to synthesize drugs through the construction of an indole core.

To create these oligomeric lectin complexes, the second key component of the ArM design looked to fuse monomeric lectins to a PduU shell protein, which should drive self-assembly into hexameric complexes. This strategy is based on the fact that PduU shell proteins are found in nature as the hexameric building blocks that form bacterial microcompartment organelles found in *Salmonella enterica* (Figure 1B).^93^

Finally, the third key component relates to the protein scaffold that will house the transition metal catalyst. To benefit from its capacity for covalent conjugation via a haloalkane linker (Figure 1C), the HaloTag protein was chosen as a suitable scaffold to anchor the metal catalyst used in this study.^94^

Taking these key concepts into consideration, a Halotag-PduU-ACG lectin fusion protein (**HtPA**) was created, followed by its functionalization with a gold catalyst (**HtPA-Au**). Justification in the design of this ArM is best observed with its predicted 3D structure (Figure S1), which forecasts that minimal interference will occur between the three core components: PduU-based oligomerization, glycan-binding ACG lectin, and the gold-embedded HaloTag protein.

In the next part of this study, efforts were made to create an alternative prodrug activation mechanism. To do this, the propargylbenzoxime (PBO) group was developed to release carbonyl functional groups (Figure 1D), which was confirmed to proceed under mild, physiological conditions. The basis behind this reaction is through the formation of a 6-*exo*-dig cyclization intermediate (promoted via hydroamination), followed by N–O bond cleavage to release a carbonyl moiety.

Overall, this study reports on the conceptualization of multivalent lectin-directed artificial metalloenzymes, as well as the development of a gold-catalyzed carbonyl-releasing reaction that is compatible with mild, physiological conditions. Once both the viability of PBO release chemistry and **HtPA** ArMs were proven, the focus of this study then shifted toward creating a working example of an anticancer prodrug therapy. To do this, a PBO prodrug was designed so that phenstatin (a ketone-containing antimitotic agent) could be released on the surfaces of hypersialylated cancer cell surfaces, thereby exerting its anticancer effects.

## Results and Discussion

### Reaction Development and Catalyst Screening

To begin this study, the hypothesis that PBO groups can undergo hydroamination followed by N–O bond cleavage needed to be investigated and confirmed. To facilitate this, model substrates **1a**–**b** were first synthesized according to Scheme S1.

As a preliminary test, the aldehyde releasing capabilities of substrate **1a** was tested through screening a variety of transition metals (i.e., Au, Pt, Pd, Ag, Ru, and Cu) under aqueous conditions (Figure 2A, Table S2). From these experiments, the release of its corresponding aldehyde product, benzaldehyde **3a** was indeed observed, which was best induced via a gold­(III) catalyst. For example, a turnover (TON) of 13.9 was achieved using 5 mol % of **Au1** catalyst in THF/PBS buffer conditions for 1 h. The only other metal that showed the ability to induce PBO-based aldehyde release was palladium, albeit at a lower reactivity (TON of 8.3) under similar conditions. Taking this data into account, this study proceeded forward with a focus on developing gold-catalyzed PBO chemistry.

**Figure 2:**
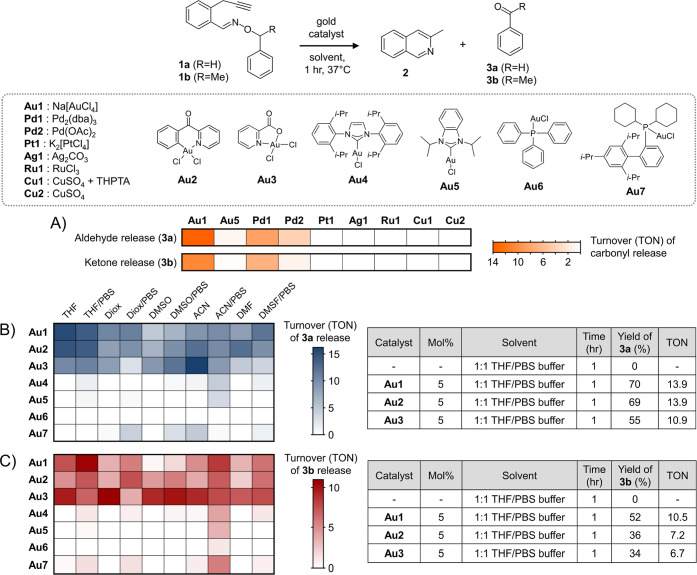
Reactivity studies to explore the carbonyl release capabilities of PBO precursors **1a**–**b** under different catalysts and conditions. (A) Testing for the release of carbonyl products **3a** and **3b** from PBO precursors **1a** and **1b** exposed to different transition metal catalysts in a 1:1 solution of THF/PBS buffer pH 7.4. Reactions were incubated at 37 °C for 1 h at 5 mol % of catalyst. Subsequently, a screen with gold catalysts **Au1–Au7** was conducted for the gold-catalyzed release of (B) the aldehyde product **3a** from precursor **1a**, and (C) the ketone product **3b** from precursor **1b**. In general, release yields were obtained and then converted to turnover (TON). Heat map summaries are shown to summarize the TON numbers obtained in different tested solvents. For solvent mixtures, 1:1 solutions of organic solvent with PBS buffer pH 7.4 were used. In the embedded tables, selected reaction examples and their obtained yields are shown.

To standardize conditions, varying the percentage of **Au1** (1–20%) was shown to correlate well with reactivity (entries 20–24, Table S3). Since a near quantitative yield of **3a** release was realized by only 10 mol % of gold catalyst, further experiments were thus conducted at 5 mol % of catalyst using a 1 h reaction time frame.

In the next step, substrates **1a**–**b** were screened against a variety of gold­(I) and gold­(III) catalysts to observe for the release of their aldehyde/ketone counterparts under aqueous conditions. For aldehyde release, a concurrent screen of compatible solvent systems and gold catalysts (**Au1**–**Au7**) was carried out using substrate **1a**, as summarized by the heat map in Figure 2B (details in Table S3). TON values ranged from 2.7 to 16.2 when using gold­(III) catalysts **Au1**–**Au3** under aqueous conditions. On the other hand, reactivity with gold­(I) catalysts **Au4**–**Au7** were found to be much poorer, with a majority showing TON values < 2.

Next, ketone release was confirmed using substrate **1b**. As expected, data indicated successful release of the corresponding ketone product, acetophenone **3b**, under similar conditions as before. Operating at 5 mol % of catalyst with a 1 h reaction time frame, another reactivity screen was performed under varying solvents and gold catalysts (**Au1**–**Au7**), as summarized by the heat map in Figure 2C (details in Table S4). With gold­(III) catalysts **Au1**–**Au3**, TON values under aqueous conditions ranged from 1.7 to 10.9, while gold­(I) catalysts **Au3**–**Au7** were again found to be poor (TON values <2).

From the screening data for aldehyde/ketone release via PBO removal, there appeared to be no significant trends with regards to the ideal solvent system. Likely, this is due to the differential solubilities of the tested gold catalysts. However, it can be clearly seen that the inclusion of water does not substantially hamper gold catalysis (compared to corresponding conditions with only organic solvent). It should be further noted that the high usage of organic solvent for these preliminary reactivity tests was largely dictated by the insoluble nature of the ligated metal complexes. Thus, considering the data, it can be concluded that gold-catalyzed PBO removal for the release of aldehydes/ketones is largely compatible under aqueous conditions.

One other observation that can be noted is that TON values for ketone release are generally lower than their aldehyde equivalent. One likely explanation stems from the fact that *O*-alkyl oxidation requires abstraction of the α-hydrogen. It is likely that access to this hydrogen is more sterically encumbered for substrates that release ketones compared to aldehydes.

### Adoption of Carbonyl Release toward Indole Synthesis

Moving on, the next focus of this study shifted to indoles, which is a common scaffold widely present in a number of FDA-approved drugs.^95,96^ With this consideration, adaptations were made for gold-triggered PBO removal to release carbonyl groups capable of eliciting indole formation. The key to this concept is the release of a phenylacetaldehyde with an ortho-positioned amine. Then through imine formation and bond rearrangement, an indole structure should be favorably produced.

To investigate this, substrates **4b**–**g** were synthesized according to Schemes S2 and S3 and then tested for the gold-triggered formation of their corresponding indoles under aqueous conditions (Figure 3, Tables S5–S10). As a control, substrate **4a** was also tested to ensure that phenylacetaldehyde could be properly released, which occurred with a TON of 2.9. It should be noted that the turnover of **4a** is considerably lower compared to **1a** (TON of 9.9 under similar conditions). A likely explanation for this observation is that the α-hydrogen of **1a** is located at the benzylic position, thereby making it more acidic and easier to facilitate oxidation.

**Figure 3:**
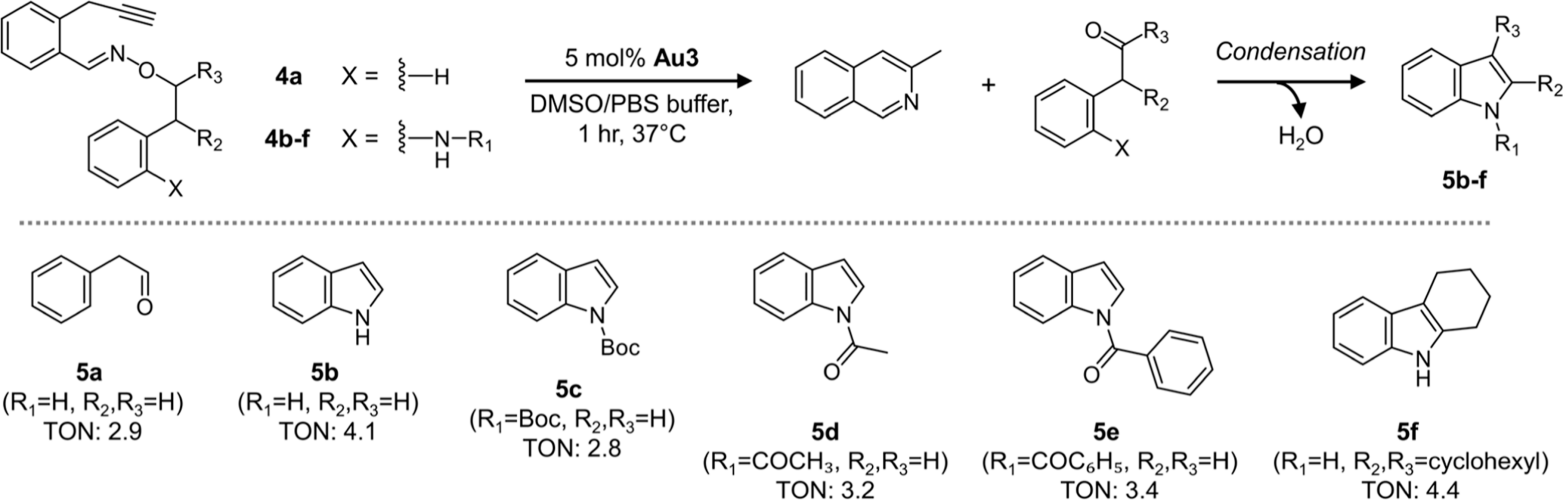
Reactivity and substrate scope studies were conducted for the conversion of PBO precursors **4b**–**f** to their respective indoles **5b–f** under gold-catalyzed, aqueous conditions. TON values were calculated for the formation of indoles from substrate exposure to 5 mol % of **Au3** in DMSO/PBS buffer conditions for 1 h. As a control, the production of phenylacetaldehyde **5a** from substrate **4a** was done to confirm aldehyde release from a relevant structural scaffold.

To begin, substrate **4b** was reacted with **Au3** under aqueous conditions, successfully producing a simple indole **5b** with a TON of 4.1. Given these promising results, a number of adjustments were next made to probe the substrate scope of PBO-based indole formation that could be applicable to various drug scaffolds.

One such adjustment was to probe the formation of indoles from ortho-positioned carbamates and amides. Surprisingly, reactivity remained strong as Boc-indole **5c**, acetyl-indole **5d**, and benzoyl-indole **5e** could all be produced with TON values in the range of 2.8–3.4. These scaffolds could potentially be of interest since acylated indoles are known to be present in several FDA-approved drugs (i.e., indomethacin, acemetacin). In another investigation, experiments looked at whether the synthesis of a 2,3,4,9-tetrahydro-1*H*-carbazole core could be possible, which is relevant to drugs like Selisistat, GSK983, and Frovatriptan. Starting from **4f**, indole **5f** was successfully formed with a TON of about 4.4.

Overall, the substrate scope studies showed that several indole types could be feasibly generated via PBO-based chemistry. Additionally, most of the indole structures generated in Figure 3 have relevance in several bioactive molecules. Thus, PBO-based prodrugs could theoretically be applied to numerous drugs with clinical relevance.

### Mechanistic Analysis

Inspired by a previous report where isoquinoline-*N*-oxides were found to undergo spontaneous N–O bond cleavage,^97^ the PBO group was originally designed so that gold-activation of the propargyl group could hypothetically promote 6-*exo*-dig cyclization through hydroamination of the oxime nitrogen atom (Figure 4A). The resultant intermediate will then likely tautomerize to favor aromatization. The formed isoquinoline *N*-oxide is then expected to undergo N–O bond cleavage because of *O*-alkyl oxidation following abstraction of its α-hydrogen. This sequence of steps should thus lead to the release of a carbonyl moiety.

**Figure 4:**
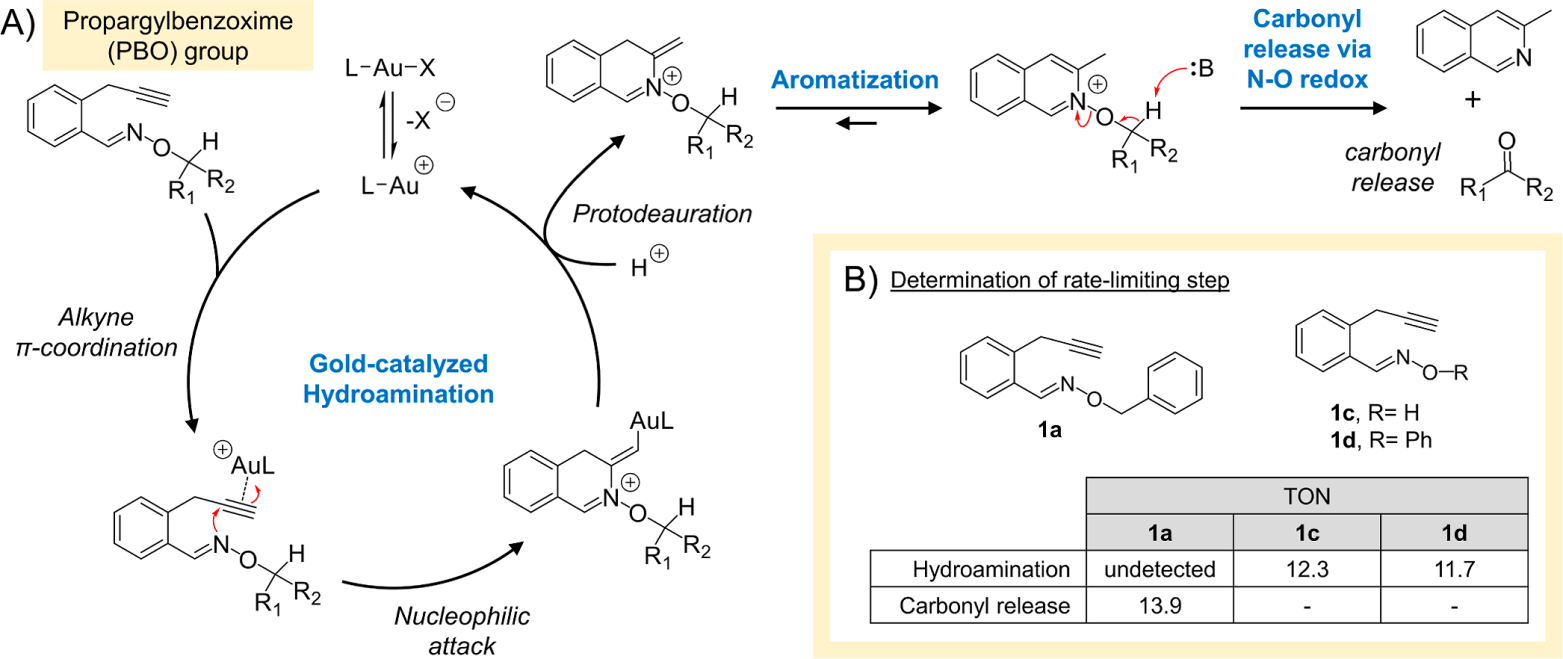
A) Proposed mechanism for the gold-catalyzed release of carbonyls from the PBO group. Following gold coordination to the alkyne, 6-*exo*-dig cyclization is expected to proceed through hydroamination of the oxime nitrogen atom. Following aromatization, the isoquinoline *N*-oxide intermediate is then expected to undergo N–O bond cleavage, leading to carbonyl release. (B) With analogues **1c** and **1d**, which cannot undergo carbonyl release, their TONs (for hydroamination) were revealed to be about 84 to 88% of the TON (for carbonyl release) of **1a**. This suggests that hydroamination is likely the rate-limiting step for PBO chemistry.

Given that there are two major steps in PBO chemistry (hydroamination and N–O bond scission leading to carbonyl release), the next set of experiments set out to determine which step was rate-limiting. To do this, analogues **1c** and **1d** were synthesized, which are substrates unable to undergo *O*-alkyl oxidation. When exposed to the **Au1** catalyst, TON values recorded for simple hydroamination was 12.3 for **1c** and 11.7 for **1d** (Figure 4B). These hydroamination TONs are roughly 84 to 88% of the TON acquired for **1a**, which instead pushes completely toward carbonyl release without any detectable traces of its hydroamination intermediate. Thus, it can indirectly suggested that hydroamination is likely the rate-limiting step for PBO chemistry.

### Preparation of Artificial Metalloenzymes

Moving onto the next portion of this study, the construction and activity of HaloTag-based ArMs was investigated. While considering the feasibility of embedding ligated gold complexes into a HaloTag protein, inspection of the crystal structure revealed a rather small opening (∼6 Å radius) leading into the binding cavity (Figure S13). Thus, it was reasoned that by opening up the cavity entrance, better positioning of the bulky gold catalysts could be possible. The Met175Ala and Phe144Ala mutations were planned during the construction of the HaloTag gene, which theoretically opens up the binding cavity entrance to a radius of 8–10 Å.

Genes for the HaloTag M175A/F144A mutant (**Ht**) and the corresponding HaloTag-ACG (**HtA**) and HaloTag-PduU-ACG (**HtPA**) proteins were synthesized and then subcloned into pET-19b vectors. Following standard expression and His-tag purification protocols, these recombinant proteins were then produced and purified. In addition, gold catalysts **Au8**–**Au11** were synthesized according to Scheme S5.

### PBO Reactivity Studies Using Gold-Based ArMs

To conduct reactivity studies, it was reasoned that **Ht-Au** ArMs would be representative of **HtPA-Au** ArM reactivity given the shared HaloTag protein component. To prepare the **Ht-Au** ArMs, treated **Ht** was incubated with **Au8**–**Au11** (catalysts with differing linker lengths) in PBS buffer for roughly 2 h to facilitate covalent attachment (Figure 5A, Scheme S7). Following removal of excess reagents, these protein complexes were then used immediately for testing.

**Figure 5:**
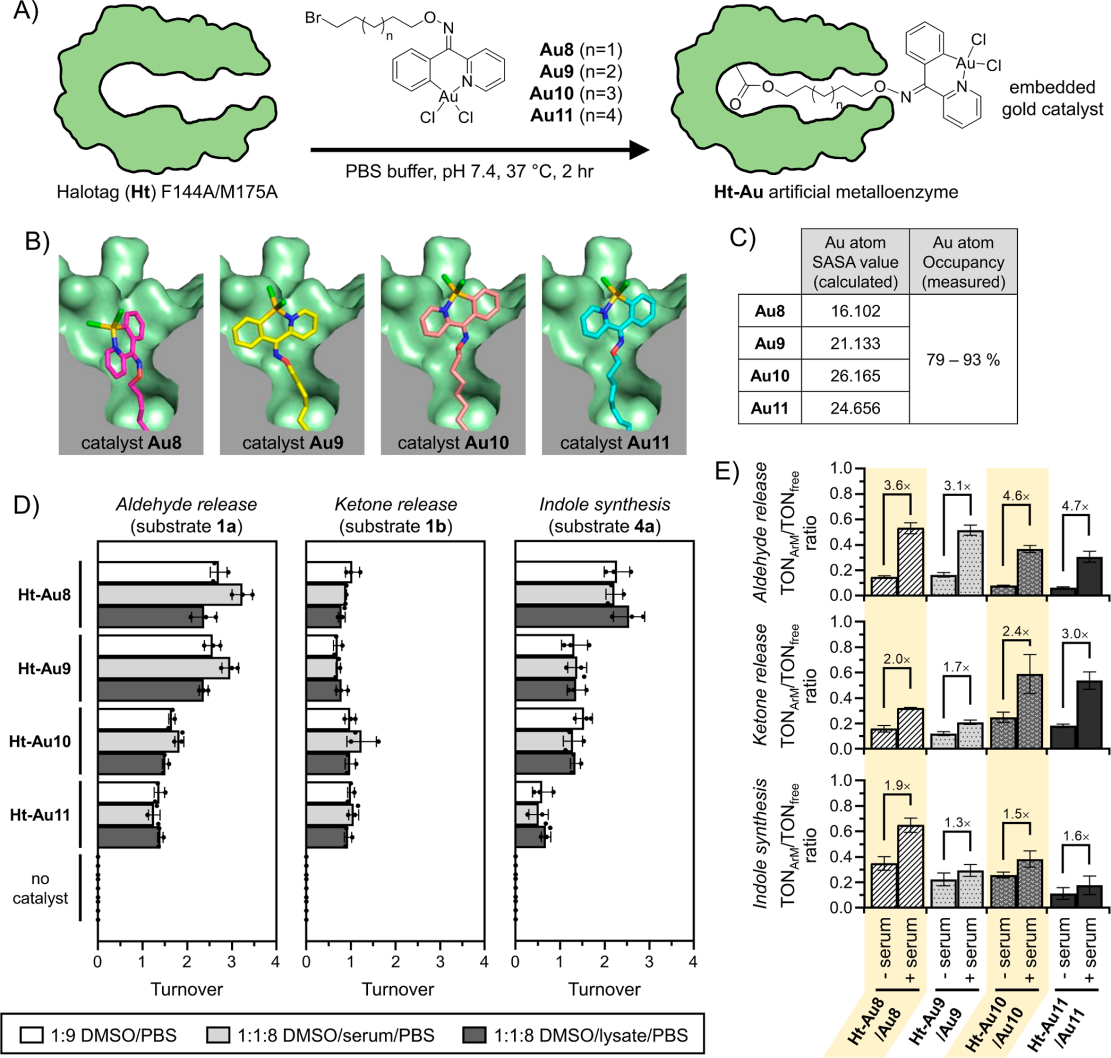
Investigations into the activity of Halotag ArMs. (A) To produce **Ht-Au** artificial metalloenzymes, the chloroalkane-containing **Au8–Au11** catalysts were incubated with **Ht** for 2 h at 37 °C to facilitate covalent bond attachment. (B) Covalent docking studies were conducted to determine the orientation and positioning of the gold catalyst moiety of **Au8–Au11** once anchored to Asp106 in the **Ht** binding pocket. The similar binding modes of **Au9–Au11** suggests that linker flexibility allows the gold moiety to adopt its most favorable orientation. (C) Table of the solvent accessibility calculations for each embedded gold catalyst, as well as the experimental occupancy of gold in each ArM quantitatively determined by ICP–MS. (D) Using the developed **Ht-Au** ArMs, turnovers were acquired for the PBO-based release of aldehydes from **1a**, ketones from **1b**, and the formation of indoles from **4b**. Reactions were incubated for 4 h in biologically relevant solvents using 2.5 mol % of **Ht-Au** ArMs. (E) As a comparative control, the TON ratios of reactions catalyzed by **Ht-Au** ArMs and the free gold catalysts **Au8–Au11** were acquired in PBS buffered media supplemented with and without serum. In all cases, the free catalysts in solution lost significant activity when exposed to serum, while **Ht-Au** ArMs maintained reactivity under similar conditions. Thus, the activities of the **Ht-Au** ArMs and the free gold catalysts **Au8–Au11** are generally closer in serum-containing containing media.

As a preliminary investigation into the effects of the haloalkane linker, covalent docking calculations were conducted to predict the conformation and solvent exposure of **Au8**–**Au11** once bound inside the HaloTag binding pocket (Figure 5B). In terms of the lengths from the Asp106 anchor point to the gold atom (Figure S14), the shortest distances were observed with **Au8** and **Au9** (15.7–15.9 Å), which was expected given their relatively shorter 5- and 6-carbon linkers, respectively. Interestingly, both **Au10** and **Au11** reach a similar length of 18.5 Å with their gold moieties having similar orientations in space. This suggests that with longer linkers, the added flexibility allows the gold moiety to possibly adopt its most favorable orientation. This statement can be further supported by analyzing the substrate accessibility of the embedded gold catalyst. According to the solvent-accessible surface area (SASA) values calculated for the gold atom in each of the **Au8**–**Au11** docked poses (Figure 5C, Table S11), bound **Au8** is predicted to be the least solvent available while longer chained **Au10** and **Au11** are equally found to be the most solvent available.

To experimentally confirm catalyst loading, **Ht-Au** ArMs were subjected to ICP–MS analysis. From a 5 μM ArM solution, Au atoms were detected in the range of 3.9–4.6 μg/mL, which would mean that **Ht-Au** ArMs produced in this study had a roughly 79–93% Au occupancy level (Figure 5C). Given the nearly 1:1 ratio of gold/protein loading, no normalization of the turnover was carried out for subsequent reactions comparing free to ArM-bound gold catalysts.

Moving forward, the catalytic capabilities of the **Ht-Au** ArMs were next tested (Figure 5D, Tables S12–S14). First, turnovers were acquired under mild conditions (10% DMSO/PBS buffer). For simple carbonyl release, substrate **1a** gave TONs in the range of 1.28–2.92, while substrate **1b** gave TONs between 0.63 and 1.21. To complete the investigation, indole formation for substrate **4b** was observed to proceed with TONs in the range of 0.42–2.59.

From this data, one major observation that can be made is that activity appeared to be generally higher with **Ht-Au** ArMs created with shorter linkers. For example, the shorter linker-containing **Ht-Au8** gave TONs between 2.58 and 2.92 (for aldehyde release) and 2.03–2.59 (for indole synthesis). Conversely, these TONS respectively drop to 1.28–1.37 and 0.42–0.85 for the longer linker-containing **Ht-Au11**. Surprisingly, ketone release did not fit the observed trend as all activity stayed consistently in the same range. This may simply be an outcome of the lower activity previously identified with PBO-based ketone release.

Another observation from these activity studies was that turnovers were generally lower when catalyzed by artificial metalloenzymes compared to the free gold catalysts in solution. This is evident from the low TON_ArM_/TON_free_ ratios (<0.25) consistently observed for all reaction types (Figure 5E). One minor explanation could be technical, where the high instability of the produced carbonyl groups may form imines with exposed lysine residues on HaloTag protein surfaces, thereby suppressing product detection. One major explanation could be the fact that the substrate binding pocket of the protein scaffold was never optimized by evolution. Thus, substrate binding is likely not strong, which is evident from the micromolar substrate *K*_M_ values obtained during kinetic studies (Table S15).

In the next step, investigations shifted toward testing the biocompatibility of the **Ht-Au** ArMs. To do this, tests were conducted using more biologically relevant conditions containing serum and cell lysate. Compared to reactions run under buffered conditions (Figure 5D), TONs acquired in serum- and lysate-containing conditions remained largely similar. For example, TONs were found in the range between 1.11 and 3.46 for aldehyde release (**1a**), 0.66 to 1.62 for ketone release (**1b**), and from 0.27–2.43 for indole synthesis (**4b**). As a control, it was confirmed that incubation of substrates **1a**, **1b**, or **4a** alone under similar conditions did not result in substrate consumption or any detectable traces of unwanted carbonyl release.

A further observation of note was that when using serum-containing media, TON_ArM_/TON_free_ ratios were all found to be increased (Figure 5E). That signifies that the activities obtained by the artificial metalloenzymes are closer to those obtained using free gold catalysts in solution. This situation suggests that while ArM activities are generally lower (due to the unevolved protein scaffold), the ArM protein scaffold may offer protection to the embedded gold catalyst from the surrounding environment. On the other hand, free gold catalysts exhibit higher reactivities in simple buffered conditions, but appear to be more easily quenched when exposed to biological media.

### Cell Imaging Studies to Confirm ArM Cancer Targeting

Moving forward, the cancer-targeting properties of **HtPA** were next investigated. To quantify cellular binding, fluorescent cell imaging was carried out using slightly modified protein complexes. As depicted in Figure 6A and Scheme S8, HaloTag-containing proteins were fluorescently labeled using an azido-PEG3-C6–Cl/DBCO-fluorescein conjugate (**FL**). In this manner, these proteins can be imbued with fluorescence, allowing for the observation and quantification of its cellular accumulation.

**Figure 6:**
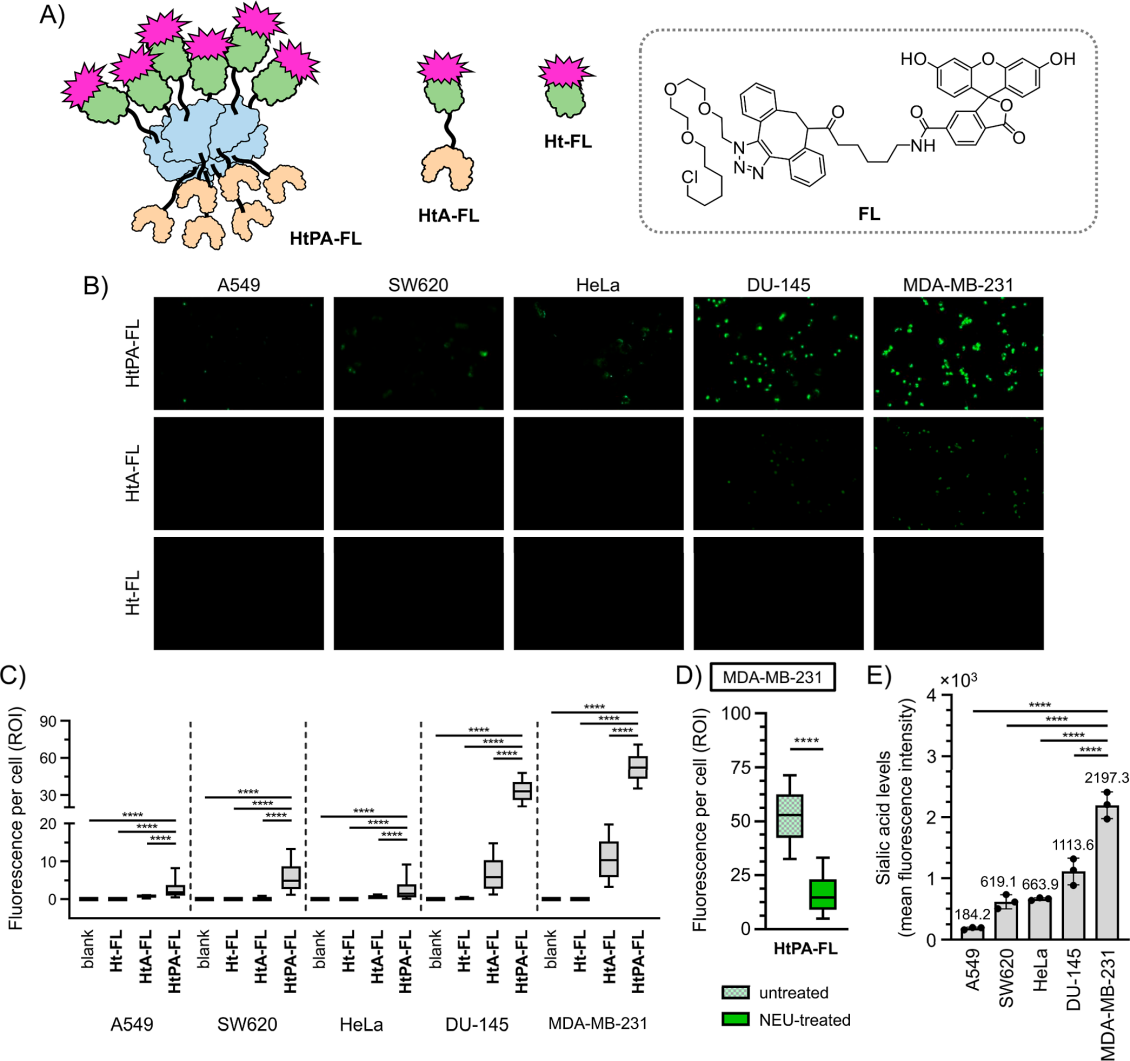
Characterization of **HtPA** targeting toward hypersialyated cancer cells. (A) Depiction of the **Ht-FL**, **HtA-FL**, and **HtPA-FL** proteins used for cell imaging studies. The **FL** probe was created through ligation of Azido-PEG3-C6–Cl and DBCO-FITC, which was in turn anchored into relevant protein scaffolds. **HtA-FL** is regarded as a control unable to benefit from multivalent lectin binding, while **Ht-FL** is regarded as a nontargeting control. (B) To quantify cellular binding via fluorescent imaging, the **Ht-FL**, **HtA-FL**, and **HtPA-FL** proteins (10 μM) were incubated with various cancer cell lines for 6 h, washed, fixed, and then analyzed. Shown are sample images obtained from the FITC channels. (C) Summary of imaging data to probe the cancer-targeting properties of **HtPA-FL** and relevant controls. Presented values are the mean fluorescence per cell observed among the different cell lines (*n* = 671 to 4472 ROIs). (D) As an additional control experiment, MDA-MB-231 cells were treated with or without neuraminidase (NEU) for 1 h before incubation with **HtPA-FL**. The relatively depressed mean fluorescence of **HtPA-FL** to NEU-treated cells (*n* = 932 to 1554 ROIs) can indirectly prove the importance of hypersialylation for targeting. (E) Summary of metabolic labeling studies done to determine total levels of surface accessible sialic acid among various cancer cell lines. Cells were first treated with Ac4ManNAz (40 μM), followed by labeling with DBCO-FITC (10 μM). Cell suspensions were then prepared and analyzed by FACS, where the mean fluorescence intensities indirectly correlate to sialic acid expression.

To perform cell imaging experiments, **HtPA-FL** was incubated with 5 different cancer cell types: MDA-MB-231 (breast), HeLa (cervical), SW620 (colorectal), DU-145 (prostate), and A549 (lung). Incubations were also done using a nontargeting control (i.e., **Ht-FL**), as well as a control that cannot take advantage of lectin multivalency (i.e., **HtA-FL**). With the blank control, cells were simply incubated with equivalent volumes of PBS buffer to set a baseline for autofluorescence. Following an incubation period of 6 h, fluorescent and brightfield images were acquired for all conditions (Figures 6B and S25–S29). In general, nonsignificant levels of fluorescence was detected from the blank, **Ht-FL**, and **HtA-FL** controls. However, **HtPA-FL** elicited much stronger signals for detection. A summary of **HtPA-FL** fluorescence is shown in Figure 6C. When ranking this data, it is clear to see that **HtPA-FL** exhibited the strongest binding to MDA-MB-231 cells. On the other hand, binding to A549 cells appeared to be the weakest.

As an added confirmation of the sialic-acid targeting properties of **HtPA**, cellular binding experiments were similarly run using cells pretreated with or without *Clostridium perfringens* neuraminidase (NEU). Since this enzyme is capable of cleaving α 2,3- and α 2,6-linked sialic acids from complex carbohydrates, the observation that **HtPA-FL** binds at a lowered capacity to NEU-treated MDA-MB-231 cells indirectly proves that hypersialylation is necessary for HtPA binding (Figure 6D, S30).

To consolidate the results obtained through fluorescent cell imaging, metabolic labeling experiments were next carried out to determine the relative expression levels of surface accessible sialic acid among the various tested cancer cell lines (Figure S24). To do this, cells were first metabolically incorporated with Ac_4_ManNAz so that cell surface sialic acids would be naturally decorated with an azide handle. Then by exposure to DBCO-fluorescein, these fluorescent probes could be covalently attached to the azide-containing sialic acid residues found on cell surfaces. Through analysis by fluorescence-activated cell sorting (FACS), the mean fluorescence intensity (MFI) per cell was then determined. A summary of these results is shown in Figure 6E. When ranking this data, MDA-MB-231 cells appear to produce the highest level of sialic acid (∼2197 MFI), while A549 cells showed the lowest levels (∼184 MFI). Interestingly, the ranked order of sialic acid production among the 5 tested cell lines roughly correlates to the ranked order of detected fluorescence observed during cell imaging studies. Thus, it can be strongly suggested that **HtPA** cellular binding is largely driven by the level of surface sialyation, since the ACG lectin component of **HtPA** is a known receptor of α 2,3-sialic acids.

### Validation of ArM Prodrug Therapy in Cell Assays

In the final stage of this study, cell viability assays were performed to validate the anticancer ArM prodrug therapy. To conduct these experiments, **HtPA-Au8** was prepared according to Scheme S7, which will allow formation of **HtPA** oligomeric complexes capable of shuttling the bound gold catalyst to hypersialylated cancer cells. To confirm **HtPA** self-assembly, a solution run through size exclusion chromatography gave a peak corresponding to the size of a hexameric protein complex (Figure S19).

Another important aspect to consider for any prodrug therapy is the choice of bioactive drug to be released, which in this study requires carbonyl groups to make use of PBO chemistry. At present, ketone-containing drugs are the best option, as they are widely present in a number of FDA-approved drugs (i.e., doxorubicin, haloperidol, tolcapone, ganaxolone, etc.). On the other hand, aldehydes are much too reactive to be commonly found on FDA-approved drugs. Instead, aldehydes serve the most utility as a chemical warhead in several literature examples of suicide inhibitors. In publications spanning from 2001 to 2022,^98^ aldehydes were the third most used reactive warhead used (falling only behind α,β-unsaturated carbonyls and epoxides).

In this study, the drug chosen to be used for PBO-based prodrug therapy is phenstatin **9** (Figure 7A), which is a combretastatin A4 analogue that replaces the ethylene bridge with a ketone.^99^ Studies have shown this structural change can help to increase metabolic stability,^100^ while also maintaining potent biological activity. Acting similarly to other antimitotic agents, phenstatin works by binding the colchicine binding site at the interface of αβ-tubulin heterodimers, leading to the disruption of microtubule assembly and cell division arrest.^101^ Synthesized according to Scheme S6, PBO prodrug **10** was designed so that gold-catalyzed removal of the PBO group could eventually lead to the release of phenstatin.

**Figure 7:**
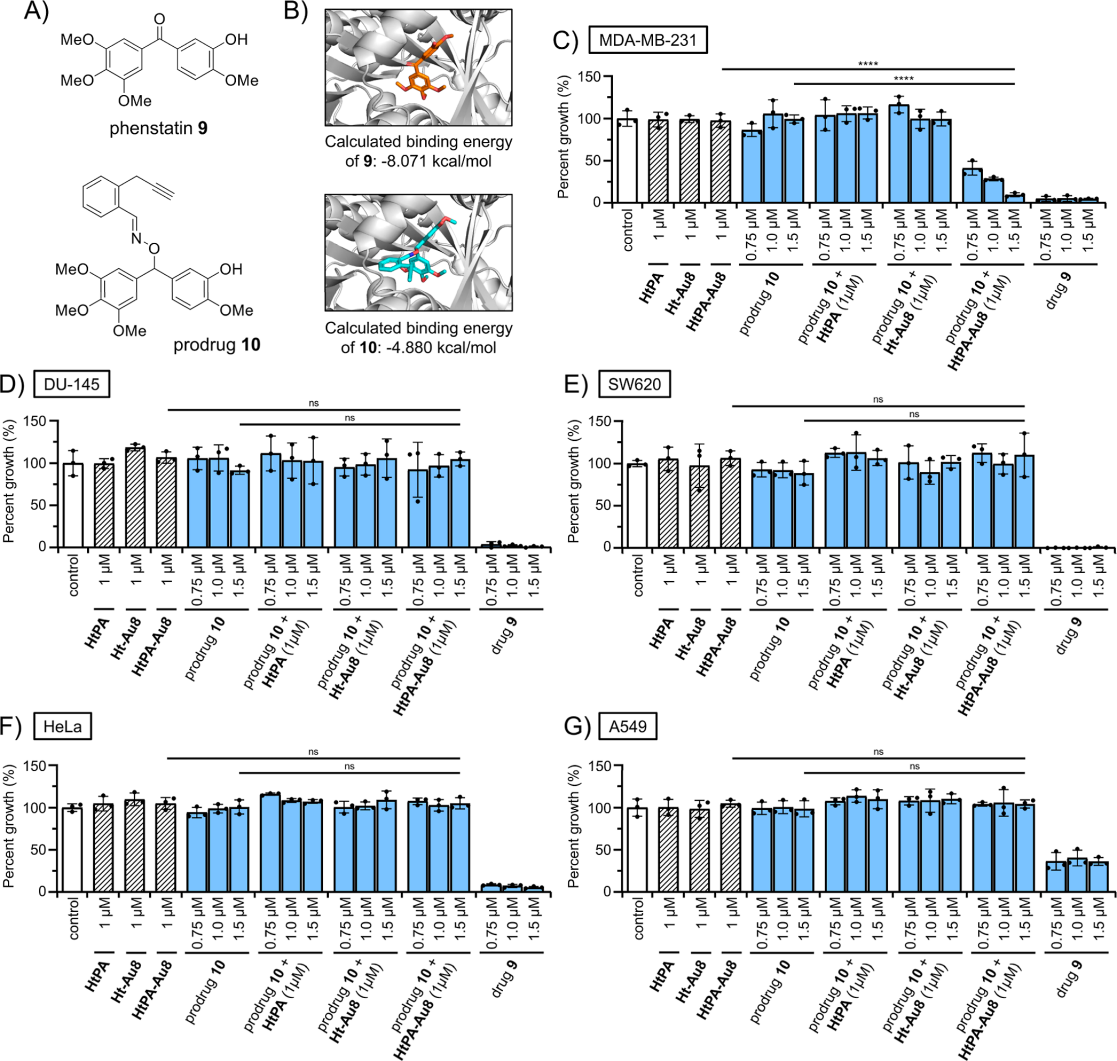
Investigation of an anticancer prodrug strategy designed for localized, gold-activation of a PBO-based prodrug using a hypersialylated cancer-targeting **HtPA-Au8** ArM system. (A) Structures of relevant compounds to this study. Phenstatin **9** is known to exert its anticancer effects through inhibition of tubulin polymerization. Prodrug **10** was designed so that a PBO group can mask the ketone group of phenstatin **9**. (B) Molecular docking studies were performed to probe differences in theoretical binding to αβ-tubulin, which showed that the calculated binding energy of prodrug **10** is significantly weaker than phenstatin **9**. Cell viability experiments were conducted using the developed ArM prodrug therapy against various cancer cell lies, which include (C) MDA-MB-231, (D) DU-145, (E) SW620, (F) HeLa, and (G) A549. Mixtures of prodrug **10** (0.75 to 1.5 μM) and **HtPA-Au8** (1 μM) were shown to exert significantly stronger cytotoxic activities in hypersialylated MDA-MB-231 cells compared to controls (prodrug only or ArM only). In addition, control treatments using prodrug mixtures with **HtPA** (lacking gold control) or **Ht-Au8** (lacking targeting) showed no activity. For the other tested cell lines that possess lower levels of sialylation, no activity was observed.

As a preliminary test to probe whether prodrug **10** could have weaker bioactivity (due to reduced αβ-tubulin binding), modeling studies were carried out (Figures 7B, S15–S17). This docking experiment generated a configuration of prodrug **10** with a calculated binding affinity of −4.880 kcal/mol. Since this value is significantly lower compared to a similar pose obtained with **9** (−8.071 kcal/mol), it can be thus suggested that prodrug **10** has reduced bioactivity compared to its activated drug counterpart.

Finally, to validate the anticancer ArM prodrug therapy developed in this study, cytotoxicity assays were performed with all cell lines examined in this study (Figure 7C–G). In a preliminary test using MDA-MB-231 as the model cell line (Figure S31A), the IC_50_ for prodrug **10** was determined to be 4.7 μM, which represented a 167-fold decrease in activity compared to the drug (IC_50_ of **9** is 28 nM). This data suggests that the added steric bulk of the PBO group is likely responsible for disrupting prodrug binding to its αβ-tubulin target. In addition, **HtPA-Au8** alone was tested, which did not show any cytotoxic effects up to 2 μM (Figure S31B).

Finally, to validate the ArM prodrug therapy, all cell lines were sequentially treated with **HtPA-Au8** (1 μM) and prodrug **10** (0.75 to 1.5 μM). From this data, only with the highly hypersialylated MDA-MB-231 cancer cell line is there a dose-dependent decrease in cell viability when exposed to the ArM prodrug therapy. For example, a mixture of 1 μM **HtPA-Au8** and 1.5 μM prodrug **10** was found to reduce MDA-MB-231 cell viability by 92% (compared to controls). Drug formation was confirmed through LC–MS analysis of the lysate obtained from treated cells (Figure S32). In terms of controls, equivalent concentrations of prodrug only or ArM only had no effect on cell viability. Control treatments were also run using prodrug mixtures with a gold-deficient control (i.e., **HtPA**) and a nontargeting control (**Ht-Au8**). In all these control mixtures, no significant reduction of MDA-MB-231 cell viability could be observed.

To complete the experiment, similar treatment conditions were also applied to the DU-145, HeLa, SW620, and A549 cell lines. From this data, no observable decreases in cell viability were found, which is likely linked to poorer targeting due to the lower levels of sialylation present in these cancer cells. Collectively, these results highlight the potential utility of **HtPA** ArM prodrug therapy as a way to target hypersialylated cancers.

## Conclusions

In conclusion, this study developed two main concepts (multivalent lectin-directed artificial metalloenzymes and a gold-catalyzed aldehyde releasing reaction) that were successfully adapted into a working example of an anticancer ArM prodrug therapy. Furthermore, PBO cleavage chemistry was shown to proceed under biological conditions, thereby allowing the catalytic release of carbonyl-containing molecules under mild and physiological conditions. This is a significant discovery as bioorthogonal release reactions for aldehyde/ketone moieties have thus far been limited to only isonitrile click chemistry.^102–104^ Additionally, adapting aldehyde release from a PBO prodrug to synthesize an indole-based drug allows this study to join a growing list of studies that have developed synthetic prodrugs activated by abiotic transition metals.^38,39,41,67,105–112^ Overall, we believe this work will not only be a useful addition to the growing library of new-to-nature reactions, but also represents an alternative strategy in the development of cancer targeting ArM biocatalysts. Our aim is to continue building on this platform to further develop and improve this ArM system to make use of the unique properties of gold-catalyzed reactions. For example, to address any potential adverse immunological responses, protein components of the ArM could be replaced with human-sourced proteins that fulfill similar roles. Additionally, different lectins could be installed to target other glycans (i.e., fucose, high mannose) that are overexpressed in other cancer types. Since one weakness of the **HtPA** ArMs is its lowered activity compared to the free metal, efforts could be made to either improve the substrate binding pocket of the HaloTag protein through directed evolution, or to explore other protein scaffolds that could improve catalytic yields. Furthermore, efforts could also focus on improving catalytic efficiency by screening other types of gold catalysts.^113,114^ Overall, there are numerous avenues to continually improve upon this ArM prodrug system, which can hopefully lead to the creation of a viable anticancer therapy in the future.

## Supplementary Material



## Notes

Detailed materials and methods, compound synthesis and characterization, ^1^H and ^13^C NMR spectra, experimental procedures for biological studies, and supplementary figures, tables, and schemes, including Figures S1–S32, Tables S1–S15, and Schemes S1–S8 (PDF)
