# Supplementary material for: Gold-Catalyzed Carbonyl Release and its Adaptation for Prodrug Therapy Using Multivalent Lectin-Directed Artificial Metalloenzymes
Source: JACS Au. 2026 Jan 12;6(1):389–402. doi: 10.1021/jacsau.5c01331 (PMC12848676; doi:10.1021/jacsau.5c01331)
Supplement: Supplementary file 1 [file au5c01331_si_001.pdf]

Supporting Information

Gold-catalyzed carbonyl release and its adaptation for prodrug therapy using multivalent lectin-directed artificial metalloenzymes

Jing Huang,[1#] Yiling Liu,[1#] Yufei Li,[1] Jianghui Du,[1] Xiao Han,[1] Kenward Vong[1*]

[1] Department of Chemistry, The Hong Kong University of Science and Technology, Clear Water Bay, Kowloon, Hong Kong, China

Table of Contents

1. Design analysis 2
2. Reactivity studies 3
   2.1 General information 3
   2.2 Synthetic protocols 3
      2.2.1 Model substrate preparation 3
      2.2.2 Catalyst preparation 23
      2.2.3 Prodrug preparation 27
   2.3 HPLC methods and standard curves 31
   2.4 Reactivity screening using transition metal catalysts 37
3. Modelling studies 46
   3.1 General information 46
   3.2 HaloTag cavity opening analysis 46
   3.3 Docking of gold catalysts to the HaloTag protein 47
   3.4 SASA analysis of bound gold catalysts 48
   3.5 Docking studies to predict tubulin binding properties of prodrug 48
4. Protein preparations and experiments 50
   4.1 General information 50
   4.2 Recombinant protein expression and purification 50
   4.3 Analysis of protein complexation 51
   4.4 Artificial metalloenzyme preparation 51
   4.5 Artificial metalloenzyme characterization 52
   4.6 Reactivity studies using artificial metalloenzymes 52
   4.7 Kinetic studies using artificial metalloenzymes 56
   4.8 Protein complexes used for cell imaging studies 59
5. Cell-based Assays 60
   5.1 General cell culture 60
   5.2 Statistical analysis 60
   5.3 Fluorescence activated cell sorting experiments 61
   5.4 Cell imaging studies 62
   5.5 Cell cytotoxicity studies 69
   5.6 LC/MS analysis 70
6. NMR spectra 71
7. References 137

1. Design analysis

In the planning of this project, the 3D structure of the recombinant **HtPA** fusion protein and its hexameric complex were considered (Figure S1). Due to the fact that the N- and C-terminuses of PduU point in opposing directions, this will ensure the HaloTag protein (linked to the N-terminus) and ACG lectin (linked to the C-terminus) are adequately separated. Once oligomerization of the PduU monomer protein occurs, it is expected that the long linker lengths should promote minimal interference between the three core components: PduU hexamer, glycan-binding ACG lectin, and the gold-embedded HaloTag protein. Furthermore, PduU complexation will occur in a highly specific orientation, thereby creating a lectin-only side, as well as a HaloTag-only side.

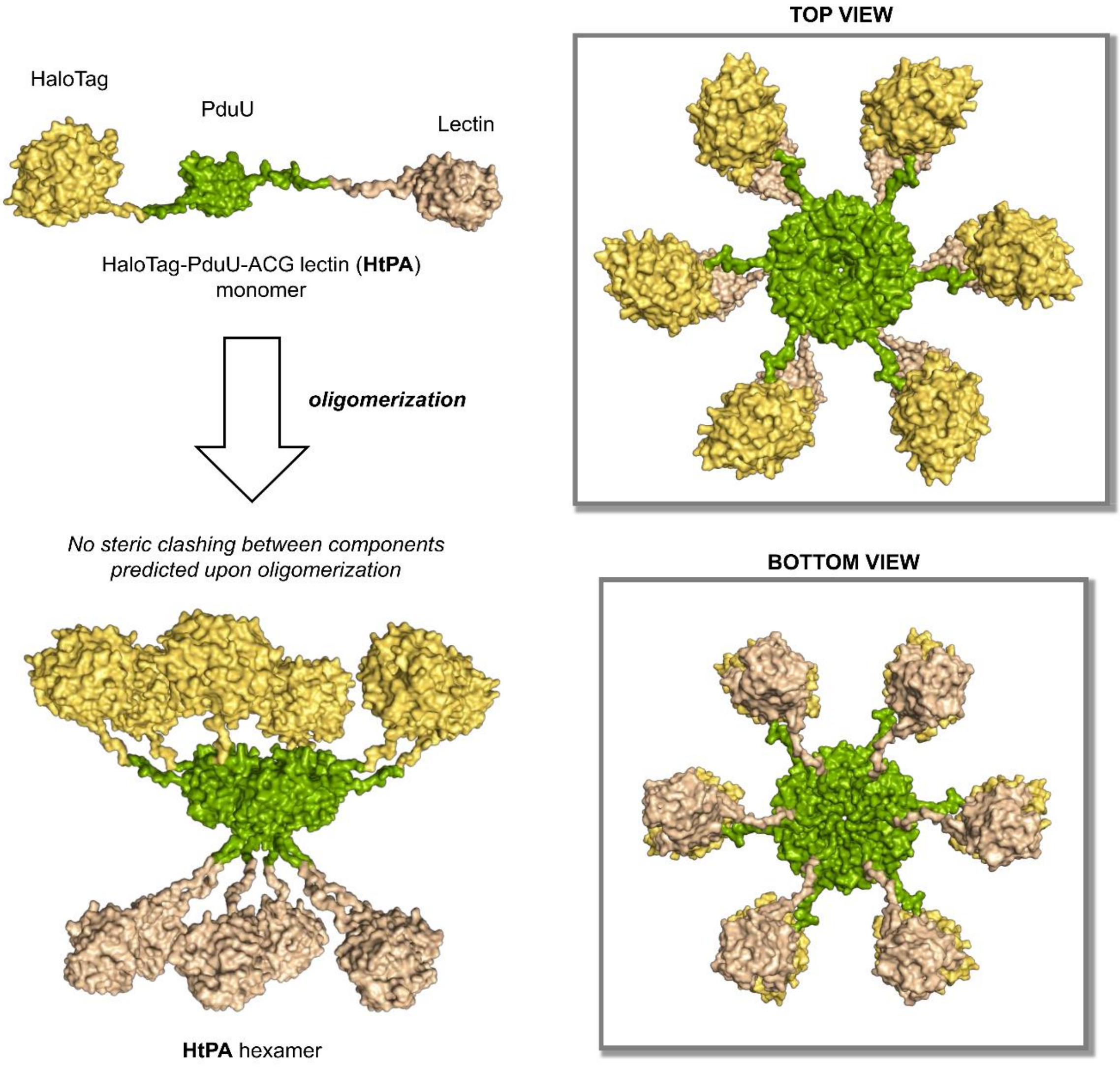

Figure S1. Design analysis of the recombinant **HtPA** fusion protein and its hexameric complex. Protein images were generated from the following Protein Data Bank files: HaloTag protein from 6U32, PduU shell protein from 3CGI, and ACG lectin from 1WW4.

2. Reactivity studies

2.1 General information

All reagents and buffer components were purchased from Sigma-Aldrich, TCI chemicals, JK Chemicals, or Macklin without further purification. TLC analysis (F-254) was performed with 60 Å silica gel from Merck Millipore. ^{1}H and ^{13}C NMR spectra were measured on a Bruker AVIII-400 spectrometer (400 MHz) with the solvent peaks as internal standards. High-resolution mass spectroscopy (HRMS) measurements were carried out at the Hong Kong University of Science and Technology Mass Spectrometry Service Center on either an Agilent GC/MS 5975C system or an API QSTAR XL System.

2.2 Synthetic protocols

2.2.1 Model substrate preparation

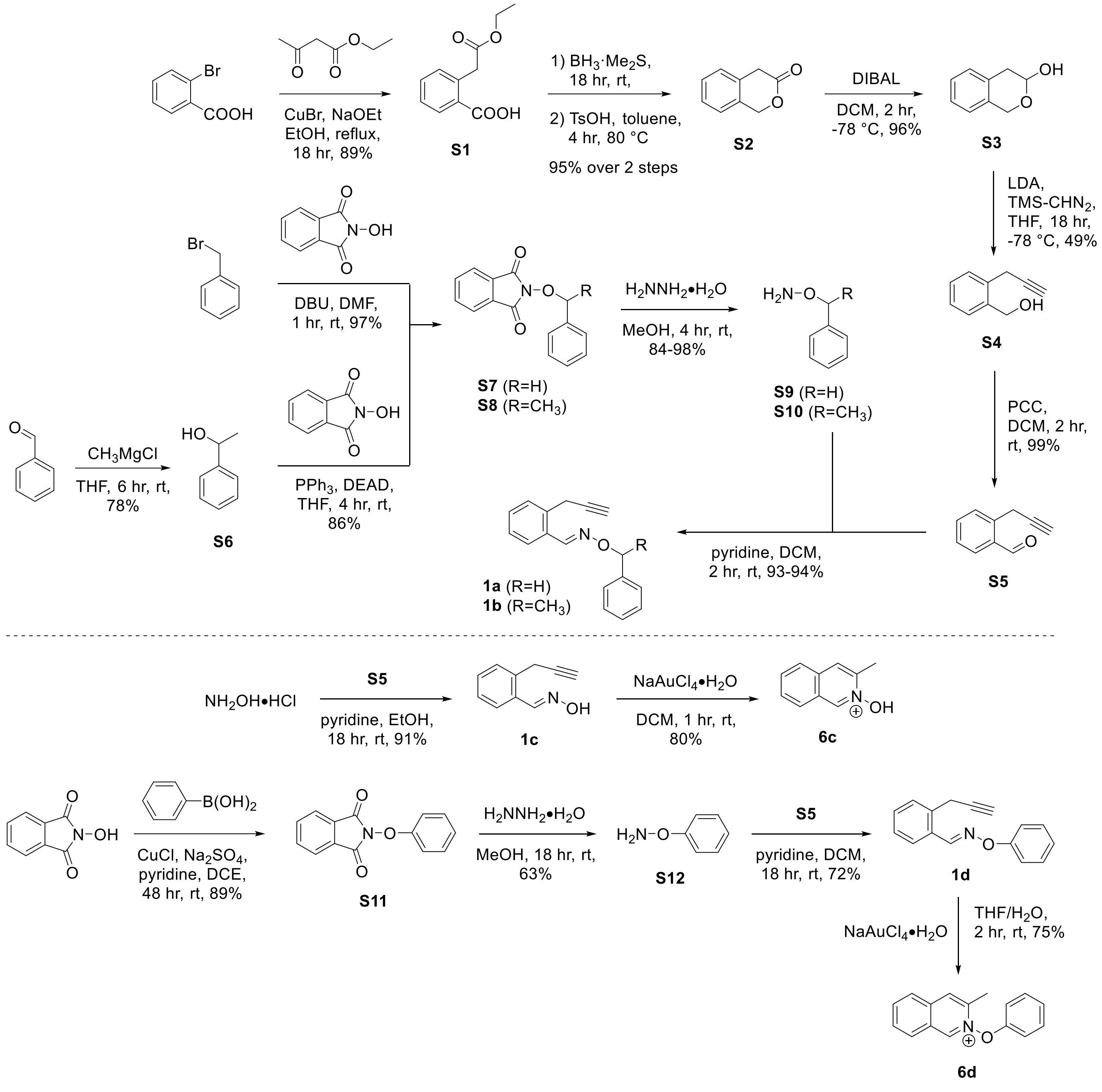

Scheme S1. Synthesis of compounds **1a-d**

Preparation of **S1**

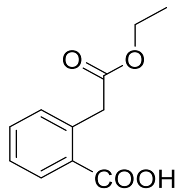

To a dry flask was charged with NaOEt (2.03 g, 29.85 mmol) under argon. EtOH (30 mL) was added and stirred for 5 min until the suspension become a clear solution. Ethyl acetoactate (2.54 mL, 19.90 mmol) was added and the mixture was stirred at room temperature for 10 min. Then, 2-bromobenzoic acid (2.00 g, 9.95 mmol) and CuBr (0.14 g, 0.99 mmol) were added, and the reaction mixture was refluxed under argon. After overnight stirring, the reaction mixture was concentrated in vacuo, acidified by 2.0 M HCl (30 mL), and then extracted with EtOAc (40 mL). The organic phase was washed with H_2O and brine, dried over Na_2SO_4. The solution was then concentrated under vacuum to around 5 mL EtOAc in flask. The solution was then stirred at 45 °C and n-hexane (10 mL) was added dropwise. After slowly cooling down to 0 °C, a solid crashed out and filtered to afford **S1** as a white solid (1.85g, 89%). R_f = 0.30 (EtOAc/Hex, 3:7). ^{1}H NMR ($CDCl_3$, 400 MHz): δ 8.13 (dd, *J* = 7.9, 1.5 Hz, 1H), 7.54 (td, *J* = 7.5, 1.5 Hz, 1H), 7.40 (td, *J* = 7.7, 1.3 Hz, 1H), 7.29 (dd, *J* = 7.7, 1.3 Hz, 1H), 4.17 (q, *J* = 7.1 Hz, 2H), 4.04 (s, 2H), 1.26 (t, *J* = 7.1 Hz, 3H). ^{13}C NMR ($CDCl_3$, 101 MHz): δ 171.77, 171.70, 136.96, 133.37, 132.54, 132.00, 128.80, 127.69, 60.99, 40.95, 14.32. HRMS for $C_{11}H_{13}O_4$ $[M+H]^+$ calcd. 209.0814, found 209.0813.

Preparation of **S2**

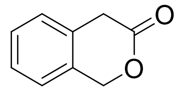

To a stirred solution of **S1** (1.55g, 7.44 mmol) in THF (10 mL) was added $BH_3{\cdot}Me_2S$ (8.19 mL, 16.38 mmol, 2.0 M in THF) at 0 °C under argon. After overnight stirring, the reaction was quenched with saturated NH_4Cl solution (15 mL) and extracted with EtOAc (2 × 30 mL). The organic phase was washed with H_2O and brine, then dried over Na_2SO_4 and concentrated. The crude oil was dissolved in toluene (5 mL) and *p*-toluenesulfonic acid (12.7 mg, 0.07 mmol) was added. The reaction mixture was stirred at 80 °C for 4 hr to push the alcohol intermediate into the desired lactone. After cooling down to room temperature, the solution was diluted with EtOAc (20 mL), washed with H_2O and brine, and then dried over Na_2SO_4. The solution was then concentrated under vacuum to afford **S2** as a solid (1.05g, 95%). R_f = 0.32 (EtOAc/Hex, 3:7). ^{1}H NMR ($CDCl_3$, 400 MHz): δ 7.33 (dtd, *J* = 16.1, 7.4, 1.6 Hz, 2H), 7.26 – 7.21 (m, 2H), 5.32 (s, 2H), 3.72 (s, 2H). ^{13}C NMR ($CDCl_3$, 101 MHz): δ 170.83, 131.70, 131.12, 128.99, 127.54, 127.25, 124.84, 70.25, 36.36. HRMS for $C_9H_8NaO_2$ $[M+Na]^+$ calcd. 171.0422, found 171.0422.

Preparation of **S3**

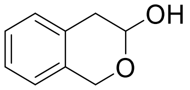

To a solution of **S2** (1.01g, 6.82 mmol) in anhydrous DCM (10 mL) was added diisobutylaluminium hydride (13.63 mL, 13.63 mmol, 1.0 M in THF) under argon at -78 °C. After stirring at -78 °C for 2 hr, the reaction was quenched with MeOH (5 mL) at -78 °C. Then a saturated potassium sodium tartrate solution (20 mL) was added and the mixture continued to be stirred for 1 hr. The mixture was extracted with DCM (2 × 30 mL). The organic phase was washed with H_2O and brine, dried over Na_2SO_4, and then concentrated under vacuum to afford **S3** as a solid (0.98 g, 96%). R_f = 0.28 (EtOAc/Hex, 3:7). ^{1}H NMR ($CDCl_3$, 400 MHz): δ 7.19 – 7.11 (m, 2H), 7.11 – 7.05 (m, 1H), 6.97 (dd, *J* = 6.3, 2.6 Hz, 1H), 5.29 (dd, *J* = 5.5, 3.8 Hz, 1H), 4.98 – 4.67 (m, 2H), 4.55 (s, 1H), 3.07 – 2.73 (m, 2H). ^{13}C NMR ($CDCl_3$, 101 MHz): δ 133.68, 131.22, 129.12, 126.84, 126.23, 123.95, 92.49, 63.94, 34.65. HRMS for $C_9H_{10}NaO_2$ $[M+Na]^+$ calcd. 173.0578, found 173.0573.

Preparation of **S4**

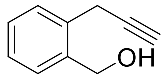

(Trimethylsilyl)diazomethane (4.1 mL, 8.21 mmol, 2.0 M in hexane) was added dropwise to a solution of lithium diisopropylamide (8.21 mL, 16.41 mmol, 2.0 M in THF) at -78 °C under argon. After 30 min of stirring at -78 °C, a solution of **S3** (948 mg, 6.31 mmol) in anhydrous THF (2 mL) was added to the reaction mixture at -78 °C. After stirring at -78 °C for 30 min, the reaction mixture was slowly warmed to room temperature. After overnight stirring, the reaction was quenched with saturated NH_4Cl solution (20 mL) and extracted with EtOAc (2 × 30 mL). The organic phase was washed with H_2O and brine, dried over Na_2SO_4, and then concentrated under vacuum. Flash column chromatography with a gradient of 2-10% EtOAc/Hex was used to purify the desired compound **S4** (450 mg, 49%). R_f = 0.40 (EtOAc/Hex, 3:7). ^{1}H NMR ($CDCl_3$, 400 MHz): δ 7.45 (dd, *J* = 7.0, 1.9 Hz, 1H), 7.34 (dd, *J* = 6.8, 2.1 Hz, 1H), 7.25 (ddd, *J* = 11.8, 6.7, 3.6 Hz, 2H), 4.71 (s, 2H), 3.64 (d, *J* = 2.7 Hz, 2H), 2.17 (t, *J* = 2.8 Hz, 1H). ^{13}C NMR ($CDCl_3$, 101 MHz): δ 138.29, 134.65, 129.18, 128.65, 128.47, 127.47, 82.08, 70.97, 63.35, 22.20. HRMS for $C_{10}H_{10}NaO$ $[M+Na]^+$ calcd. 169.0629, found 169.0629.

Preparation of **S5**

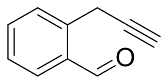

To a solution of **S4** (295 mg, 2.02 mmol) in DCM (10 mL) was added pyridiniumchlorochromate (1.30 g, 6.05 mmol) and silica gel (1.30 g). After stirring for 2 hr at room temperature, the reaction mixture was run through a short silica gel column. The solution was concentrated under vacuum to afford **S5** (290 mg, 99%). R_f = 0.55 (EtOAc/Hex, 1:9). ^{1}H NMR ($CDCl_3$, 400 MHz): δ 10.19 (s, 1H), 7.81 (dd, *J* = 7.6, 1.5 Hz, 1H), 7.74 (dd, *J* = 7.7, 1.2 Hz, 1H), 7.59 (td, *J* = 7.6, 1.5 Hz, 1H), 7.47 (td, *J* = 7.5, 1.2 Hz, 1H), 4.08 (d, *J* = 2.7 Hz, 2H), 2.26 (t, *J* = 2.7 Hz, 1H). ^{13}C NMR ($CDCl_3$, 101 MHz): δ 192.93, 138.03, 134.13, 134.07, 133.28, 129.96, 127.53, 81.26, 71.89, 22.75. HRMS for $C_{10}H_8NaO$ $[M+Na]^+$ calcd. 167.0473, found 167.0478.

Preparation of **S6**

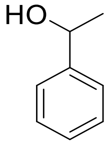

Methylmagnesium chloride (3.92 mL, 11.76 mmol, 3.0 M in THF) was added dropwise to a solution of benzylaldehyde (1.00 mL, 9.80 mmol) in anhydrous THF at 0 °C under argon. After 30 min of stirring at 0 °C, the reaction was then allowed to warm to room temperature and continued to stir for an additional 6 hr. The reaction was quenched with saturated NH_4Cl solution (15 mL) and extracted with EtOAc (30 mL). The organic phase was washed with H_2O and brine, dried over Na_2SO_4, and then concentrated under vacuum. Flash column chromatography (5% EtOAc/Hex) was used to purify the desired compound **S6** (930 mg, 78%). R_f = 0.11 (EtOAc/Hex, 1:9). ^{1}H NMR ($CDCl_3$, 400 MHz): δ 7.38 – 7.31 (m, 4H), 7.29 – 7.23 (m, 1H), 4.86 (qd, *J* = 6.5, 2.8 Hz, 1H), 2.08 (d, *J* = 3.3 Hz, 1H), 1.48 (d, *J* = 6.5 Hz, 3H). ^{13}C NMR ($CDCl_3$, 101 MHz): δ 145.93, 128.59, 127.56, 125.50, 70.48, 25.24. HRMS for $C_8H_{10}NaO$ $[M+Na]^+$ calcd. 145.0629, found 145.0630.

Preparation of **S7**

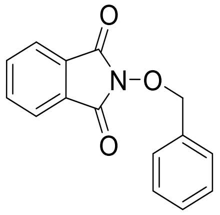

Bromobenzene (0.80 mL, 6.74 mmol) and DBU (1.10 mL, 7.36 mmol) were added to a solution of N-Hydroxyphthalimide (1.00 g, 6.13 mmol) in DMF (7 mL). After 30 min stirring at room temperature, the reaction mixture was acidified with 1.0 M HCl (20 mL). The precipitate was filtered and washed with H_2O (5 mL) to afford **S7** (1.50g, 97%). ^{1}H NMR ($CDCl_3$, 400 MHz): δ 7.81 (dd, *J* = 5.5, 3.1 Hz, 2H), 7.73 (dd, *J* = 5.5, 3.1 Hz, 2H), 7.58 – 7.48 (m, 2H), 7.43 – 7.34 (m, 3H), 5.21 (s, 2H). ^{13}C NMR ($CDCl_3$, 101 MHz): δ 163.62, 134.56, 133.80, 130.03, 129.48, 128.99, 128.68, 123.62, 80.00. HRMS for $C_{15}H_{11}NNaO_3$ $[M+Na]^+$ calcd. 276.0637, found 276.0635.

Preparation of **S8**

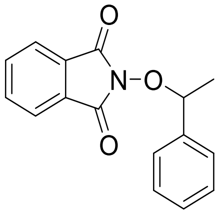

Diethyl azodicarboxylate (1.16 mL, 7.37 mmol) was added dropwise to a solution of **S6** (900 mg, 7.37 mmol), N-Hydroxyphthalimide (1.20 g, 7.37 mmol) and triphenylphosphine (2.13 g, 8.10 mmol) in anhydrous THF (10 mL) under argon at 0 °C. After stirring overnight at room temperature, the reaction was diluted with EtOAc, washed with H_2O and brine, dried over Na_2SO_4, and then concentrated under vacuum. Flash column chromatography with a gradient of 10-20% EtOAc/Hex was used to purify the desired compound **S8** (1.70 g, 86%). R_f = 0.49 (EtOAc/Hex, 1:4). ^{1}H NMR ($CDCl_3$, 400 MHz): δ 7.77 – 7.65 (m, 4H), 7.53 – 7.48 (m, 2H), 7.37 – 7.30 (m, 3H), 5.50 (q, *J* = 6.6 Hz, 1H), 1.72 (d, *J* = 6.6 Hz, 3H). ^{13}C NMR ($CDCl_3$, 101 MHz): δ 163.94, 139.12, 134.42, 129.11, 128.95, 128.49, 127.76, 123.49, 85.26, 20.58. HRMS for $C_{16}H_{13}NNaO_3$ $[M+Na]^+$ calcd. 290.0793, found 290.0795.

Preparation of **S9**

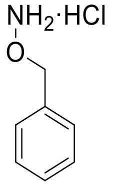

Hydrazine hydrate (1.14 mL, 18.36 mmol, 50%) was added to a suspension of **S7** (1.55g, 6.12 mmol) in MeOH (10 mL) at room temperature. After overnight stirring, the white solid was filtered off. The filtrate was concentrated under vacuum. The residue was dissolved in Et_2O (30 mL) and washed with H_2O and brine. Conc. HCl (4 mL) was added to the organic phase and a white solid crashed out. After filtration, the white solid was collected to afford compound **S9** (961 mg, 98%). ^{1}H NMR (MeOD, 400 MHz): δ 7.48 – 7.40 (m, 5H), 5.04 (s, 2H). 13C NMR (MeOD, 101 MHz): δ 134.31, 130.74, 130.50, 129.98, 78.18. HRMS for $C_7H_{10}NO$ $[M+H]^+$ calcd.124.0762, found 124.0754.

Preparation of **S10**

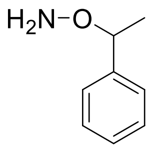

Hydrazine hydrate (1.83 mL, 18.86 mmol, 50%) was added to a solution of **S8** (1.68g, 6.29 mmol) in DCM (3 mL) / MeOH (15 mL) at room temperature. After overnight stirring, the white solid was filtered off. The filtrate was then concentrated under vacuum, redissolved in EtOAc (30 mL), and washed with H_2O and brine. The solution was then dried over Na_2SO_4 and concentrated under vacuum to afford compound **S10** (725 mg, 84%). R_f = 0.41 (EtOAc/Hex, 1:4). ^{1}H NMR ($CDCl_3$, 400 MHz): δ 7.38 – 7.25 (m, 5H), 5.18 (s, 2H), 4.62 (q, *J* = 6.6 Hz, 1H), 1.41 (d, *J* = 6.6 Hz, 3H). ^{13}C NMR ($CDCl_3$, 101 MHz): δ 143.00, 128.56, 127.73, 126.33, 82.85, 21.87. HRMS for $C_8H_{12}NO$ $[M+H]^+$ calcd.138.0919, found 138.0910.

Preparation of **S11**

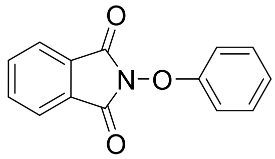

N-Hydroxyphthalimide (200 mg, 1.23 mmol), phenylboronic acid (299 mg, 2.45 mmol), copper(I) chloride (121 mg, 1.23 mmol) and Na_2SO_4 (871 mg, 6.13 mmol) were dissolved in DCE (6 mL). After 48 h stirring at room temperature, the solid was filtered off. The filtrate was then concentrated under vacuum. Flash column chromatography with 20% EtOAc/Hex was used to purify the desired compound **S11** (260 mg, 89%). ^{1}H NMR (400 MHz, Chloroform-*d*) δ 7.90 (dd, *J* = 5.5, 3.1 Hz, 2H), 7.84 – 7.76 (m, 2H), 7.39 – 7.28 (m, 2H), 7.20 – 7.09 (m, 3H). ^{13}C NMR (101 MHz, $CDCl_3$) δ 163.01, 158.93, 135.00, 129.83, 128.85, 124.68, 124.05, 114.46. HRMS for $C_{14}H_9NNaO_3$ $[M+Na]^+$ calcd.262.0480, found 262.0494.

Preparation of **S12**

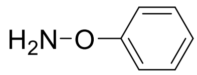

Hydrazine hydrate (0.32 mL, 3.26 mmol, 50%) was added to a solution of **S11** (260 mg, 1.09 mmol) in MeOH (5 mL) at room temperature. After overnight stirring, the reaction mixture was diluted with EtOAc, washed with H_2O and brine. The solution was then dried over Na_2SO_4 and concentrated under vacuum. Flash column chromatography with 20% EtOAc/Hex was used to purify the desired compound **S12** (75 mg, 63%). ^{1}H NMR (400 MHz, Chloroform-*d*) δ 7.34 – 7.27 (m, 2H), 7.19 – 7.13 (m, 2H), 6.97 (tt, *J* = 7.2, 1.2 Hz, 1H), 5.84 (s, 2H). ^{13}C NMR (101 MHz, $CDCl_3$) δ 161.36, 129.32, 121.19, 113.22. HRMS for C_6H_6NNaO $[M+Na]^+$ calcd.132.0425, found 132.0435.

Preparation of **1a**

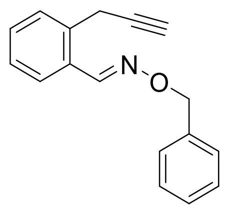

Pyridine (0.22 mL, 2.77 mmol) was added to a solution of **S5** (100 mg, 0.69 mmol) and **S9** (133 mg, 0.83 mmol) in DCM (3 mL) at room temperature. After stirring for 2 hr, the solution was concentrated under vacuum. Flash column chromatography with a gradient of 0-1% EtOAc/Hex was used to purify the desired compound **1a** (160 mg, 93%). R_f = 0.66 (EtOAc/Hex, 1:9). ^1H NMR ($CDCl_3$, 400 MHz): δ 8.42 (s, 1H), 7.64 (dd, *J* = 7.6, 1.5 Hz, 1H), 7.56 (dd, *J* = 7.7, 1.3 Hz, 1H), 7.48 – 7.42 (m, 2H), 7.42 – 7.26 (m, 5H), 5.24 (s, 2H), 3.75 (d, *J* = 2.7 Hz, 2H), 2.23 (t, *J* = 2.7 Hz, 1H). ^{13}C NMR ($CDCl_3$, 101 MHz): δ 148.14, 137.58, 134.83, 130.00, 129.96, 129.40, 128.65, 128.59, 128.46, 128.16, 127.31, 81.51, 76.66, 71.57, 23.52. HRMS for $C_{17}H_{15}NNaO$ $[M+Na]^+$ calcd. 272.1051, found 272.1047.

Preparation of **1b**

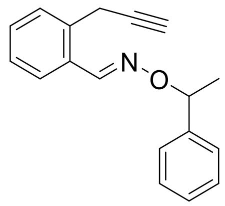

Pyridine (0.22 mL, 2.77 mmol) was added to a solution of **S5** (100 mg, 0.69 mmol) and **S10** (114 mg, 0.83 mmol) in DCM (3 mL) at room temperature. After stirring for 2 hr, the solution was concentrated under vacuum. Flash column chromatography with a gradient of 0-1% EtOAc/Hex was used to purify the desired compound **1b** (172 mg, 94%). R_f = 0.62 (EtOAc/Hex, 1:9). ^1H NMR ($CDCl_3$, 400 MHz): δ 8.40 (s, 1H), 7.57 (td, *J* = 7.6, 1.4 Hz, 2H), 7.44 – 7.23 (m, 7H), 5.39 (q, *J* = 6.6 Hz, 1H), 3.72 (dd, *J* = 10.1, 2.7 Hz, 2H), 2.22 (t, *J* = 2.7 Hz, 1H), 1.66 (d, *J* = 6.6 Hz, 3H). ^{13}C NMR ($CDCl_3$, 101 MHz): δ 147.85, 143.00, 134.74, 130.20, 129.78, 129.34, 128.62, 128.49, 127.73, 127.22, 126.67, 81.61, 71.48, 23.54, 21.83. HRMS for $C_{18}H_{17}NNaO$ $[M+Na]^+$ calcd. 286.1208, found 286.1208.

Preparation of **1c**

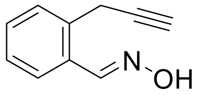

Pyridine (0.07 mL, 0.83 mmol) was added to a solution of **S5** (40 mg, 0.28 mmol) and hydroxylamine hydrochloride (29 mg, 0.42 mmol) in EtOH (3 mL) at room temperature. After stirring for 18 hr, the solution was concentrated under vacuum. Flash column chromatography with a gradient of 10-20% EtOAc/Hex was used to purify the desired compound **1c** (40 mg, 91%). ^1H NMR (400 MHz, Chloroform-*d*) δ 8.43 (s, 1H), 8.16 (s, 1H), 7.63 (dd, *J* = 7.7, 1.7 Hz, 1H), 7.54 (d, *J* = 6.5 Hz, 1H), 7.37 (td, *J* = 7.6, 1.6 Hz, 1H), 7.29 (t, *J* = 8.3 Hz, 1H), 3.74 (d, *J* = 2.8 Hz, 2H), 2.23 (t, *J* = 2.8 Hz, 1H). ^{13}C NMR (101 MHz, $CDCl_3$) δ 149.25, 134.87, 130.17, 129.98, 129.51, 128.06, 127.45, 81.43, 71.58, 23.37. HRMS for $C_{10}H_9NNaO$ $[M+Na]^+$ calcd. 182.0582, found 182.0591.

Preparation of **1d**

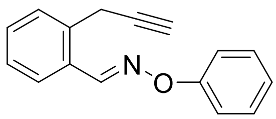

Pyridine (0.08 mL, 1.04 mmol) was added to a solution of **S5** (50 mg, 0.35 mmol) and **S12** (45 mg, 0.42 mmol) in DCM (3 mL) at room temperature. After stirring for 18 hr, the solution was concentrated under vacuum. Flash column chromatography with a gradient of 1-2% EtOAc/Hex was used to purify the desired compound **1d** (59 mg, 72%). ^1H NMR (400 MHz, Chloroform-*d*) δ 8.71 (s, 1H), 7.76 (dd, *J* = 7.7, 1.7 Hz, 1H), 7.65 (dd, *J* = 7.7, 1.2 Hz, 1H), 7.45 (td, *J* = 7.5, 1.5 Hz, 1H), 7.41 – 7.34 (m, 3H), 7.33 –

7.27 (m, 2H), 7.08 (tt, J = 7.3, 1.3 Hz, 1H), 3.90 (d, J = 2.8 Hz, 2H), 2.29 (t, J = 2.8 Hz, 1H). ^{13}C NMR (101 MHz, $CDCl_3$) δ 159.45, 150.94, 135.41, 130.70, 129.69, 129.51, 129.41, 129.30, 127.47, 122.50, 114.54, 81.39, 71.80, 23.90. HRMS for $C_{16}H_{13}NNaO$ $[M+Na]^+$ calcd. 258.0895, found 258.0899.

Preparation of **6c**

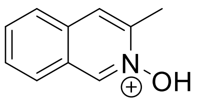

$NaAuCl_4 \cdot 2H_2O$ (5 mg, 13 μmol) was added to the solution of **1c** (20 mg, 125 μmol) in THF (1 mL)/H_2O (1 mL). After 2 hr stirring at room temperature, the reaction mixture was diluted with EtOAc and washed with H_2O. The organic phase was concentrated under vacuum. Flash column chromatography with a gradient of 10% MeOH/DCM was used to purify the desired compound **6c** (15 mg, 75%). ^{1}H NMR (400 MHz, Chloroform-*d*) δ 8.89 (s, 1H), 7.72 (dt, J = 7.4, 2.8 Hz, 2H), 7.66 (s, 1H), 7.58 – 7.52 (m, 2H), 2.66 (s, 3H). ^{13}C NMR (101 MHz, $CDCl_3$) δ 136.71, 129.09, 128.51, 126.01, 124.81, 123.37, 17.87. HRMS for $C_{10}H_{10}NO^+$ calcd. 160.0757, found 160.0762.

Preparation of **6d**

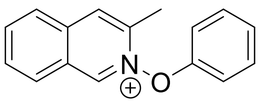

$NaAuCl_4 \cdot 2H_2O$ (3 mg, 9 μmol) was added to the solution of **1d** (20 mg, 125 μmol) in DCM (1 mL). After 1 hr stirring at room temperature, the solution was concentrated under vacuum. PTLC with 5% EtOAc/Hex was used to purify the desired compound **6d** (16 mg, 80%). ^{1}H NMR (400 MHz, Chloroform-*d*) δ 11.75 (s, 1H), 9.13 (s, 1H), 7.95 (dt, J = 8.3, 1.1 Hz, 1H), 7.79 (dd, J = 8.3, 1.1 Hz, 1H), 7.71 (ddd, J = 8.3, 6.9, 1.4 Hz, 1H), 7.65 (s, 1H), 7.58 (ddd, J = 8.1, 6.8, 1.3 Hz, 1H), 7.21 (dd, J = 7.4, 1.8 Hz, 1H), 7.15 (td, J = 7.6, 1.8 Hz, 1H), 7.00 (dd, J = 8.1, 1.4 Hz, 1H), 6.82 (td, J = 7.4, 1.4 Hz, 1H), 4.24 (s, 2H). ^{13}C NMR (101 MHz, $CDCl_3$) δ 156.63, 153.91, 151.17, 137.22, 131.46, 130.38, 128.69, 128.03, 127.32, 127.29, 127.02, 126.44, 120.19, 118.65, 118.40, 41.61. HRMS for $C_{16}H_{14}NNaO^+$ calcd. 236.1070, found 236.1084.

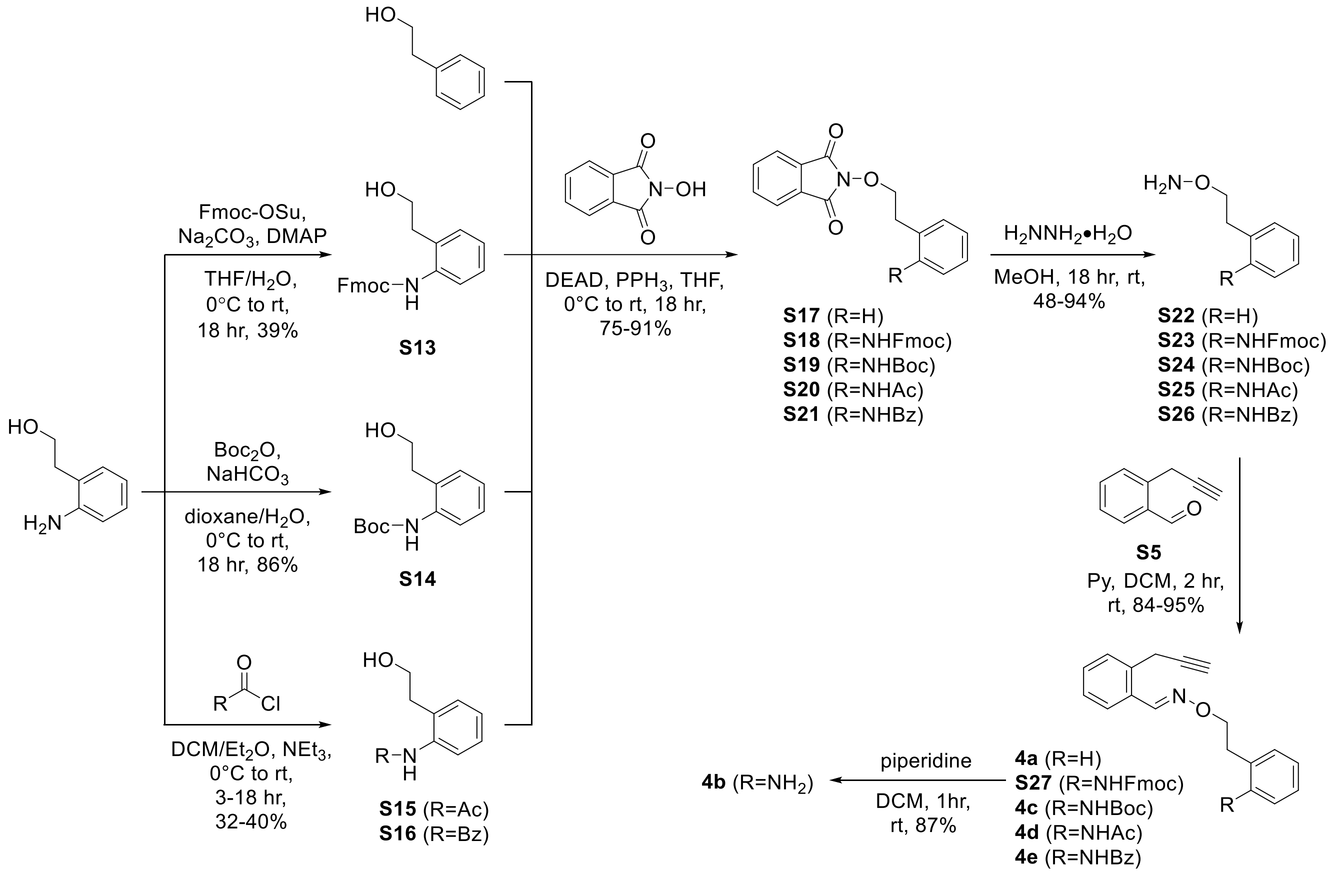

Scheme S2. Synthesis of compounds **4a-e**.

Preparation of **S13**

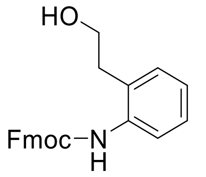

To a flask charged with 2-(2-Aminophenyl)ethanol (1.00 g, 7.29 mmol), Na_2CO_3 (1.93g, 18.22 mmol) and DMAP (89 mg, 0.73 mmol) in THF (6 mL) and H_2O (6 mL), a solution of Fmoc-OSu (3.69 g, 10.93 mmol) in THF (3 mL) was added at 0 °C. After overnight stirring at room temperature, the reaction mixture was diluted with EtOAc, then washed with H_2O and brine. The solution was then dried over Na_2SO_4 and concentrated under vacuum. The crude was recrystallized with EtOAc (7 mL) and the white solid was collected after filtration to afford compound **S13** (1.02 g, 39%). R_f = 0.26 (EtOAc/Hex, 3:7). ^{1}H NMR ($CDCl_3$, 400 MHz): δ 8.27 (s, 1H), 7.74 (s, 3H), 7.67 – 7.54 (m, 2H), 7.38 (m, 2H), 7.28 (m, 2H), 7.23 (m, 2H), 7.13 (m, 1H), 7.06 (m, 1H), 4.44 (d, J = 7.2 Hz, 2H), 4.25 (t, J = 7.4 Hz, 1H), 3.90 (t, J = 5.5 Hz, 2H), 2.83 (t, J = 5.5 Hz, 2H). ^{13}C NMR ($CDCl_3$, 101 MHz): δ 154.51, 144.02, 141.43, 136.85, 130.46, 127.82, 127.52, 127.18, 125.28, 120.09, 67.07, 64.59, 47.29, 34.88. HRMS for $C_{23}H_{21}NNaO_3$ $[M+Na]^+$ calcd. 382.1419, found 382.1425.

Preparation of **S14**

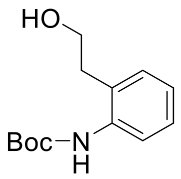

To a solution of 2-(2-Aminophenyl)ethanol (1.37 g, 9.99 mmol) in dioxane (10 mL), H_2O (5 mL) and saturated $NaHCO_3$ solution (5 mL) was added Di-tert-butyl decarbonate (2.40 g, 10.99 mmol) at 0 °C. After overnight stirring at room temperature, the reaction mixture was diluted with EtOAc, then washed with H_2O and brine. The solution was then dried over Na_2SO_4 and concentrated under vacuum to afford compound **S14** (2.03 g, 86%). R_f = 0.30 (EtOAc/Hex, 3:7). ^1H NMR ($CDCl_3$, 400 MHz): δ 7.72 (d, *J* = 8.1 Hz, 1H), 7.58 (s, 1H), 7.23 (td, *J* = 7.7, 1.7 Hz, 1H), 7.14 (dd, *J* = 7.6, 1.7 Hz, 1H), 7.05 (td, *J* = 7.4, 1.3 Hz, 1H), 3.92 (td, *J* = 5.7, 4.3 Hz, 2H), 2.84 (t, *J* = 5.7 Hz, 2H), 1.51 (s, 9H). ^{13}C NMR ($CDCl_3$, 101 MHz): δ 137.29, 130.86, 130.34, 127.43, 124.33, 123.14, 80.23, 64.41, 34.78, 28.54. HRMS for $C_{13}H_{19}NNaO_3$ $[M+Na]^+$ calcd. 260.1263, found 260.1264.

Preparation of **S15**

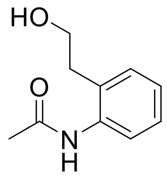

To a solution of 2-(2-Aminophenyl)ethanol (1.37 g, 10 mmol) in DCM (25 mL) and Et_2O (25 mL) was added NEt_3 (2.8 mL, 20 mmol) at 0 °C under argon. The reaction mixture was stirred for 15 min. Then acetic anhydride (1.10 mL, 12 mmol) at 0 °C and stirred at room temperature for 3 hr. To workup, the reaction mixture was quenched with iced water. Then the mixture was washed with 5% HCl, saturated $NaHCO_3$ and brine. The solution was then dried over Na_2SO_4 and concentrated under vacuum. Flash column chromatography with 30% EtOAc/Hex was used to purify the desired compound **S15** (0.72 g, 40%). R_f = 0.12 (EtOAc/Hex, 1:1). ^1H NMR ($CDCl_3$, 400 MHz): δ 9.34 (s, 1H), 7.65 (dd, *J* = 8.0, 1.3 Hz, 1H), 7.19 – 7.13 (m, 1H), 7.07 (dtd, *J* = 14.6, 7.6, 1.6 Hz, 2H), 4.04 – 3.95 (m, 1H), 3.78 (td, *J* = 5.4, 3.6 Hz, 2H), 2.78 – 2.69 (m, 2H), 2.03 (s, 3H). ^{13}C NMR ($CDCl_3$, 101 MHz): δ 169.50, 136.53, 132.68, 130.44, 127.06, 125.34, 124.13, 64.25, 34.98, 24.04. HRMS for $C_{10}H_{13}NNaO_2$ $[M+Na]^+$ calcd. 202.0844, found 202.0854.

Preparation of **S16**

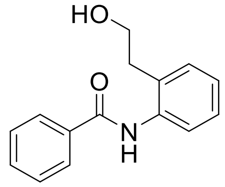

To a solution of 2-(2-Aminophenyl)ethanol (1.48 g, 10.8 mmol) in DCM (25mL) and Et_2O (25 mL) was added NEt_3 (2.2 mL, 16.2 mmol) at 0 °C under argon. The reaction mixture was stirred for 15 min. Then benzyl chloride (1.3 mL, 10.8 mmol) at 0 °C and stir at room temperature overnight. To workup, the reaction mixture was quenched with iced water. Then the mixture was washed with 5% HCl, saturated $NaHCO_3$ and brine. The solution was then dried over Na_2SO_4 and concentrated under vacuum to afford **S16** (0.83 g, 32%). R_f = 0.27 (EtOAc/Hex, 3:7). ^1H NMR ($CDCl_3$, 400 MHz): δ 9.75 (s, 1H), 8.03 – 7.91 (m, 3H), 7.56 – 7.42 (m, 3H), 7.30 (td, *J* = 7.7, 1.8 Hz, 1H), 7.22 – 7.09 (m, 2H), 4.07 – 3.94 (m, 2H), 2.90 (t, *J* = 5.4 Hz, 2H), 2.42 (t, *J* = 3.6 Hz, 1H). ^{13}C NMR ($CDCl_3$, 101 MHz): δ 165.82, 137.12, 135.03, 133.72, 132.41, 131.76, 130.56, 130.29, 128.75, 128.60, 127.50, 127.46, 125.41, 124.44, 65.31, 34.88. HRMS for $C_{15}H_{15}NNaO_2$ $[M+Na]^+$ calcd. 264.1000, found 264.1012.

Preparation of **S17**

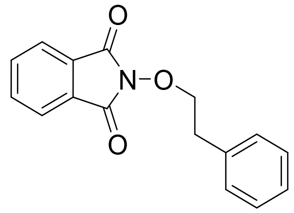

Diethyl azodicarboxylate (0.64 mL, 4.09 mmol) was added dropwise to a solution of phenethyl alcohol (500 mg, 4.09 mmol), N-Hydroxyphthalimide (668 mg, 4.09 mmol) and triphenylphosphine (1.18 g, 4.50 mmol) in anhydrous THF (5 mL) under argon at 0 °C. After stirring overnight at room temperature, the reaction was diluted with EtOAc. The solution was washed with H_2O and brine, then dried over Na_2SO_4, and concentrated under vacuum. Flash column chromatography with 10% EtOAc/Hex was used to purify the desired compound **S17** (0.87 g, 80%). R_f = 0.46 (EtOAc/Hex, 1:4). ^{1}H NMR ($CDCl_3$, 400 MHz): δ 7.84 – 7.78 (m, 2H), 7.76 – 7.70 (m, 2H), 7.29 (d, *J* = 4.4 Hz, 4H), 7.24 – 7.17 (m, 1H), 4.42 (t, *J* = 7.4 Hz, 2H), 3.14 (t, *J* = 7.4 Hz, 2H). ^{13}C NMR ($CDCl_3$, 101 MHz): δ 163.72, 136.87, 134.61, 129.03, 128.97, 128.68, 126.76, 123.64, 78.64, 34.74. HRMS for $C_{16}H_{13}NNaO_3$ $[M+Na]^+$ calcd. 290.0793, found 290.0812.

Preparation of **S18**

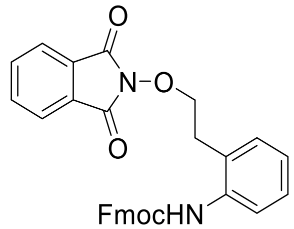

Diethyl azodicarboxylate (0.5 mL, 2.84 mmol) was added dropwise to a solution of **S13** (1.02 g, 0.84 mmol), N-Hydroxyphthalimide (463 mg, 2.84 mmol) and triphenylphosphine (819 mg, 3.12 mmol) in anhydrous THF (7 mL) under argon at 0 °C. After stirring overnight at room temperature, the reaction was diluted with EtOAc. The white solid was collected after filtration. The solid was washed with EtOAc to afford the desired compound **S18** (1.30 g, 91%). R_f = 0.35 (EtOAc/Hex, 3:7). ^{1}H NMR ($CDCl_3$, 400 MHz): δ 7.82 (dd, *J* = 5.5, 3.1 Hz, 2H), 7.78 – 7.70 (m, 4H), 7.70 – 7.58 (m, 3H), 7.41 – 7.33 (m, 2H), 7.27 (ddd, *J* = 8.6, 6.5, 3.2 Hz, 4H), 7.19 – 7.12 (m, 1H), 4.48 (dd, *J* = 13.0, 6.7 Hz, 4H), 4.27 (t, *J* = 7.0 Hz, 1H), 3.14 (t, *J* = 6.3 Hz, 2H). ^{13}C NMR ($CDCl_3$, 101 MHz): δ 163.61, 154.70, 144.07, 141.44, 136.18, 134.79, 130.17, 128.91, 127.89, 127.80, 127.21, 125.40, 123.81, 120.06, 79.59, 67.27, 47.32, 31.05. HRMS for $C_{31}H_{24}N_2NaO_5$ $[M+Na]^+$ calcd. 527.1583, found 527.1587.

Preparation of **S19**

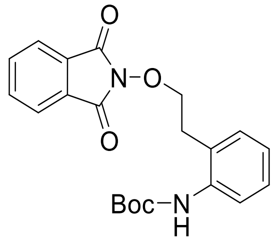

Diethyl azodicarboxylate (0.13 mL, 0.84 mmol) was added dropwise to a solution of **S14** (200 mg, 0.84 mmol), N-Hydroxyphthalimide (138 mg, 0.84 mmol) and triphenylphosphine (243 mg, 0.93 mmol) in anhydrous THF (5 mL) under argon at 0 °C. After stirring for 3 hr at room temperature, the reaction was diluted with EtOAc. The solution was washed with H_2O and brine, then dried over Na_2SO_4, and concentrated under vacuum. Flash column chromatography with 20% EtOAc/Hex was used to purify the desired compound **S19** (1.70 g, 86%). R_f = 0.53 (EtOAc/Hex, 3:7). ^{1}H NMR ($CDCl_3$, 400 MHz): δ 7.82 (dd, *J* = 5.5, 3.1 Hz, 2H), 7.74 (dd, *J* = 5.5, 3.1 Hz, 2H), 7.27 – 7.19 (m, 2H), 7.08 (t, 1H), 6.81 (s, 1H), 4.44 (t, *J* = 6.8 Hz, 2H), 3.11 (t, *J* = 6.8 Hz, 2H), 1.50 (s, 9H). ^{13}C NMR ($CDCl_3$, 101 MHz): δ 163.50,

153.79, 136.48, 134.68, 129.91, 128.90, 127.69, 124.88, 123.69, 80.39, 78.73, 77.48, 77.36, 77.16, 77.00, 76.84, 30.66, 28.42. HRMS for $C_{21}H_{22}N_2NaO_5$ $[M+Na]^+$ calcd. 405.1426, found 405.1423.

Preparation of **S20**

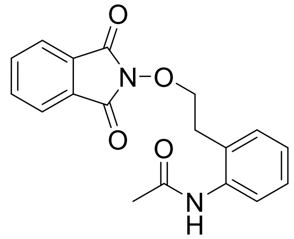

Diethyl azodicarboxylate (0.15 mL, 0.97 mmol) was added dropwise to a solution of **S15** (173.84 mg, 0.97 mmol), N-Hydroxyphthalimide (158 mg, 0.97 mmol) and triphenylphosphine (279 mg, 1.07 mmol) in anhydrous THF (5 mL) under argon at 0 °C. After stirring overnight at room temperature, the reaction was diluted with EtOAc. The solution was washed with H_2O and brine, then dried over Na_2SO_4, and concentrated under vacuum. Flash column chromatography with 30% EtOAc/Hex was used to purify the desired compound **S20** (272 mg, 87%). R_f = 0.38 (EtOAc/Hex, 7:3). ^{1}H NMR ($CDCl_3$, 400 MHz): δ 8.16 (s, 1H), 7.84 (dd, *J* = 5.2, 2.0 Hz, 2H), 7.77 (dd, *J* = 5.5, 3.1 Hz, 2H), 7.75 – 7.70 (d, 1H), 7.30 – 7.21 (m, 3H), 7.15 (td, *J* = 7.5, 1.3 Hz, 1H), 4.50 (t, *J* = 6.1 Hz, 2H), 3.12 (t, *J* = 6.1 Hz, 2H), 2.27 (s, 3H). ^{13}C NMR ($CDCl_3$, 101 MHz): δ 169.33, 163.56, 136.19, 134.80, 130.23, 130.05, 128.70, 127.69, 125.86, 125.57, 123.77, 80.32, 77.35, 77.04, 76.72, 31.25, 24.11. HRMS for $C_{18}H_{16}N_2NaO_4$ $[M+Na]^+$ calcd. 347.1008, found 347.1015.

Preparation of **S21**

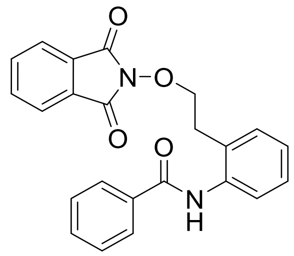

Diethyl azodicarboxylate (0.26 mL, 1.66 mmol) was added dropwise to a solution of **S16** (400 mg, 1.66 mmol), N-Hydroxyphthalimide (271 mg, 1.66mmol) and triphenylphosphine (480 mg, 1.83 mmol) in anhydrous THF (10 mL) under argon at 0 °C. After stirring overnight at room temperature, the reaction was concentrated under vacuum to afford the crude. Then the solid was with EtOAc and H_2O to afford **S21** (479 mg, 75%). R_f = 0.69 (EtOAc/Hex, 1:1). ^{1}H NMR ($CDCl_3$, 400 MHz): δ 9.75 (s, 1H), 8.02 – 7.93 (m, 3H), 7.56 – 7.41 (m, 3H), 7.30 (td, *J* = 7.7, 1.8 Hz, 1H), 7.22 – 7.11 (m, 2H), 4.02 (dd, *J* = 6.0, 4.8 Hz, 2H), 2.94 – 2.85 (m, 2H). ^{13}C NMR ($CDCl_3$, 101 MHz): δ 165.82, 137.12, 135.03, 133.72, 132.41, 131.76, 130.56, 130.29, 128.75, 128.60, 127.50, 127.46, 125.41, 124.44, 65.31, 34.88. HRMS for $C_{23}H_{18}NNaO_4$ $[M+Na]^+$ calcd. 409.1164, found 409.1168.

Preparation of **S22**

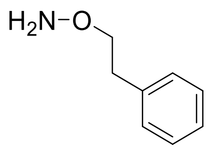

Hydrazine hydrate (0.95 mL, 9.76 mmol, 50%) was added to a solution of **S17** (870 mg, 3.25 mmol) in MeOH (5 mL) at room temperature. After overnight stirring, the reaction mixture was diluted with EtOAc (20 mL). The solution was washed with H_2O and brine, then dried over Na_2SO_4, and concentrated under vacuum. Flash column chromatography with 20% EtOAc/Hex was used to purify the desired compound **S22** (420 mg, 94%). R_f = 0.20 (EtOAc/Hex, 1:4). ^{1}H NMR ($CDCl_3$, 400 MHz): δ 7.31 – 7.19 (m, 6H), 5.35 (s, 2H), 3.87 (t, *J* = 6.9 Hz, 2H), 2.89 (t, *J* = 7.0 Hz, 2H). ^{13}C NMR ($CDCl_3$, 101 MHz): δ 138.85,

129.11, 128.91, 128.58, 128.46, 126.45, 126.27, 76.49, 35.07. HRMS for $C_8H_{11}NNaO$ $[M+Na]^+$ calcd. 160.0738, found 160.0742.

Preparation of **S23**

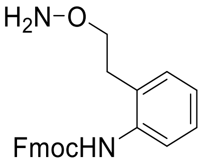

Hydrazine hydrate (0.88 mL, 9.04 mmol, 50%) was added to a solution of **S18** (1.52 g, 3.01 mmol) in MeOH (15 mL) at room temperature. After overnight stirring, the reaction mixture was diluted with EtOAc (30 mL). The solution was washed with H_2O and brine, then dried over Na_2SO_4, and concentrated under vacuum. Flash column chromatography with 20% EtOAc/Hex was used to purify the desired compound **S23** (910 mg, 81%). $R_f = 0.68$ (EtOAc/Hex, 1:1). ^1H NMR ($CDCl_3$, 400 MHz): δ 8.12 (s, 1H), 7.76 (d, J = 7.5 Hz, 2H), 7.60 (d, J = 7.3 Hz, 2H), 7.44 – 7.35 (m, 2H), 7.30 (t, J = 7.4 Hz, 2H), 7.26 – 7.17 (m, 1H), 7.09 (dd, J = 7.6, 1.7 Hz, 1H), 7.06 – 6.97 (m, 1H), 5.26 (s, 2H), 4.55 (d, J = 6.4 Hz, 2H), 4.26 (t, J = 6.4 Hz, 1H), 3.83 (t, J = 5.9 Hz, 2H), 2.82 (t, J = 5.9 Hz, 2H). ^{13}C NMR ($CDCl_3$, 101 MHz): δ 154.16, 144.04, 141.46, 136.63, 130.35, 127.82, 127.56, 127.19, 125.05, 120.04, 66.43, 47.35, 31.44. HRMS for $C_{23}H_{22}N_2NaO_3$ $[M+Na]^+$ calcd. 397.1528, found 397.1532.

Preparation of **S24**

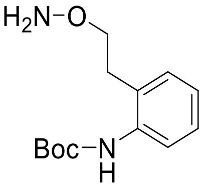

Hydrazine hydrate (0.22 mL, 2.30 mmol, 50%) was added to a solution of **S19** (293 mg, 0.77 mmol) in MeOH (5 mL) at room temperature. After overnight stirring, the white solid was filtered off. The filtrate was then concentrated under vacuum, and then redissolved in EtOAc (20 mL). The solution was washed with H_2O and brine, then dried over Na_2SO_4, and concentrated under vacuum to afford compound **S24** (176 mg, 91%). $R_f = 0.44$ (EtOAc/Hex, 3:7). ^1H NMR ($CDCl_3$, 400 MHz): δ 7.77 (d, J = 8.1 Hz, 1H), 7.49 (s, 1H), 7.21 (td, J = 7.7, 1.7 Hz, 1H), 7.12 (dd, J = 7.6, 1.7 Hz, 1H), 7.01 (td, J = 7.4, 1.3 Hz, 1H), 5.47 (s, 2H), 3.88 (t, J = 6.0 Hz, 2H), 2.85 (t, J = 6.0 Hz, 2H), 1.51 (s, 9H). ^{13}C NMR ($CDCl_3$, 101 MHz): δ 153.68, 137.03, 130.21, 129.95, 127.37, 123.92, 122.35, 80.14, 76.76, 31.34, 28.51. HRMS for $C_{13}H_{21}N_2O_3$ $[M+H]^+$ calcd. 253.1552, found 253.1553.

Preparation of **S25**

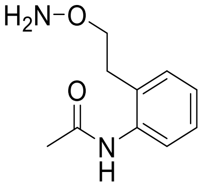

Hydrazine hydrate (0.19 mL, 3.00 mmol, 50%) was added to a solution of **S20** (324 mg, 1.00 mmol) in MeOH (4 mL) at room temperature. After overnight stirring, reaction mixture was concentrated under vacuum, and then redissolved in EtOAc. The solution was washed with H_2O and brine, then dried over Na_2SO_4, and concentrated under vacuum. Flash column chromatography with 50% EtOAc/Hex was used to purify the desired compound **S25** (94 mg, 48%). $R_f = 0.16$ (EtOAc/Hex, 7:3). ^1H NMR ($CDCl_3$, 400 MHz): δ 8.54 (s, 1H), 7.78 (dd, J = 8.1, 1.3 Hz, 1H), 7.20 (td, J = 7.7, 1.8 Hz, 1H), 7.13 (dd, J = 7.7, 1.7 Hz, 1H), 7.05 (td, J = 7.4, 1.3 Hz, 1H), 3.85 (t, J = 6.0 Hz, 2H), 2.82 (t, J = 6.0 Hz, 2H), 2.12 (s, 3H). ^{13}C NMR ($CDCl_3$, 101 MHz): δ 168.85, 136.31, 130.88, 130.18, 127.24, 125.07, 123.91, 31.17, 24.24. HRMS for $C_{10}H_{14}NNaO_2$ $[M+Na]^+$ calcd. 217.0953, found 217.0965.

Preparation of **S26**

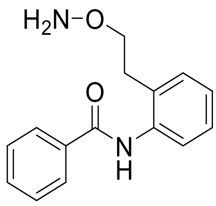

Hydrazine hydrate (0.15 mL, 2.33 mmol, 50%) was added to a solution of **S21** (300 mg, 0.78 mmol) in MeOH (4 mL) at room temperature. After overnight stirring, the reaction mixture was concentrated under vacuum to afford the solid. The crude was washed with EtOAc and H_2O to afford compound **S26** (120 mg, 60%). R_f = 0.47 (EtOAc/Hex, 7:3). ^{1}H NMR ($CDCl_3$, 400 MHz): δ 9.22 (s, 1H), 8.01 (d, *J* = 8.1 Hz, 1H), 7.98 – 7.90 (m, 2H), 7.57 – 7.44 (m, 3H), 7.30 (td, *J* = 7.7, 1.7 Hz, 1H), 7.20 (dd, *J* = 7.6, 1.7 Hz, 1H), 7.13 (td, *J* = 7.4, 1.3 Hz, 1H), 5.47 (s, 2H), 3.98 (dd, *J* = 6.1, 5.1 Hz, 2H), 2.90 (t, *J* = 5.6 Hz, 2H). ^{13}C NMR ($CDCl_3$, 101 MHz): δ 165.70, 136.59, 135.10, 131.70, 131.56, 130.42, 128.68, 127.40, 127.25, 125.23, 124.07, 77.89, 31.73. HRMS for $C_{15}H_{16}NNaO_2$ $[M+Na]^+$ calcd. 279.1109, found 279.1119.

Preparation of **S27**

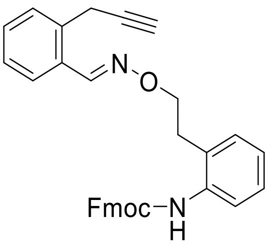

Pyridine (0.22 mL, 2.77 mmol) was added to a solution of **S23** (210 mg, 0.83 mmol) and **S5** (100 mg, 0.69 mmol) in DCM (4 mL) at room temperature. After stirring for 2 hr, the solution was concentrated under vacuum. Flash column chromatography with 5% EtOAc/Hex was used to purify the desired compound **S27** (220 mg, 84%). R_f = 0.58 (EtOAc/Hex, 3:7). ^{1}H NMR ($CDCl_3$, 400 MHz): δ 8.38 (s, 1H), 7.75 (d, *J* = 7.5 Hz, 2H), 7.63 – 7.53 (m, 3H), 7.49 (d, *J* = 7.7 Hz, 1H), 7.37 (dd, *J* = 8.0, 6.9 Hz, 2H), 7.33 – 7.22 (m, 8H), 7.15 (dt, *J* = 22.8, 7.5 Hz, 2H), 4.50 – 4.37 (m, 4H), 4.20 (t, *J* = 7.3 Hz, 1H), 3.66 (s, 2H), 3.07 (t, *J* = 6.1 Hz, 2H), 2.16 (t, *J* = 2.7 Hz, 1H). ^{13}C NMR ($CDCl_3$, 101 MHz): δ 143.98, 141.44, 135.01, 130.53, 130.27, 129.51, 128.60, 127.86, 127.73, 127.35, 127.22, 125.22, 120.15, 75.25, 71.62, 67.26, 47.28, 29.85, 23.51. HRMS for $C_{33}H_{28}N_2NaO_3$ $[M+Na]^+$ calcd. 523.1998, found 523.1999.

Preparation of **4a**

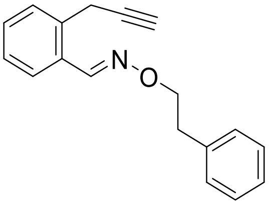

Pyridine (0.22 mL, 2.77 mmol) was added to a solution of **S22** (114 mg, 0.83 mmol) and **S5** (100 mg, 0.69 mmol) in DCM (4 mL) at room temperature. After stirring overnight, the solution was concentrated under vacuum. Flash column chromatography with 2% EtOAc/Hex was used to purify the desired compound **4a** (170 mg, 93%). R_f = 0.58 (EtOAc/Hex, 1:9). ^{1}H NMR ($CDCl_3$, 400 MHz): δ 8.38 (s, 1H), 7.66 (dd, *J* = 7.6, 1.5 Hz, 1H), 7.61 – 7.55 (m, 1H), 7.41 – 7.26 (m, 6H), 7.24 (d, *J* = 7.0 Hz, 1H), 4.43 (t, *J* = 7.1 Hz, 2H), 3.78 (d, *J* = 2.7 Hz, 2H), 3.09 (t, *J* = 7.1 Hz, 2H), 2.25 (t, *J* = 2.7 Hz, 1H). ^{13}C NMR ($CDCl_3$, 101 MHz): δ 147.78, 138.65, 134.77, 130.13, 129.90, 129.43, 129.15, 128.57, 128.53, 128.41, 128.00, 127.33, 126.43, 125.94, 81.56, 75.04, 71.54, 35.91, 23.53. HRMS for $C_{18}H_{17}NNaO$ $[M+Na]^+$ calcd. 286.1208, found 286.1212.

Preparation of **4b**

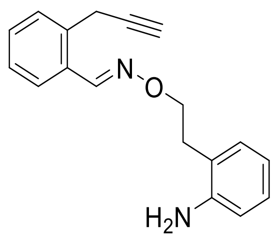

Piperidine (0.34 mL, 3.40 mmol) was added to a solution of **S27** (340 mg, 0.68 mmol) in DCM (3 mL) at room temperature. After stirring for 1 hr, the solution was concentrated under vacuum. Flash column chromatography with 10% EtOAc/Hex was used to purify the desired compound **4b** (164 mg, 87%). R_f = 0.50 (EtOAc/Hex, 3:7). ^{1}H NMR ($CDCl_3$, 400 MHz): δ 8.37 (s, 1H), 7.63 (dd, *J* = 7.6, 1.5 Hz, 1H), 7.54 (dd, *J* = 7.5, 1.3 Hz, 1H), 7.37 (td, *J* = 7.5, 1.5 Hz, 1H), 7.29 (td, *J* = 7.6, 1.4 Hz, 1H), 7.08 (ddd, *J* = 15.5, 7.7, 1.6 Hz, 2H), 6.75 (td, *J* = 7.4, 1.3 Hz, 1H), 6.70 (dd, *J* = 7.9, 1.2 Hz, 1H), 4.41 (t, *J* = 7.0 Hz, 2H), 3.96 – 3.77 (m, 2H), 3.76 (d, *J* = 2.7 Hz, 2H), 2.98 (t, *J* = 7.0 Hz, 2H), 2.22 (t, *J* = 2.7 Hz, 1H). ^{13}C NMR ($CDCl_3$, 101 MHz): δ 148.03, 144.95, 134.83, 130.53, 130.05, 130.01, 129.56, 128.44, 127.78, 127.41, 123.10, 118.96, 115.95, 81.58, 73.78, 71.51, 31.57, 23.58. HRMS for $C_{18}H_{18}N_2NaO$ $[M+Na]^+$ calcd. 301.1317, found 301.1324.

Preparation of **4c**

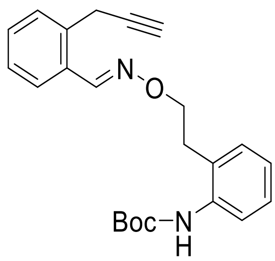

Pyridine (0.08 mL, 0.97 mmol) was added to a solution of **S24** (35 mg, 0.24 mmol) and **S5** (74 mg, 0.29 mmol) in DCM (3 mL) at room temperature. After stirring for 2 hr, the solution was concentrated under vacuum. Flash column chromatography with 5% EtOAc/Hex was used to purify the desired compound **4c** (87 mg, 95%). R_f = 0.38 (EtOAc/Hex, 1:9). ^{1}H NMR ($CDCl_3$, 400 MHz): δ 8.39 (s, 1H), 7.82 – 7.80 (d, *J* = 8.1 Hz, 1H), 7.64 – 7.62 (dd, *J* = 7.7, 1.4 Hz, 1H), 7.58 – 7.56 (dd, *J* = 7.7, 1.2 Hz, 1H), 7.38 (td, *J* = 7.6, 1.5 Hz, 1H), 7.29 (t, *J* = 7.5, 1.3 Hz, 1H), 7.22 (ddd, *J* = 12.5, 7.7, 1.6 Hz, 2H), 7.06 (td, *J* = 7.4, 1.3 Hz, 1H), 7.04 – 6.99 (m, 1H), 4.42 (t, *J* = 6.2 Hz, 2H), 3.77 (d, *J* = 2.7 Hz, 2H), 3.04 (t, *J* = 6.2 Hz, 2H), 2.23 (t, *J* = 2.7 Hz, 1H), 1.46 (s, 9H). ^{13}C NMR ($CDCl_3$, 101 MHz): δ 153.51, 148.84, 136.82, 134.94, 130.34, 130.18, 129.70, 129.51, 129.39, 128.64, 127.51, 127.36, 124.12, 81.40, 80.26, 77.36, 75.13, 71.66, 31.78, 28.43, 23.61. HRMS for $C_{23}H_{26}N_2NaO_3$ $[M+Na]^+$calcd. 401.1841, found 401.1845.

Preparation of **4d**

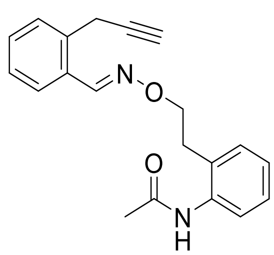

Pyridine (0.11 mL, 1.39 mmol) was added to a solution of **S25** (74 mg, 0.38 mmol) and **S5** (50 mg, 0.35 mmol) in DCM (4 mL) at room temperature. After stirring for 2 hr, the solution was concentrated under vacuum. Flash column chromatography with 30% EtOAc/Hex was used to purify the desired compound **4d** (103 mg, 93%). R_f = 0.12 (EtOAc/Hex, 3:7). ^{1}H NMR ($CDCl_3$, 400 MHz): δ 8.41 (s, 1H), 7.82 (dd, *J* = 8.0, 1.3 Hz, 1H), 7.81 – 7.76 (m, 1H), 7.65 (dd, *J* = 7.7, 1.5 Hz, 1H), 7.51 (dd, *J* = 7.8, 1.3 Hz, 1H), 7.38 (td, *J* = 7.6, 1.5 Hz, 1H), 7.33 – 7.21 (m, 3H), 7.13 (td, *J* = 7.5, 1.3 Hz, 1H), 4.43 (t, *J* = 6.1 Hz, 2H), 3.72 (d, *J* = 2.7 Hz, 2H), 3.04 (t, *J* = 6.1 Hz, 2H), 2.22 (t, *J* = 2.7 Hz, 1H), 2.09 (s, 3H). ^{13}C NMR ($CDCl_3$,

101 MHz): δ 168.60, 148.77, 136.21, 135.01, 130.62, 130.49, 130.41, 129.78, 129.58, 128.30, 127.57, 127.53, 125.50, 124.21, 81.40, 75.42, 71.61, 31.77, 24.45, 23.57. HRMS for $C_{20}H_{20}N_2NaO_2$ $[M+Na]^+$ calcd. 343.1422, found 343.1431.

Preparation of **4e**

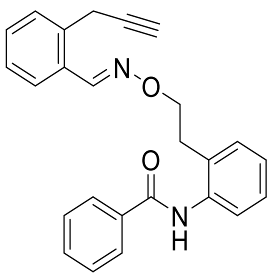

Pyridine (0.11 mL, 1.39 mmol) was added to a solution of **S26** (89 mg, 0.35 mmol) and **S5** (50 mg, 0.35 mmol) in DCM (3 mL) at room temperature. After stirring for 2 hr, the solution was concentrated under vacuum. Flash column chromatography with 10% EtOAc/Hex was used to purify the desired compound **4e** (121 mg, 91%). R_f = 0.31 (EtOAc/Hex, 1:4). ^{1}H NMR ($CDCl_3$, 400 MHz): δ 8.65 (s, 1H), 8.23 (s, 1H), 8.04 (d, J = 8.1 Hz, 1H), 7.99 – 7.87 (m, 2H), 7.49 (ddt, J = 10.9, 5.5, 1.4 Hz, 3H), 7.42 – 7.27 (m, 5H), 7.21 (dtd, J = 18.9, 7.5, 1.3 Hz, 2H), 4.56 – 4.46 (m, 2H), 3.54 (d, J = 2.6 Hz, 2H), 3.12 (t, J = 5.9 Hz, 2H), 2.14 (t, J = 2.7 Hz, 1H). ^{13}C NMR ($CDCl_3$, 101 MHz): δ 148.68, 136.41, 134.95, 131.84, 131.18, 130.58, 130.38, 129.52, 129.41, 128.85, 128.25, 127.70, 127.43, 127.32, 125.65, 124.23, 81.21, 76.01, 71.66, 32.07, 23.36. HRMS for $C_{25}H_{22}N_2NaO_2$ $[M+Na]^+$ calcd. 405.1579, found 405.1580.

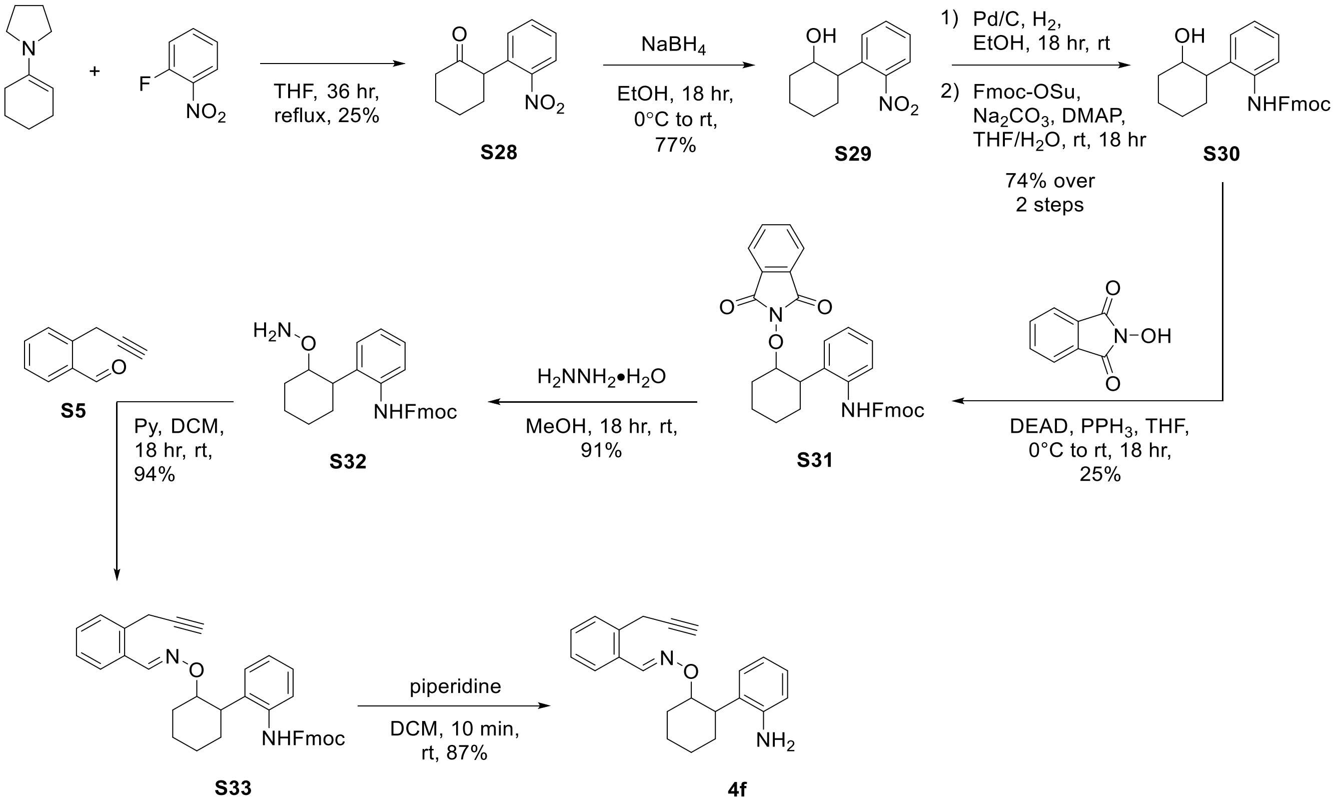

Scheme S3. Synthesis of compounds **4f**.

Preparation of **S28**

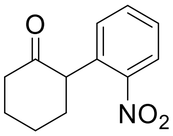

To a solution of 1-pyrrolidino-1-cyclohexane (604 mg, 4.0 mmol) in dry THF (4 mL) was added 1-fluor-2-nitrobenzene slowly (593 mg, 4.2 mmol) in dry THF (3 mL) at room temperature. The resulting mixture was refluxed under argon for 36 h. The mixture was cooled to room temperature and treated with 2.0 M HCl (10 mL) and refluxed for 2 h. The mixture was cooled to room temperature, diluted with EtOAc (20 mL) and H_2O (10 mL), and separated. The aqueous layer was extracted with EtOAc (15 mL × 3). The combined organic phase was washed with brine (15 mL), dried over Na_2SO_4, and then concentrated in vacuo. Flash column chromatography with 10% EtOAc/Hex was used to purify the desired compound **S28** (216 mg, 25%). R_f = 0.46 (EtOAc/Hex, 3:7). ^{1}H NMR ($CDCl_3$, 400 MHz): δ 7.99 (dd, *J* = 8.2, 1.4 Hz, 1H), 7.60 (td, *J* = 7.6, 1.4 Hz, 1H), 7.42 (ddd, *J* = 8.6, 7.5, 1.4 Hz, 1H), 7.35 (dd, *J* = 7.9, 1.4 Hz, 1H), 4.30 (dd, *J* = 12.9, 5.3 Hz, 1H), 2.62 – 2.47 (m, 2H), 2.44 – 2.33 (m, 1H), 2.26 – 2.15 (m, 1H), 2.15 – 2.01 (m, 2H), 1.94 – 1.74 (m, 2H). ^{13}C NMR ($CDCl_3$, 101 MHz): δ 208.26, 149.49, 134.15, 133.26, 130.41, 127.95, 125.08, 53.57, 42.32, 33.91, 27.41, 25.67. HRMS for $C_{12}H_{13}NNaO_3$ $[M+Na]^+$ calcd. 242.0793, found 242.0792.

Preparation of **S29**

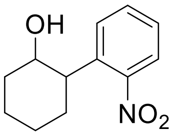

A solution of **S28** (1.10g, 5.0 mmol) in EtOH (3 mL) was added dropwise to the solution of $NaBH_4$ (0.83g, 21.9 mmol) in EtOH (8 mL) at 0 °C. After stirring overnight, the reaction mixture was quenched with saturated NH_4Cl and evaporate EtOH under vacuum. Extract with EtOAc (20 mL) and the organic layer was washed with brine. Dried over Na_2SO_4 and concentrated in vacuo. Flash column chromatography with 15-30% EtOAc/Hex was used to purify the desired compound **S29** (849 mg, 77%). R_f = 0.35 (EtOAc/Hex, 3:7). ^{1}H NMR ($CDCl_3$, 400 MHz): δ 7.72 (dd, *J* = 8.1, 1.4 Hz, 1H), 7.58 (td, *J* = 7.6, 1.4 Hz, 1H), 7.51 (dd, *J* = 8.0, 1.5 Hz, 1H), 7.35 (ddd, *J* = 8.5, 7.2, 1.6 Hz, 1H), 3.70 (tt, *J* = 10.1, 5.3 Hz, 1H), 3.02 (ddd, *J* = 11.9, 10.0, 3.5 Hz, 1H), 2.15 (ddd, *J* = 9.8, 5.1, 2.6 Hz, 1H), 2.02 – 1.92 (m, 1H), 1.87 (dt, *J* = 8.4, 2.4 Hz, 1H), 1.82 – 1.69 (m, 2H), 1.56 – 1.31 (m, 4H). ^{13}C NMR ($CDCl_3$, 101 MHz): δ 151.48, 138.02, 132.78, 128.08, 127.17, 124.13, 74.46, 46.75, 36.01, 33.45, 25.83, 25.13. HRMS for $C_{12}H_{15}NNaO_3$ $[M+Na]^+$ calcd. 244.0950, found 244.0950.

Preparation of **S30**

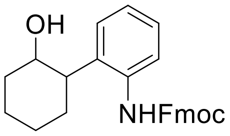

10% Pd/C (170 mg, 2% Pd) was dissolved in EtOH (5 mL) in a dry flask purged and charged with H_2. A solution of **S29** in EtOH (3 mL) was added dropwise to the flask and stirred at room temperature overnight. The palladium was filtered off with celite and the solution was concentrated in vacuo without purification to afford the amine-containing intermediate. R_f = 0.21 (EtOAc/Hex, 3:7). To a flask charged with the intermediate (383 mg, 2.0 mmol), Na_2CO_3 (193 mg, 1.8 mmol) and DMAP (24 mg, 0.2 mmol) in THF (3 mL) and H_2O (3 mL), a solution of Fmoc-Cl (569 mg, 2.2 mmol) in THF (3 mL) was added. After overnight stirring at room temperature, the reaction mixture was diluted with EtOAc, then washed with H_2O and brine. The solution was then dried over Na_2SO_4 and concentrated under vacuum. Flash column chromatography with 15% EtOAc/Hex was used to purify the desired compound **S30** (614 mg, 74%). R_f = 0.44 (EtOAc/Hex, 3:7). ^{1}H NMR ($CDCl_3$, 400 MHz): δ 7.79 (d, *J* = 7.6 Hz, 2H), 7.63 (s, 3H), 7.42 (t, *J* = 7.4 Hz, 2H), 7.36 – 7.27 (m, 3H), 7.25 – 7.16 (m, 2H), 4.50 – 4.44 (m, 2H), 4.32 (d, *J* = 8.0 Hz, 1H),

3.54 (ddt, J = 10.3, 7.5, 3.7 Hz, 1H), 2.77 (ddd, J = 12.9, 10.0, 3.1 Hz, 1H), 2.15 – 2.07 (m, 1H), 1.89 (dq, J = 12.1, 2.7 Hz, 1H), 1.82 (dq, J = 12.9, 2.4 Hz, 2H), 1.73 – 1.65 (m, 1H), 1.59 – 1.48 (m, 1H), 1.48 – 1.26 (m, 2H). ^{13}C NMR ($CDCl_3$, 101 MHz): δ 154.77, 144.05, 144.02, 141.46, 136.48, 127.83, 127.17, 126.99, 126.36, 125.74, 125.31, 120.13, 76.66, 67.16, 47.29, 45.46, 36.17, 31.39, 26.08, 25.16. HRMS for $C_{27}H_{27}NNaO_3$ $[M+Na]^+$ calcd. 436.1889, found 436.1889.

Preparation of **S31**

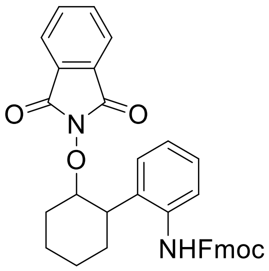

Diethyl azodicarboxylate (0.23 mL, 1.48 mmol) was added dropwise to a solution of **S30** (614 mg, 1.50 mmol), N-Hydroxyphthalimide (341 mg, 1.48 mmol) and triphenylphosphine (428 mg, 1.63 mmol) in anhydrous THF (5 mL) under argon at 0 °C. After stirring overnight at 35°C, the reaction was diluted with EtOAc. The solution was washed with H_2O and brine, then dried over Na_2SO_4, and concentrated under vacuum. Flash column chromatography with 20% EtOAc/Hex was used to purify the desired compound **S31** (210 mg, 25%). R_f = 0.33 (EtOAc/Hex, 3:7). ^{1}H NMR ($CDCl_3$, 400 MHz): δ 7.79 – 7.66 (m, 6H), 7.51 – 7.16 (m, 10H), 4.71 (d, J = 3.0 Hz, 1H), 4.48 (s, 2H), 4.26 (s, 1H), 3.13 (d, J = 12.4 Hz, 1H), 2.37 (qd, J = 12.7, 3.6 Hz, 1H), 2.22 (q, J = 14.1, 13.6 Hz, 1H), 2.15 – 2.07 (m, 1H), 2.04 – 1.95 (m, 1H), 1.75 (dd, J = 13.0, 3.7 Hz, 1H), 1.62 (t, J = 12.1 Hz, 2H), 1.47 (t, J = 13.0 Hz, 1H). ^{13}C NMR ($CDCl_3$, 101 MHz): δ 163.98, 144.02, 141.47, 135.00, 134.49, 129.83, 129.05, 127.83, 127.39, 127.17, 125.31, 125.23, 123.48, 120.11, 47.33, 29.64, 26.23, 25.58, 20.02. HRMS for $C_{35}H_{30}N_2NaO_5$ $[M+Na]^+$ calcd. 581.2052, found 581.2050.

Preparation of **S32**

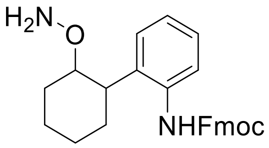

Hydrazine hydrate (0.17 mL, 1.77 mmol, 50%) was added to a solution of **S31** (330 mg, 0.59 mmol) in MeOH (3 mL) at room temperature. After overnight stirring, MeOH was removed under vacuum and the residue was dissolved in EtOAc (20 mL). The solution was washed with H_2O and brine, then dried over Na_2SO_4, and concentrated under vacuum. Flash column chromatography with 20% EtOAc/Hex was used to purify the desired compound **S32** (231 mg, 91%). R_f = 0.20 (EtOAc/Hex, 3:7). ^{1}H NMR ($CDCl_3$, 400 MHz): δ 8.31 (s, 1H), 7.79 (d, J = 7.6 Hz, 2H), 7.64 (s, 3H), 7.42 (t, J = 7.4 Hz, 2H), 7.32 (t, J = 7.5 Hz, 2H), 7.25 – 7.18 (m, 2H), 7.08 (t, J = 7.4 Hz, 1H), 5.22 (s, 2H), 4.52 (ddd, J = 33.7, 10.5, 6.8 Hz, 2H), 4.31 (d, J = 7.0 Hz, 1H), 3.91 (s, 1H), 2.82 (d, J = 13.0 Hz, 1H), 2.42 – 2.28 (m, 1H), 2.14 (qd, J = 12.9, 3.7 Hz, 1H), 1.88 (dt, J = 13.0, 4.1 Hz, 1H), 1.74 – 1.34 (m, 5H). ^{13}C NMR ($CDCl_3$, 101 MHz): δ 144.11, 144.03, 141.52, 141.51, 130.89, 127.82, 127.37, 127.14, 125.20, 125.17, 120.08, 83.50, 47.39, 29.02, 26.80, 25.54, 19.99. HRMS for $C_{27}H_{28}N_2NaO_3$ $[M+Na]^+$ calcd. 451.1998, found 451.1999.

Preparation of **S33**

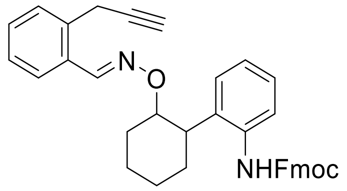

Pyridine (0.11 mL, 1.39 mmol) was added to a solution of **S32** (163 mg, 0.38 mmol) and **S5** (50 mg, 0.35 mmol) in DCM (2 mL) at room temperature. After stirring overnight, the solution was concentrated under vacuum. Flash column chromatography with 10% EtOAc/Hex was used to purify the desired compound **S33** (180 mg, 94%). R_f = 0.57 (EtOAc/Hex, 3:7). ^{1}H NMR ($CDCl_3$, 400 MHz): δ 8.28 (s, 1H), 7.85 – 7.03 (m, 16H), 4.63 (s, 2H), 4.48 (dd, *J* = 10.6, 6.7 Hz, 1H), 4.33 (s, 1H), 3.69 – 3.51 (m, 2H), 2.97 (d, *J* = 12.6 Hz, 1H), 2.40 – 2.31 (m, 1H), 2.24 (qd, *J* = 13.0, 3.7 Hz, 1H), 2.14 (t, *J* = 2.7 Hz, 1H), 2.01 – 1.88 (m, 1H), 1.71 – 1.55 (m, 4H), 1.49 (dddd, *J* = 16.3, 12.8, 8.2, 3.5 Hz, 1H). ^{13}C NMR ($CDCl_3$, 101 MHz): δ 148.23, 144.05, 143.93, 141.52, 134.75, 130.85, 129.90, 129.83, 129.32, 128.70, 127.86, 127.41, 127.19, 127.18, 125.19, 125.12, 120.13, 81.50, 71.50, 47.45, 31.05, 26.76, 25.81, 23.51, 19.91. HRMS for $C_{37}H_{34}N_2NaO_3$ $[M+Na]^+$ calcd. 577.2467, found 577.2468.

Preparation of **4f**

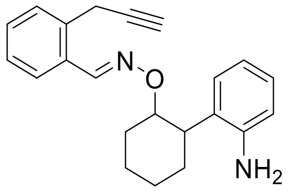

Piperidine (0.15 mL, 1.47 mmol) was added to a solution of **S33** (163 mg, 0.29 mmol) in DCM (2 mL) at room temperature. After stirring for 10 min, the solution was concentrated under vacuum. Flash column chromatography with 20% EtOAc/Hex was used to purify the desired compound **4f** (85 mg, 87%). R_f = 0.56 (EtOAc/Hex, 3:7). ^{1}H NMR ($CDCl_3$, 400 MHz): δ 8.26 (s, 1H), 7.53 (dd, *J* = 7.7, 1.3 Hz, 1H), 7.44 (dd, *J* = 7.8, 1.5 Hz, 1H), 7.30 (td, *J* = 7.5, 1.5 Hz, 1H), 7.26 – 7.19 (m, 2H), 7.02 (td, *J* = 7.6, 1.5 Hz, 1H), 6.75 (td, *J* = 7.5, 1.3 Hz, 1H), 6.70 (dd, *J* = 7.8, 1.3 Hz, 1H), 4.66 (s, 1H), 3.74 – 3.55 (m, 4H), 2.84 (dt, *J* = 12.8, 3.2 Hz, 1H), 2.33 (d, *J* = 10.5 Hz, 1H), 2.26 (d, *J* = 3.6 Hz, 1H), 2.21 (t, *J* = 2.7 Hz, 1H), 1.95 (d, *J* = 12.9 Hz, 1H), 1.73 – 1.66 (m, 2H), 1.64 (s, 1H), 1.60 (ddt, *J* = 12.9, 5.4, 2.8 Hz, 2H), 1.49 (dt, *J* = 12.7, 3.6 Hz, 1H). ^{13}C NMR ($CDCl_3$, 101 MHz): δ 147.21, 143.77, 134.51, 130.31, 129.48, 129.43, 129.17, 129.07, 128.69, 128.65, 127.09, 127.00, 126.79, 125.59, 119.74, 118.97, 116.25, 81.76, 79.49, 71.44, 55.05, 42.17, 31.19, 26.77, 26.39, 25.91, 23.57, 20.20. HRMS for $C_{22}H_{25}N_2O$ $[M+H]^+$ calcd. 333.1967, found 333.1966.

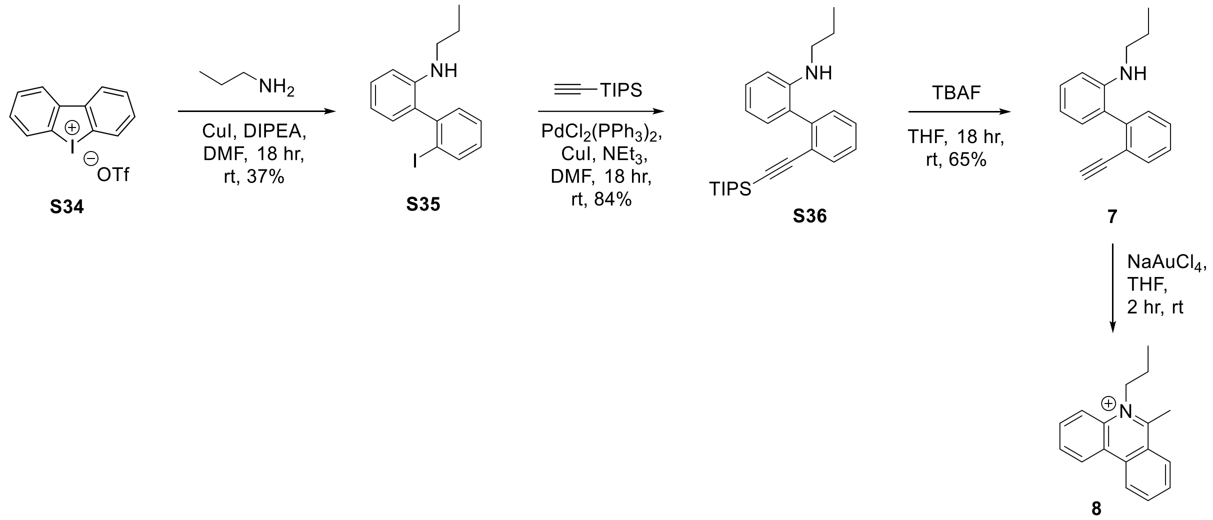

Scheme S4. Synthesis of model substrate **7**.

Preparation of **S34**

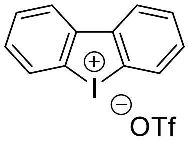

To a stirred solution of 2-iodobiphenyl (3.84 mL, 21.80 mmol) in DCM (60 mL) was added m-CPBA (4.51g, 25.16 mmol) and then TfOH (5.89 mL, 10.0 g, 66.70 mmol) was added to the mixture at 0 °C. The reaction mixture was slowly warm to room temperature and stirred for 1 hr. To workup, DCM was evaporated under vacuum. Et_2O (20 mL) was added and stir for 30 min. The white precipitate was filtered and washed by Et_2O. Yield: 7.10 g, 76%. ^{1}H NMR (400 MHz, Methanol-d_4): δ 8.40 (d, J = 9.3 Hz, 2H), 8.15 (d, J = 8.3 Hz, 2H), 7.88 (t, J = 8.1 Hz, 2H), 7.77 – 7.67 (m, 2H). ^{13}C NMR (101 MHz, MeOD): δ 142.35, 131.06, 130.95, 130.19, 126.99, 120.06. HRMS for $C_{12}H_8I$ $[M]^+$ calcd. 278.9665, found 278.9669.

Preparation of **S35**

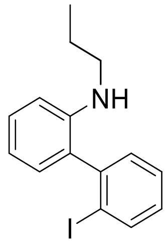

To a solution of **S34** (856.3 mg, 2.0 mmol) and copper iodide (38.1 mg, 0.2 mmol) dissolved in DMF (4mL), reagents propylamine (0.164 mL, 2.0 mmol) and DIPEA (1.05 mL, 6.0 mmol) were added dropwise. The reaction mixture was then stirred for 18 hr at room temp. To workup, the mixture was diluted with DCM, followed by washing with water, brine, and then dried over sodium sulfate. The solvent was then removed under vacuum, before the crude product was purified by column chromatography on silica gel (Ethyl Acetate/Hexane= 1/24) to give **S35** as a clear oil (250 mg, 37%). R_f = 0.56 (Ethyl Acetate/Hexane = 1/19). ^{1}H NMR (400 MHz, Chloroform-d) δ 7.99 (dd, J = 8.0, 1.2 Hz, 1H), 7.44 (td, J = 7.5, 1.2 Hz, 1H), 7.34 – 7.27 (m, 2H), 7.07 (td, J = 7.7, 1.8 Hz, 1H), 6.94 (dd, J = 7.4, 1.7 Hz, 1H), 6.80 – 6.70 (m, 2H), 3.30 (s, 1H), 3.09 (t, J = 7.1 Hz, 2H), 1.60 – 1.53 (m, 2H), 0.91 (t, J = 7.4 Hz, 3H). ^{13}C NMR ($CDCl_3$, 101 MHz): δ 145.19, 144.29, 139.68, 131.09, 130.18, 129.91, 129.40, 129.30, 128.90, 116.43, 110.61, 101.30, 45.87, 22.69, 11.77. HRMS for $C_{15}H_{17}IN$ $[M+H]^+$ calcd. 338.0405, found 338.0401.

Preparation of **S36**

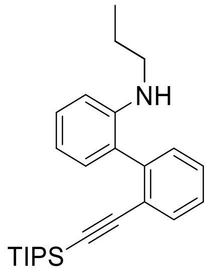

To a solution of **S35** (320 mg, 0.95 mmol), bis(triphenylphosphine)palladium chloride (67 mg, 0.095 mmol) and copper iodide (36.2 mg, 0.19 mmol) dissolved in DMF (4 mL) under argon, reagents (triisopropylsilyl)acetylene (0.43 mL, 1.90 mmol) and triethylamine (0.4 mL, 2.85 mmol) were added dropwise. The reaction mixture was then stirred for 18 hr at room temp. To workup, the mixture was first filtered to remove black impurities. Extraction using ethyl acetate was then performed, followed by washing with water, brine, and then dried over sodium sulfate. The solvent was then removed under vacuum, before the crude product was purified by column chromatography on silica gel (Ethyl Acetate/Hexane= 1/49) to give **S36** (313 mg, 84%). R_f = 0.76 (Ethyl Acetate/Hexane = 1/19). ^{1}H NMR (400 MHz, Chloroform-*d*) δ 7.64 – 7.59 (m, 1H), 7.41 – 7.35 (m, 1H), 7.31 (ddd, *J* = 7.2, 3.9, 2.3 Hz, 2H), 7.19 (ddd, *J* = 8.1, 7.4, 1.7 Hz, 1H), 7.05 (dd, *J* = 7.4, 1.7 Hz, 1H), 6.73 – 6.63 (m, 2H), 3.46 (s, 1H), 3.03 (td, *J* = 7.0, 4.5 Hz, 2H), 1.52 (h, *J* = 7.3 Hz, 2H), 0.94 (s, 22H), 0.88 (t, *J* = 7.4 Hz, 3H). ^{13}C NMR ($CDCl_3$, 101 MHz): δ 145.64, 142.39, 133.38, 130.53, 130.26, 128.86, 128.81, 127.35, 126.69, 123.99, 116.49, 110.52, 105.55, 45.98, 22.69, 18.63, 11.69, 11.30. HRMS for $C_{26}H_{38}NSi$ $[M+H]^+$ calcd. 392.2773, found 392.2769.

Preparation of **7**

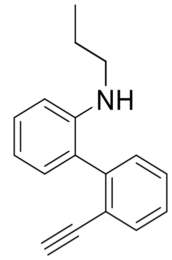

To a solution of **S36** (315 mg, 0.80 mmol) dissolved in THF (2 mL), TBAF (1.0 M in THF, 2 mL, 2.09 mmol) was added dropwise. The reaction mixture was then stirred for 18 hr at room temp. To workup, the mixture was quenched with saturated NH_4Cl solution. Extraction using ethyl acetate was then performed, followed by washing with water, brine, and then dried over sodium sulfate. The solvent was then removed under vacuum, before the crude product was purified by column chromatography on silica gel (Ethyl Acetate/Hexane= 1/49) to give **7** (127 mg, 65%). R_f = 0.75 (Ethyl Acetate/Hexane = 1/19). ^{1}H NMR (400 MHz, Chloroform-*d*) δ 7.62 (d, *J* = 7.6 Hz, 1H), 7.44 – 7.38 (m, 1H), 7.33 (d, *J* = 7.1 Hz, 2H), 7.29 – 7.22 (m, 1H), 7.07 (dd, *J* = 7.4, 1.6 Hz, 1H), 6.77 – 6.69 (m, 2H), 3.54 (s, 1H), 3.07 (q, *J* = 6.7 Hz, 2H), 2.96 (s, 1H), 1.54 (h, *J* = 7.3 Hz, 2H), 0.90 (t, *J* = 7.4 Hz, 3H). ^{13}C NMR ($CDCl_3$, 101 MHz): δ 145.74, 142.39, 133.72, 130.70, 130.42, 129.40, 129.15, 127.49, 126.16, 122.36, 116.45, 110.64, 82.40, 80.25, 46.04, 22.65, 11.74. HRMS for $C_{17}H_{18}N$ $[M+H]^+$ calcd. 236.1439, found 236.1438.

2.2.2 Catalyst preparation

Catalysts **Au1**, **Au3-Au7** were purchased from commercial sources. Catalysts **Au2**, **Au8-Au11** were prepared as outlined in Scheme S5.

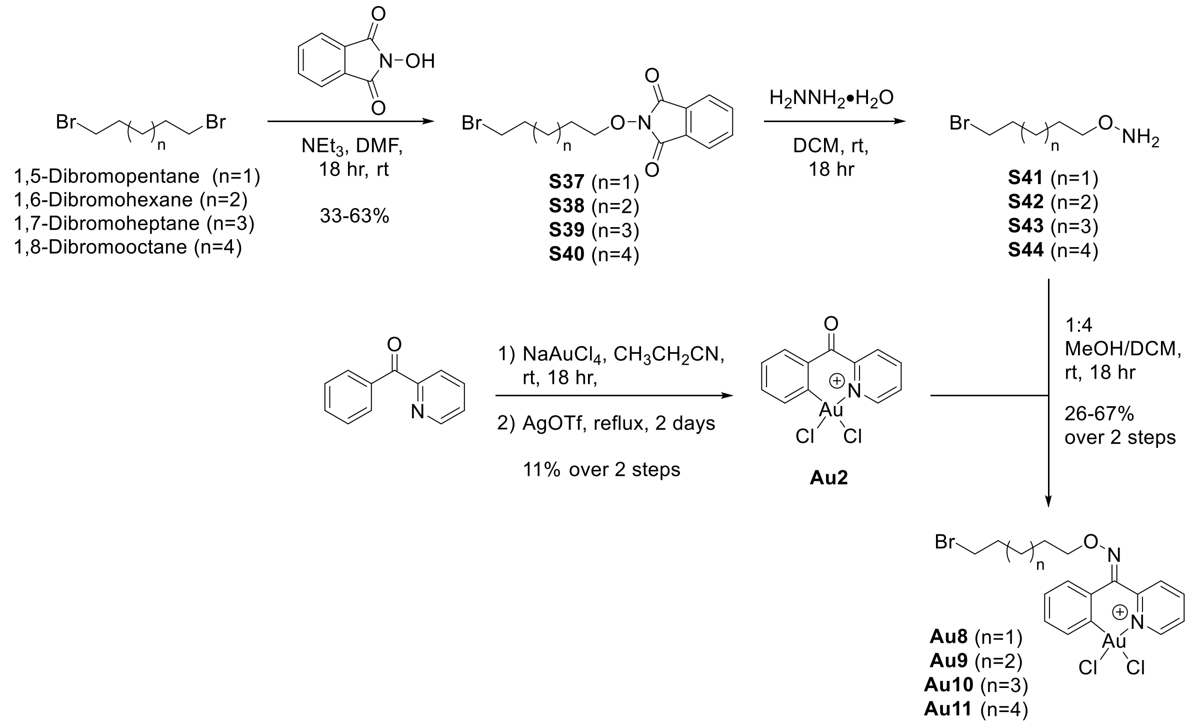

Scheme S5. Synthesis of gold catalysts **Au2, Au8-Au11**.

Preparation of **S37**

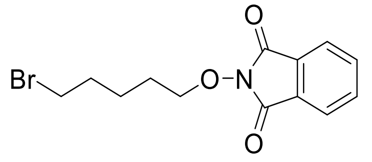

To a solution of N-hydroxy-phthalimide (1.0 g, 6.13 mmol) dissolved in DMF (4 mL), reagents 1,5-dibromopentane (1 mL, 7.34 mmol) and triethylamine (2.3 mL, 16.5 mmol) were added. The reaction mixture was then stirred for 18 hr at room temp. The red precipitate formed was then filtered off, followed by dilution of the filtrate with DCM (50 mL). After washing the organic solution with water (3×50 mL), the solvent was removed under vacuum. The crude product was purified by column chromatography on silica gel (Ethyl Acetate/Hexane= 1/9) to give **S37** as a white solid (495 mg, 50%). R_f = 0.72 (Ethyl Acetate/Hexane = 1/1). ^{1}H NMR (400 MHz, Chloroform-*d*) δ 7.84 – 7.77 (m, 2H), 7.75 – 7.67 (m, 2H), 4.18 (t, J = 6.4 Hz, 2H), 3.41 (t, J = 6.8 Hz, 2H), 1.98 – 1.85 (m, 2H), 1.84 – 1.72 (m, 2H), 1.71 – 1.56 (m, 2H). ^{13}C NMR ($CDCl_3$, 100 MHz): δ 163.68, 134.57, 128.91, 123.56, 78.14, 33.60, 32.39, 27.38, 24.36. HRMS for $C_{13}H_{14}BrNNaO_3$ $[M+Na]^+$ calcd. 334.0057, found 334.0055.

Preparation of **S38**

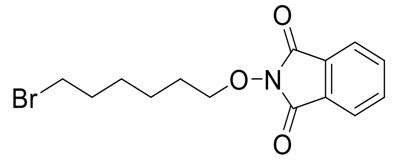

To a solution of N-hydroxy-phthalimide (163 mg, 1.00 mmol) dissolved in DMF (4 mL), reagents 1,6-dibromohexane (0.2 mL, 0.99 mmol) and triethylamine (0.4 mL, 2.87 mmol) was added. The reaction mixture was then stirred for 18 hr at room temp. The red precipitate formed was then filtered off, followed by dilution of the filtrate with DCM (50 mL). After washing the organic solution with water (3×50 mL),

the solvent was removed under vacuum. The crude product was purified by column chromatography on silica gel (Ethyl Acetate/Hexane= 1/9) to give **S38** as a white solid (150 mg, 46%). R_f = 0.60 (Ethyl Acetate/Hexane = 1/1). ^{1}H NMR (400 MHz, Chloroform-*d*) δ 7.83 – 7.77 (m, 2H), 7.73 (td, *J* = 5.2, 2.1 Hz, 2H), 4.18 (t, *J* = 6.5 Hz, 2H), 3.40 (t, *J* = 6.8 Hz, 2H), 1.93 – 1.82 (m, 2H), 1.81 – 1.72 (m, 2H), 1.57 – 1.43 (m, 4H). ^{13}C NMR ($CDCl_3$, 100 MHz): δ 134.60, 129.04, 123.63, 78.43, 33.92, 32.70, 28.10, 27.94, 24.91. HRMS for $C_{14}H_{16}BrNNaO_3$ $[M+Na]^+$ calcd. 348.0212, found 348.0209.

Preparation of **S39**

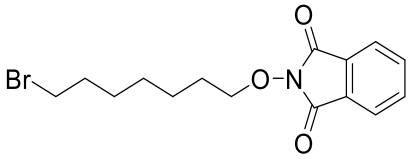

To a solution of N-hydroxy-phthalimide (1.0 g, 6.13 mmol) dissolved in DMF (4 mL), reagents 1,7-dibromoheptane (1.3 mL, 7.32 mmol) and triethylamine (2.3 mL, 16.5 mmol) were added. The reaction mixture was then stirred for 18 hr at room temp. The red precipitate formed was then filtered off, followed by dilution of the filtrate with DCM (50 mL). After washing the organic solution with water (3×50 mL), the solvent was removed under vacuum. The crude product was purified by column chromatography on silica gel (Ethyl Acetate/Hexane= 1/9) to give **S39** as a white solid (1.3 g, 63%). R_f = 0.79 (Ethyl Acetate/Hexane = 1/1). ^{1}H NMR (400 MHz, Chloroform-*d*) δ 7.82 – 7.76 (m, 2H), 7.74 – 7.69 (m, 2H), 4.16 (t, *J* = 6.6 Hz, 2H), 3.37 (t, *J* = 6.8 Hz, 2H), 1.83 (dd, *J* = 7.7, 6.6 Hz, 2H), 1.74 (dt, *J* = 8.4, 6.7 Hz, 2H), 1.52 – 1.30 (m, 6H). ^{13}C NMR ($CDCl_3$, 100 MHz): δ 163.67, 134.50, 128.91, 123.49, 78.42, 34.01, 32.64, 28.38, 28.03, 27.98, 25.38. HRMS for $C_{15}H_{18}BrNNaO_3$ $[M+Na]^+$ calcd. 362.0368, found 362.0370.

Preparation of **S40**

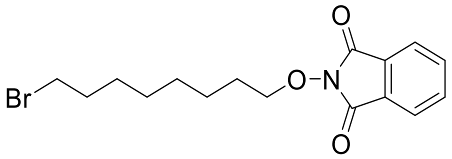

To a solution of N-hydroxy-phthalimide (1.0 g, 6.13 mmol) dissolved in DMF (4 mL), reagents 1,8-dibromooctane (1.4 mL, 7.33 mmol) and triethylamine (2.3 mL, 16.5 mmol) were added. The reaction mixture was then stirred for 18 hr at room temp. The red precipitate formed was then filtered off, followed by dilution of the filtrate with DCM (50 mL). After washing the organic solution with water (3×50 mL), the solvent was removed under vacuum. The crude product was purified by column chromatography on silica gel (Ethyl Acetate/Hexane= 1/9) to give **S40** as a white solid (720.4 mg, 33%). R_f = 0.72 (Ethyl Acetate/Hexane = 2/3). ^{1}H NMR (400 MHz, Chloroform-*d*) δ 7.77 – 7.71 (m, 2H), 7.71 – 7.66 (m, 2H), 4.11 (t, *J* = 6.7 Hz, 2H), 3.32 (t, *J* = 6.8 Hz, 2H), 1.84 – 1.61 (m, 4H), 1.47 – 1.19 (m, 8H). ^{13}C NMR ($CDCl_3$, 100 MHz): δ 163.48, 134.38, 128.79, 123.33, 78.34, 33.97, 32.64, 28.96, 28.48, 27.98, 27.94, 25.33. HRMS for $C_{16}H_{20}BrNNaO_3$ $[M+Na]^+$ calcd. 376.0525, found 376.0525.

Preparation of **Au2**

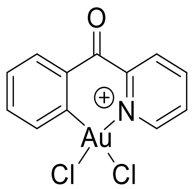

A mixture of sodium tetrachloroaurate dihydrate (413 mg, 1.04 mmol) and 2-benzoylpyridine (190 mg, 1.04 mmol) in propionitrile (15 mL) stirred at room temp for 18 hr before the addition of AgOTf (533 mg, 2.07 mmol). The reaction mixture was then stirred at reflux (100 °C) for 2 days. Afterwards, the solution was filtered while hot, and the filtrate volume was then reduced to ~2 mL. The concentrated solution was stirred at 0 °C for 15 min to afford a white precipitate, which was then filtered with cold acetone to obtain **Au2** (52.4 mg, 11%). ^{1}H NMR (DMSO, 400 MHz): δ 9.47 (dd, *J* = 5.9, 1.5 Hz, 1H),

8.55 (td, J = 7.7, 1.4 Hz, 1H), 8.36 (dd, J = 7.7, 1.6 Hz, 1H), 8.08 (ddd, J = 7.6, 5.9, 1.7 Hz, 1H), 7.79 – 7.72 (m, 1H), 7.71 – 7.64 (m, 1H), 7.53 – 7.41 (m, 2H).^{13}C NMR (DMSO, 100 MHz): δ 152.80, 144.34, 136.78, 133.98, 133.26, 129.95, 129.59, 128.61, 127.12. HRMS for $C_{12}H_8AuCl_2NNaO^+$ $[M+Na]^+$ calcd. 471.9541, found 471.9555.

Preparation of **Au8**

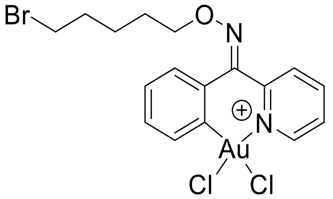

To a suspension of **S37** (40 mg, 0.128 mmol) in MeOH (3 ml), hydrazine (10 μL, 0.16 mmol) was added. The reaction mixture was then stirred for 18 hr at room temp. To workup, the mixture was diluted with EtOAc (10 mL), followed by washing with water (3×50 mL). The organic layer was then dried over sodium sulfate for at least 1 hr, before removing the solvent under vacuum to produce **S41** as a crude mixture. To a solution of crude **S41** and **Au2** (15 mg, 0.033 mmol) in 1:4 MeOH/DCM (5 mL) supplemented with a little Na_2SO_4 under argon, pyridine (0.01 mL, 0.124 mmol) was added. The reaction mixture was then stirred for 18 hr at room temp. To workup, the reaction mixture was filtered, and the filtrate volume was then reduced to ~2 ml. To the concentrated solution, diethyl ether (5 mL) was added and then stirred at room temp for 30 min to afford a precipitate. The residue was then filtered to obtain **Au8** (13.7 mg, 67%). 1H NMR (400 MHz, Chloroform-d) δ 9.48 (ddd, J = 6.0, 1.6, 0.7 Hz, 1H), 8.26 (ddd, J = 8.0, 1.7, 0.7 Hz, 1H), 8.16 (td, J = 7.8, 1.5 Hz, 1H), 7.71 (dd, J = 8.0, 1.2 Hz, 1H), 7.65 (ddd, J = 7.6, 6.0, 1.7 Hz, 1H), 7.41 (dd, J = 7.4, 1.8 Hz, 1H), 7.30 (dd, J = 7.3, 1.2 Hz, 1H), 7.25 – 7.20 (m, 1H), 4.38 (qt, J = 10.6, 6.6 Hz, 2H), 3.47 (t, J = 6.6 Hz, 2H), 1.94 (dq, J = 8.0, 6.6 Hz, 2H), 1.88 – 1.80 (m, 2H), 1.64 – 1.60 (m, 2H).^{13}C NMR ($CDCl_3$, 100 MHz): δ 152.12, 151.45, 145.58, 141.58, 138.40, 133.52, 130.08, 128.98, 128.75, 128.72, 127.99, 126.76, 76.31, 33.97, 32.35, 28.12, 24.67. HRMS for $C_{17}H_{18}AuBrCl_2N_2NaO^+$ $[M+Na]^+$ calcd. 634.9538, found 634.9552.

Preparation of **Au9**

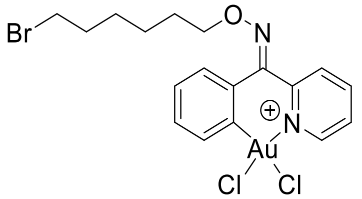

To a suspension of **S38** (50 mg, 0.153 mmol) in MeOH (3 mL), hydrazine (15 μL, 0.24 mmol) was added. The reaction mixture was then stirred for 18 hr at room temp. To workup, the mixture was diluted with EtOAc (10 mL), followed by washing with water (3×50 ml). The organic layer was then dried over sodium sulfate for at least 1 hr, before removing the solvent under vacuum to produce **S42** as a crude mixture. To a solution of crude **S42** and **Au2** (25 mg, 0.056 mmol) in 1:4 MeOH/DCM (5 mL) supplemented with a little Na_2SO_4 under argon, pyridine (0.01 mL, 0.124 mmol) was added. The reaction mixture was then stirred for 18 hr at room temp. To workup, the reaction mixture was filtered, and the filtrate volume was then reduced to ~2 ml. To the concentrated solution, diethyl ether (5 mL) was added and then stired at room temp for 30 min to afford a precipitate. The residue was then filtered to obtain **Au9** (10 mg, 28%). ^{1}H NMR (400 MHz, Chloroform-*d*) δ 9.44 (dddd, J = 34.8, 6.0, 1.5, 0.7 Hz, 1H), 8.25 (ddd, J = 8.1, 1.7, 0.7 Hz, 1H), 8.19 – 8.11 (m, 1H), 7.73 (ddd, J = 17.2, 8.0, 1.2 Hz, 1H), 7.64 (ddd, J = 7.6, 6.2, 1.7 Hz, 1H), 7.41 (dd, J = 7.4, 1.8 Hz, 1H), 7.31 – 7.27 (m, 1H), 7.23 (ddd, J = 8.0, 7.3, 1.9 Hz, 1H), 4.44 – 4.29 (m, 2H), 3.43 (t, J = 6.7 Hz, 2H), 1.95 – 1.77 (m, 4H), 1.56 – 1.40 (m, 4H). ^{13}C NMR ($CDCl_3$, 100 MHz): δ 152.12, 151.30, 141.51, 138.41, 133.52, 130.07, 128.95, 128.75, 128.72, 128.02, 126.73, 126.01, 34.02, 32.64, 28.85, 27.98, 25.30. HRMS for $C_{18}H_{20}AuBrCl_2N_2NaO^+$ $[M+Na]^+$ calcd.

648.9694, found 648.9700.

Preparation of **Au10**

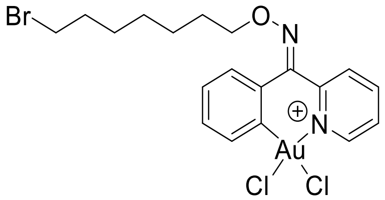

To a suspension of **S39** (40 mg, 0.118 mmol) in MeOH (3 mL), hydrazine (10 µL, 0.16 mmol) was added. The reaction mixture was then stirred for 18 hr at room temp. To workup, the mixture was diluted with EtOAc (10 mL), followed by washing with water (3×50 mL). The organic layer was then dried over sodium sulfate for at least 1 hr, before removing the solvent under vacuum to produce **S43** as a crude mixture. To a solution of crude **S43** and **Au2** (15 mg, 0.033 mmol) in 1:4 MeOH/DCM (5 mL) supplemented with a little Na_2SO_4 under argon, pyridine (0.01 mL, 0.124 mmol) was added. The reaction mixture was then stirred for 18 hr at room temp. To workup, the reaction mixture was filtered, and the filtrate volume was then reduced to ~2 ml. To the concentrated solution, diethyl ether (5 mL) was added and then stirred at room temp for 30 min to afford a precipitate. The residue was then filtered to obtain **Au10** (8.8 mg, 41%). ^{1}H NMR (400 MHz, Chloroform-*d*) δ 9.44 (ddd, *J* = 34.9, 6.1, 1.5 Hz, 1H), 8.25 (dd, *J* = 8.1, 1.6 Hz, 1H), 8.16 (td, *J* = 7.8, 1.5 Hz, 1H), 7.73 (ddd, *J* = 17.0, 8.0, 1.2 Hz, 1H), 7.64 (ddd, *J* = 7.7, 6.0, 1.7 Hz, 1H), 7.41 (dd, *J* = 7.4, 1.8 Hz, 1H), 7.29 (td, *J* = 7.4, 1.2 Hz, 1H), 7.25 – 7.20 (m, 1H), 4.42 – 4.26 (m, 2H), 3.42 (t, *J* = 6.8 Hz, 2H), 1.90 – 1.78 (m, 4H), 1.49 – 1.37 (m, 6H). ^{13}C NMR ($CDCl_3$, 100 MHz): δ 151.34, 150.46, 144.90, 140.73, 137.64, 132.75, 129.29, 128.18, 127.99, 127.96, 127.27, 125.96, 33.38, 32.03, 28.15, 27.85, 27.37, 25.13. HRMS for $C_{19}H_{22}AuBrCl_2N_2NaO^+$ $[M+Na]^+$ calcd. 662.9851, found 662.9846.

Preparation of **Au11**

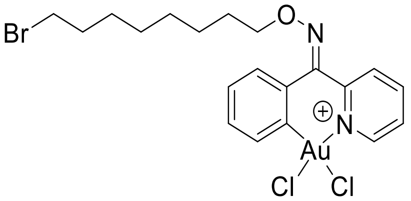

To a suspension of **S40** (40 mg, 0.113 mmol) in MeOH (3 mL), hydrazine (10 µL, 0.16 mmol) was added. The reaction mixture was then stirred for 18 hr at room temp. To workup, the mixture was diluted with EtOAc (10 mL), followed by washing with water (3×50 mL). The organic layer was then dried over sodium sulfate for at least 1 hr, before removing the solvent under vacuum to produce **S44** as a crude mixture. To a solution of crude **S44** and **Au2** (15 mg, 0.033 mmol) in 1:4 MeOH/DCM (5 mL) supplemented with a little Na_2SO_4 under argon, pyridine (0.01 mL, 0.124 mmol) was added. The reaction mixture was then stirred for 18 hr at room temp. To workup, the reaction mixture was filtered, and the filtrate volume was then reduced to ~2 ml. To the concentrated solution, diethyl ether (5 mL) was added and then stired at room temp for 30 min to afford a precipitate. The residue was then filtered to obtain **Au11** (5.6 mg, 26%). ^{1}H NMR (400 MHz, Chloroform-*d*) δ 9.54 – 9.34 (m, 1H), 8.31 – 8.21 (m, 1H), 8.15 (tdd, *J* = 7.6, 2.9, 1.5 Hz, 1H), 7.73 (ddd, *J* = 16.8, 8.0, 1.2 Hz, 1H), 7.67 – 7.58 (m, 1H), 7.41 (dd, *J* = 7.4, 1.8 Hz, 1H), 7.29 (td, *J* = 7.4, 1.3 Hz, 1H), 7.23 (td, *J* = 7.7, 1.9 Hz, 1H), 4.41 – 4.29 (m, 2H), 3.42 (td, *J* = 6.8, 2.6 Hz, 2H), 1.91 – 1.74 (m, 4H), 1.48 – 1.31 (m, 8H). ^{13}C NMR ($CDCl_3$, 100 MHz): δ 152.08, 151.66, 142.30, 141.46, 133.68, 133.53, 130.48, 130.05, 128.96, 128.75, 127.94, 126.68, 126.51, 125.96, 34.26, 32.86, 29.85, 29.31, 28.98, 28.75, 28.18, 25.96. HRMS for $C_{20}H_{24}AuBrCl_2N_2NaO^+$ $[M+Na]^+$ calcd. 677.0007, found 677.0009.

2.2.3 Prodrug preparation

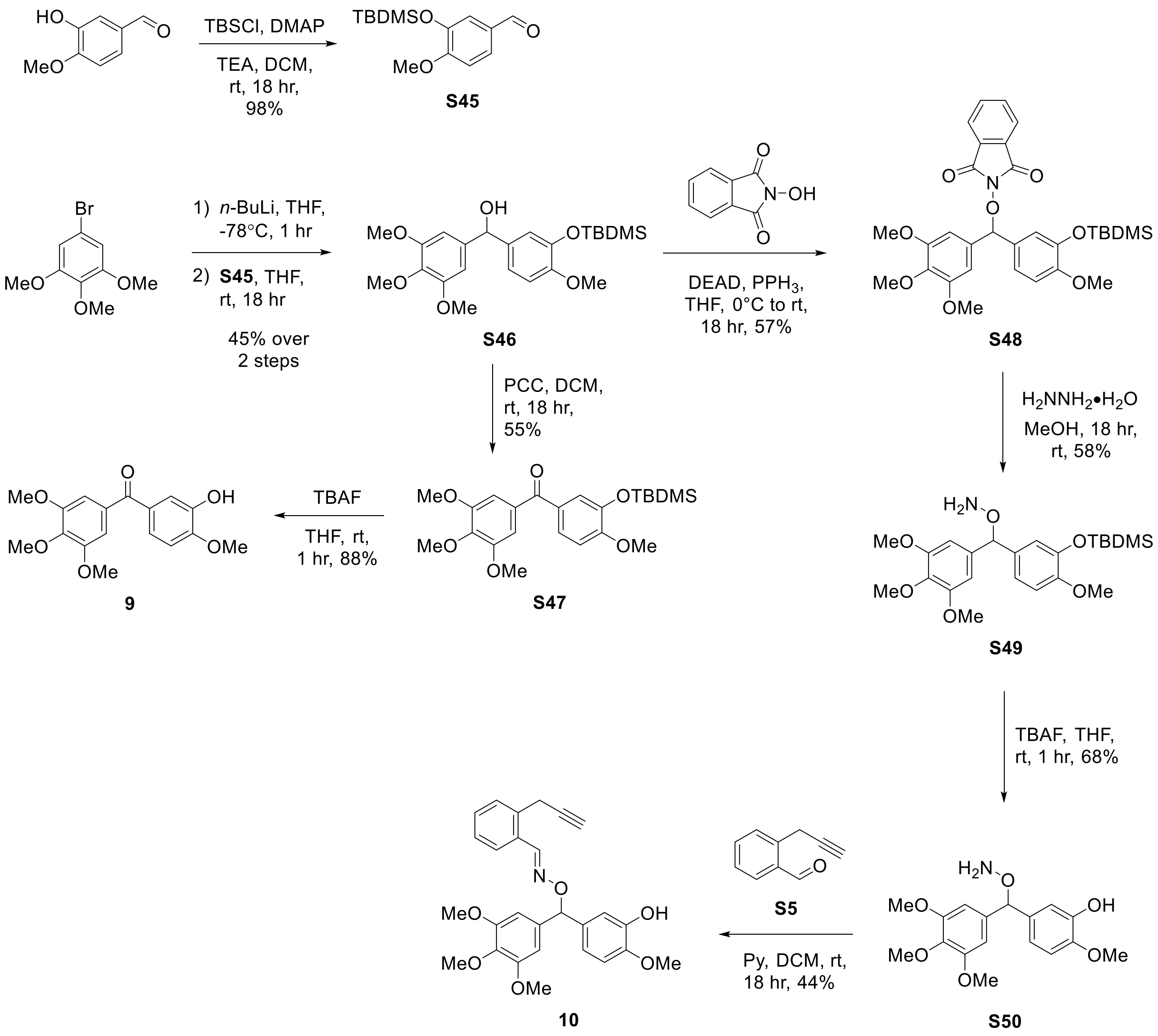

Scheme S6. Synthesis of phenstatin **9** and prodrug **10**.

Preparation of **S45**

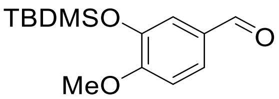

3-Hydroxy-4-methoxybenzaldehyde (2.00 g, 13.15 mmol) and 4-dimethylpyridine (48 mg, 0.39 mmol) were dissolved in DCM (10 mL). Triethylamine (2.75 mL, 19.72 mmol) were added to the solution and the mixture was cooled to 0 °C. The *tert*-Butyldimethylsilyl chloride (2.97 g, 19.72 mmol) was added to the reaction mixture slowly. The reaction mixture was then stirred for 18 hr at room temp. To workup, the mixture was washed with brine (30 mL) and the water layer was extracted with DCM (20 mL × 3). The combined organic phase was then dried over sodium sulfate. The solvent was then removed under vacuum. The crude product was purified by column chromatography on silica gel (Ethyl Acetate/Hexane= 1/19) to give **S45** (3.44 g, 98%). R_f = 0.32 (Ethyl Acetate/Hexane = 1/9). ^{1}H NMR ($CDCl_3$, 400 MHz): δ 9.81 (s, 1H), 7.47 (dd, *J* = 8.4, 2.0 Hz, 1H), 7.36 (d, *J* = 2.0 Hz, 1H), 6.94 (d, *J* = 8.2 Hz, 1H), 3.88 (s, 3H), 0.99 (s, 10H), 0.16 (s, 6H). ^{13}C NMR ($CDCl_3$, 101 MHz): δ 191.02, 156.73, 145.69, 130.33, 126.41, 120.17, 111.29, 55.69, 31.70, 25.76, 22.77, 18.54, 14.24, -4.52. HRMS for $C_{14}H_{23}O_3Si$ $[M+H]^+$ calcd. 267.1416, found 267.1428.

Preparation of **S46**

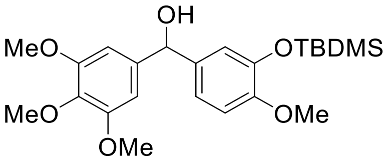

2.4 M *n*-BuLi in hexanes (6.76 mL, 16.22 mmol) was added dropwise into the solution of 5-bromo-1,2,3-trimethoxybenzene (2.94 g, 11.89 mmol) in anhydrous THF (4 mL) at -78 °C under argon. The mixture was stirred for 1 hr at -78 °C, then compound **S45** (2.88g, 10.81 mmol) in anhydrous THF (2 mL) was added dropwise to the reaction mixture. After stirred overnight at room temperature, the reaction mixture was quenched with sat. NH_4Cl (10 mL) and extracted with ethyl acetate. The organic phase was washed with brine and dried over sodium sulfate. The solvent was then removed under vacuum. The crude product was purified by column chromatography on silica gel (Ethyl Acetate/Hexane= 3/7) to give **S46** (2.10 g, 45%). R_f = 0.33 (Ethyl Acetate/Hexane = 2/3). ^{1}H NMR ($CDCl_3$, 400 MHz): δ 6.89 (dd, *J* = 8.3, 2.2 Hz, 1H), 6.84 (d, *J* = 2.2 Hz, 1H), 6.79 (d, *J* = 8.3 Hz, 1H), 6.56 (s, 2H), 5.64 (s, 1H), 3.80 (d, *J* = 2.1 Hz, 9H), 3.78 (s, 3H), 0.96 (s, 9H), 0.12 (s, 6H). ^{13}C NMR ($CDCl_3$, 101 MHz): δ 153.21, 150.53, 145.00, 139.86, 137.13, 136.54, 119.97, 119.54, 111.99, 103.49, 75.79, 60.89, 56.11, 55.61, 25.80, 18.54, -4.51. HRMS for $C_{23}H_{34}NaO_6Si$ $[M+Na]^+$ calcd. 457.2022, found 457.2025.

Preparation of **S47**

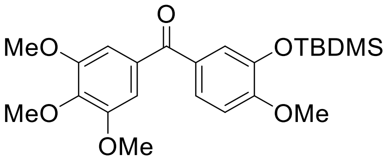

To a solution of **S46** (250 mg, 0.58mmol) in DCM (5 mL) was added pyridiniumchlorochromate (370 mg, 1.73 mmol) and celite (300 mg). After stirring for 18 hr at room temperature, the reaction mixture was filtered by celite. The solution was concentrated under vacuum purified by column chromatography on silica gel (Ethyl Acetate/Hexane= 1/4) to give **S47** (138 mg, 55%). R_f = 0.67 (Ethyl Acetate/Hexane = 2/3). ^{1}H NMR ($CDCl_3$, 400 MHz): δ 7.44 (dd, *J* = 8.4, 2.2 Hz, 1H), 7.36 (d, *J* = 2.1 Hz, 1H), 7.02 (s, 2H), 6.90 (d, *J* = 8.4 Hz, 1H), 3.93 (s, 3H), 3.89 (s, 3H), 3.87 (s, 6H), 0.99 (s, 9H), 0.17 (s, 6H). ^{13}C NMR ($CDCl_3$, 101 MHz): δ 194.68, 155.05, 152.91, 144.80, 141.66, 133.49, 130.56, 125.41, 122.46, 110.85, 107.56, 61.08, 56.40, 55.62, 25.78, 18.55, -4.43. HRMS for $C_{23}H_{32}NaO_6Si$ $[M+Na]^+$ calcd. 455.1866, found 455.1865.

Preparation of **S48**

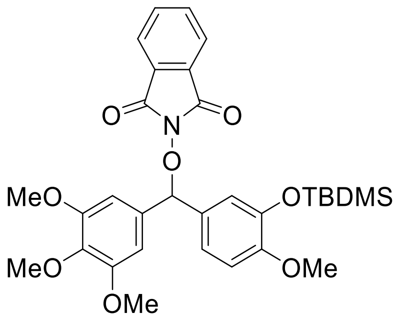

Diethyl azodicarboxylate (0.76 mL, 4.83 mmol) was added dropwise to a solution of **S46** (2.10 g, 4.83 mmol), N-Hydroxyphthalimide (788 mg, 4.83 mmol) and triphenylphosphine (1.39 g, 5.32 mmol) in anhydrous THF (4 mL) under argon at 0 °C. After stirring overnight at room temperature, the reaction was diluted with EtOAc. Then the mixture was washed with water and brine. The organic phase was dried over sodium sulfate. The solvent was then removed under vacuum. The crude product was purified by column chromatography on silica gel (Ethyl Acetate/Hexane= 1/4) to give **S48** (1.60 g, 57%). R_f = 0.41 (Ethyl Acetate/Hexane = 2/3). ^{1}H NMR ($CDCl_3$, 400 MHz): δ 7.77 – 7.72 (m, 2H), 7.71 – 7.66 (m, 2H), 7.06 (dd, *J* = 8.4, 2.2 Hz, 1H), 7.00 (d, *J* = 2.2 Hz, 1H), 6.83 – 6.78 (m, 3H), 6.41 (s, 1H), 3.85 (s, 6H), 3.82 (s, 3H), 3.78 (s, 3H), 0.95 (s, 9H), 0.10 (d, *J* = 9.2 Hz, 6H). ^{13}C NMR ($CDCl_3$, 101 MHz): δ 163.87, 153.16, 151.63, 144.82, 137.96, 134.44, 133.62, 130.13, 129.05, 123.47, 122.46, 121.57, 111.73, 105.04, 102.27, 89.30, 60.94, 56.27, 56.23, 55.59, 25.82, 18.55, -4.54. HRMS for $C_{31}H_{37}NNaO_8Si$ $[M+Na]^+$ calcd. 602.2186, found 602.2184.

Preparation of **S49**

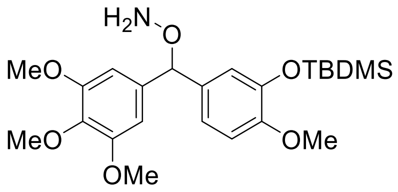

Hydrazine hydrate (0.80 mL, 8.28 mmol, 50%) was added to a solution of **S48** (1.60g, 2.76 mmol) in MeOH (5 mL) at room temperature. After overnight stirring, the suspension was diluted with EtOAc and washed with H_2O and brine. The organic phase was dried over sodium sulfate. The solvent was then removed under vacuum. The crude product was purified by column chromatography on silica gel (Ethyl Acetate/Hexane= 2/3) to give **S49** (0.72 g, 58%). R_f = 0.19 (Ethyl Acetate/Hexane = 2/3). ^{1}H NMR ($CDCl_3$, 400 MHz): δ 6.88 (dd, *J* = 8.2, 2.2 Hz, 1H), 6.85 – 6.79 (m, 2H), 6.56 (s, 2H), 5.47 (s, 1H), 3.83 (s, 9H), 3.79 (s, 3H), 0.97 (s, 9H), 0.12 (s, 6H). ^{13}C NMR ($CDCl_3$, 101 MHz): δ 153.44, 150.83, 145.13, 137.48, 136.84, 133.20, 120.95, 120.12, 112.04, 104.15, 88.53, 60.95, 56.19, 55.67, 25.85, 18.60, 14.56, -4.43. HRMS for $C_{23}H_{35}NNaO_6Si$ $[M+Na]^+$ calcd. 472.2131, found 472.2129.

Preparation of **S50**

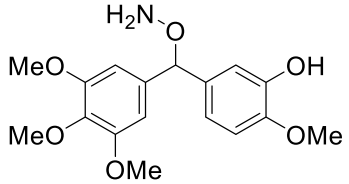

To a solution of **S49** (360 mg, 0.80 mmol) dissolved in THF (2 ml), TBAF (1.0 M in THF, 2.00 ml, 2.00 mmol) was added dropwise. The reaction mixture was then stirred for 1 hr at room temp. To workup, the mixture was quenched with a saturated NH_4Cl solution. Extraction using ethyl acetate was then performed, followed by washing with water, brine, and then dried over sodium sulfate. The solvent was then removed under vacuum. The crude was purified by column chromatography on silica gel (Ethyl Acetate/Hexane= 7/3) to give **S50** (182 mg, 68%). R_f = 0.40 (Ethyl Acetate/Hexane = 3/1). ^{1}H NMR ($CDCl_3$, 400 MHz): δ 6.92 (d, *J* = 1.8 Hz, 1H), 6.87 – 6.80 (m, 2H), 6.58 (s, 2H), 5.65 (s, 1H), 5.47 (s, 1H), 5.45 (s, 2H), 3.88

(s, 3H), 3.84 (s, 6H), 3.82 (s, 3H). ^{13}C NMR ($CDCl_3$, 101 MHz): δ 153.48, 146.40, 145.77, 137.48, 136.75, 134.11, 119.20, 113.62, 110.62, 104.00, 88.58, 60.94, 56.23, 56.11. HRMS for $C_{17}H_{20}NaO_6$ $[M-H]^+$ calcd. 334.1291, found 334.1296.

Preparation of **9**

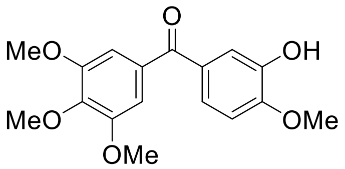

To a solution of **S47** (138 mg, 0.32 mmol) dissolved in THF (1 ml), TBAF (1.0 M in THF, 0.80 ml, 0.80 mmol) was added dropwise. The reaction mixture was then stirred for 1 hr at room temp. To workup, the mixture was quenched with a saturated NH_4Cl solution. Extraction using ethyl acetate was then performed, followed by washing with water, brine, and then dried over sodium sulfate. The solvent was then removed under vacuum. The crude was purified by column chromatography on silica gel (Ethyl Acetate/Hexane= 2/3) to give **9** (89 mg, 88%). R_f = 0.24 (Ethyl Acetate/Hexane = 2/3). ^{1}H NMR ($CDCl_3$, 400 MHz): δ 7.44 (d, *J* = 2.1 Hz, 1H), 7.39 (dd, *J* = 8.3, 2.1 Hz, 1H), 7.03 (s, 2H), 6.92 (d, *J* = 8.4 Hz, 1H), 3.98 (s, 3H), 3.93 (s, 3H), 3.88 (s, 6H). ^{13}C NMR ($CDCl_3$, 101 MHz): δ 194.82, 152.93, 150.35, 145.46, 141.76, 133.31, 131.21, 123.79, 116.36, 109.83, 107.64, 61.11, 56.44, 56.25. HRMS for $C_{17}H_{18}NaO_6$ $[M+Na]^+$ calcd. 341.1001, found 341.0998.

Preparation of **10**

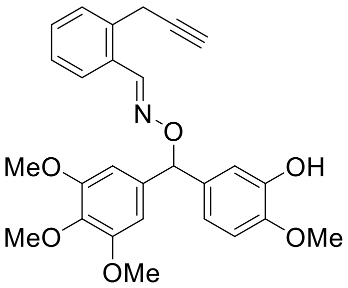

Pyridine (0.16 mL, 1.94 mmol) was added to a solution of **S50** (179 mg, 0.53 mmol) and **S5** (70 mg, 0.49 mmol) in DCM (5 mL) at room temperature. After stirring overnight, the solution was concentrated under vacuum. Flash column chromatography with 40% EtOAc/Hex was used to purify the desired compound **10** (98 mg, 44%). R_f = 0.21 (EtOAc/Hex, 2:3). ^{1}H NMR ($CDCl_3$, 400 MHz): δ 8.50 (s, 1H), 7.55 (ddd, *J* = 16.8, 7.8, 1.3 Hz, 2H), 7.34 (td, *J* = 7.6, 1.5 Hz, 1H), 7.26 – 7.22 (t, 1H), 6.98 (d, *J* = 2.0 Hz, 1H), 6.90 – 6.82 (m, 2H), 6.61 (s, 2H), 6.19 (s, 1H), 5.62 (s, 1H), 3.88 (s, 3H), 3.84 (s, 9H), 3.69 (d, *J* = 2.7 Hz, 2H), 2.21 (t, *J* = 2.7 Hz, 1H). ^{13}C NMR ($CDCl_3$, 101 MHz): δ 153.32, 148.63, 146.31, 145.64, 137.50, 136.88, 134.91, 134.36, 130.03, 129.99, 129.40, 128.68, 127.29, 119.54, 114.00, 110.51, 104.67, 86.95, 81.54, 71.58, 60.96, 56.26, 56.11, 23.56. HRMS for $C_{27}H_{26}NaO_6$ $[M-H]^+$ calcd. 460.1760, found 460.1762.

2.3 HPLC methods and standard curves

Reverse-phase HPLC was used to analyze compounds as indicated. The Shimadzu system (Kyoto, Japan) employed two LC-40D solvent pumps outfitted with a SPD-40 UV/Visible detector, a RF-20Axs fluorescence detector, and a SIL-40 autosampler. The columns used were an analytical 4.6 × 250 mm Cosmosil 5C_{18}-AR-300 and an analytical 4.6 × 250 mm Cosmosil 5CN-MS from Nacalai Tesque (Kyoto, Japan). Samples were eluted using a combination of mobile phases A (100% H_2O), B (100% acetonitrile), C (100% H_2O with 0.1% TFA), D (100% acetonitrile with 0.1% TFA). The detector was set to 220 and 254 nm. The different elution methods that were employed in this study are shown in Table S1.

Table S1. Gradient profiles for HPLC studies

	**Flow rate (ml/min)**	**Time (min)**	**%A (100% H_2O)**	**%B (100% ACN)**	**%C (H_2O with 0.1% TFA)**	**%D (ACN with 0.1% TFA)**
Method 1	1.0	0	95	5		
C_{18} column		5	95	5		
		25	10	90		
		35	10	90		
		37	95	5		
		40	95	5		
Method 2	1.0	0			95	5
C_{18} column		5			95	5
		25			10	90
		35			10	90
		37			95	5
		40			95	5
Method 3	1.0	0	70	30		
CN column		7	70	30		
		20	10	90		
		25	10	90		
		28	70	30		
		33	70	30		

To determine yields from HPLC analysis, calibration curves were first constructed using product standards of known amounts (Figures S2-S12). In general, product identification from reaction mixtures were determined by MS analysis of peaks and their retention time comparison to known product standards. Following peak integration, yields can be calculated based on these calibration curves.

Product Structure	HPLC Method	Calibration Curve
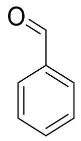 **3a**	Method 1	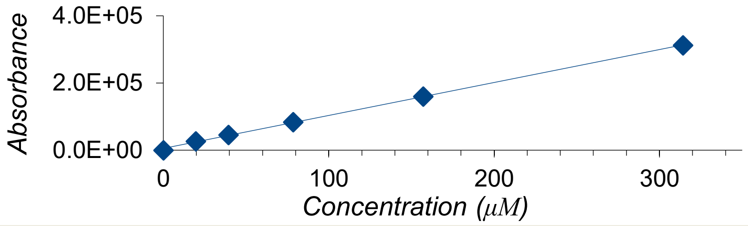

$R^2 = 1$		Value
	Slope	969,620.1
	Intercept	7,727.2

HPLC trace:

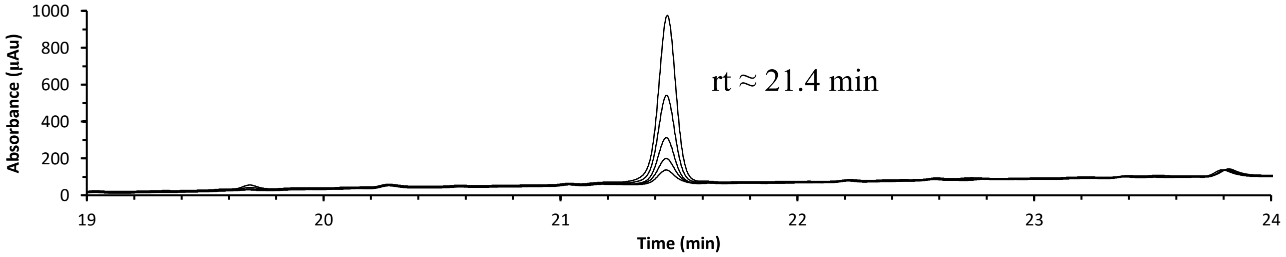

Figure S2. HPLC calibration curve of released product **3a**

Product Structure	HPLC Method	Calibration Curve
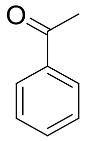 **3b**	Method 1	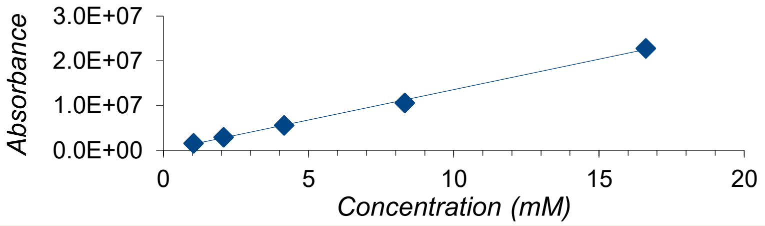

$R^2 = 0.9982$		Value
	Slope	1,358,999.3
	Intercept	-10,915.7

HPLC trace:

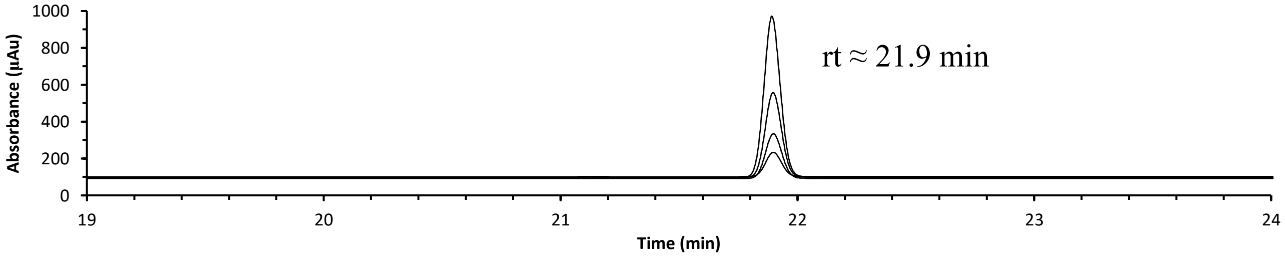

Figure S3. HPLC calibration curve of released product **3b**

Product Structure	HPLC Method	Calibration Curve
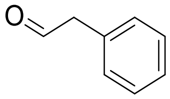 **5a**	Method 2	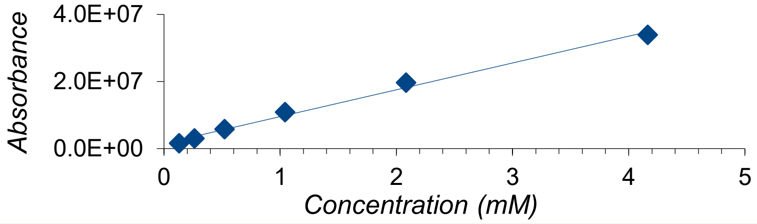

**R^2 = 0.993**		Value
	Slope	7,991,729.2
	Intercept	1,543,874.3

HPLC trace:

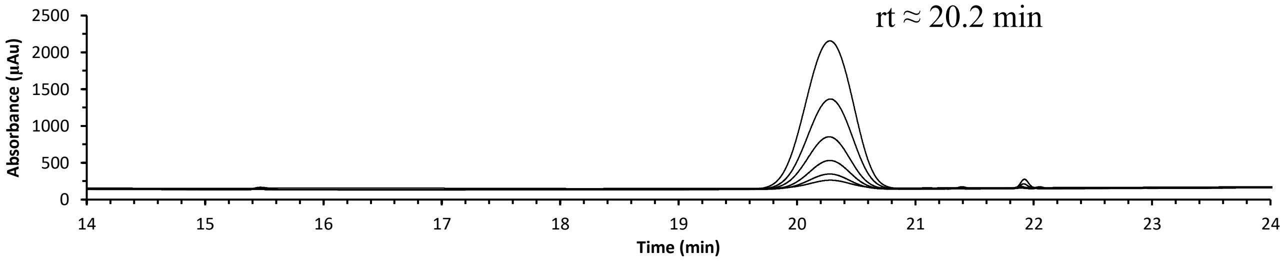

Figure S4. HPLC calibration curve of released product **5a**

Product Structure	HPLC Method	Calibration Curve
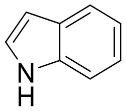 **5b**	Method 2	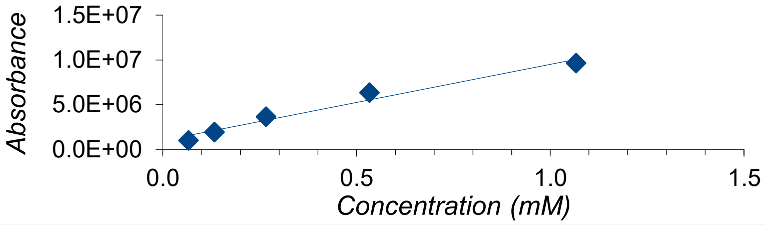

**R^2 = 0.9723**		Value
	Slope	8,510,996.2
	Intercept	989,636.2

HPLC trace:

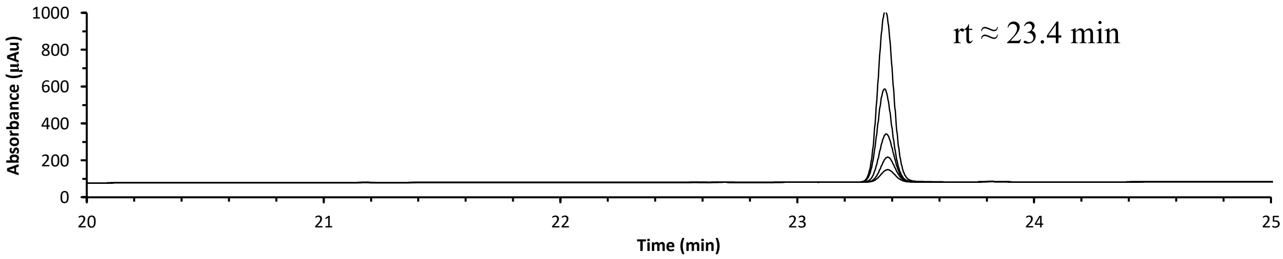

Figure S5. HPLC calibration curve of released product **5b**

Product Structure	HPLC Method	Calibration Curve
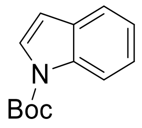 **5c**	Method 3	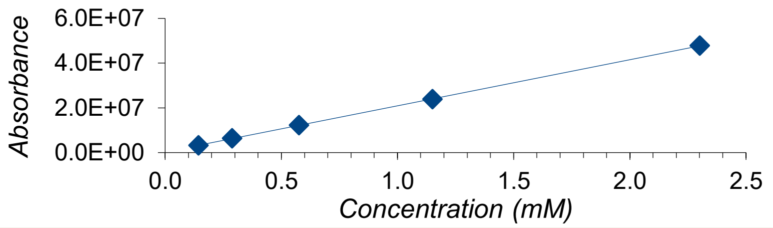

**R^2 = 1**		Value
	Slope	20,605,273.9
	Intercept	312,851.9

HPLC trace:

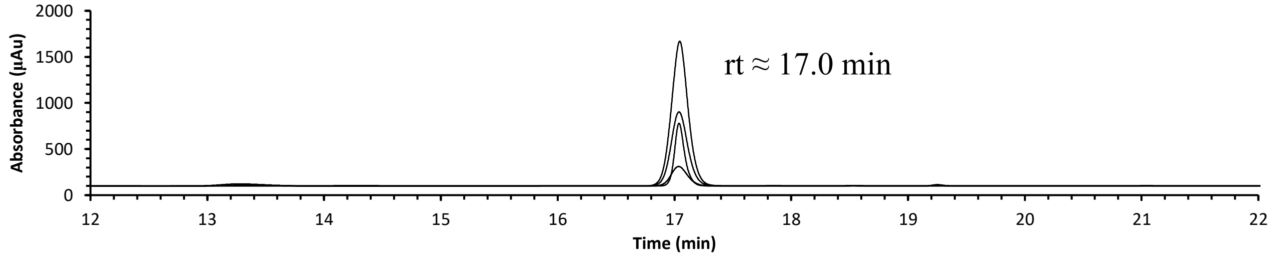

Figure S6. HPLC calibration curve of released product **5c**

Product Structure	HPLC Method	Calibration Curve
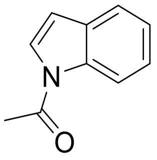 **5d**	Method 2	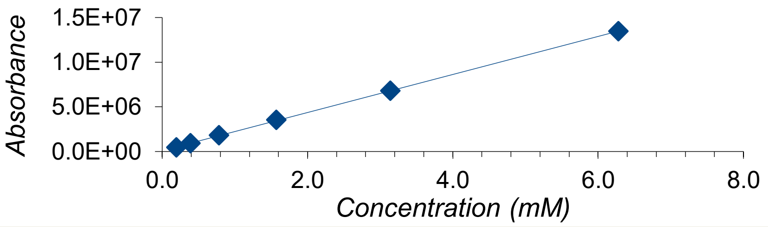

**R^2 = 0.999**		Value
	Slope	2,129,660.8
	Intercept	107,748.9

HPLC trace:

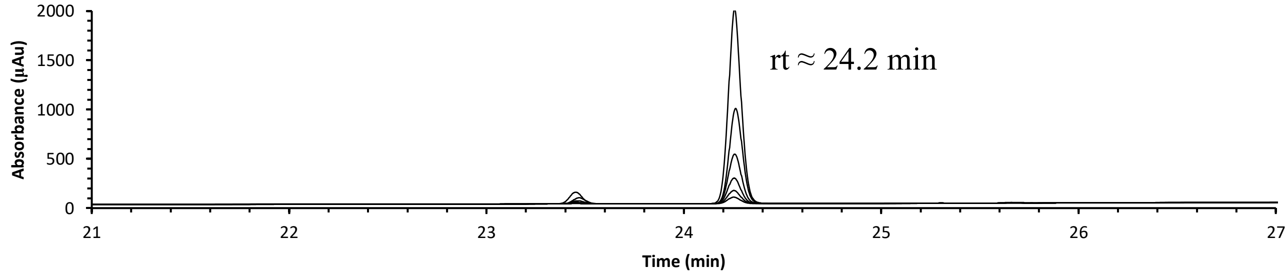

Figure S7. HPLC calibration curve of released product **5d**

Product Structure	HPLC Method	Calibration Curve
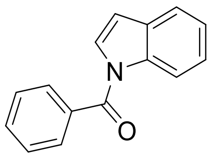 **5e**	Method 2	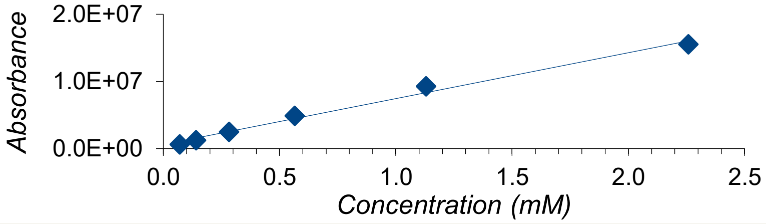

R^2 = 0.990	Value
Slope	6,831,555.7
Intercept	607,764.6

HPLC trace:

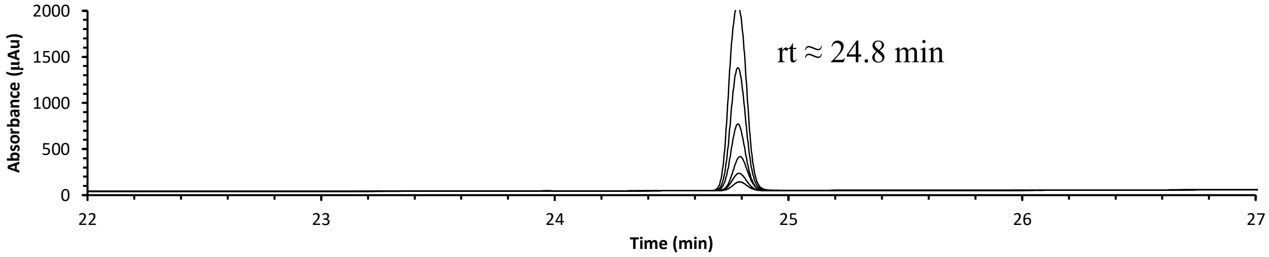

Figure S8. HPLC calibration curve of released product **5e**

Product Structure	HPLC Method	Calibration Curve
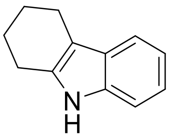 **5f**	Method 2	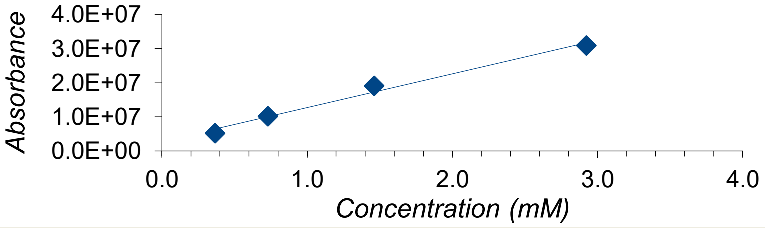

R^2 = 0.985	Value
Slope	9,895,333.3
Intercept	2,836,525.0

HPLC trace:

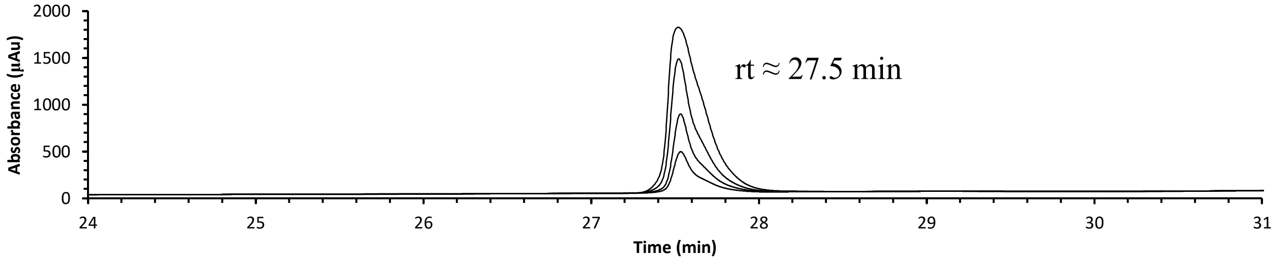

Figure S9. HPLC calibration curve of released product **5f**

Product Structure	HPLC Method	Calibration Curve
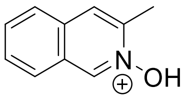 **6c**	Method 1	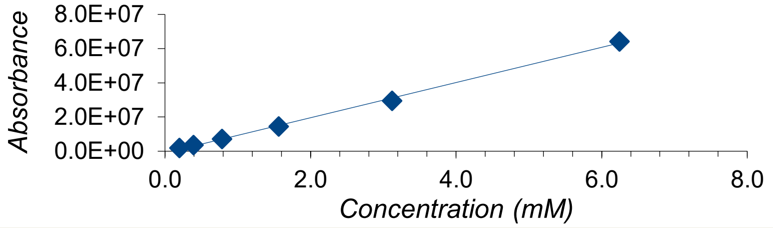

**R^2 = 0.998**		Value
	Slope	10,291,138.8
	Intercept	-1,020,062.5

HPLC trace:

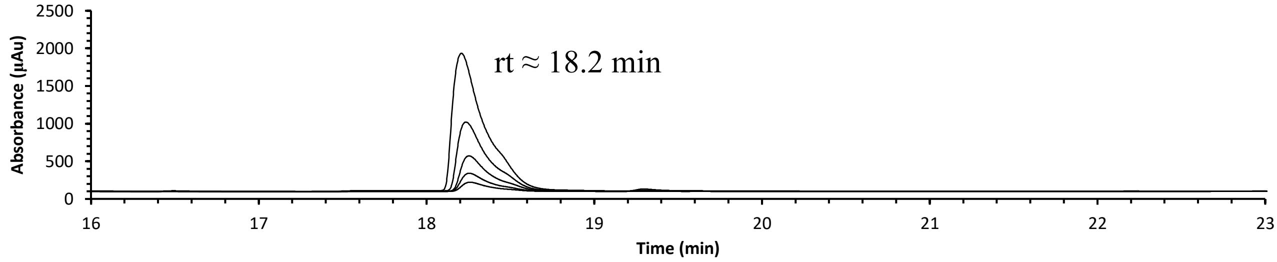

Figure S10. HPLC calibration curve of product **6c**

Product Structure	HPLC Method	Calibration Curve
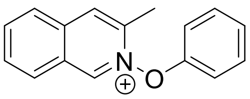 **6d**	Method 1	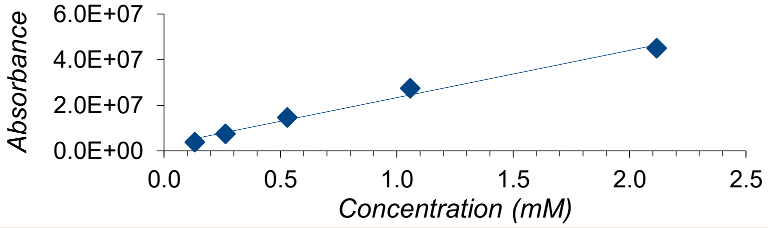

**R^2 = 0.988**		Value
	Slope	20,729,715.2
	Intercept	2,610,899.0

HPLC trace:

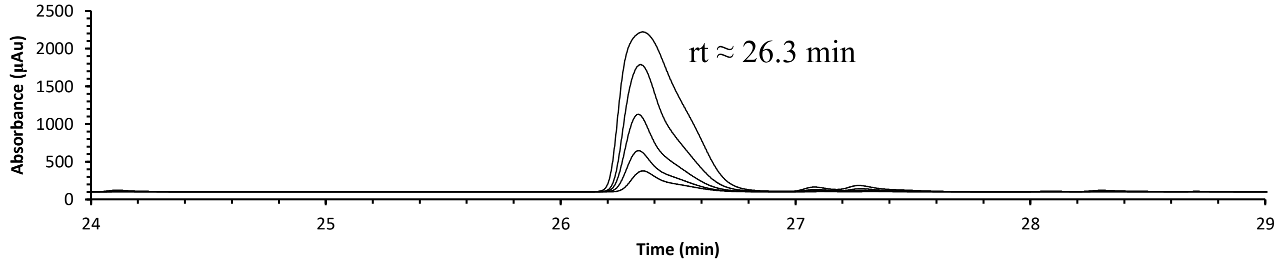

Figure S11. HPLC calibration curve of product **6d**

Product Structure	HPLC Method	Calibration Curve
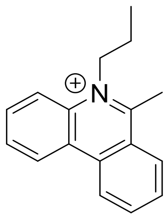 **8**	Method 2	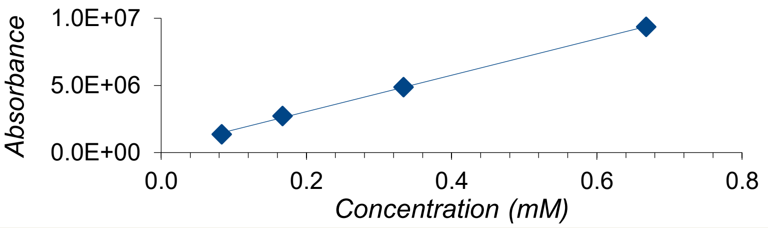 **R^2 = 0.999** — Value: Slope 13,548,859.8; Intercept 338,893.9

HPLC trace:

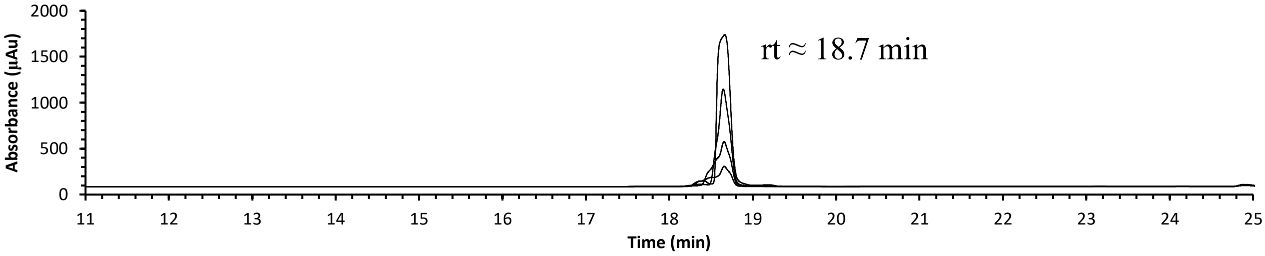

Figure S12. HPLC calibration curve of product **8**

2.4 Reactivity screening using transition metal catalysts

To investigate the reactivity of **1a**-**b** and **4a-f**, 1 mg of each substrate (3.60-4.01 µmol) was dissolved in 25 µl of organic solvent. Depending on the situation, catalysts **Au1-Au7** (mol% adjusted as indicated) were dissolved in either 25 µl of either organic solvent or PBS buffer. The substrate and catalyst were mixed, along with sufficient solvent to make up a total volume of 100 µl (unless otherwise stated). These mixtures were then placed in a sand bath at an adjusted temperature of 37°C without stirring. To workup, mixtures were quenched with 1 mM dodecanethiol in ACN (100 µl) and 1.0M HCL (50 µl). The solutions were then filtered and injected (10 µl) onto a HPLC with an autosampler using various HPLC methods (details in Figure S2-S12).

Data regarding aldehyde release of **3a** from substrate **1a** is shown in two parts; through transition metal catalyst screening in Table S2, and through gold-catalysis in Table S3. Data regarding gold-catalyzed ketone release of **3b** from substrate **1b** is shown in Table S4. Data regarding the indole synthesis of **5a-f** from substrates **4a-f** is shown in Tables S5-S10.

Table S2. Transition metal screening of compound **1a-b** for the release of carbonyls **3a-b**

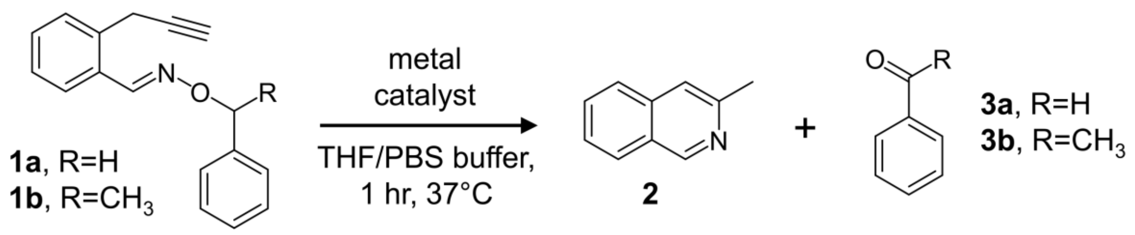

Entry	Catalyst	Mol%	Yield of **3a** (%)[a]	Turnover (TON)
1	**Au1**	5	70	13.9
2	**Au5**	5	4	0.8
3	**Pd1**	5	42	8.3
4	**Pd2**	5	20	4.1
5	**Pt1**	5	0	-
6	**Ag1**	5	0	-
7	**Ru1**	5	0	-
8	**Cu1**	5	0	-
9	**Cu2**	5	0	-
Entry	Catalyst	Mol%	Yield of **3b** (%)[a]	Turnover (TON)
10	**Au1**	5	52	10.5
11	**Au5**	5	2	0.3
12	**Pd1**	5	34	6.9
13	**Pd2**	5	5	1.1
14	**Pt1**	5	0	-
15	**Ag1**	5	0	-
16	**Ru1**	5	0	-
17	**Cu1**	5	0	-
18	**Cu2**	5	0	-

[a]Yields determined by HPLC (peak retention times compared to product standards, followed by MS analysis for confirmation, and then calculation of yields based on product standard curves). All reactions were standardized to 3.80-4.01 μmol of **1a** in 100 μl of solvent.

Table S3. Gold-catalyzed reactivity of compound **1a** for the release of aldehyde **3a**

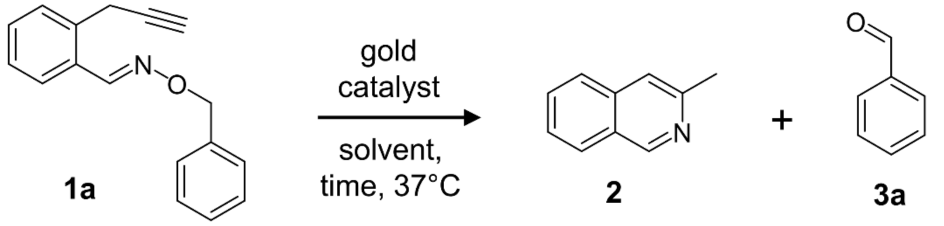

Entry	Catalyst	Mol%	Solvent	Time (hr)	Release yield of **3a** (%)[a]	Turnover (TON)
19	-	-	50% THF in PBS buffer	1	0	-
20	**Au1**	20	50% THF in PBS buffer	1	94	4.7
21	**Au1**	10	50% THF in PBS buffer	1	92	9.2
22	**Au1**	5	50% THF in PBS buffer	1	70	13.9
23	**Au1**	2	50% THF in PBS buffer	1	26	13.0
24	**Au1**	1	50% THF in PBS buffer	1	13	13.3
25	**Au1**	5	THF	1	76	15.3
26	**Au1**	5	Dioxane	1	52	10.5
27	**Au1**	5	50% Dioxane in PBS buffer	1	56	11.1
28	**Au1**	5	DMSO	1	24	4.8
29	**Au1**	5	50% DMSO in PBS buffer	1	33	6.7
30	**Au1**	5	ACN	1	47	9.3
31	**Au1**	5	50% ACN in PBS buffer	1	50	10.0
32	**Au1**	5	DMF	1	42	8.4
33	**Au1**	5	50% DMF in PBS buffer	1	60	11.9
34	**Au2**	5	THF	1	73	14.5
35	**Au2**	5	50% THF in PBS buffer	1	69	13.9
36	**Au2**	5	Dioxane	1	42	8.4
37	**Au2**	5	50% Dioxane in PBS buffer	1	48	9.6
38	**Au2**	5	DMSO	1	33	6.6
39	**Au2**	5	50% DMSO in PBS buffer	1	49	9.7
40	**Au2**	5	ACN	1	62	12.4
41	**Au2**	5	50% ACN in PBS buffer	1	50	10.1
42	**Au2**	5	DMF	1	62	12.4
43	**Au2**	5	50% DMF in PBS buffer	1	48	9.7
44	**Au3**	5	THF	1	51	10.3
45	**Au3**	5	50% THF in PBS buffer	1	55	10.9
46	**Au3**	5	Dioxane	1	43	8.6
47	**Au3**	5	50% Dioxane in PBS buffer	1	13	2.7
48	**Au3**	5	DMSO	1	45	9.1
49	**Au3**	5	50% DMSO in PBS buffer	1	60	12.0
50	**Au3**	5	ACN	1	81	16.2
51	**Au3**	5	50% ACN in PBS buffer	1	44	8.9
52	**Au3**	5	DMF	1	24	4.7
53	**Au3**	5	50% DMF in PBS buffer	1	19	3.8

[a]Yields determined by HPLC (peak retention times compared to product standards, followed by MS analysis for confirmation, and then calculation of yields based on product standard curves). [b]Catalyst added as a suspension. All reactions were standardized to 4.01 μmol of **1a** in 100 μl of solvent.

Table S3 (continued). Gold-catalyzed reactivity of compound **1a** for the release of aldehyde **3a**

Entry	Catalyst	Mol%	Solvent	Time (hr)	Release yield of **3a** (%)[a]	Turnover (TON)
54	**Au4**	5	THF	1	0	-
55	**Au4**	5	50% THF in PBS buffer	1	8	1.6
56	**Au4**	5	Dioxane	1	0[b]	-
57	**Au4**	5	50% Dioxane in PBS buffer	1	0[b]	-
58	**Au4**	5	DMSO	1	0	-
59	**Au4**	5	50% DMSO in PBS buffer	1	3	0.6
60	**Au4**	5	ACN	1	5	1.0
61	**Au4**	5	50% ACN in PBS buffer	1	22	4.4
62	**Au4**	5	DMF	1	0	-
63	**Au4**	5	50% DMF in PBS buffer	1	7	1.4
64	**Au5**	5	THF	1	0	-
65	**Au5**	5	50% THF in PBS buffer	1	4	0.8
66	**Au5**	5	Dioxane	1	0	-
67	**Au5**	5	50% Dioxane in PBS buffer	1	3	0.6
68	**Au5**	5	DMSO	1	0	-
69	**Au5**	5	50% DMSO in PBS buffer	1	0	-
70	**Au5**	5	ACN	1	0	-
71	**Au5**	5	50% ACN in PBS buffer	1	17	3.4
72	**Au5**	5	DMF	1	0	-
73	**Au5**	5	50% DMF in PBS buffer	1	0	-
74	**Au6**	5	THF	1	0	-
75	**Au6**	5	50% THF in PBS buffer	1	0	-
76	**Au6**	5	Dioxane	1	0	-
77	**Au6**	5	50% Dioxane in PBS buffer	1	0	-
78	**Au6**	5	DMSO	1	0	-
79	**Au6**	5	50% DMSO in PBS buffer	1	0	-
80	**Au6**	5	ACN	1	0[b]	-
81	**Au6**	5	50% ACN in PBS buffer	1	0[b]	-
82	**Au6**	5	DMF	1	0	-
83	**Au6**	5	50% DMF in PBS buffer	1	0	-
84	**Au7**	5	THF	1	0	-
85	**Au7**	5	50% THF in PBS buffer	1	0	-
86	**Au7**	5	Dioxane	1	3	0.5
87	**Au7**	5	50% Dioxane in PBS buffer	1	21	4.2
88	**Au7**	5	DMSO	1	0[b]	-
89	**Au7**	5	50% DMSO in PBS buffer	1	14[b]	2.9
90	**Au7**	5	ACN	1	24[b]	4.7
91	**Au7**	5	50% ACN in PBS buffer	1	3[b]	0.5
92	**Au7**	5	DMF	1	0	-
93	**Au7**	5	50% DMF in PBS buffer	1	7	1.4

[a]Yields determined by HPLC (peak retention times compared to product standards, followed by MS analysis for confirmation, and then calculation of yields based on product standard curves). [b]Catalyst added as a suspension. All reactions were standardized to 4.01 µmol of **1a** in 100 µl of solvent.

Table S4. Gold-catalyzed reactivity of compound **1b** for the release of ketone **3b**

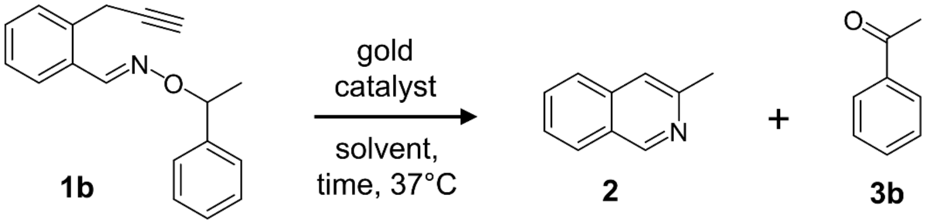

Entry	Catalyst	Mol%	Solvent	Time (hr)	Release yield of **3b** (%)[a]	Turnover (TON)[a]
94	-	-	50% THF in PBS buffer	1	0	-
95	**Au1**	20	50% THF in PBS buffer	1	48	2.4
96	**Au1**	10	50% THF in PBS buffer	1	52	5.2
97	**Au1**	5	50% THF in PBS buffer	1	52	10.5
98	**Au1**	2	50% THF in PBS buffer	1	12	6.1
99	**Au1**	1	50% THF in PBS buffer	1	4	4.3
100	**Au1**	5	THF	1	37	7.4
101	**Au1**	5	Dioxane	1	17[b]	3.5
102	**Au1**	5	50% Dioxane in PBS buffer	1	28	5.6
103	**Au1**	5	DMSO	1	2	0.4
104	**Au1**	5	50% DMSO in PBS buffer	1	8	1.7
105	**Au1**	5	ACN	1	25	5.0
106	**Au1**	5	50% ACN in PBS buffer	1	42	8.4
107	**Au1**	5	DMF	1	16	3.3
108	**Au1**	5	50% DMF in PBS buffer	1	30	6.1
109	**Au2**	5	THF	1	24	4.8
110	**Au2**	5	50% THF in PBS buffer	1	36	7.2
111	**Au2**	5	Dioxane	1	18	3.7
112	**Au2**	5	50% Dioxane in PBS buffer	1	38	7.5
113	**Au2**	5	DMSO	1	22	4.4
114	**Au2**	5	50% DMSO in PBS buffer	1	28	5.6
115	**Au2**	5	ACN	1	19	3.8
116	**Au2**	5	50% ACN in PBS buffer	1	35	7.0
117	**Au2**	5	DMF	1	11	2.2
118	**Au2**	5	50% DMF in PBS buffer	1	31	6.2
119	**Au3**	5	THF	1	48	9.5
120	**Au3**	5	50% THF in PBS buffer	1	34	6.7
121	**Au3**	5	Dioxane	1	55	10.9
122	**Au3**	5	50% Dioxane in PBS buffer	1	18	3.7
123	**Au3**	5	DMSO	1	46	9.1
124	**Au3**	5	50% DMSO in PBS buffer	1	49	9.9
125	**Au3**	5	ACN	1	45	9.1
126	**Au3**	5	50% ACN in PBS buffer	1	39	7.7
127	**Au3**	5	DMF	1	36	7.1
128	**Au3**	5	50% DMF in PBS buffer	1	38	7.6

[a]Yields determined by HPLC (peak retention times compared to product standards, followed by MS analysis for confirmation, and then calculation of yields based on product standard curves). [b]Catalyst added as a suspension. All reactions were standardized to 3.80 µmol of **1b** in 100 µl of solvent.

Table S4 (continued). Gold-catalyzed reactivity of compound **1b** for the release of ketone **3b**

Entry	Catalyst	Mol%	Solvent	Time (hr)	Release yield of **3b** (%)[a]	Turnover (TON)[a]
129	**Au4**	5	THF	1	0	-
130	**Au4**	5	50% THF in PBS buffer	1	5	1.1
131	**Au4**	5	Dioxane	1	0[b]	-
132	**Au4**	5	50% Dioxane in PBS buffer	1	4[b]	0.8
133	**Au4**	5	DMSO	1	0	-
134	**Au4**	5	50% DMSO in PBS buffer	1	3	0.5
135	**Au4**	5	ACN	1	2	0.4
136	**Au4**	5	50% ACN in PBS buffer	1	20	4.0
137	**Au4**	5	DMF	1	0	-
138	**Au4**	5	50% DMF in PBS buffer	1	5	1.1
139	**Au5**	5	THF	1	0	-
140	**Au5**	5	50% THF in PBS buffer	1	2	0.3
141	**Au5**	5	Dioxane	1	0	-
142	**Au5**	5	50% Dioxane in PBS buffer	1	0	-
143	**Au5**	5	DMSO	1	0	-
144	**Au5**	5	50% DMSO in PBS buffer	1	0	-
145	**Au5**	5	ACN	1	0	-
146	**Au5**	5	50% ACN in PBS buffer	1	17	3.4
147	**Au5**	5	DMF	1	0	-
148	**Au5**	5	50% DMF in PBS buffer	1	0	-
149	**Au6**	5	THF	1	0	-
150	**Au6**	5	50% THF in PBS buffer	1	0	-
151	**Au6**	5	Dioxane	1	0	-
152	**Au6**	5	50% Dioxane in PBS buffer	1	0	-
153	**Au6**	5	DMSO	1	0	-
154	**Au6**	5	50% DMSO in PBS buffer	1	0	-
155	**Au6**	5	ACN	1	0[b]	-
156	**Au6**	5	50% ACN in PBS buffer	1	5[b]	1.1
157	**Au6**	5	DMF	1	0	-
158	**Au6**	5	50% DMF in PBS buffer	1	0	-
159	**Au7**	5	THF	1	1	0.3
160	**Au7**	5	50% THF in PBS buffer	1	7	1.5
161	**Au7**	5	Dioxane	1	0	-
162	**Au7**	5	50% Dioxane in PBS buffer	1	7	1.4
163	**Au7**	5	DMSO	1	0[b]	-
164	**Au7**	5	50% DMSO in PBS buffer	1	0[b]	-
179	**Au7**	5	ACN	1	5[b]	0.9
180	**Au7**	5	50% ACN in PBS buffer	1	28[b]	5.6
181	**Au7**	5	DMF	1	0	-
182	**Au7**	5	50% DMF in PBS buffer	1	4	0.8

[a]Yields determined by HPLC (peak retention times compared to product standards, followed by MS analysis for confirmation, and then calculation of yields based on product standard curves). [b]Catalyst added as a suspension. All reactions were standardized to 3.80 μmol of **1b** in 100 μl of solvent.

Table S5. Gold-catalyzed reactivity of compound **4a** for the synthesis of aldehyde **5a**

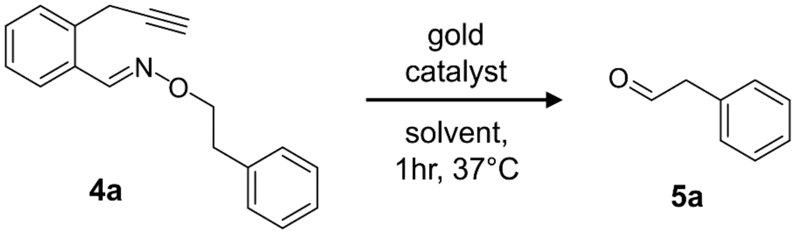

Entry	Catalyst	Mol%	Solvent	Time (hr)	Yield of **5a** (%)[a]	Turnover (TON)[a]
183	-	-	50% THF in PBS buffer	1	0	-
184	**Au1**	5	50% THF in PBS buffer	1	11	2.3
185	**Au1**	5	50% DMSO in PBS buffer	1	18	3.5
186	**Au1**	5	50% ACN in PBS buffer	1	19	3.9
187	**Au3**	5	50% THF in PBS buffer	1	10	2.0
188	**Au3**	5	50% DMSO in PBS buffer	1	14	2.9
189	**Au3**	5	50% ACN in PBS buffer	1	17	3.5

[a]Yields determined by HPLC (peak retention times compared to product standards, followed by MS analysis for confirmation, and then calculation of yields based on product standard curves). All reactions were standardized to 3.80 μmol of **4a** in 100 μl of solvent.

Table S6. Gold-catalyzed reactivity of compound **4b** for the synthesis of indole **5b**

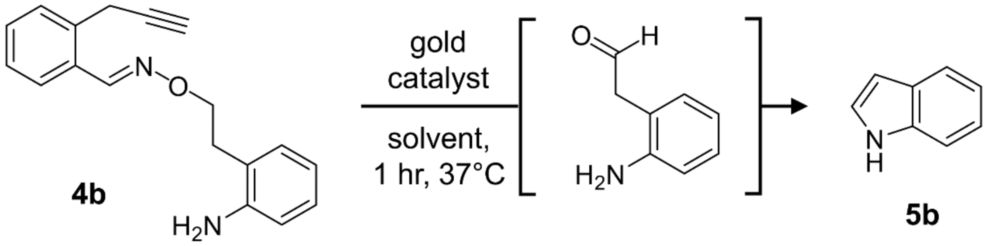

Entry	Catalyst	Mol%	Solvent	Time (hr)	Yield of **5b** (%)[a]	Turnover (TON)[a]
190	-	-	50% THF in PBS buffer	1	0	-
191	**Au1**	5	50% THF in PBS buffer	1	18	3.6
192	**Au1**	5	50% DMSO in PBS buffer	1	25	5.0
193	**Au1**	5	50% ACN in PBS buffer	1	20	4.1
194	**Au3**	5	50% THF in PBS buffer	1	118	3.6
195	**Au3**	5	50% DMSO in PBS buffer	1	20	4.1
196	**Au3**	5	50% ACN in PBS buffer	1	18	3.7

[a]Yields determined by HPLC (peak retention times compared to product standards, followed by MS analysis for confirmation, and then calculation of yields based on product standard curves). All reactions were standardized to 3.56 μmol of **4b** in 100 μl of solvent.

Table S7. Gold-catalyzed reactivity of compound **4c** for the synthesis of indole **5c**

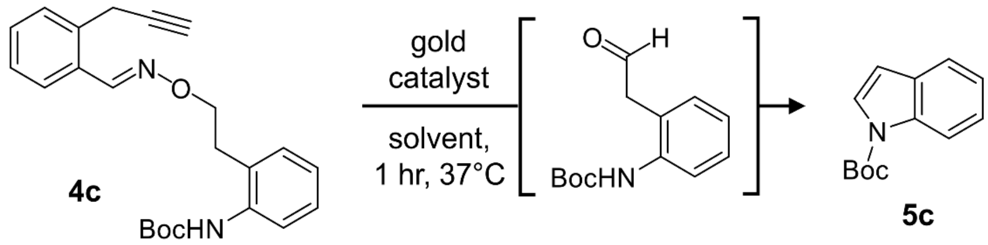

Entry	Catalyst	Mol%	Solvent	Time (hr)	Yield of **5c** (%)[a]	Turnover (TON)[a]
197	**-**	-	50% THF in PBS buffer	1	0	-
198	**Au1**	5	50% THF in PBS buffer	1	28	5.5
199	**Au1**	5	50% DMSO in PBS buffer	1	19	3.7
200	**Au1**	5	50% ACN in PBS buffer	1	28	5.5
201	**Au3**	5	50% THF in PBS buffer	1	28	5.6
202	**Au3**	5	50% DMSO in PBS buffer	1	14	2.8
203	**Au3**	5	50% ACN in PBS buffer	1	22	4.4

[a]Yields determined by HPLC (peak retention times compared to product standards, followed by MS analysis for confirmation, and then calculation of yields based on product standard curves). All reactions were standardized to 2.64 µmol of **4c** in 100 µl of solvent.

Table S8. Gold-catalyzed reactivity of compound **4d** for the synthesis of indole **5d**

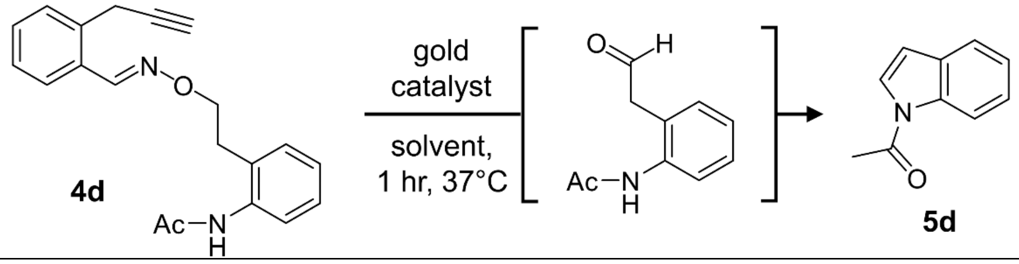

Entry	Catalyst	Mol%	Solvent	Time (hr)	Yield of **5d** (%)[a]	Turnover (TON)[a]
204	**-**	-	50% THF in PBS buffer	1	0	-
205	**Au1**	5	50% THF in PBS buffer	1	26	5.2
206	**Au1**	5	50% DMSO in PBS buffer	1	44	8.8
207	**Au1**	5	50% ACN in PBS buffer	1	28	5.5
208	**Au3**	5	50% THF in PBS buffer	1	62	12.4
209	**Au3**	5	50% DMSO in PBS buffer	1	16	3.2
210	**Au3**	5	50% ACN in PBS buffer	1	22	4.4

[a]Yields determined by HPLC (peak retention times compared to product standards, followed by MS analysis for confirmation, and then calculation of yields based on product standard curves). All reactions were standardized to 3.12 µmol of **4d** in 100 µl of solvent.

Table S9. Gold-catalyzed reactivity of compound **4e** for the synthesis of indole **5e**

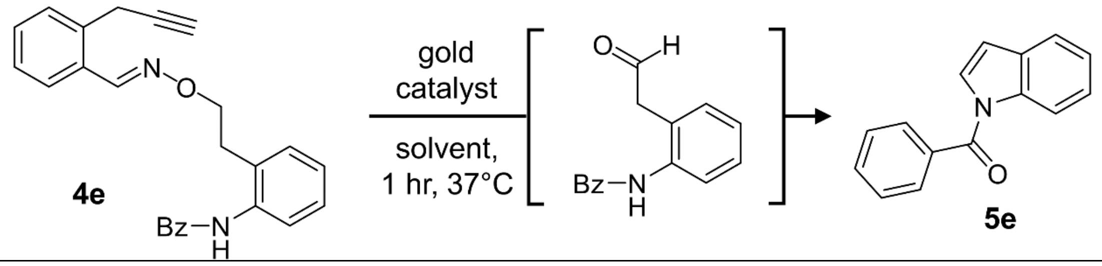

Entry	Catalyst	Mol%	Solvent	Time (hr)	Yield of **5e** (%)[a]	Turnover (TON)[a]
211	-	-	50% THF in PBS buffer	1	0	-
212	**Au1**	5	50% THF in PBS buffer	1	26	5.3
213	**Au1**	5	50% DMSO in PBS buffer	1	29	5.8
214	**Au1**	5	50% ACN in PBS buffer	1	11	2.3
215	**Au3**	5	50% THF in PBS buffer	1	6	1.2
216	**Au3**	5	50% DMSO in PBS buffer	1	17	3.4
217	**Au3**	5	50% ACN in PBS buffer	1	5	1.0

[a]Yields determined by HPLC (peak retention times compared to product standards, followed by MS analysis for confirmation, and then calculation of yields based on product standard curves). All reactions were standardized to 2.61 μmol of **4e** in 100 μl of solvent.

Table S10. Gold-catalyzed reactivity of compound **4f** for the synthesis of indole **5f**

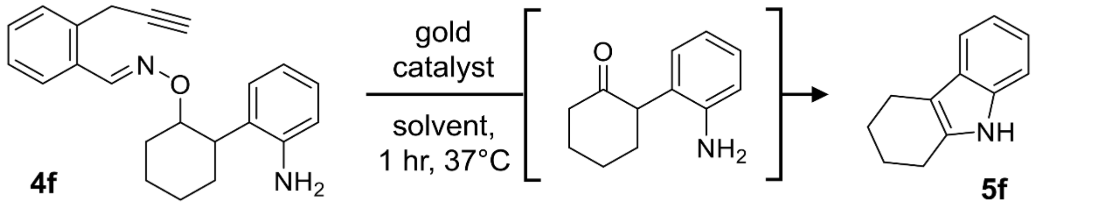

Entry	Catalyst	Mol%	Solvent	Time (hr)	Yield of **5f** (%)[a]	Turnover (TON)[a]
218	-	-	50% THF in PBS buffer	1	0	-
219	**Au1**	5	50% THF in PBS buffer	1	20	3.9
220	**Au1**	5	50% DMSO in PBS buffer	1	20	4.0
221	**Au1**	5	50% ACN in PBS buffer	1	26	5.3
222	**Au3**	5	50% THF in PBS buffer	1	28	5.6
223	**Au3**	5	50% DMSO in PBS buffer	1	22	4.4
224	**Au3**	5	50% ACN in PBS buffer	1	26	5.1

[a]Yields determined by HPLC (peak retention times compared to product standards, followed by MS analysis for confirmation, and then calculation of yields based on product standard curves). All reactions were standardized to 3.01 μmol of **4f** in 100 μl of solvent.

3. Modelling studies

3.1 General information

In the following modeling studies, the three-dimensional X-ray structures of the HaloTag protein (entry 6U32) and αβ-tubulin heterodimer (entry 5LYJ) were taken from the Protein Data Bank. Molecular docking of covalently bound ligands in the HaloTag protein was carried out using AutoDockFR software.[1, 2] With the covalently bound tetramethylrhodamine ligand acting as a reference, the docking space was calculated by AutoGridFR.[3] These calculations gave a grid box of 20.25×22.50×19.50 that is centered at 80.114, 48.737, 8.495 (x,y,z) with a spacing of 0.375 Å. Molecular docking of ligands to the colchicine binding pocket of the αβ-tubulin heterodimer was carried out using Autodock Vina software (version 1.1.2).[4] With the crystallized combretastatin A4 ligand acting as a reference, the docking space was calculated by AutoGridFR.[3] These calculations gave a grid box of 6.25×15.75×12.00 that is centered at 13.469, 10.674, -21.599 (x,y,z) with a spacing of 0.375 Å. All docking was implemented through the graphical user interface AutoDockTools (v1.5.6),[5] which was used to setup the receptors: all polar hydrogens and Kollman charges were added. The Au parameters were set as r = 3.29 Å and the van der Waals well depth at 0.039 kcal/mol. The 3D structures of the **Au8**-**Au11** catalysts, phenstatin **8**, and prodrug **9** were acquired on ChemDraw (v18.1) software, before being cleaned up and saved in PDB format with the aid of the Discovery Studio Visualizer (v17.2.0.16349) program. These molecules were further modified by AutoDockTools to give the corresponding pdbqt files. For the docking runs, default parameters were typically used along with a global search exhaustiveness set to 30. Solvent-accessibility surface area (SASA) was determined for specific atoms of the docked ligands using Discovery Studio Visualizer (v17.2.0.16349).

3.2 HaloTag cavity opening analysis

In the planning stage of this project, analysis of the HaloTag protein (PDB 6U32) revealed that the opening to the cavity (HaloTag ligand binding site) was rather small with an average radius of ~6Å (Figure S13A). To increase the cavity opening, the M175A and F144A mutations were chosen and the relevant protein constructs then synthesized and cloned. With an increased width of 8-10Å (Figure S13B), the cavity opening should be better positioned to accommodate bound Au catalysts used in this study.

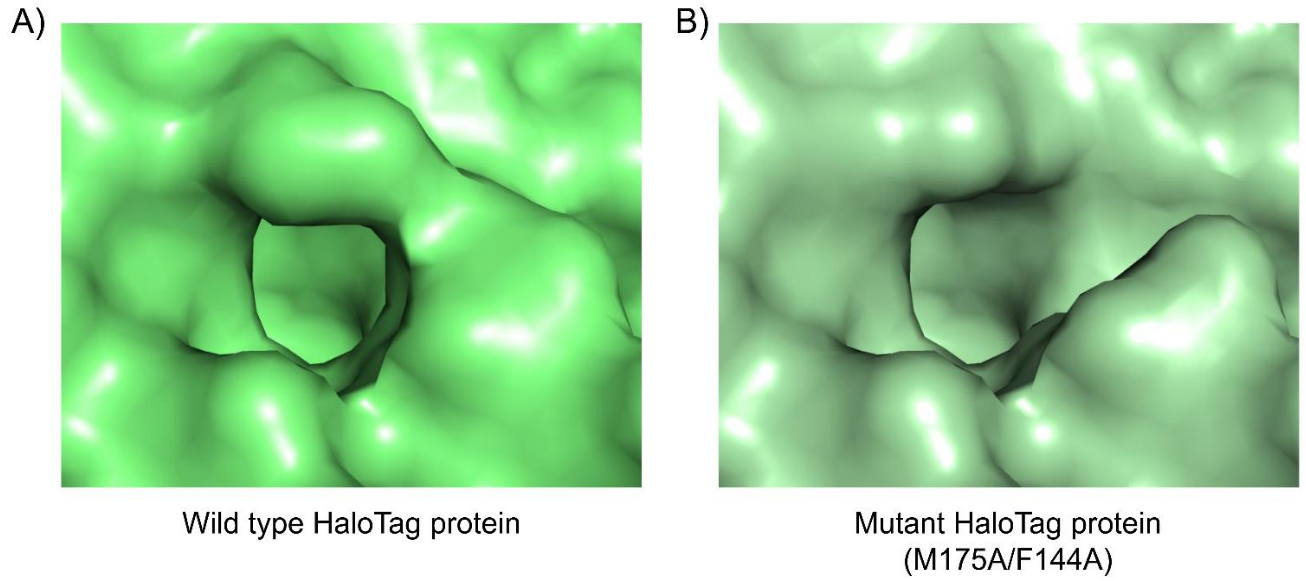

Figure S13. Comparison of the cavity openings between A) wild type HaloTag protein and B) mutant HaloTag M175A/F144A protein.

3.3 Docking of gold catalysts to the HaloTag protein

Once **Au8-Au11** are covalently bound to the HaloTag protein, the aim is to have the gold catalyst moiety positioned near the cavity opening to react with any relevant substrates. To analyze this situation, covalent docking was performed using **Au8-Au11** anchored to Asp106 of the HaloTag protein (PDB 6U32). Following docking, the lengths from the Asp106 anchor point to the gold atom were then measured (Figure S14). From this data, it appears that the shortest length observed is with **Au8** (15.7Å), which is expected given its relatively short 5-carbon linker. Next increasing in length is **Au9** (15.9Å), before both **Au10** and **Au11** reach a similar length of 18.5Å. This suggests that the flexibility of the longer linkers possibly allows the gold moiety to adopt its most favorable orientation.

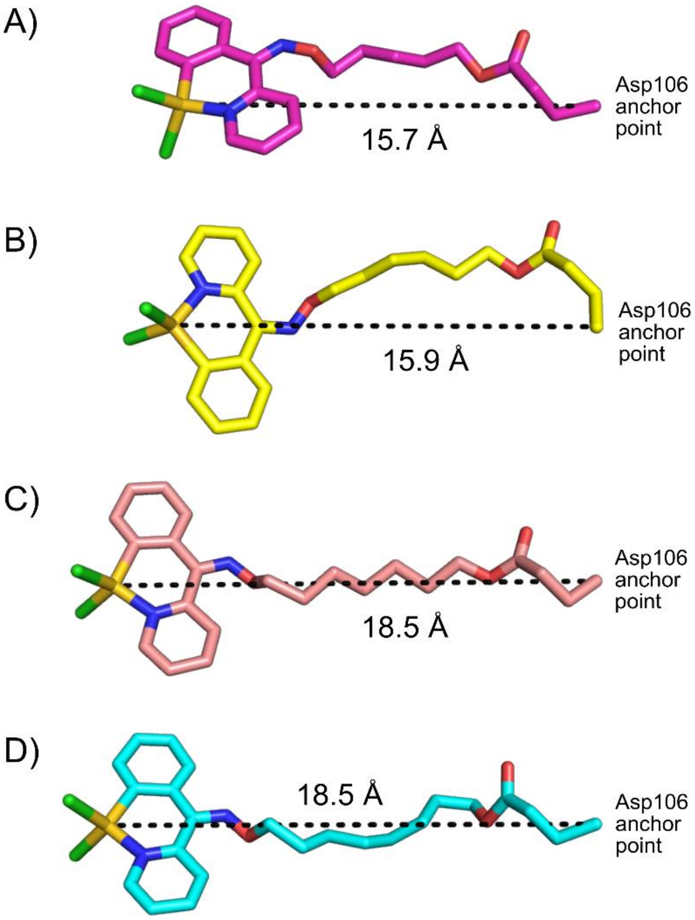

Figure S14. Summary of the lengths from the Asp106 anchor point to the gold atom for catalysts A) **Au8** (dark pink), B) **Au9** (yellow), C) **Au10** (light pink), and D) **Au11** (turquoise).

3.4 SASA analysis of bound gold catalysts

To analyze the substrate accessibility of the embedded gold catalyst, solvent accessible surface area (SASA) values were calculated for the gold atom (and its bonded carbon and nitrogen atom) in each of the **Au8-Au11** docked poses (Table S11). Calculated SASA values reveal that **Au8** has the least solvent accessible gold, while **Au10** has the most solvent accessible gold. This trend correlates well with regards to the carbon linker length, as the short linker in **Au8** likely pulls the gold atom deeper into the substrate pocket, limiting its solvent accessibility.

Table S11. Calculated SASA of specific atoms of the docked catalyst **Au8** to **Au11**

		Catalyst **Au8** (linker = 5 carbons)	Catalyst **Au9** (linker = 6 carbons)	Catalyst **Au10** (linker = 7 carbons)	Catalyst **Au11** (linker = 8 carbons)
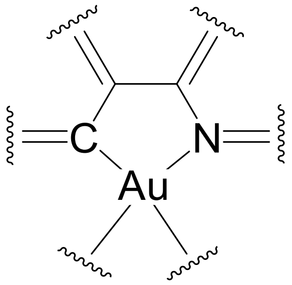	C	0	0	0.503	0
	N	1.461	0	0	0
	Au	16.102	21.133	26.165	24.656

3.5 Docking studies to predict tubulin binding properties of prodrug

In this study, prodrug **10** was designed as a non-toxic analogue incapable of functioning as a tubulin inhibitor. To theoretically predict binding properties prior to synthesis, docking studies were carried out (Figure S15-S17). First, as a control to validate the docking methodology, colchicine was modelled with αβ-tubulin and compared to its known crystallized binding mode. As depicted in Figure S15, the docked and crystallized colchichine (from literature) clearly show similar binding configurations. Moving on, prodrug **10** was then modelled with αβ-tubulin, giving a calculated binding affinity of -4.880 kcal/mol (Figure S16). This value was determined to be weaker than the calculated binding of -8.071 kcal/mol obtained for Phenstatin **9** (Figure S17). Thus, modelling studies predict that prodrug **10** likely possesses lower cytotoxicity compared to its activated drug molecule due to poorer αβ-tubulin binding.

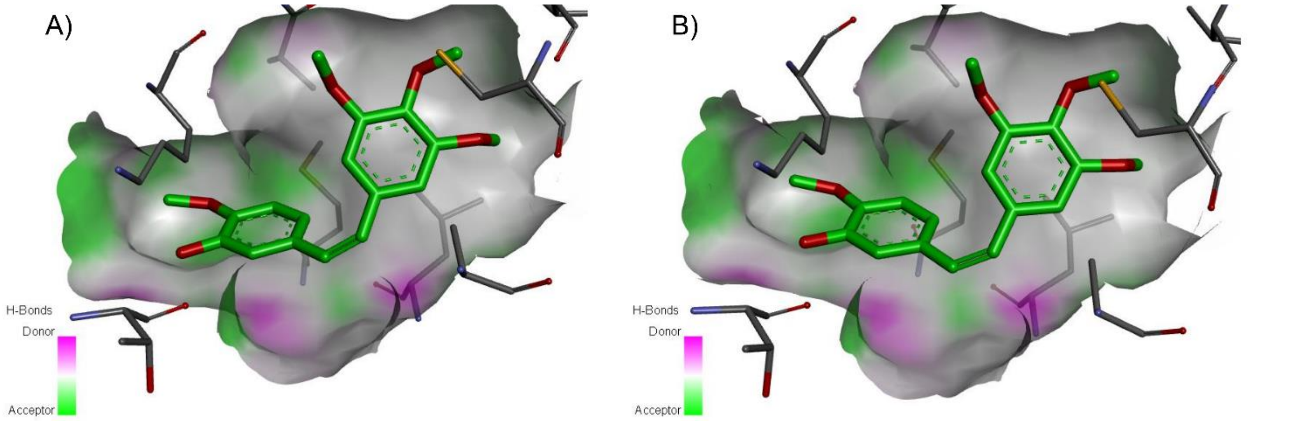

Figure S15. A) Modeled docking of combretastatin A4 (green) in the colchicine binding site of the αβ-tubulin heterodimers (PDB 5LYJ), which returned a calculated binding affinity of -7.487 kcal/mol. B) As a reference binding pose, crystallized combretastatin A4 with the αβ-tubulin heterodimer (PDB 5LYJ) is also shown. The view of the surface and residues around the docked molecule in the binding pocket is shown, with hydrophobic surfaces colored in grey and hydrogen bonding surfaces colored in purple/light green.

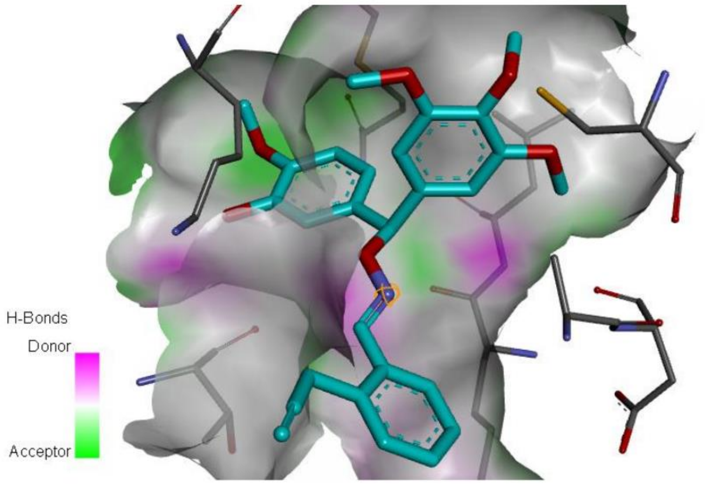

Figure S16. A) Modeled docking of prodrug **10** (cyan) in the colchicine binding site of the αβ-tubulin heterodimers (PDB 5LYJ), which returned a calculated binding affinity of -4.880 kcal/mol. The view of the surface and residues around the docked molecule in the binding pocket is shown, with hydrophobic surfaces colored in grey and hydrogen bonding surfaces colored in purple/light green.

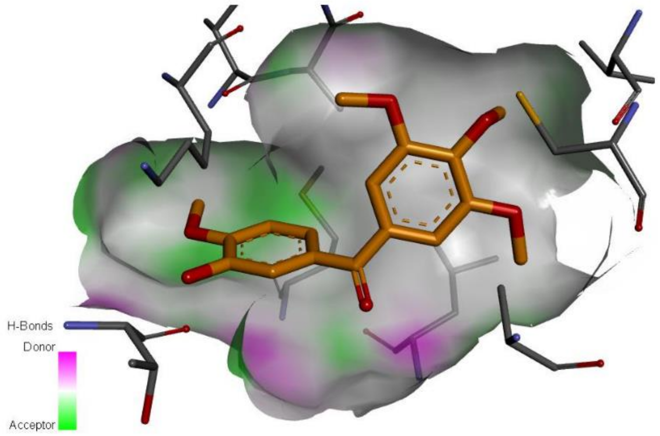

Figure S17. A) Modeled docking of Phenstatin **9** (orange) in the colchicine binding site of the αβ-tubulin heterodimers (PDB 5LYJ), which returned a calculated binding affinity of -8.071 kcal/mol. The view of the surface and residues around the docked molecule in the binding pocket is shown, with hydrophobic surfaces colored in grey and hydrogen bonding surfaces colored in purple/light green.

4. Protein preparations and experiments

4.1 General information

General reagents and buffer components were purchased from Sigma-Aldrich, Scharlau, or Oxoid without further purification. Specialized chemicals for this study include Ellman's reagent (Sigma), DBCO-FITC (Lumiprobe), and Azido-PEG3-C6-Cl (MedChemExpress). Restriction enzymes used in this study were acquired from New England Biolabs. For plasmid transformations, One Shot™ BL21(DE3) Chemically Competent *E. coli* were acquired from ThermoFisher. For protein expression and purification, items such as Pierce protease inhibitor tablets, HisPur Ni-NTA Cartridges, 10K MWCO protein concentrators, and the Pierce Bradford Protein Assay Kit were all acquired from ThermoFisher.

4.2 Recombinant protein expression and purification

Genes encoding fusion proteins were synthesized by Genscript and inserted into pET-21a(+) vectors between differing combinations of NdeI, KpnI, XhoI, and BamHI cut sites. All protein sequences are shown in Figure S18. For expression, plasmids were transformed into BL21(DE3) *E. coli* and then incubated on Luria-Bertani (LB) Agar plates with ampicillin (50 µg/mL) overnight at 37 °C. Isolated colonies were picked and cultured in 7 mL LB broth with ampicillin (50 µg/mL) overnight in shaking incubators at 37 °C. These overnight cultures were then used to inoculate larger LB cultures (500 mL), which were grown in shaking incubators at 37 °C until an O.D. reading (at 600 nm) of 0.6 was reached. To induce protein expression, cultures were supplemented with 0.5 mM isopropyl β-D-1-thiogalactopyranoside (IPTG) and then grown for an additional 4 hours at 28 °C. Bacterial pellets were obtained through centrifugation (7,350 rpm at 4 °C for 10 min) and then resuspended in lysis buffer (20 mM Tris, 300 mM NaCl, 1 mM PMSF, pH 7.4) supplemented with a protease inhibitor tablet. Sonication was performed (10s on/15s off for 15 min), followed by centrifugation (12,000 rpm at 4 °C for 20 min) to isolate the supernatant. To perform protein purification, the the supernatant was loaded onto a 5 mL Ni-NTA column connected to an ÄKTA start FPLC system (Cytiva). At least 10 column volumes of an equilibration buffer (20 mM Tris, 300 mM NaCl, pH 7.4) was used to wash the column. An imidazole gradient (0–300 mM) was then applied to the column by mixing the equilibration buffer with an elution buffer (20 mM Tris, 300 mM NaCl, 300 mM imidazole, pH 7.4). Eluted protein fractions were analyzed by SDS-PAGE, with appropriate fractions then collected and combined. Following volume reduction using 30K MWCO concentrators, buffer exchange using PBS buffer was done to remove any remaining eluting agents. Final protein concentrations were determined via a Bradford protein assay.

HtPA

```
MGHHHHHHHHHHSSGHIDDDDKHMTTSAVNIYNISAGASVDLAAPVTTGDIVTFFSSALNLSAGAGSPNNTALNLLSENGAYLLHIAFRLQENVIVFNSRQPNAPWLVEQRVSN
VANQFIGSGGKAMVTVFDHGDKYQVVINEKTVIQYTKQISGTTSSLSYNSTEGTSIFSTVVEAVTYTGLAGSSGGSGGSGSGSGGTERQPTTDRMIQEYVPGKQVTLAHLIANP
GKDLFKKLGLQDAVSAIGILTITPSEASIIACDIATKSGAVEIGFLDRFTGAVVLTGDVSAVEYALKQVTRTLGEMMQFTTCSITRTLEGSANGFVGGSMAEIGTGFPFDPHYV
EVLGERMHYVDVGPRDGTPVLFLHGNPTSSYVWRNIIPHVAPTHRCIAPDLIGMGKSDKPDLGYFFDDHVRFMDAFIEALGLEEVVLVIHDWGSALGFHWAKRNPERVKGIAFM
EFIRPIPTWDEWPEAARETFQAFRTTDVGRKLIIDQNVFIEGTLPAGVVRPLTEVEMDHYREPFLNPVDREPLWRFPNELPIAGEPANIVALVEEYMDWLHQSPVPKLLFWGTP
GVLIPPAEAARLAKSLPNCKAVDIGPGLNLLQEDNPDLIGSEIARWLSTLEISGGSGC
```

HtA

```
MGHHHHHHHHHHSSGHIDDDDKHMTTSAVNIYNISAGASVDLAAPVTTGDIVTFFSSALNLSAGAGSPNNTALNLLSENGAYLLHIAFRLQENVIVFNSRQPNAPWLVEQRVSN
VANQFIGSGGKAMVTVFDHGDKYQVVINEKTVIQYTKQISGTTSSLSYNSTEGTSIFSTVVEAVTYTGLAGSSGGSGGSGSGSGGTSGAVLEGSANGFVGGSMAEIGTGFPFDP
HYVEVLGERMHYVDVGPRDGTPVLFLHGNPTSSYVWRNIIPHVAPTHRCIAPDLIGMGKSDKPDLGYFFDDHVRFMDAFIEALGLEEVVLVIHDWGSALGFHWAKRNPERVKGI
AFMEFIRPIPTWDEWPEAARETFQAFRTTDVGRKLIIDQNVFIEGTLPAGVVRPLTEVEMDHYREPFLNPVDREPLWRFPNELPIAGEPANIVALVEEYMDWLHQSPVPKLLFW
GTPGVLIPPAEAARLAKSLPNCKAVDIGPGLNLLQEDNPDLIGSEIARWLSTLEISGGSGC
```

Ht

```
MGHHHHHHHHHHSSGHIDDDDKHMLEGSANGFVGGSMAEIGTGFPFDPHYVEVLGERMHYVDVGPRDGTPVLFLHGNPTSSYVWRNIIPHVAPTHRCIAPDLIGMGKSDKPDLG
YFFDDHVRFMDAFIEALGLEEVVLVIHDWGSALGFHWAKRNPERVKGIAFMEFIRPIPTWDEWPEAARETFQAFRTTDVGRKLIIDQNVFIEGTLPAGVVRPLTEVEMDHYREP
FLNPVDREPLWRFPNELPIAGEPANIVALVEEYMDWLHQSPVPKLLFWGTPGVLIPPAEAARLAKSLPNCKAVDIGPGLNLLQEDNPDLIGSEIARWLSTLEISGGSGC
```

ACG Lectin | PduU aggregating unit | HaloTag

Figure S18. Sequences of protein complexes designed and created for this study

4.3 Analysis of protein complexation

As depicted in Figure S19, size exclusion chromatography was carried out to confirm the self-assembly of the hexameric state of **HtPA**.

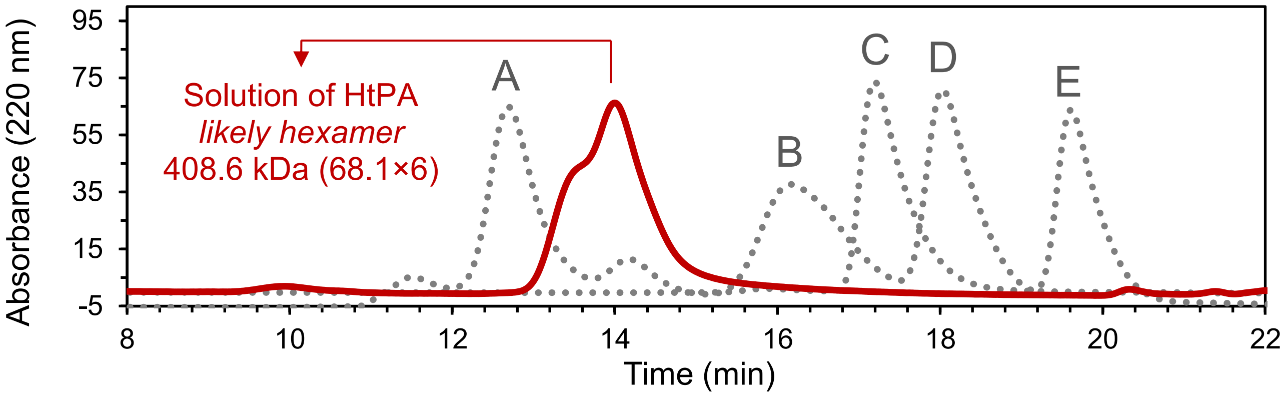

Figure S19. Overlayed size-exclusion chromatogram run with a solution of **HtPA** (red) and protein standards of varying sizes (grey; A=670 kDa, B=158 kDa, C=66 kDa, D=44 kDa, E=17 kDa).

4.4 Artificial metalloenzyme preparation

Scheme S7 depicts the general steps taken to produce any of the ArMs used in this study (**Ht-Au8**, **Ht-Au9**, **Ht-Au10**, **Ht-Au11**, **HtPA-Au8**). Since the HaloTag protein naturally possess two free cysteine residues (Cys61 and Cys262), these first need to be blocked using *N*-ethylmaleimide (NEM). To do this, purified Halotag-containing proteins (83 nmol, 500 µl from a 165 µM stock solution in PBS buffer) were mixed with NEM (30 µmol, 200 µl from a 148 mM stock solution in H_2O). The solution was then mildly mixed and incubated at room temp for 2 hr. To confirm cysteine blockage, a small sample of the solution was removed (1 µl) and mixed with Ellman's reagent (9 mM, 9 µl from a 10 mM stock solution in PBS buffer). Following a short 1 min incubation, the absorbance at 412 nm was measured. Under these conditions, a significant signal reduction was observed compared to the signal produced by unprotected HaloTag (data not shown). After buffer exchange using 10K MWCO protein concentrators to remove excess NEM, the protein solution was diluted to create a 100 µM stock solution. In the next step, the NEM-protected HaloTag-containing proteins (10 nmol, 100 µl from a 100 µM stock solution in PBS buffer) was then mixed with 260 µl of PBS buffer and either **Au8**, **Au9**, **Au10**, or **Au11** (20 nmol, 40 µl from a 500 µM stock solution in DMSO). The solution was then mildly mixed and incubated at room temp for 2 hr. Afterwards, buffer exchange/volume reduction was carried using 10K MWCO protein concentrators to obtain the desired ArM concentrations.

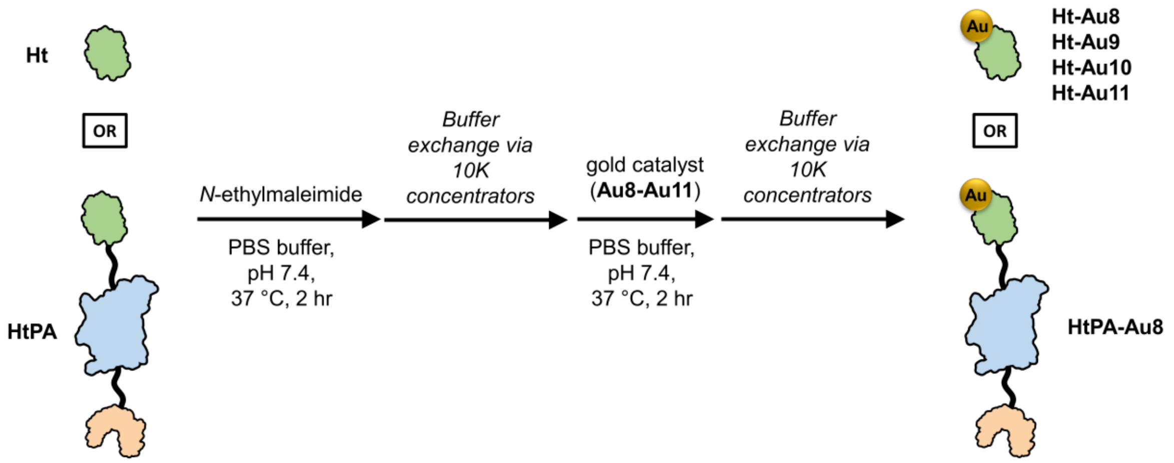

Scheme S7 Preparation of Halotag-based ArMs (**Ht-Au8**, **Ht-Au9**, **Ht-Au10**, **Ht-Au11**, **HtPA-Au8**) used in this study.

4.5 Artificial metalloenzyme characterization

To determine the Au content in the prepared artificial metalloenzymes, 200 µl of various **HtPA** ArMs (100 µM) were submitted to the Health, Safety, and Environment Office (HSEO) laboratory services of HKUST. Samples were first digested with nitric acid, before analysis on an Inductively Coupled Plasma Mass Spectrometer (ICP-MS) Agilent 7900.

4.6 Reactivity studies using artificial metalloenzymes

The biocompatibility of PBO chemistry was investigated using varying HaloTag ArMs (**Ht-Au8**, **Ht-Au9**, **Ht-Au10**, **Ht-Au11**) with substrates **1a**, **1b**, and **4b**. To initiate the reaction, 10 µl of each substrate (20 mM stock solution in DMSO) was mixed with 10 µl of each ArM (500 µM stock solution in PBS buffer) and 80 µl of relevant media. These mixtures were then incubated for 4 hours in a sand bath at an adjusted temperature of 37°C without stirring. To workup, mixtures were quenched with 1 mM dodecanethiol in ACN (100 µl) and 1.0M HCl (50 µl). The solutions were then filtered and injected (100 µl) onto a HPLC with an autosampler using various HPLC methods (details in Figure S2, S3, and S5). Data regarding HaloTag ArMs reactivities for substrates **1a**, **1b**, and **4b** are shown in Tables S12-S14.

Table S12. Artificial metalloenzyme catalyzed conversion of **1a** for the release of aldehyde **3a**

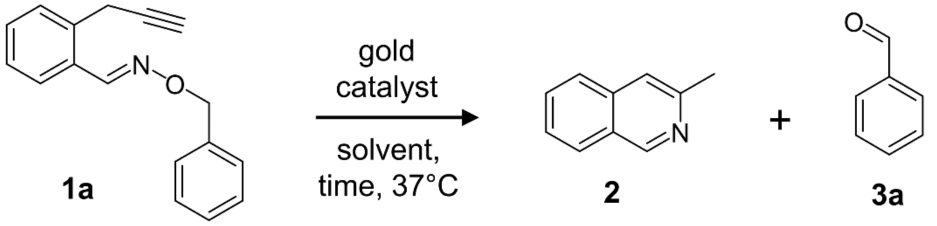

Entry	Catalyst	Mol%	Solvent	Yield of **3a** (%)[a]	Turnover (TON)[a]
225	**Ht-Au8**	2.5	1:9 DMSO/PBS buffer	5.5 ± 1.0	2.70 ± 0.19
226	**Ht-Au8**	2.5	1:1:8 DMSO/serum/PBS buffer	5.9 ± 2.0	3.23 ± 0.23
227	**Ht-Au8**	2.5	1:1:8 DMSO/lysate/PBS buffer	5.5 ± 0.3	2.38 ± 0.28
228	**Ht-Au9**	2.5	1:9 DMSO/PBS buffer	5.6 ± 0.3	2.57 ± 0.18
229	**Ht-Au9**	2.5	1:1:8 DMSO/serum/PBS buffer	5.8 ± 1.1	2.96 ± 0.19
230	**Ht-Au9**	2.5	1:1:8 DMSO/lysate/PBS buffer	5.3 ± 0.4	2.37 ± 0.10
231	**Ht-Au10**	2.5	1:9 DMSO/PBS buffer	4.8 ± 0.7	1.66 ± 0.07
232	**Ht-Au10**	2.5	1:1:8 DMSO/serum/PBS buffer	4.7 ± 0.3	1.82 ± 0.10
233	**Ht-Au10**	2.5	1:1:8 DMSO/lysate/PBS buffer	4.7 ± 0.9	1.51 ± 0.07
234	**Ht-Au11**	2.5	1:9 DMSO/PBS buffer	4.4 ± 0.8	1.33 ± 0.05
235	**Ht-Au11**	2.5	1:1:8 DMSO/serum/PBS buffer	4.5 ± 1.3	1.31 ± 0.18
236	**Ht-Au11**	2.5	1:1:8 DMSO/lysate/PBS buffer	4.7 ± 1.2	1.40 ± 0.10
237	-	-	1:9 DMSO/PBS buffer	0	-
238	-	-	1:1:8 DMSO/serum/PBS buffer	0	-
239	-	-	1:1:8 DMSO/lysate/PBS buffer	0	-
240	**Au8**	2.5	1:9 DMSO/PBS buffer	46.1 ± 1.9	18.44 ± 0.75
241	**Au8**	2.5	1:1:8 DMSO/serum/PBS buffer	15.2 ± 1.5	6.08 ± 0.60
242	**Au9**	2.5	1:9 DMSO/PBS buffer	39.0 ± 7.7	15.60 ± 3.06
243	**Au9**	2.5	1:1:8 DMSO/serum/PBS buffer	14.3 ± 1.6	5.74 ± 0.63
244	**Au10**	2.5	1:9 DMSO/PBS buffer	52.0 ± 2.4	20.78 ± 0.96
245	**Au10**	2.5	1:1:8 DMSO/serum/PBS buffer	12.4 ± 1.7	4.95 ± 0.69
246	**Au11**	2.5	1:9 DMSO/PBS buffer	51.1 ± 9.9	20.46 ± 3.98
247	**Au11**	2.5	1:1:8 DMSO/serum/PBS buffer	10.7 ± 0.7	4.26 ± 0.27

[a]Yields determined by HPLC (peak retention times compared to product standards, followed by MS analysis for confirmation, and then calculation of yields based on product standard curves). All reactions were standardized to 0.2 μmoles of **1a** and 5 nmoles of catalyst in 100 μl of solvent.

Table S13. Artificial metalloenzyme catalyzed conversion of **1b** for the release of ketone **3b**

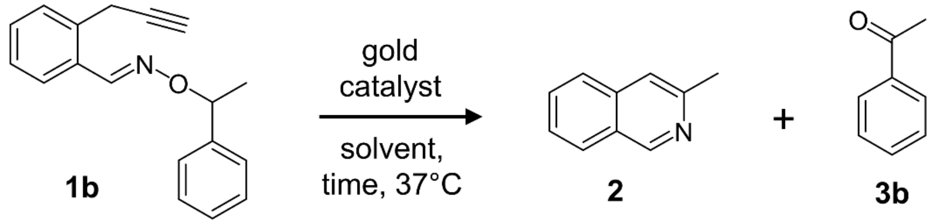

Entry	Catalyst	Mol%	Solvent	Yield of **3b** (%)[a]	Turnover (TON)[a]
248	**Ht-Au8**	2.5	1:9 DMSO/PBS buffer	4.1 ± 1.3	1.04 ± 0.16
249	**Ht-Au8**	2.5	1:1:8 DMSO/serum/PBS buffer	4.1 ± 1.7	0.90 ± 0.01
250	**Ht-Au8**	2.5	1:1:8 DMSO/lysate/PBS buffer	4.2 ± 2.1	0.79 ± 0.07
251	**Ht-Au9**	2.5	1:9 DMSO/PBS buffer	3.7 ± 1.6	0.70 ± 0.09
252	**Ht-Au9**	2.5	1:1:8 DMSO/serum/PBS buffer	3.9 ± 1.9	0.71 ± 0.05
253	**Ht-Au9**	2.5	1:1:8 DMSO/lysate/PBS buffer	3.9 ± 1.8	0.80 ± 0.13
254	**Ht-Au10**	2.5	1:9 DMSO/PBS buffer	4.1 ± 1.5	0.98 ± 0.12
255	**Ht-Au10**	2.5	1:1:8 DMSO/serum/PBS buffer	4.6 ± 2.8	1.25 ± 0.32
256	**Ht-Au10**	2.5	1:1:8 DMSO/lysate/PBS buffer	4.5 ± 1.9	0.99 ± 0.11
257	**Ht-Au11**	2.5	1:9 DMSO/PBS buffer	4.4 ± 1.8	1.00 ± 0.07
258	**Ht-Au11**	2.5	1:1:8 DMSO/serum/PBS buffer	4.5 ± 1.9	1.06 ± 0.11
259	**Ht-Au11**	2.5	1:1:8 DMSO/lysate/PBS buffer	4.1 ± 1.7	0.94 ± 0.08
260	-	-	1:9 DMSO/PBS buffer	0	-
261	-	-	1:1:8 DMSO/serum/PBS buffer	0	-
262	-	-	1:1:8 DMSO/lysate/PBS buffer	0	-
263	**Au8**	2.5	1:9 DMSO/PBS buffer	16.3 ± 1.7	6.53 ± 0.66
264	**Au8**	2.5	1:1:8 DMSO/serum/PBS buffer	7.0 ± 0.4	2.79 ± 0.16
265	**Au9**	2.5	1:9 DMSO/PBS buffer	14.7 ± 0.5	5.87 ± 0.21
266	**Au9**	2.5	1:1:8 DMSO/serum/PBS buffer	8.5 ± 0.7	3.40 ± 0.27
267	**Au10**	2.5	1:9 DMSO/PBS buffer	9.9 ± 2.1	3.97 ± 0.84
268	**Au10**	2.5	1:1:8 DMSO/serum/PBS buffer	5.3 ± 0.2	2.12 ± 0.07
269	**Au11**	2.5	1:9 DMSO/PBS buffer	13.8 ± 0.4	5.52 ± 0.15
270	**Au11**	2.5	1:1:8 DMSO/serum/PBS buffer	4.9 ± 0.7	1.98 ± 0.27

[a]Yields determined by HPLC (peak retention times compared to product standards, followed by MS analysis for confirmation, and then calculation of yields based on product standard curves). All reactions were standardized to 0.2 μmoles of **1b** and 5 nmoles of catalyst in 100 μl of solvent.

Table S14. Artificial metalloenzyme catalyzed conversion of **4b** for the synthesis of indole **5b**

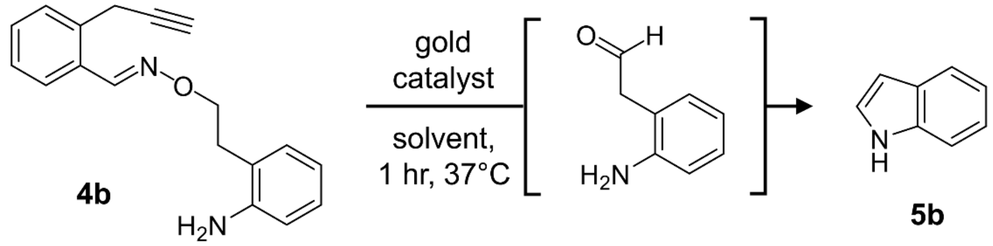

Entry	Catalyst	Mol%	Solvent	Yield of **5b** (%)[a]	Turnover (TON)[a]
271	**Ht-Au8**	2.5	1:9 DMSO/PBS buffer	5.3 ± 1.3	2.27 ± 0.29
272	**Ht-Au8**	2.5	1:1:8 DMSO/serum/PBS buffer	5.2 ± 0.7	2.22 ± 0.19
273	**Ht-Au8**	2.5	1:1:8 DMSO/lysate/PBS buffer	5.3 ± 1.4	2.55 ± 0.35
274	**Ht-Au9**	2.5	1:9 DMSO/PBS buffer	5.0 ± 2.3	1.32 ± 0.29
275	**Ht-Au9**	2.5	1:1:8 DMSO/serum/PBS buffer	4.8 ± 2.0	1.38 ± 0.22
276	**Ht-Au9**	2.5	1:1:8 DMSO/lysate/PBS buffer	4.8 ± 1.6	1.36 ± 0.20
277	**Ht-Au10**	2.5	1:9 DMSO/PBS buffer	4.5 ± 1.3	1.34 ± 0.11
278	**Ht-Au10**	2.5	1:1:8 DMSO/serum/PBS buffer	4.8 ± 1.9	1.29 ± 0.22
279	**Ht-Au10**	2.5	1:1:8 DMSO/lysate/PBS buffer	4.9 ± 1.4	1.54 ± 0.19
280	**Ht-Au11**	2.5	1:9 DMSO/PBS buffer	3.7 ± 2.4	0.60 ± 0.22
281	**Ht-Au11**	2.5	1:1:8 DMSO/serum/PBS buffer	4.9 ± 5.2	0.52 ± 0.21
282	**Ht-Au11**	2.5	1:1:8 DMSO/lysate/PBS buffer	4.2 ± 2.5	0.68 ± 0.10
283	-	-	1:9 DMSO/PBS buffer	0	-
284	-	-	1:1:8 DMSO/serum/PBS buffer	0	-
285	-	-	1:1:8 DMSO/lysate/PBS buffer	0	-
286	**Au8**	2.5	1:9 DMSO/PBS buffer	16.3 ± 2.8	6.51 ± 1.11
287	**Au8**	2.5	1:1:8 DMSO/serum/PBS buffer	8.5 ± 0.2	3.41 ± 0.09
288	**Au9**	2.5	1:9 DMSO/PBS buffer	14.8 ± 1.1	5.92 ± 0.46
289	**Au9**	2.5	1:1:8 DMSO/serum/PBS buffer	11.8 ± 0.7	4.72 ± 0.29
290	**Au10**	2.5	1:9 DMSO/PBS buffer	13.0 ± 1.0	5.20 ± 0.42
291	**Au10**	2.5	1:1:8 DMSO/serum/PBS buffer	8.4 ± 0.3	3.36 ± 0.12
292	**Au11**	2.5	1:9 DMSO/PBS buffer	13.4 ± 2.8	5.38 ± 1.13
293	**Au11**	2.5	1:1:8 DMSO/serum/PBS buffer	7.3 ± 0.3	2.93 ± 0.11

[a]Yields determined by HPLC (peak retention times compared to product standards, followed by MS analysis for confirmation, and then calculation of yields based on product standard curves). All reactions were standardized to 0.2 μmoles of **4b** and 5 nmoles of catalyst in 100 μl of solvent.

4.7 Kinetic studies using artificial metalloenzymes

Michaelis-Menten kinetics were determined for substrates **1a**, **1b**, **4b**, and **7** using varying HaloTag ArMs (**Ht-Au8**, **Ht-Au9**, **Ht-Au10**, **Ht-Au11**). Reactivity was performed using varying concentrations of substrate with a set concentration of ArM. A table of calculated values are summarized in Table S15, which includes the maximum velocity (V_{max}), substrate affinity (K_M), turnover frequency (k_{cat}), catalytic efficiency (k_{cat}/K_M), and the coefficient of determination (R^2). Individual plots are shown in Figure S20-S23.

Table S15. Michaelis-Menten kinetics for obtained for substrates **1a**, **1b**, **4b**, and **7** using varying ArMs

	V_{max} (μM/s)	K_M (mM)	k_{cat} (s^{-1})	k_{cat}/K_M (M^{-1} s^{-1})
Substrate **1a**				
**Ht-Au8**	0.0247 ± 0.0019	2.2289 ± 0.5184	0.00099 ± 0.00007	0.44 ± 0.11
**Ht-Au9**	0.0131 ± 0.0009	7.1547 ± 0.8075	0.00052 ± 0.00003	0.07 ± 0.01
**Ht-Au10**	0.0149 ± 0.0071	5.3575 ± 4.6557	0.00059 ± 0.00028	0.11 ± 0.11
**Ht-Au11**	0.0115 ± 0.0030	5.4191 ± 2.5933	0.00046 ± 0.00012	0.09 ± 0.05
Substrate **1b**				
**Ht-Au8**	0.0091 ± 0.0033	5.3123 ± 3.5320	0.00036 ± 0.00013	0.07 ± 0.05
**Ht-Au9**	0.0044 ± 0.0008	7.1303 ± 2.1282	0.00017 ± 0.00003	0.02 ± 0.01
**Ht-Au10**	0.0105 ± 0.0023	6.0420 ± 2.2685	0.00042 ± 0.00009	0.07 ± 0.03
**Ht-Au11**	0.0187 ± 0.0048	10.3167 ± 3.7490	0.00075 ± 0.00019	0.07 ± 0.03
Substrate **4b**				
**Ht-Au8**	0.1172 ± 0.0289	8.1254 ± 3.0943	0.00469 ± 0.00116	0.58 ± 0.26
**Ht-Au9**	0.0536 ± 0.0114	3.8151 ± 1.7840	0.00214 ± 0.00046	0.56 ± 0.29
**Ht-Au10**	0.1100 ± 0.0200	6.4566 ± 1.9574	0.00217 ± 0.00045	0.35 ± 0.08
**Ht-Au11**	0.0261 ± 0.0016	1.7724 ± 0.3874	0.00106 ± 0.00013	0.59 ± 0.13
Substrate **7**				
**Ht-Au8**	0.0037 ± 0.0010	0.0802 ± 0.0549	0.00007 ± 0.00002	1.26 ± 0.72
**Ht-Au9**	0.0059 ± 0.0047	0.1183 ± 0.0631	0.00012 ± 0.00009	0.92 ± 0.28
**Ht-Au10**	0.0086 ± 0.0132	0.2171 ± 0.5542	0.00017 ± 0.00026	0.43 ± 0.21
**Ht-Au11**	0.0087 ± 0.0051	0.2541 ± 0.1703	0.00017 ± 0.00010	0.77 ± 0.20
initial velocity values were obtained in triplicate				

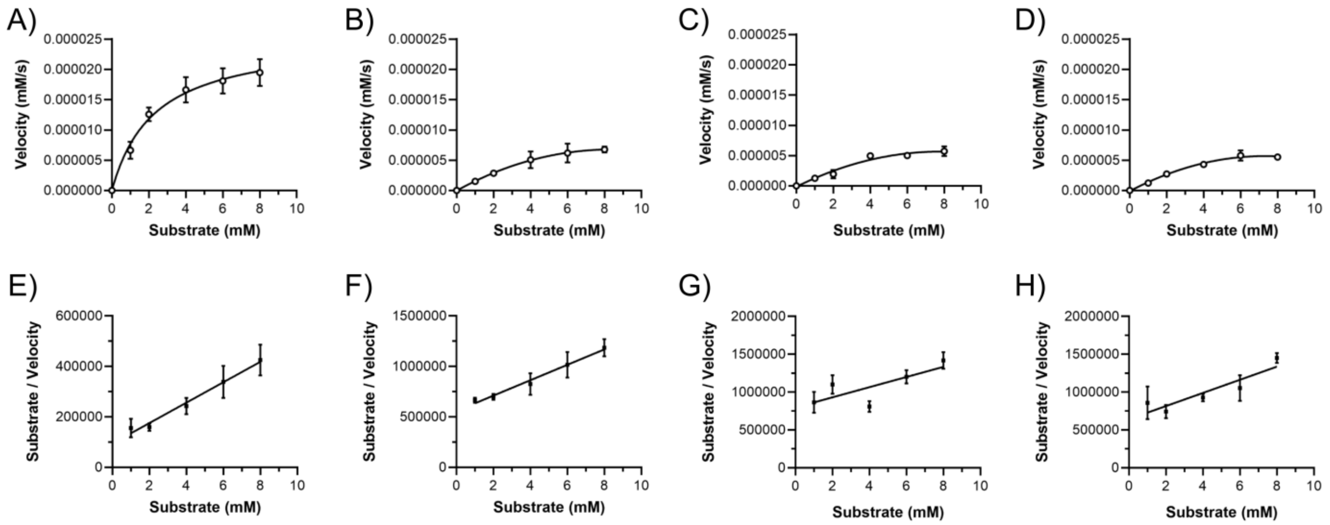

Figure S20. Michaelis-Menten plots for substrate **1a** using A) **Ht-Au8**, B) **Ht-Au9**, C) **Ht-Au10**, D) **Ht-Au11**. Hanes-Woolf plot for substrate **1a** using E) **Ht-Au8**, F) **Ht-Au9**, G) **Ht-Au10**, H) **Ht-Au11**. The concentration of each ArM used to acquire this kinetic data was set to 25 µM.

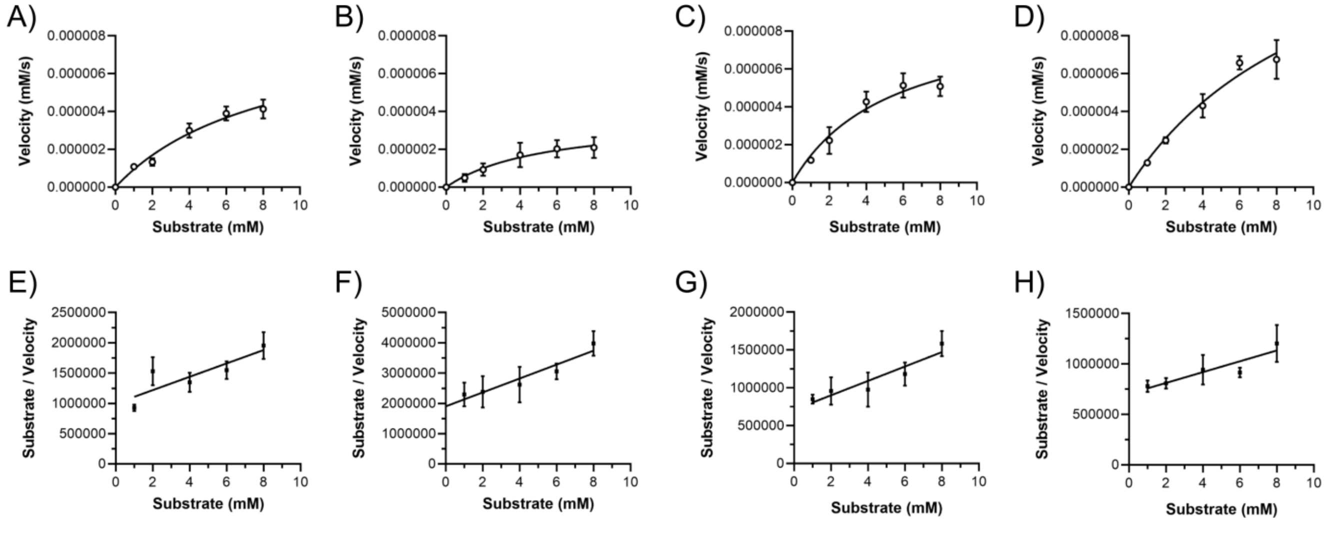

Figure S21. Michaelis-Menten plots for substrate **1b** using A) **Ht-Au8**, B) **Ht-Au9**, C) **Ht-Au10**, D) **Ht-Au11**. Hanes-Woolf plot for substrate **1b** using E) **Ht-Au8**, F) **Ht-Au9**, G) **Ht-Au10**, H) **Ht-Au11**. The concentration of each ArM used to acquire this kinetic data was set to 25 µM.

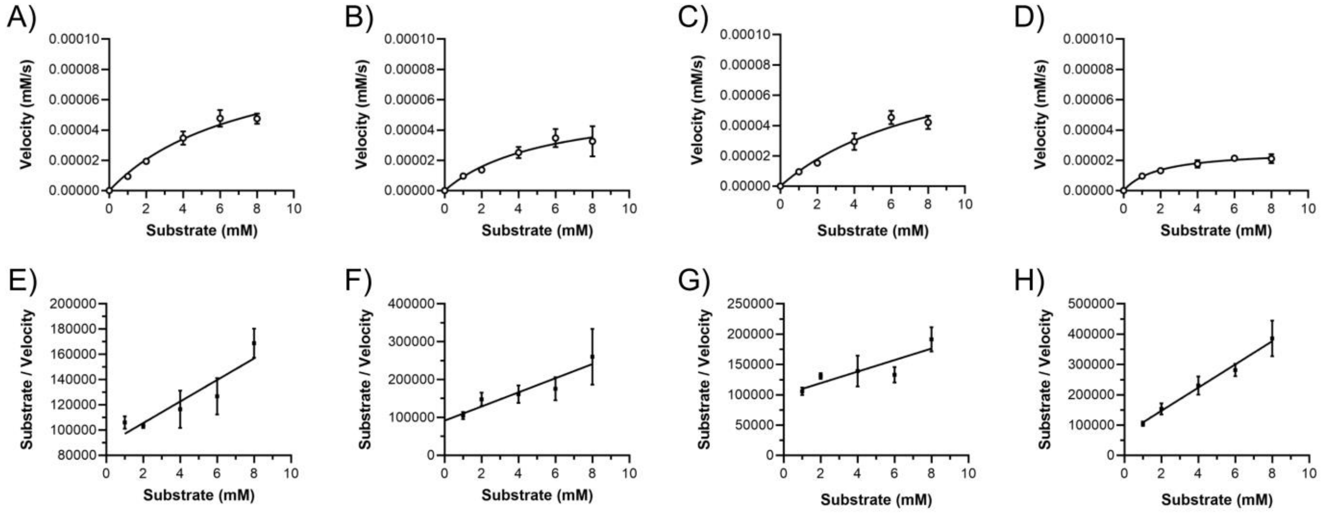

Figure S22. Michaelis-Menten plots for substrate **4b** using A) **Ht-Au8**, B) **Ht-Au9**, C) **Ht-Au10**, D) **Ht-Au11**. Hanes-Woolf plot for substrate **4b** using E) **Ht-Au8**, F) **Ht-Au9**, G) **Ht-Au10**, H) **Ht-Au11**. The concentration of each ArM used to acquire this kinetic data was set to 25 µM.

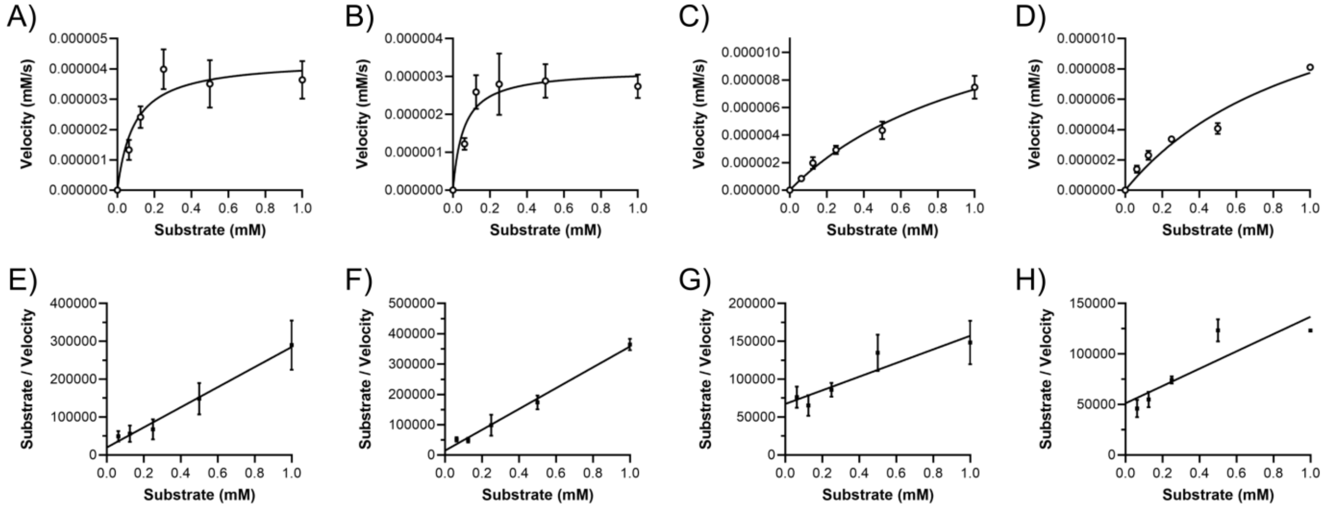

Figure S23. Michaelis-Menten plots for substrate **7** using A) **Ht-Au8**, B) **Ht-Au9**, C) **Ht-Au10**, D) **Ht-Au11**. Hanes-Woolf plot for substrate **7** using E) **Ht-Au8**, F) **Ht-Au9**, G) **Ht-Au10**, H) **Ht-Au11**. The concentration of each ArM used to acquire this kinetic data was set to 50 µM.

4.8 Protein complexes used for cell imaging studies

Scheme S8 depicts the general steps taken to produce the fluorescent HaloTag-containing proteins used in this study (**HtPA-FL**, **HtA-FL**, or **Ht-FL**). To do this, Azido-PEG3-C6-Cl (232 nmol, 116 µl from a 2 mM stock solution in DMSO) and DBCO-fluorescein (232 nmol, 116 µl from a 2 mM stock solution in DMSO) were coupled together by incubation at room temp for 2 hr. Afterwards, 116 µl of this mixture was added to 1048 µl of purified **HtPA**, **HtA**, or **Ht** (26 nmol, 25 µM stock solution in PBS buffer). The solution was then mildly mixed and incubated at room temp for 2 hr. To purify the fluorescein-labelled proteins, buffer exchange was performed using 10K MWCO protein concentrators to remove excess reagents. Subsequent volume reduction to the desired concentrations were done to provide **HtPA-FL**, **HtA-FL**, or **Ht-FL** solutions for testing.

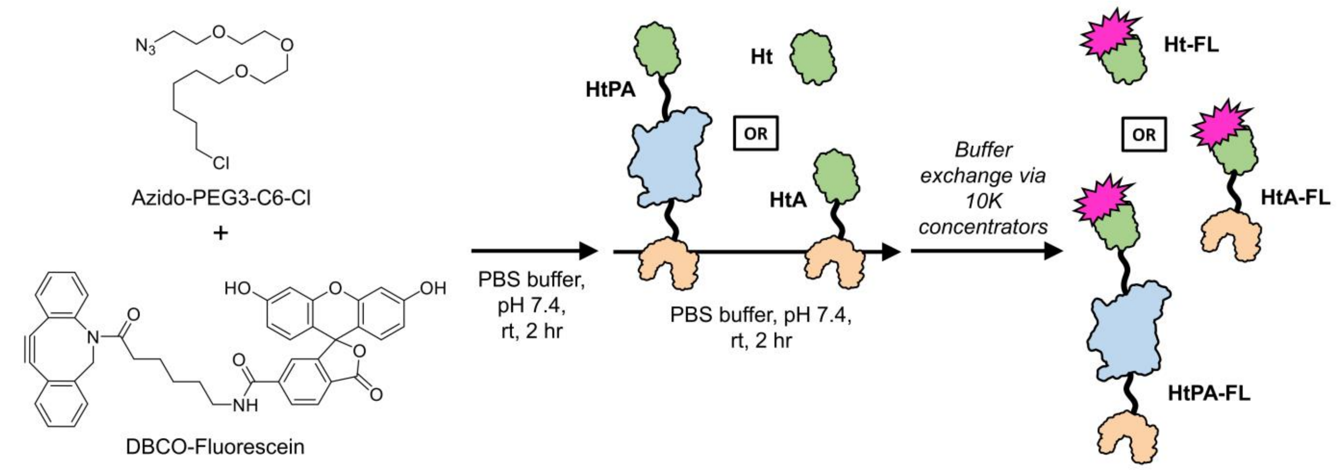

Scheme S8. Preparation of fluorescent HaloTag-containing proteins (**HtPA-FL**, **HtA-FL**, **Ht-FL**) used in this study.

5. Cell-based Assays

5.1 General cell culture

In this study, the cancer cell lines SW620, and DU-145 were obtained from the Japanese Collection of Research Bioresources cell bank (Japan), A549 was obtained from the iCell Bioscience (China), while HeLa and MDA-MB-231 were obtained from ATCC (USA) via donation from Prof. Randy YC Poon. Cells were incubated at 37 °C in a humidified incubator with an atmosphere of 5% CO2 and 95% air. Specific growth media used are indicated as follows:

Name	Type	Medium	FBS	Penicillin-Streptomycin
HeLa	human cervix adenocarcinoma cells	DMEM	10%	1%
MDA-MB-231	human breast adenocarcinoma cells	DMEM	10%	1%
KKU-213	human cholangiocarcinoma cell	DMEM	10%	1%
SW620	human colon adenocarcinoma cells	RPMI 1640	10%	1%
DU-145	human prostate carcinoma cells	DMEM	10%	1%
A549	human lung adenocarcinoma cells	DMEM	10%	1%
NEU-treated MDA-MB-231	human breast adenocarcinoma cells	RPMI 1640	10%	1%

5.2 Statistical analysis

In this study, statistical analysis was performed using a one-way ANOVA with Tukey's multiple comparisons test. All numerical data is presented as mean ± s.e.m. of three replicates. *P<0.03, **P<0.002, ***P<0.0002, ****P<0.0001, ns = not significant.

5.3 Fluorescence activated cell sorting experiments

Total sialic acid content on cell surfaces was measured using metabolic labelling (Figure S24). 6-well plates were seeded with 5×10^5 of cells in 2 mL of appropriate growth media and incubated overnight at 37 °C. Cells were then incubated with fresh media supplemented with or without 40 μM of $Ac_4ManNAz$ (Lumiprobe). Following a 48 hr incubation time, the culture media was replaced with 2 mL of 10 μM DBCO-fluorescein (Lumiprobe) in PBS with 5% FBS. Cells were then incubated for an additional 1 hr at 37 °C, before adherent cells were washed four times with PBS buffer and harvested. Following centrifugation, $\sim1\times10^6$ of cell suspension was analyzed by fluorescence-activated cell sorting using a *FACSAria*™ III Cell Sorter (BD). Gating was set to 10,000 events using a FITC channel (250V) with other standard settings (filter=2.0, nozzle=85μm, flow rate=1.0 ml/min).

A)

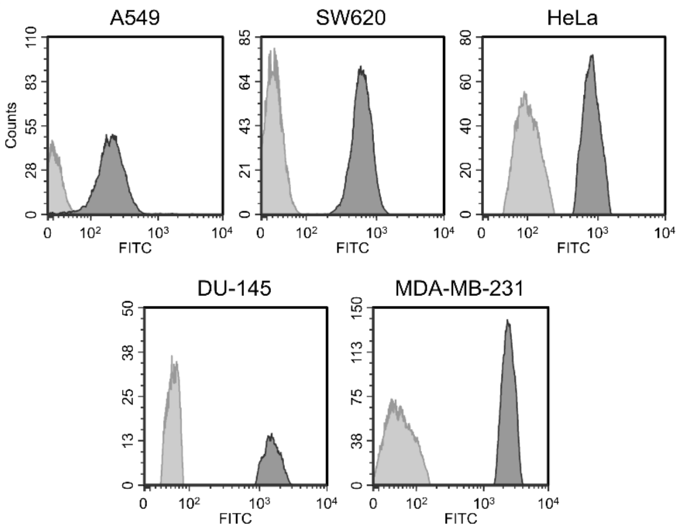

B)

Total sialic acid levels

Cell line	Cells + $Ac_4ManNAz$ + DBCO-FITC	Cells + DBCO-FITC
A549	169.84<br>202.40<br>206.80	4.40<br>9.68<br>12.32
SW620	524.29<br>631.11<br>753.19	15.26<br>16.35<br>19.62
HeLa	733.34<br>775.52<br>760.35	85.1<br>87.32<br>105.08
DU-145	1373.10<br>1190.25<br>940.70	65.55<br>55.20<br>42.55
MDA-MB-231	2026.22<br>2261.52<br>2463.61	48.60<br>54.27<br>56.70

Figure S24. Metabolic labelling studies designed to determine the relative levels of surface accessible sialic acid for various cancer cell lines.

5.4 Cell imaging studies

Fluorescent cell imaging was conducted to monitor the cancer-targeting potential of **HtPA** (along with relevant controls). For the general cell imaging experiments, various cancer cells were simply plated onto 24-well plates at approximately 2.5×10^4 cells/well and then grown overnight at 37 °C. These cells were directly used for the next step.

For imaging experiments using desialylated cells, MDA-MB-231 cells grown in RPMI media were plated onto 24-well plates at approximately 2.5×10^4 cells/well and then grown overnight at 37 °C. To initiate desialylation, cells were incubated at 37 °C with serum-free RPMI media supplemented with or without *Clostridium perfringens* neuraminidase (Sigma) at a final concentration of (0.2 U/ml). After 1 hr, cells were washed with serum-free RPMI growth media (2×) to remove any remaining neuraminidase and cleaved sialic acid products.

To begin all imaging experiment, current media was removed and replaced with 160 µl of fresh growth media and 40 µl of protein under study (50 µM stock solution of **HtPA-FL**, **HtA-FL**, or **Ht-FL**) to give a final concentration of 10 µM. After a 6 hr incubation period at 37 °C, cells were washed with PBS buffer (2×), fixed with 300 µl of a 4% formaldehyde solution for 20 min at room temperature, and then washed again with PBS buffer (2×). Cell imaging was then carried out using a Cell Discoverer 7 microscope (Zeiss) at 5× magnification where brightfield images were obtained at a 2 millisec exposure setting and fluorescent images were obtained at a 200 millisec exposure setting using the FITC channel. Obtained brightfield images were processed by cellpose 2.0 software to obtain regions of interest (ROIs) around individual cells. These ROIs were then imported into ImageJ to quantify the average individual cell fluorescence, and then used to generate box and whisker plots (displaying the 90/10 percentile at the whiskers, the 75/25 percentiles at the boxes, and the median in the center line). The uncropped cell images used for quantification are shown in Figure S25-S30.

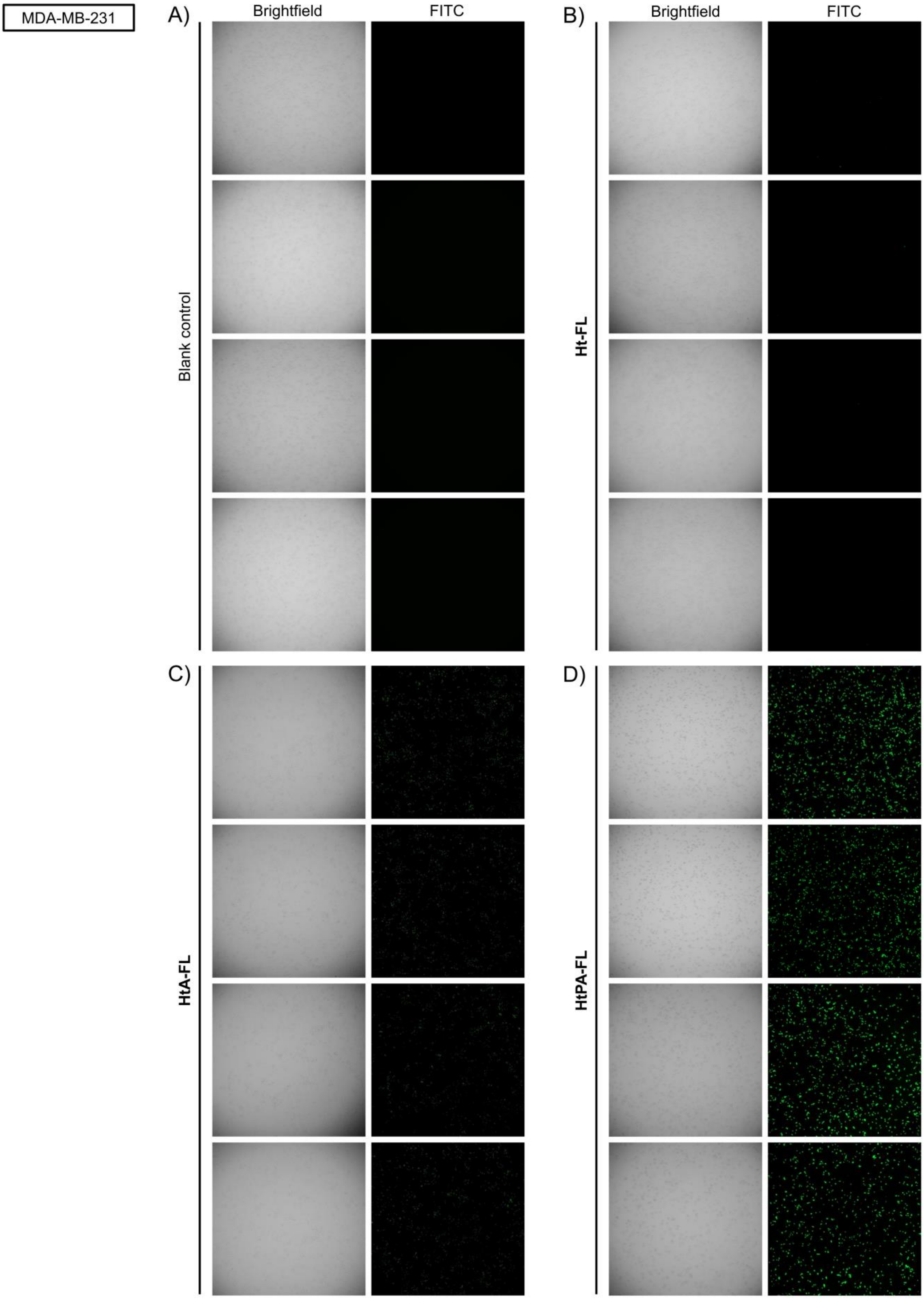

Figure S25. Imaging studies of MDA-MB-231 cells incubated A) PBS buffer (blank control), B) **Ht-FL** (10 μM), B) **HtA-FL** (10 μM), or D) **HtPA-FL** (10 μM) for 6 hr.

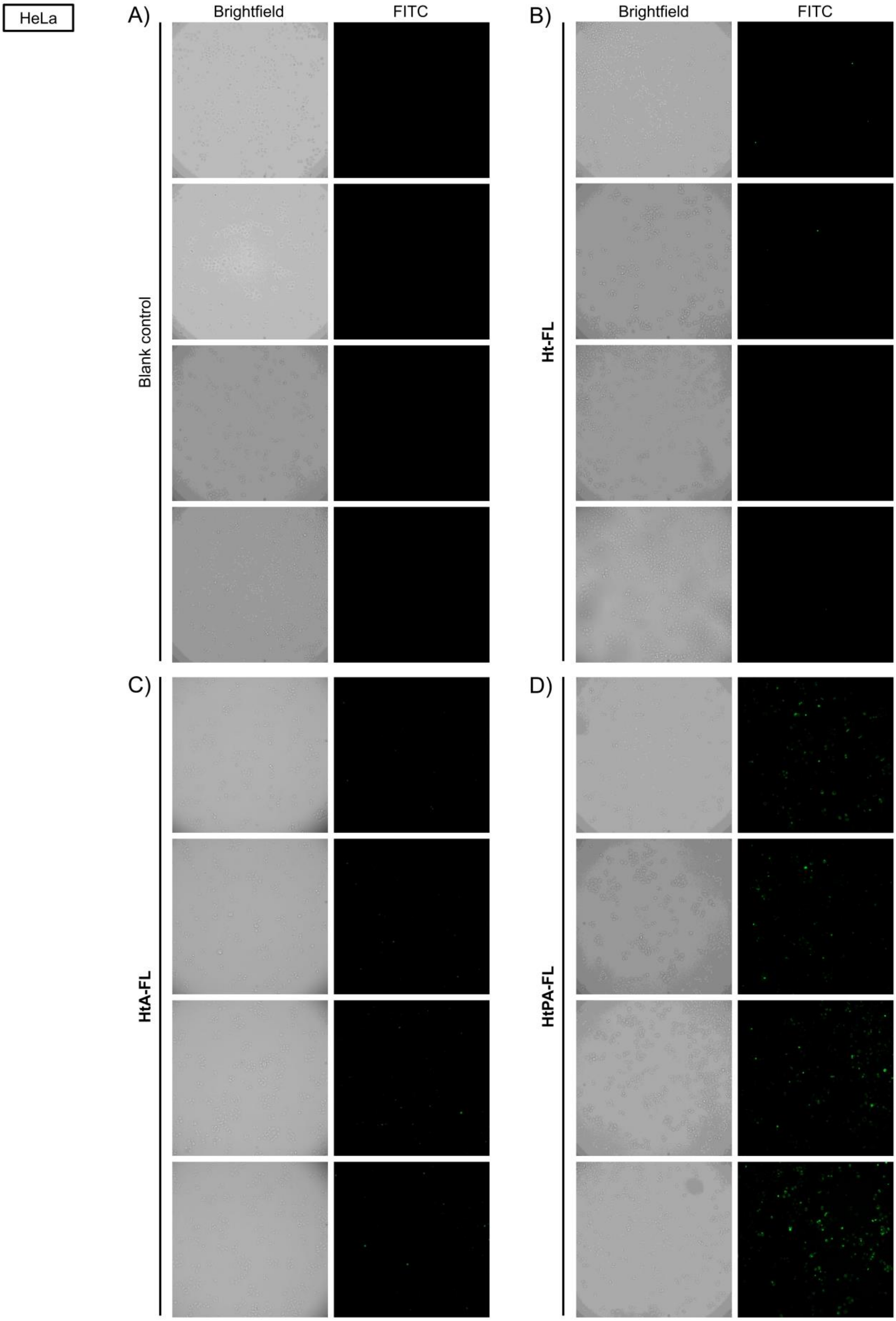

Figure S26. Imaging studies of HeLa cells incubated A) PBS buffer (blank control), B) **Ht-FL** (10 μM), B) **HtA-FL** (10 μM), or D) **HtPA-FL** (10 μM) for 6 hr.

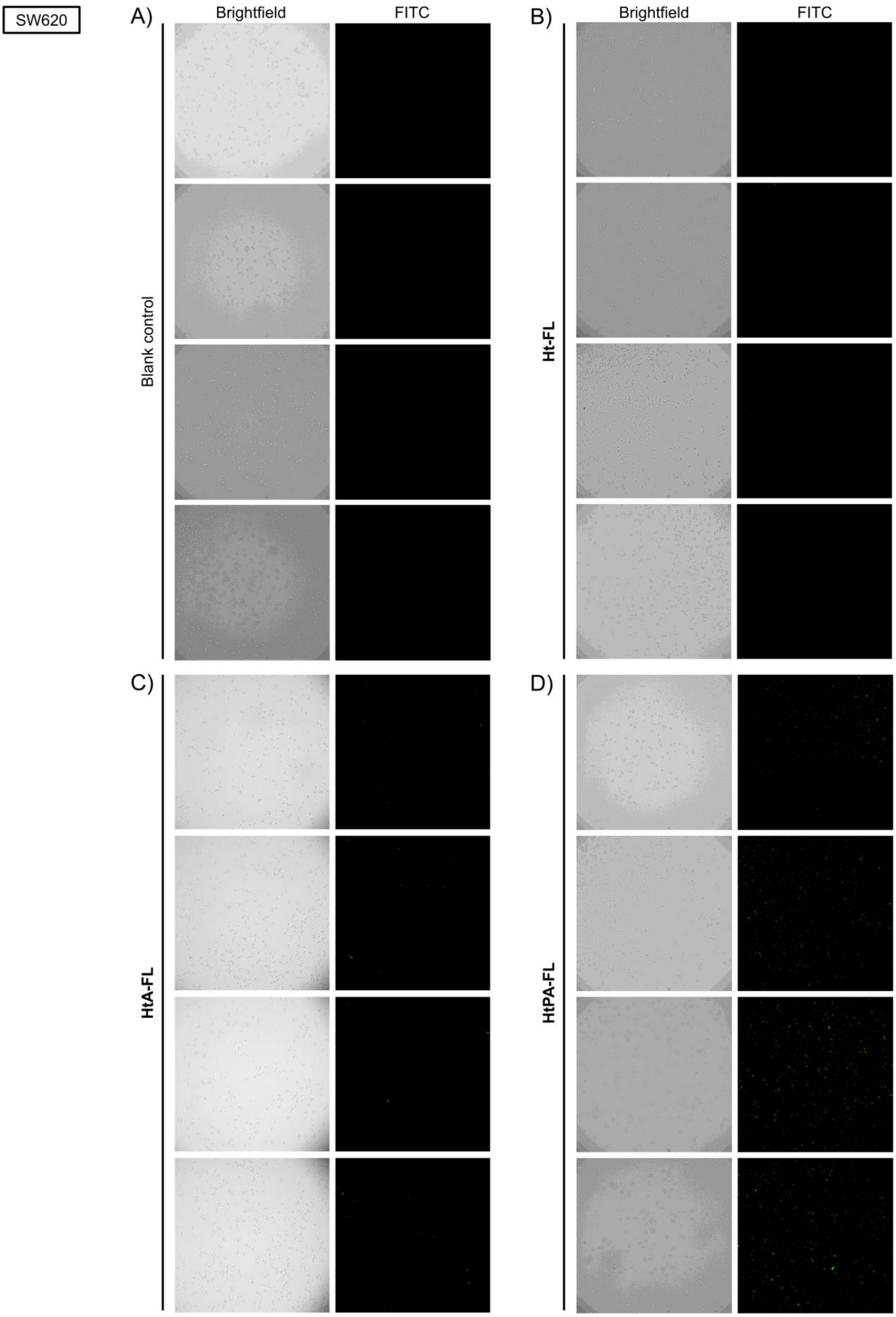

Figure S27. Imaging studies of SW620 cells incubated A) PBS buffer (blank control), B) **Ht-FL** (10 μM), B) **HtA-FL** (10 μM), or D) **HtPA-FL** (10 μM) for 6 hr.

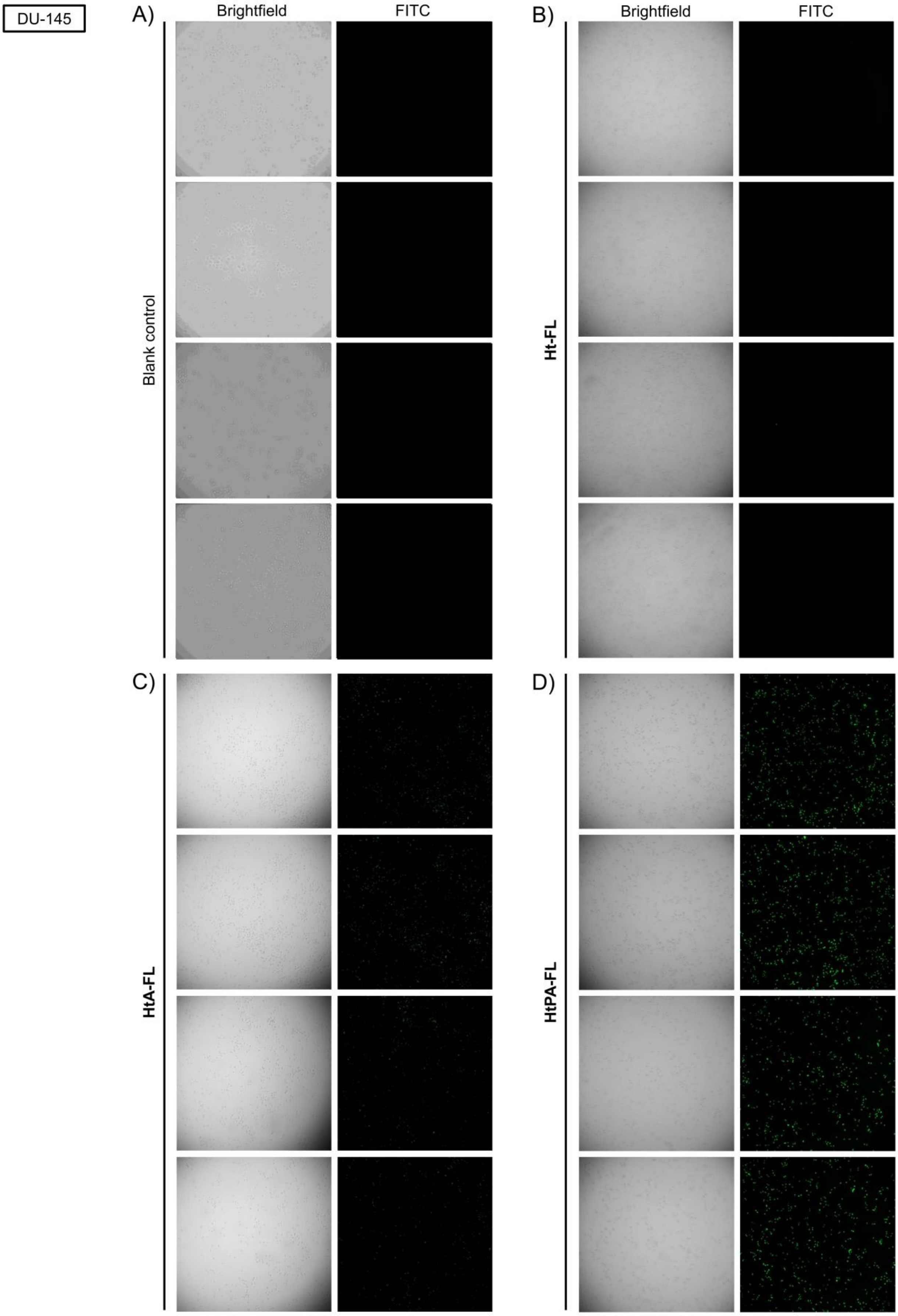

Figure S28. Imaging studies of DU-145 cells incubated A) PBS buffer (blank control), B) **Ht-FL** (10 μM), B) **HtA-FL** (10 μM), or D) **HtPA-FL** (10 μM) for 6 hr.

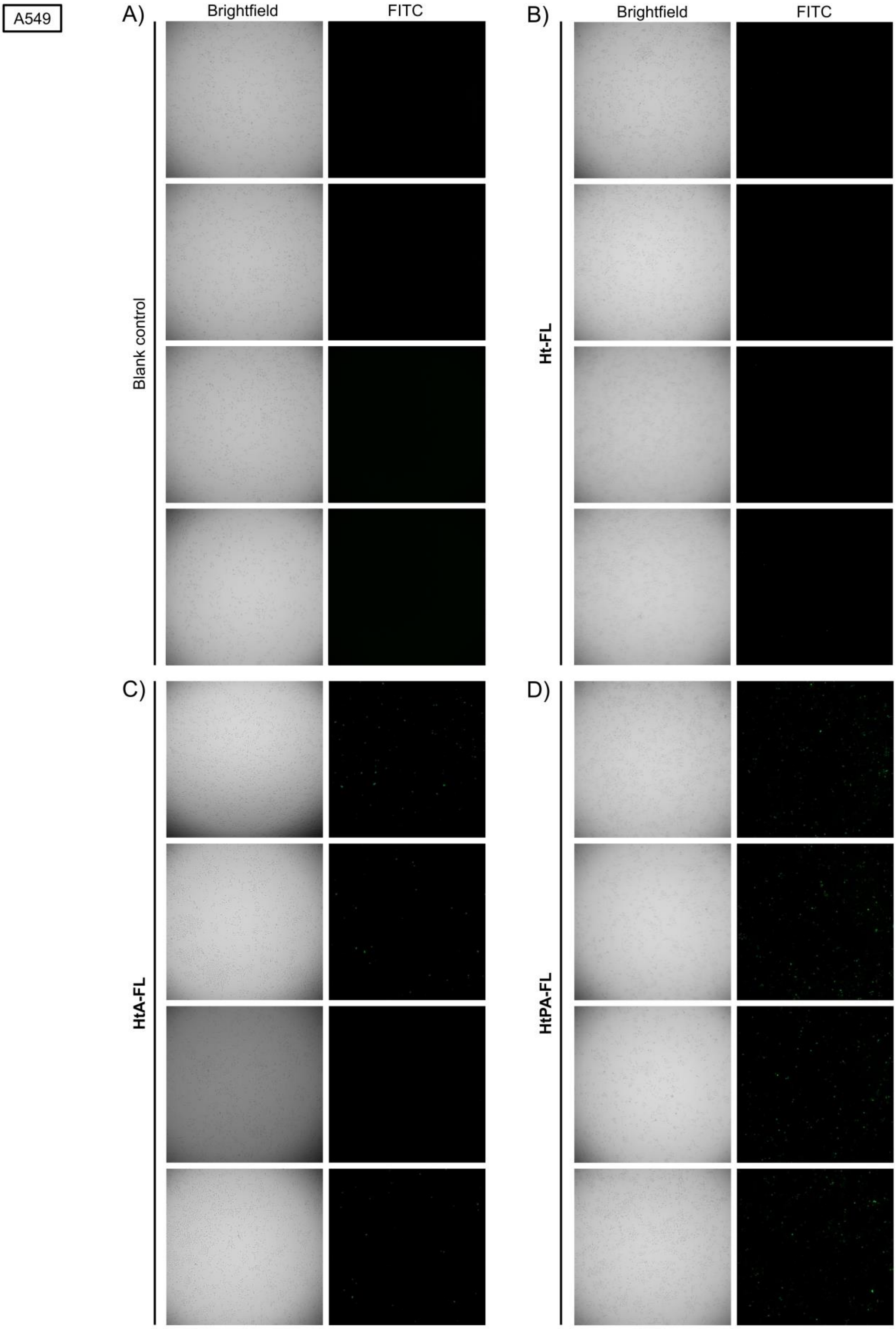

Figure S29. Imaging studies of A549 cells incubated A) PBS buffer (blank control), B) **Ht-FL** (10 μM), B) **HtA-FL** (10 μM), or D) **HtPA-FL** (10 μM) for 6 hr.

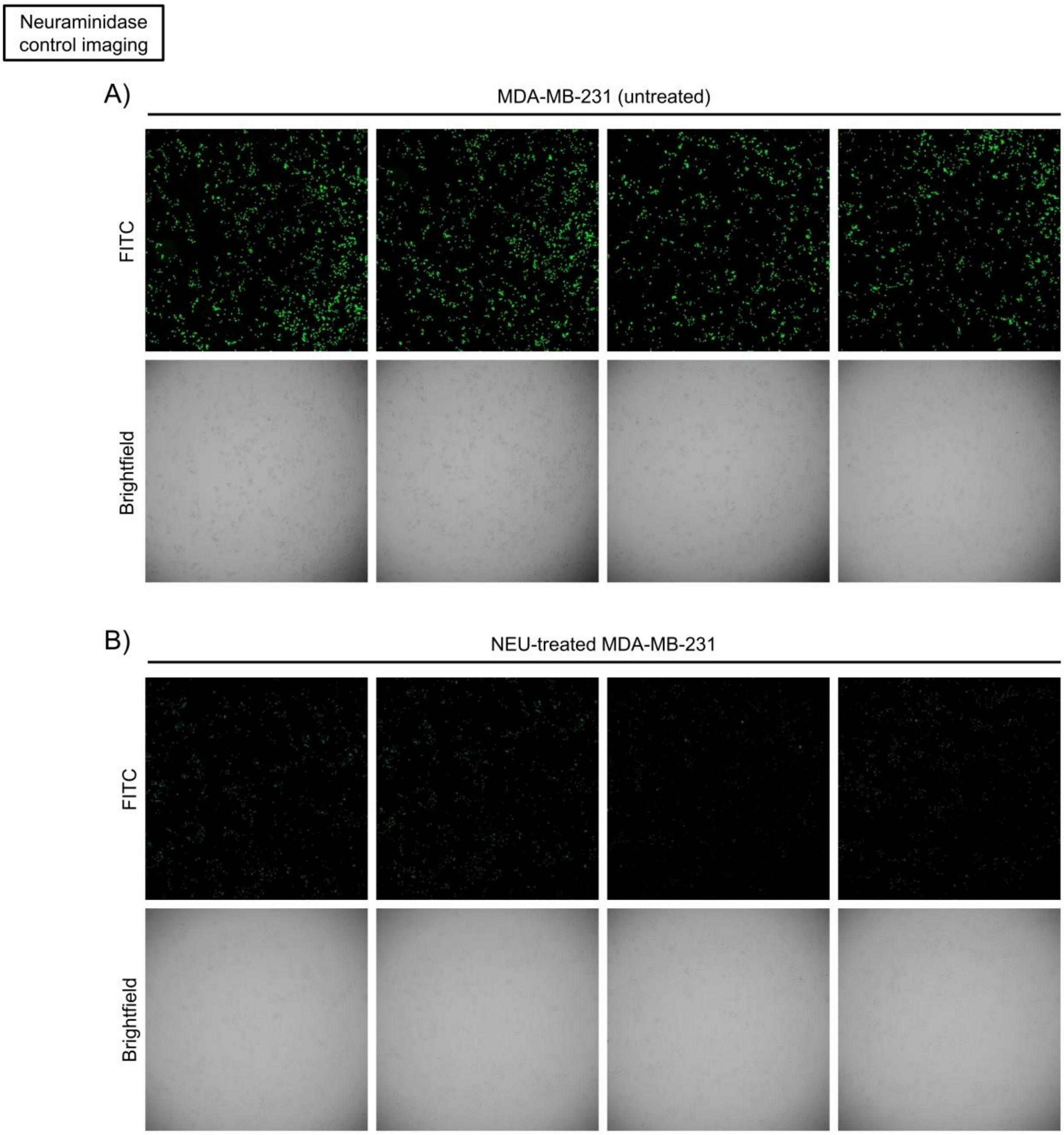

Figure S30. Desialylation cell imaging controls. To ensure artificial metalloenzyme targeting is due to sialic acid binding, cell imaging was performed using **HtPA-FL** (10 µM) incubated with MDA-MB-231 cells either A) untreated, or B) treated with *Clostridium perfringens* neuraminidase (0.2 U/ml) for 1 hr. After cells of both conditions were incubated with **HtPA-FL** for 6 hr, cells were washed and imaged.

5.5 Cell cytotoxicity studies

Cell viability was determined using a colorimetric MTS Assay Kit (Abcam). Based on cell titration experiments (data not shown), cells were first seeded onto 96-well plates at a density of 1000 cells per well and then grown overnight at 37 °C. On the following day, the media was replaced with the addition of varying compound mixtures (20 µl) and growth media (80 µl). For the mixtures, these contained varying concentrations of prodrug **10** and/or **HtPA-Au8**. As a control to determine cell populations at the time of treatment addition, a separately seeded plate was analyzed (via MTS reagent) and used for later data corrections. After an incubation period of 4 days, the media of the treated cells was removed and replaced with fresh growth media (80 µl) supplemented with MTS reagent (20 µl). After cells were further incubated at 37 ºC for 2 hr, cell viability was determined by the absorbance at 490 nm measured using a VANTAstar Microplate Reader (BMG). Growth curves for MDA-MB-231 treated with phenstatin **9** or prodrug **10** only are shown in Figure S31A. The toxicity of **HtPA-Au8** was also tested up to a concentration of 2 µM (Figure S31B).

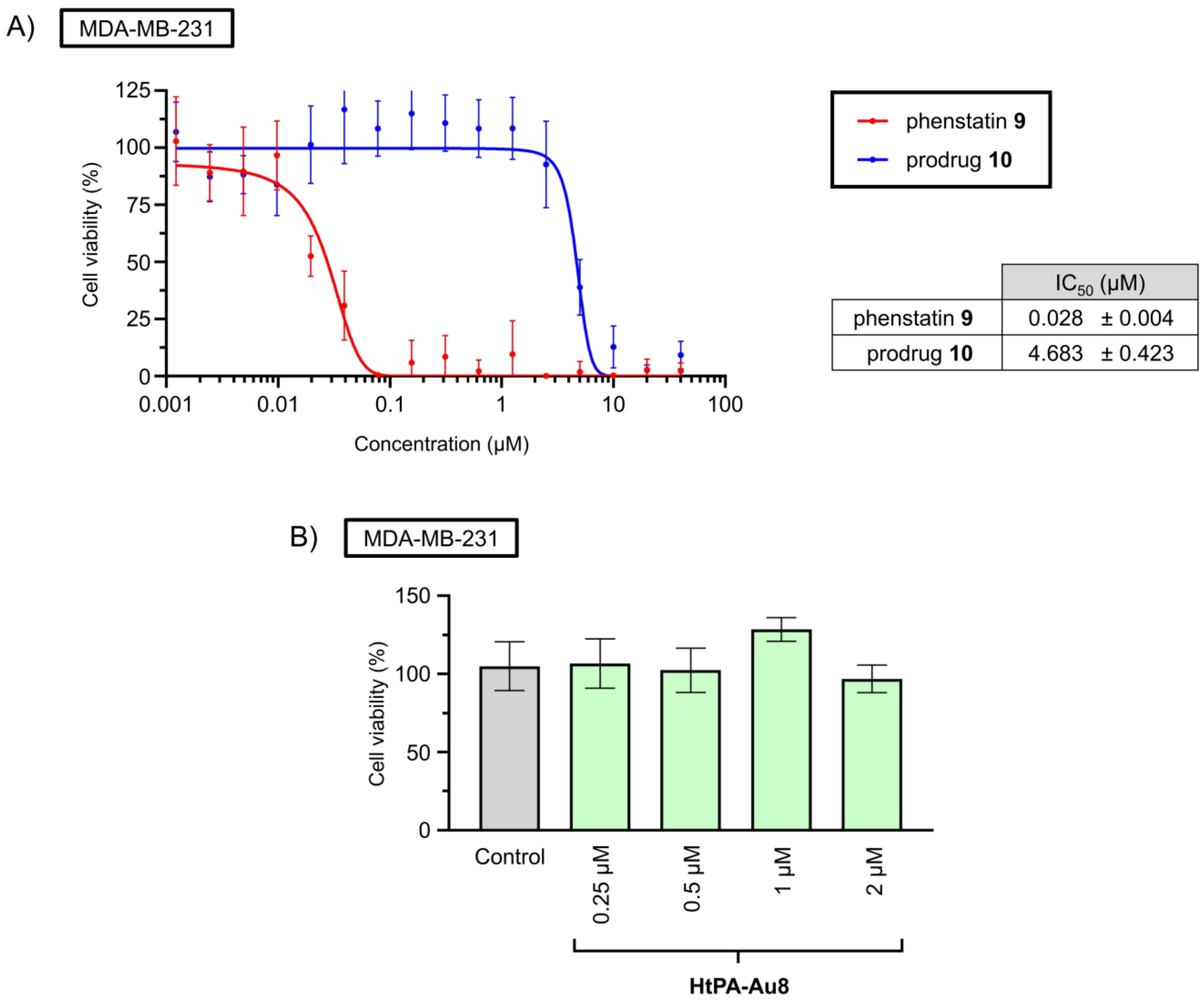

	IC_{50} (µM)
phenstatin **9**	0.028 ± 0.004
prodrug **10**	4.683 ± 0.423

Figure S31. Cell cytotoxicity assays. A) Growth curves were constructed for MDA-MB-231 cancer cells treated with either phenstatin **9** (red) or prodrug **10** (blue). Subsequent analysis allowed determination of the IC_{50} for each compound. B) Cell viability tests for MDA-MB-231 cancer cells treated with **HtPA-Au8** to determine its intrinsic toxicity. All numerical data is presented as mean ± s.e.m. of three replicates.

5.6 LC/MS analysis

Confirmation of drug formation in cells was determined through analysis of cell lysates. MDA-MB-231 cells were first seeded onto 6-well plates at a density of 500,000 cells per well and then grown overnight at 37 °C. On the following day, the media was replaced with the addition of varying compound mixtures (300 µl) and growth media (1.2 ml). The mixtures are classified as treatment (150 µl of a 2 mM prodrug **10** solution in 10%DMSO/H_2O + 150 µl of a 100 µM **HtPA-Au8** solution in PBS buffer), drug (150 µl of a 2 mM phenstatin **9** solution in 10%DMSO/H_2O + 150 µl of PBS buffer), and prodrug (150 µl of a 2 mM prodrug **10** solution in 10%DMSO/H_2O + 150 µl of PBS buffer). After an incubation period of 1 day, the media was first removed. Cells were then trypsinized and collected by centrifugation in 1.5 ml Eppendorf tubes. The cell pellet was resuspended in 250 µl of PBS buffer before a freeze-thaw cell lysis procedure was carried out. In each round, cells were first flash frozen at -80 °C (acetone/dry ice bath), followed by melting in a 37 °C water bath. A total of 5 freeze-thaw rounds were carried out. The cell lysate suspension was then centrifuged. The supernatant was transferred to 1.5 ml Eppendorf tube, followed by an extraction with 150 µl of ethyl acetate. The organic layer was then transferred to a clean tube, dried to a solid, followed by the addition of acetonitrile (100 µl). Samples were injected onto a HPLC with an autosampler using various HPLC method 2. Mass spec analysis of the peak corresponding to phenstatin **9** confirmed the presence of formed drug inside the treated MDA-MB-231 cells. Acquired HPLC chromatograms are shown in Figure S32.

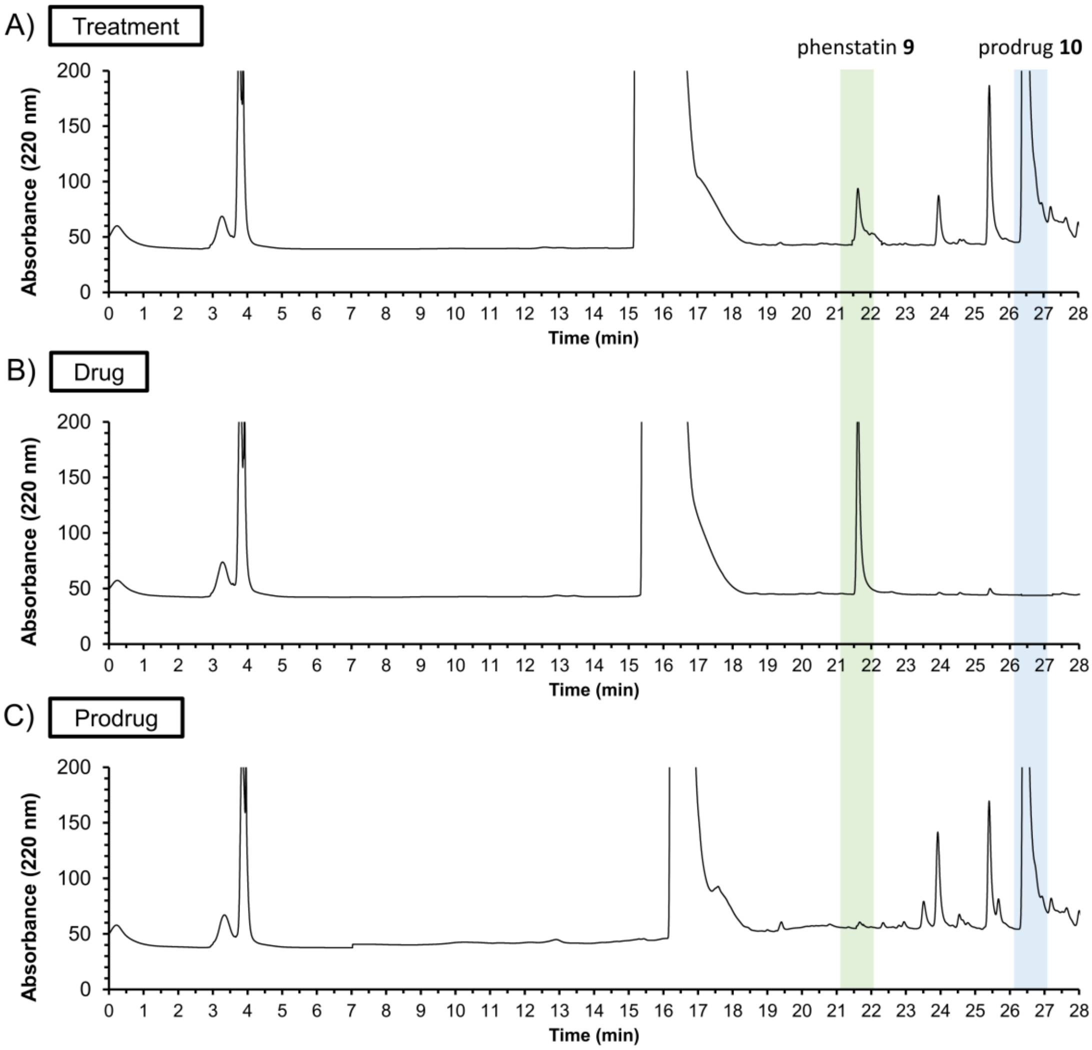

Figure S32. HPLC chromatograms of cellular lysates obtained from MDA-MB-231 cells treated with A) a mixture of prodrug **10** and **HtPA-Au8**, B) phenstatin **9**, and C) prodrug **10**.

6. NMR spectra

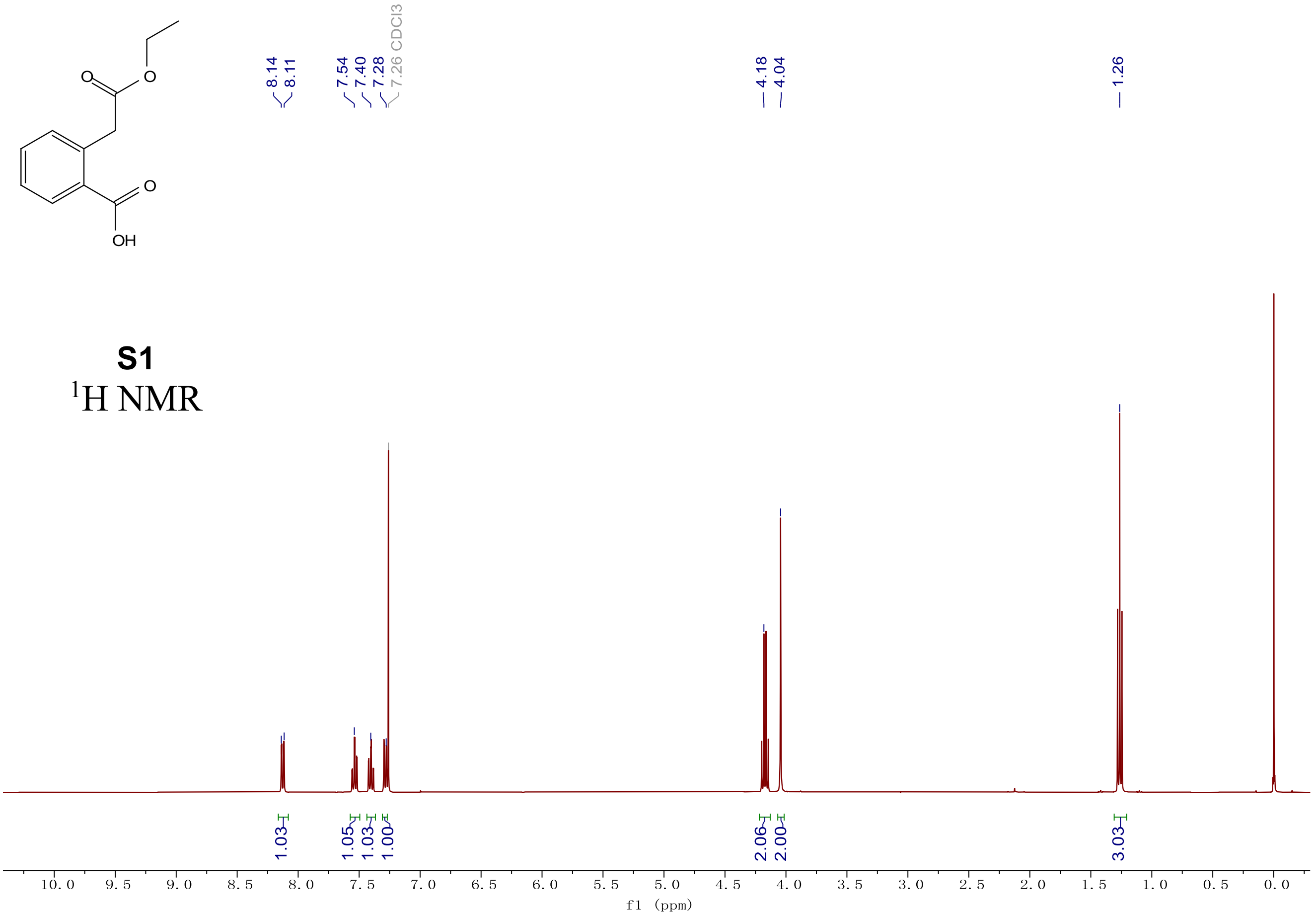

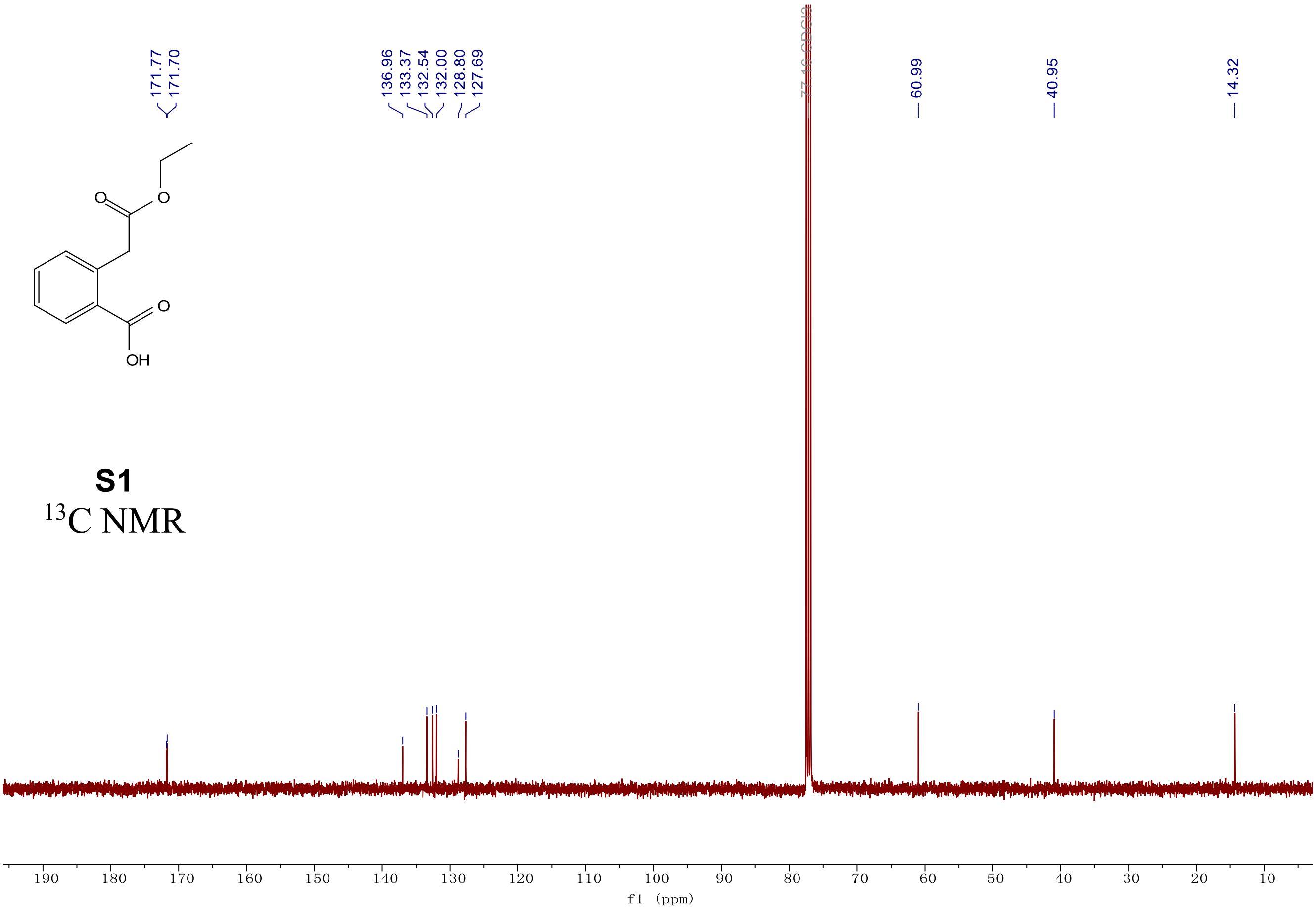

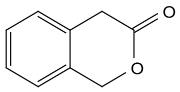

S2
^{1}H NMR

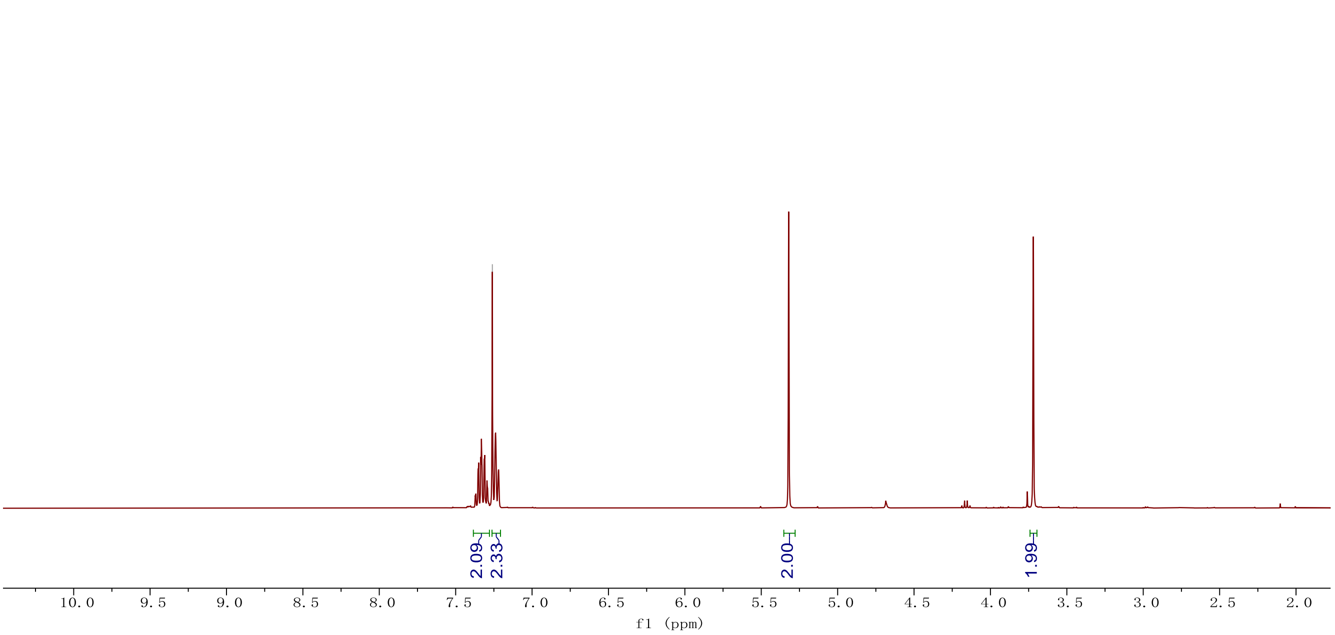

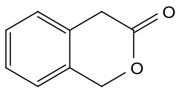

S2
^{13}C NMR

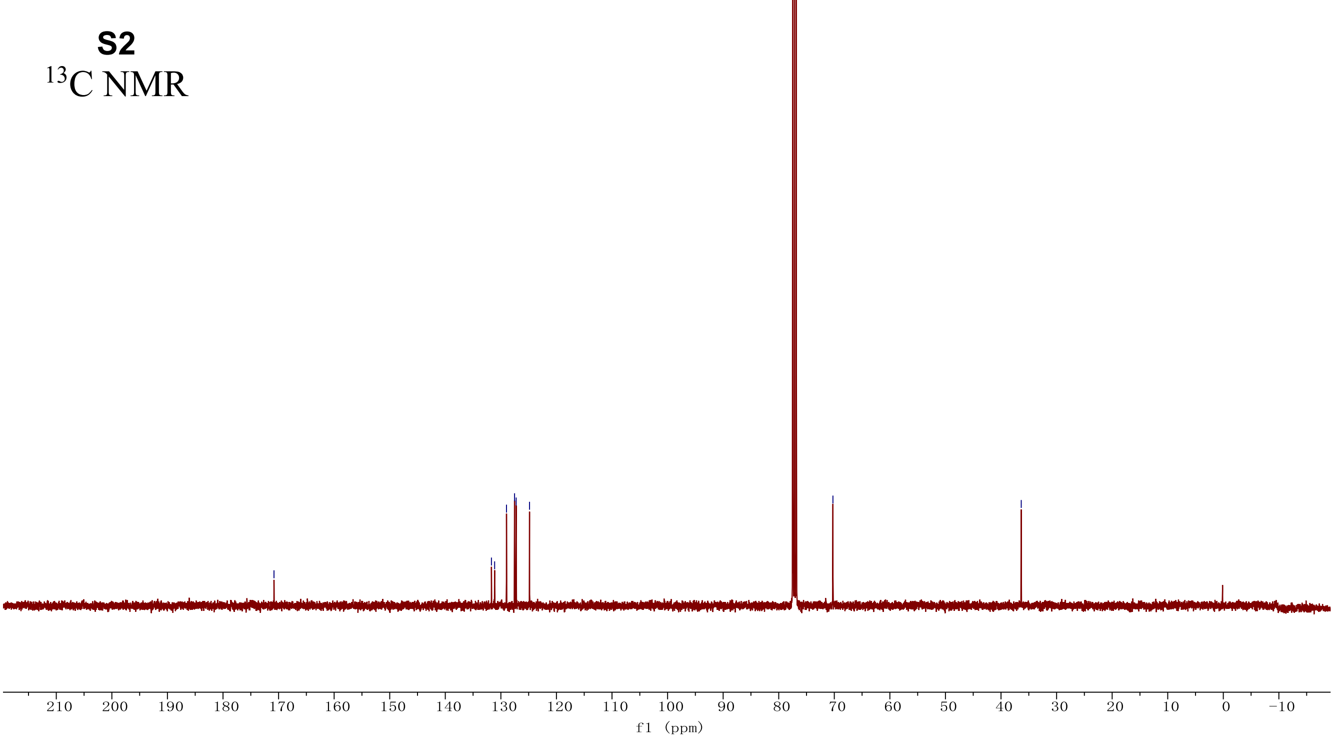

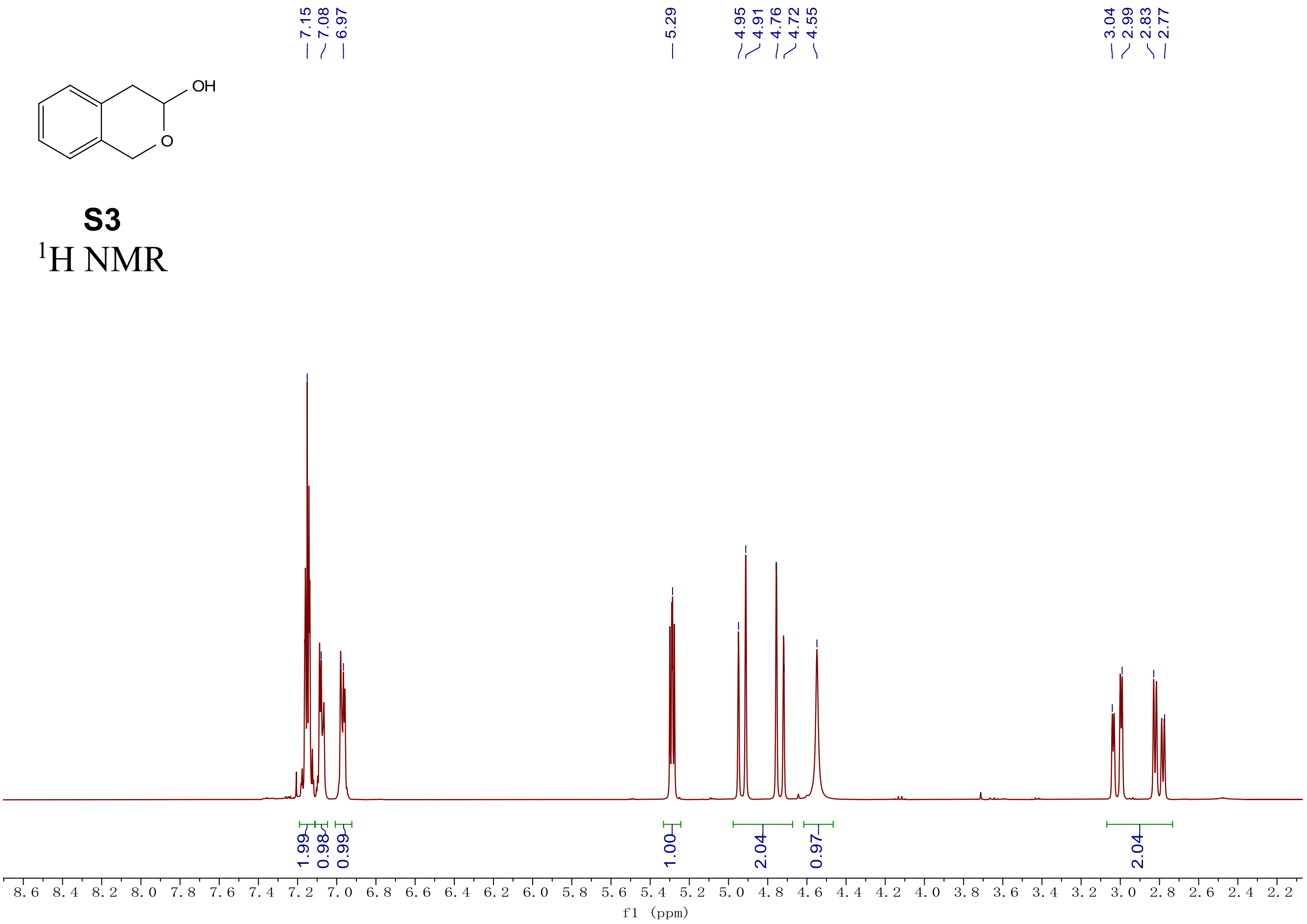

S3
^{1}H NMR

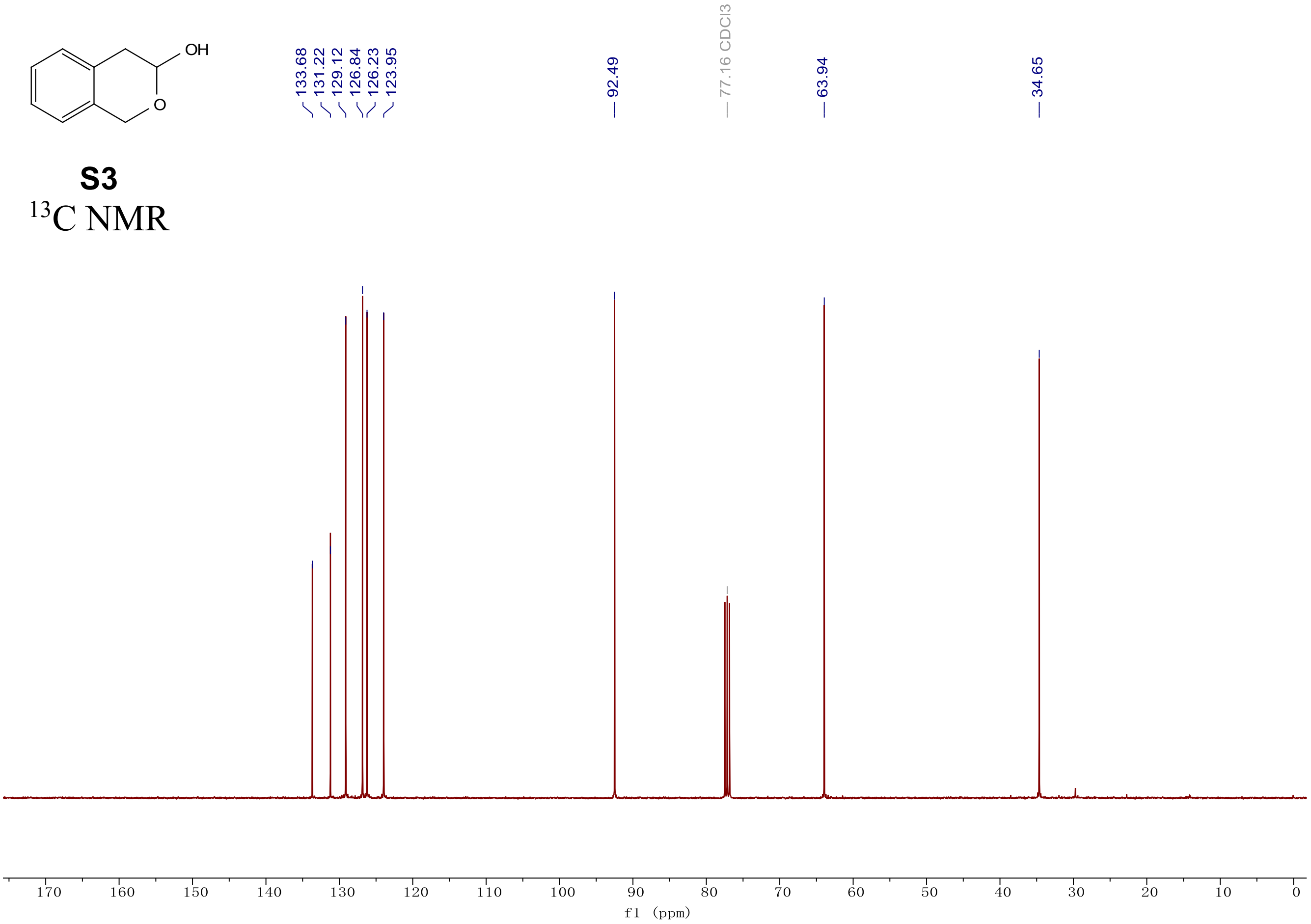

S3
^{13}C NMR

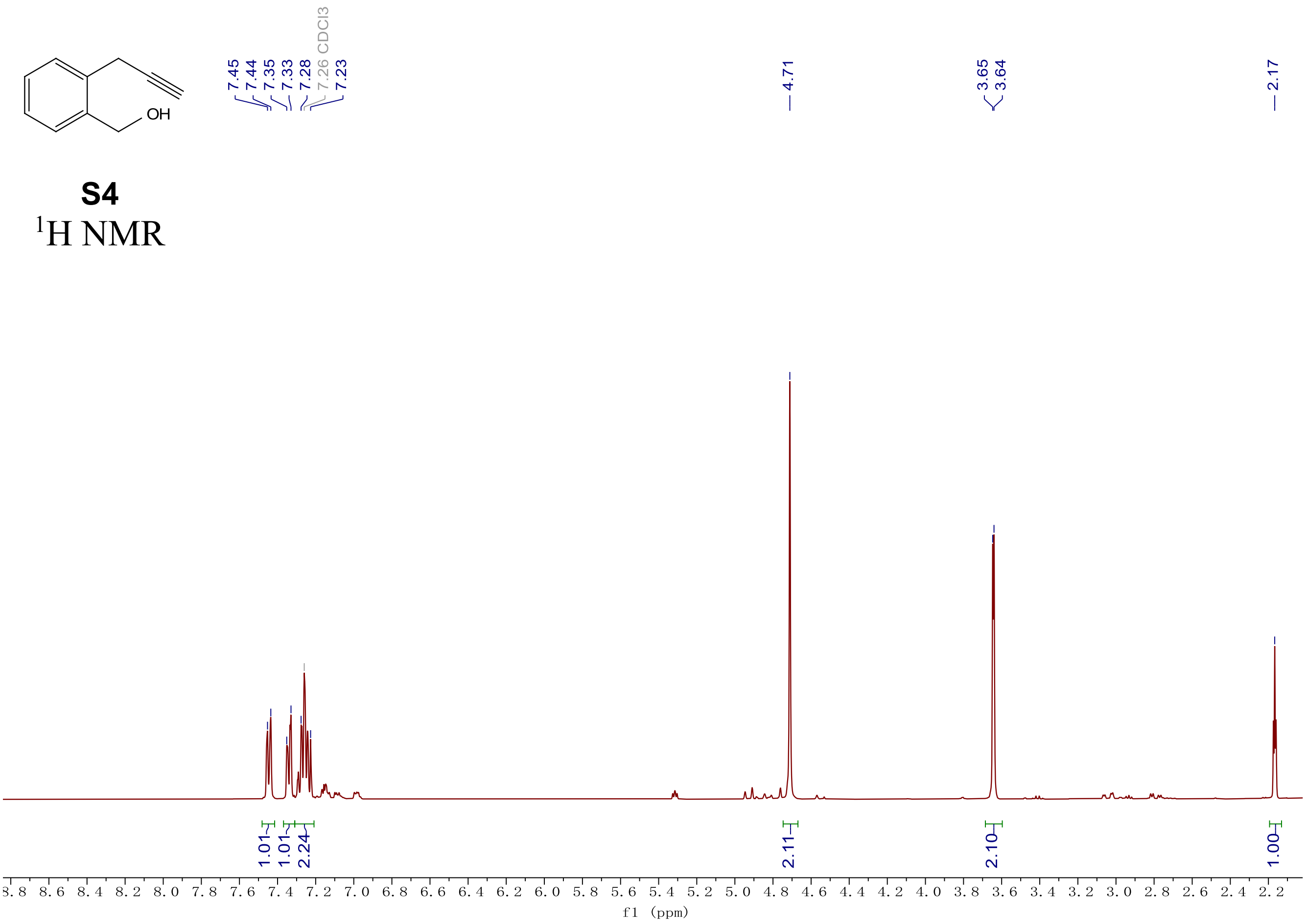

S4
^{1}H NMR

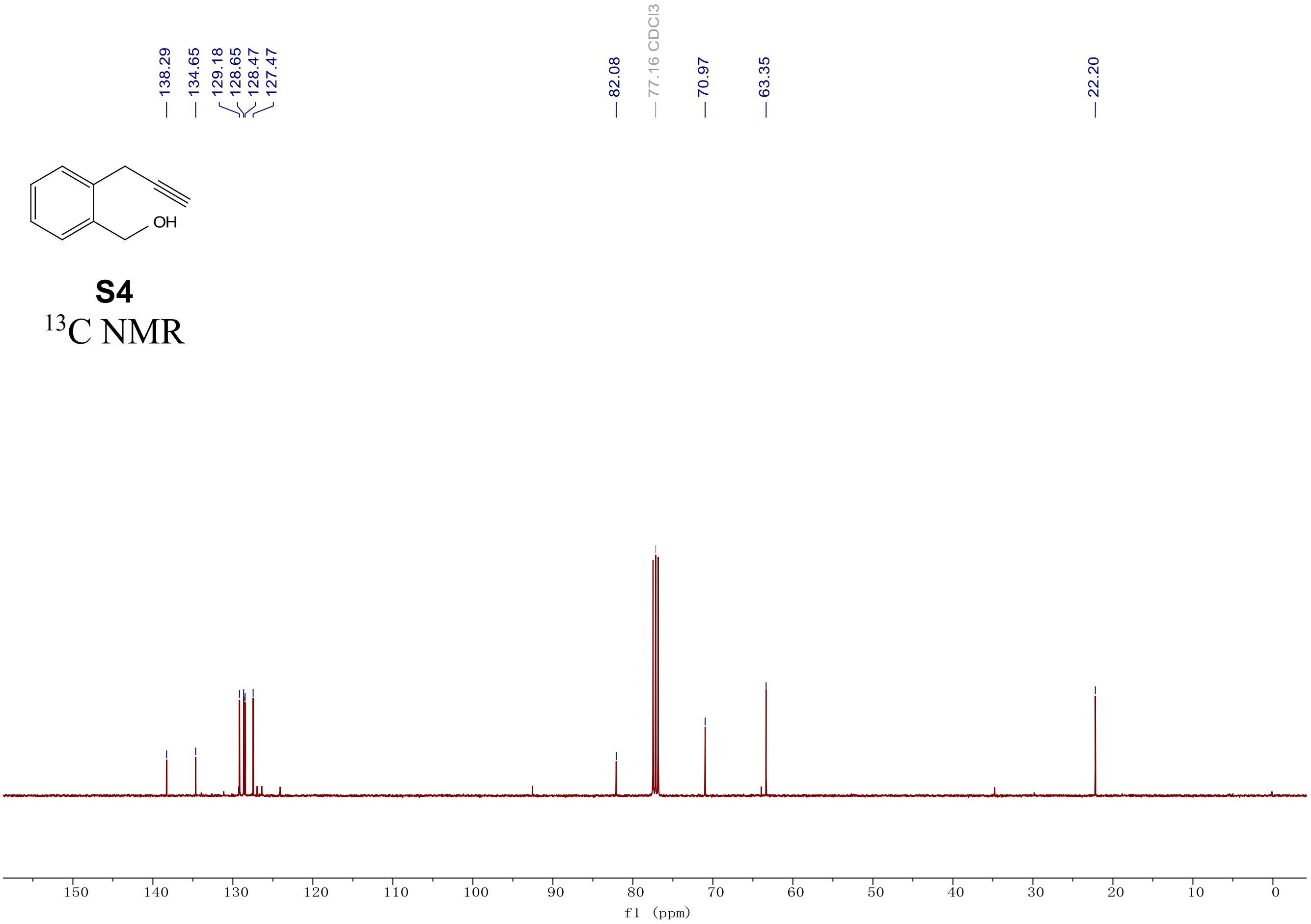

S4
^{13}C NMR

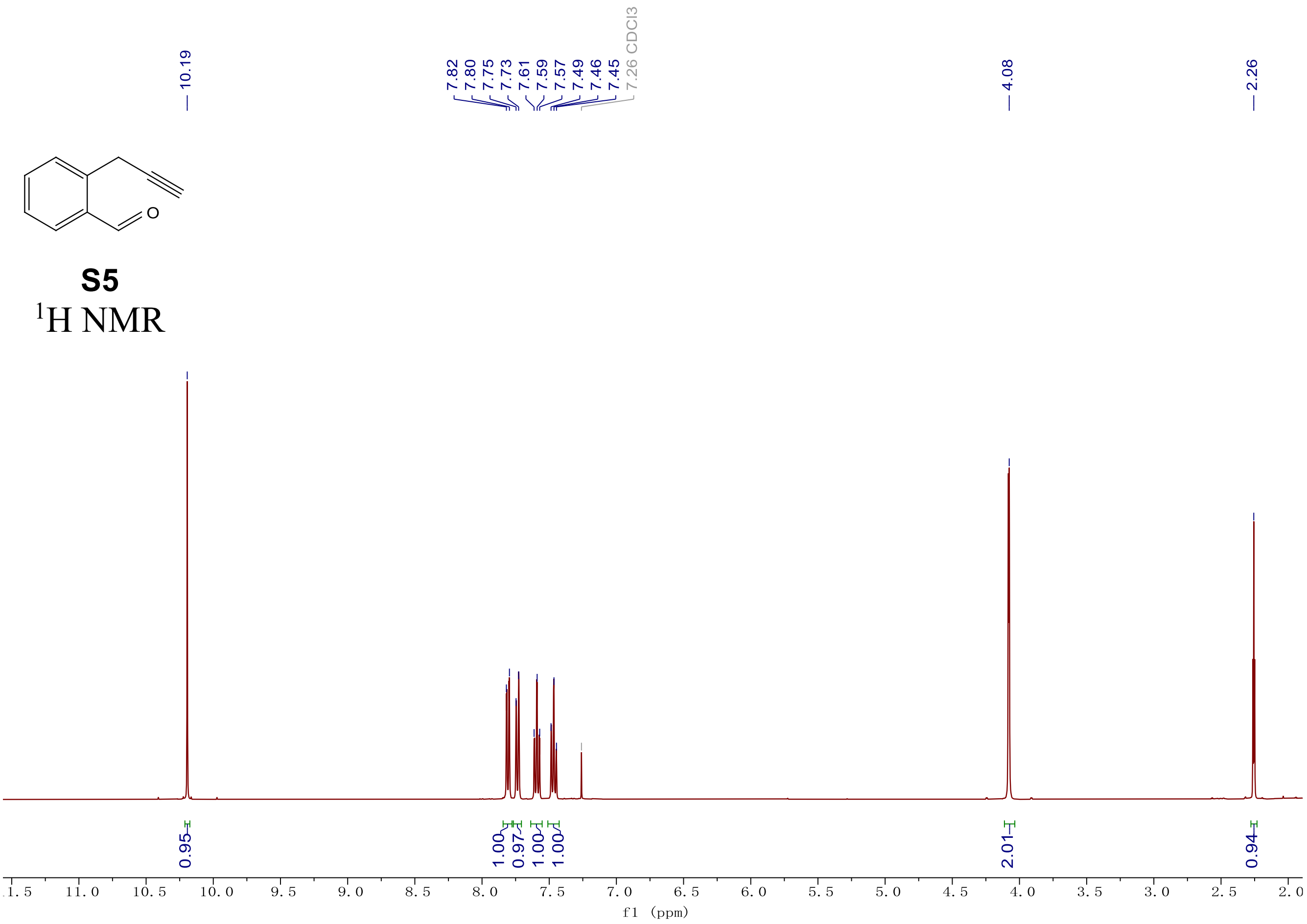
S5
^{1}H NMR
10.19
7.82 7.80 7.75 7.73 7.61 7.59 7.57 7.49 7.46 7.45
7.26 CDCl3
4.08
2.26
0.95
1.00 0.97 1.00 1.00
2.01
0.94
f1 (ppm)

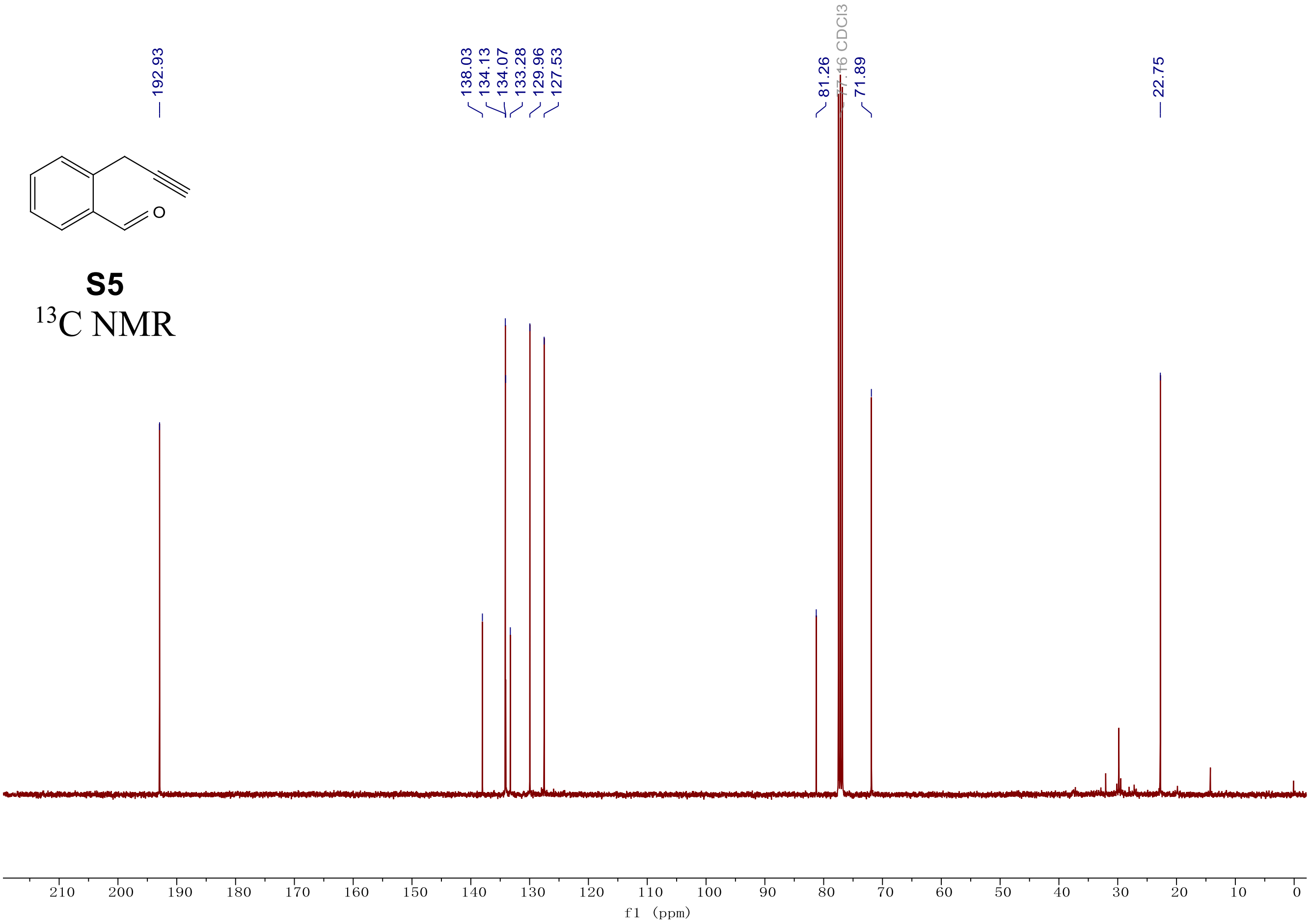
S5
^{13}C NMR
192.93
138.03 134.13 134.07 133.28 129.96 127.53
81.26
77.16 CDCl3
71.89
22.75
f1 (ppm)

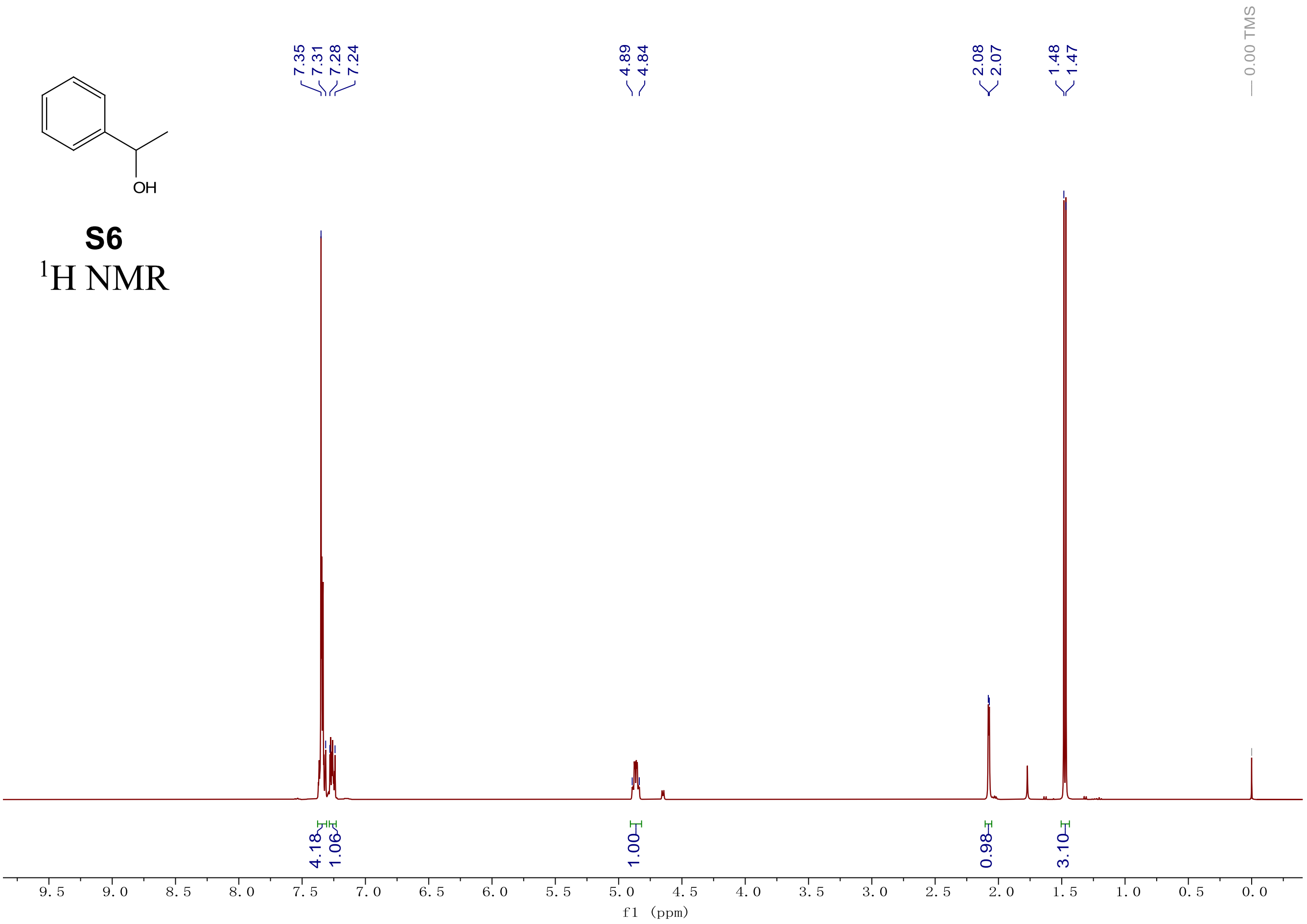
OH
S6
1H NMR
7.35
7.31
7.28
7.24
4.89
4.84
2.08
2.07
1.48
1.47
0.00 TMS
4.18
1.06
1.00
0.98
3.10
9.5 9.0 8.5 8.0 7.5 7.0 6.5 6.0 5.5 5.0 4.5 4.0 3.5 3.0 2.5 2.0 1.5 1.0 0.5 0.0
f1 (ppm)

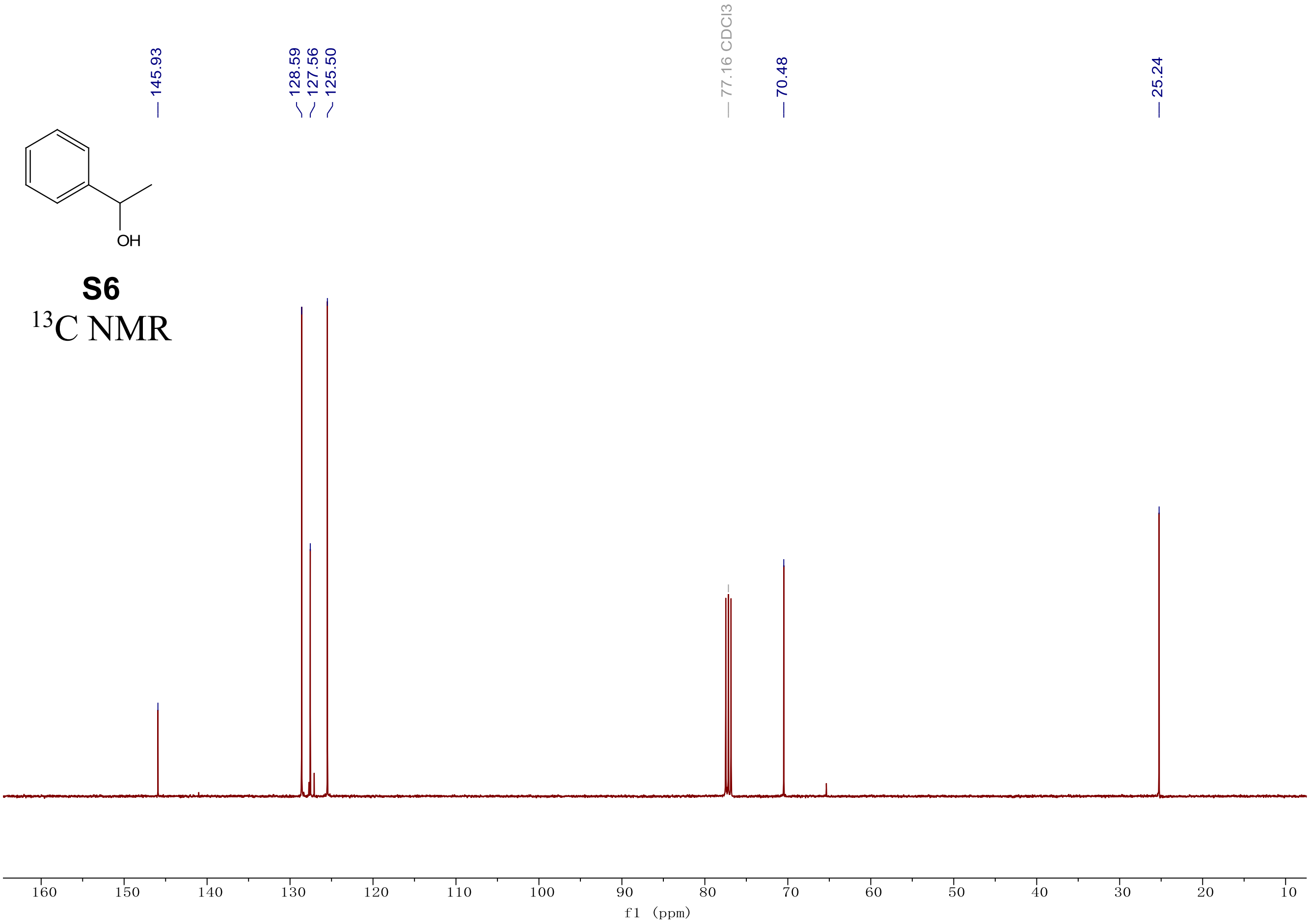
OH
S6
13C NMR
145.93
128.59
127.56
125.50
77.16 CDCl3
70.48
25.24
160 150 140 130 120 110 100 90 80 70 60 50 40 30 20 10
f1 (ppm)

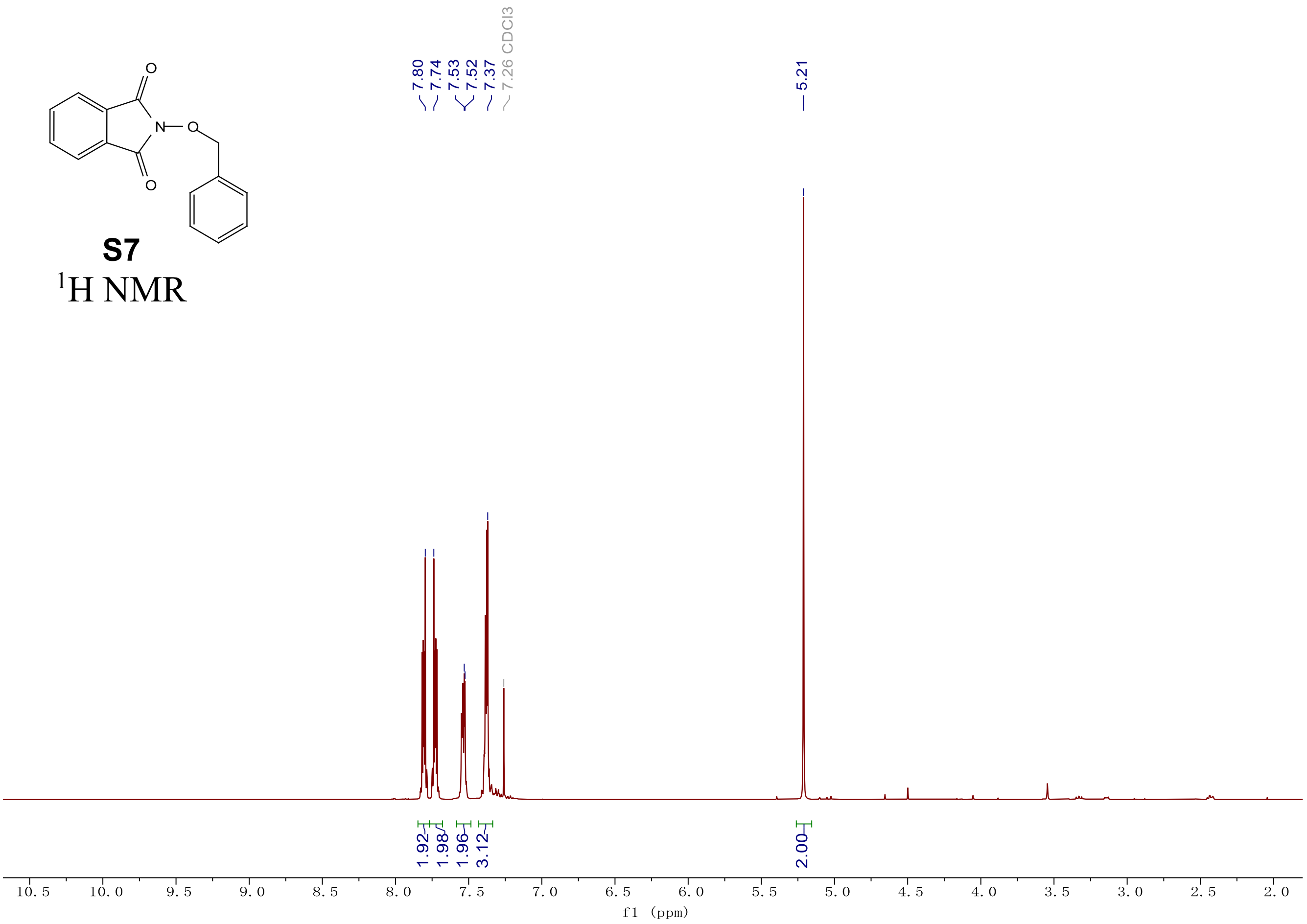

S7
1H NMR
7.80
7.74
7.53
7.52
7.37
7.26 CDCl3
5.21
1.92
1.98
1.96
3.12
2.00
10.5
10.0
9.5
9.0
8.5
8.0
7.5
7.0
6.5
6.0
5.5
5.0
4.5
4.0
3.5
3.0
2.5
2.0
f1 (ppm)

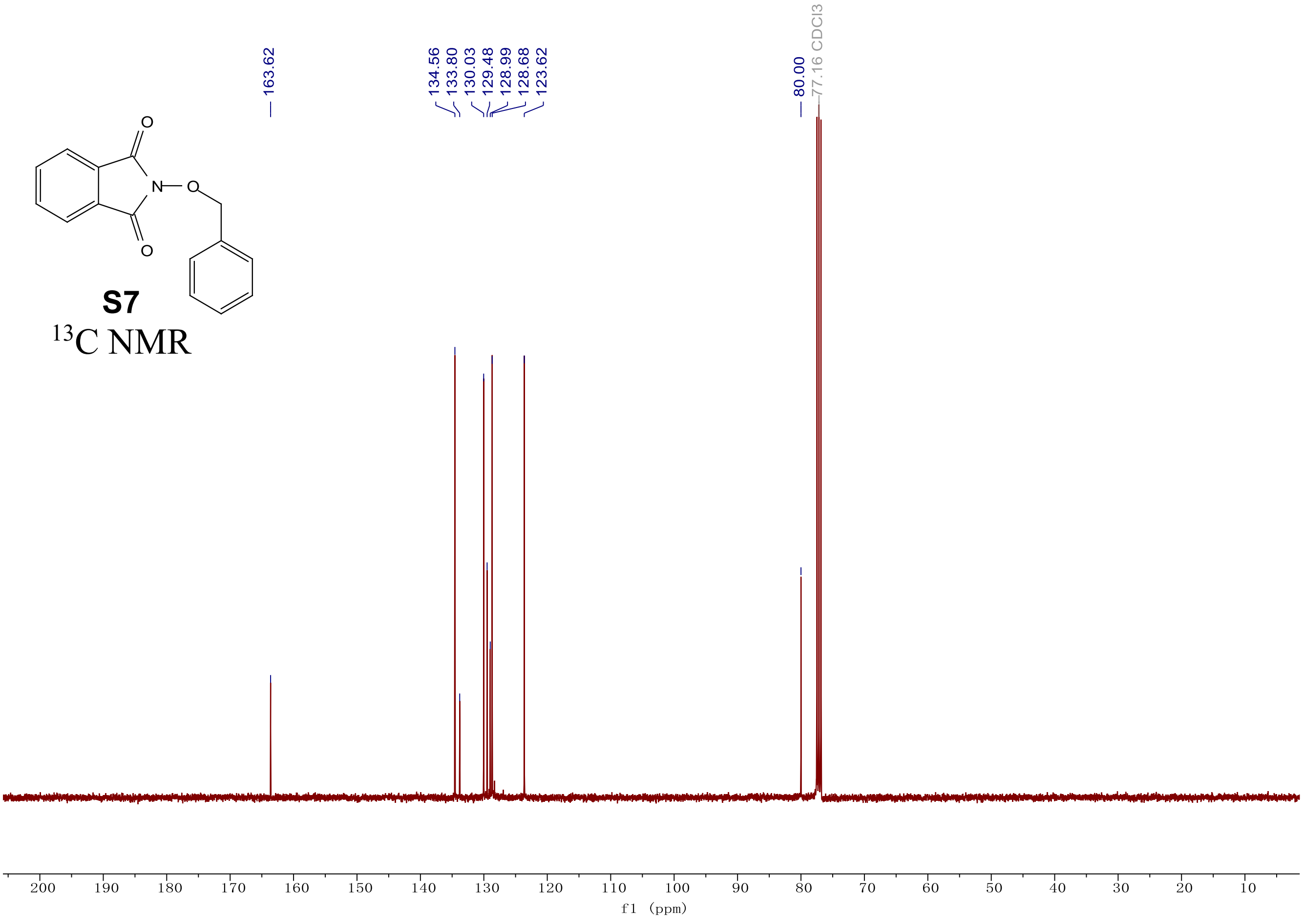

S7
13C NMR
163.62
134.56
133.80
130.03
129.48
128.99
128.68
123.62
80.00
77.16 CDCl3
200
190
180
170
160
150
140
130
120
110
100
90
80
70
60
50
40
30
20
10
f1 (ppm)

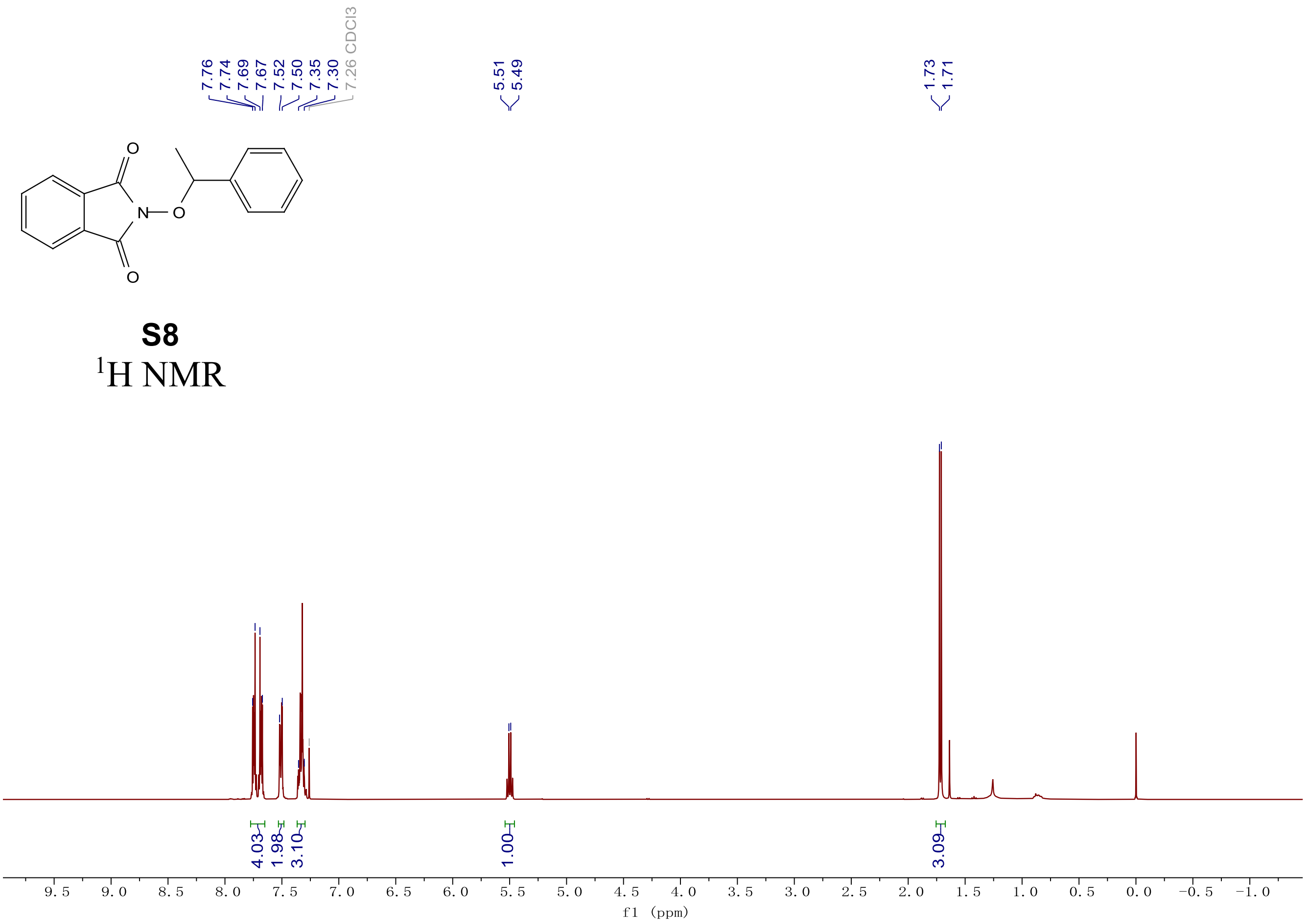

S8
^{1}H NMR

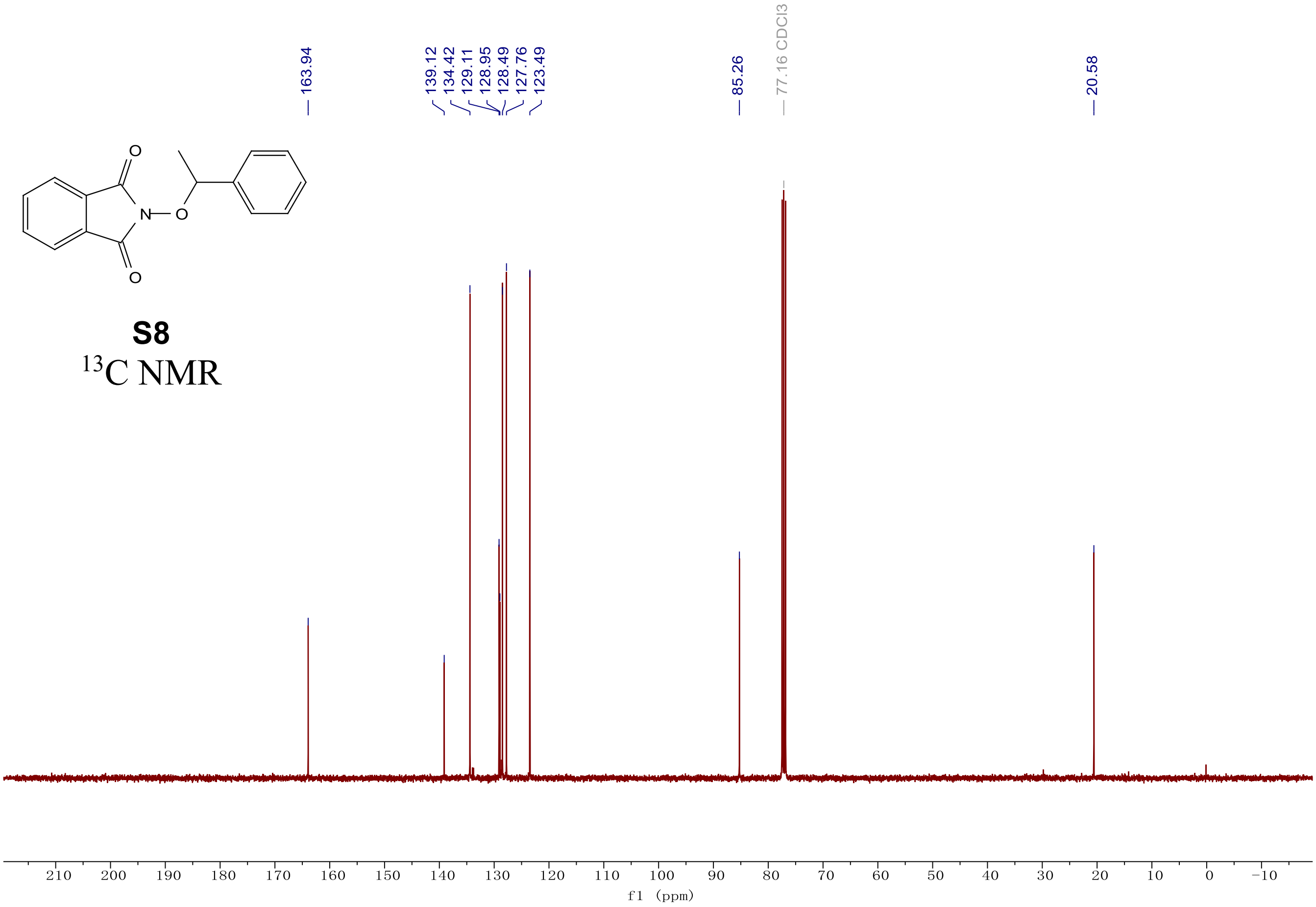

S8
^{13}C NMR

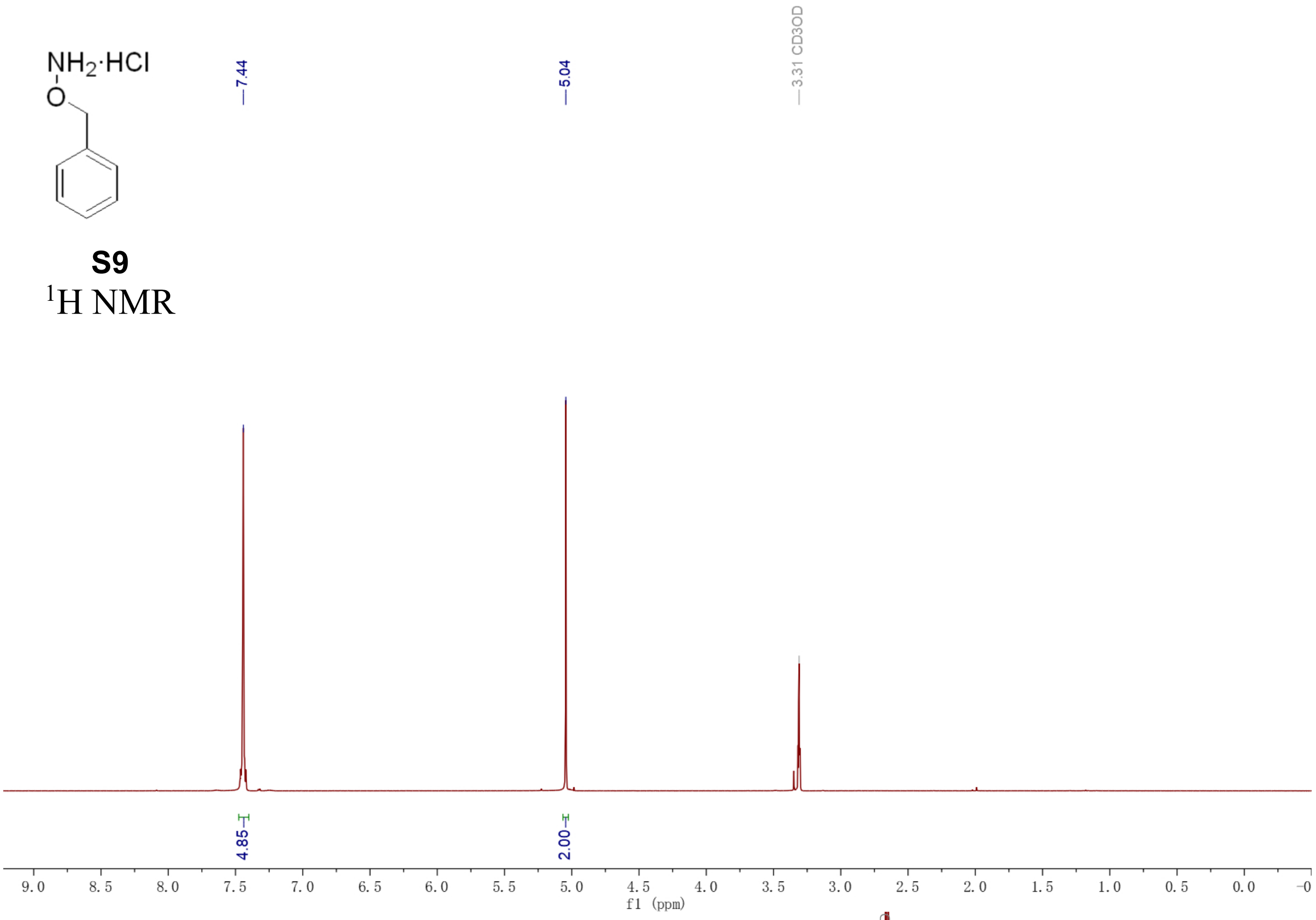

S9

^{1}H NMR

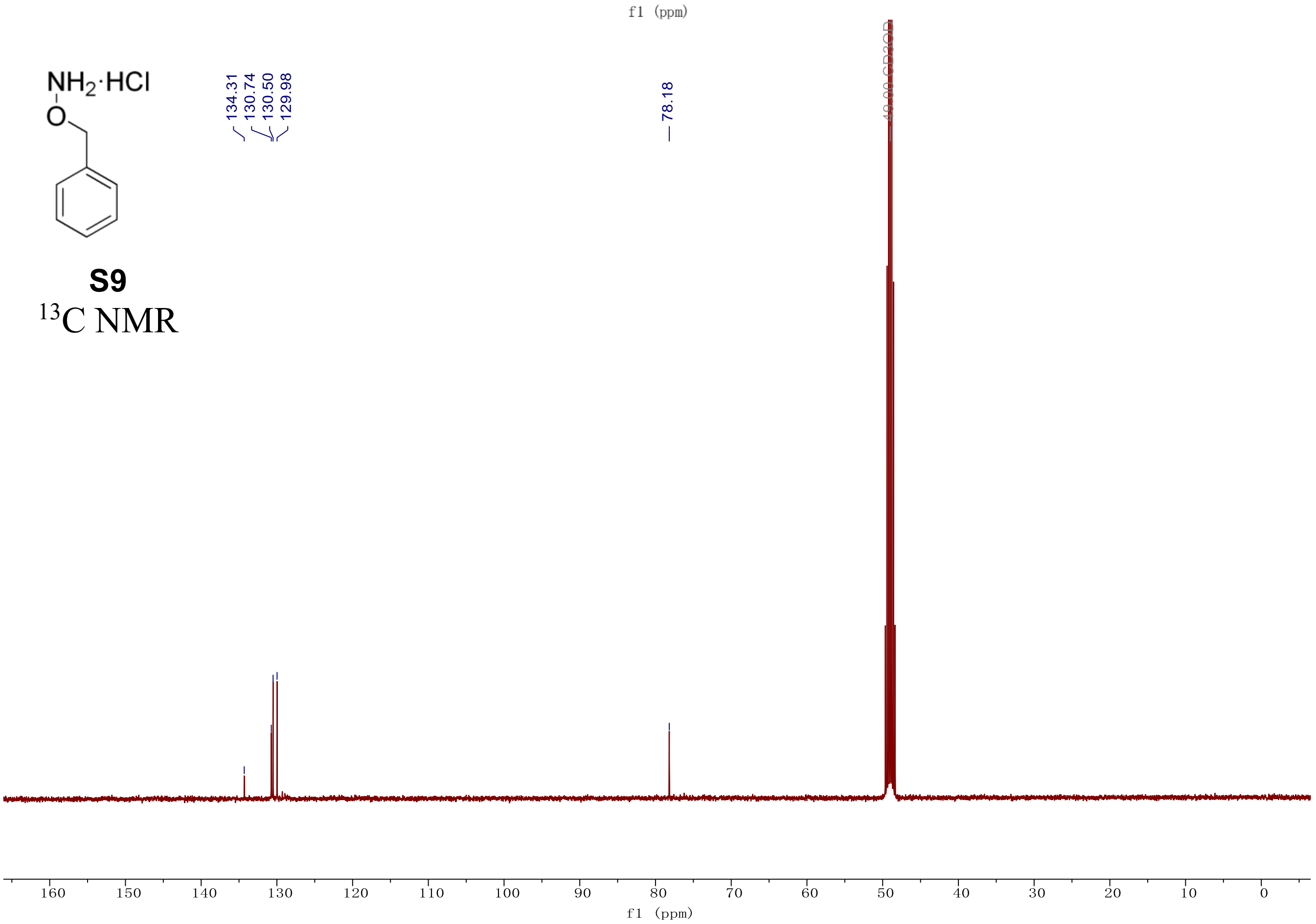

S9

^{13}C NMR

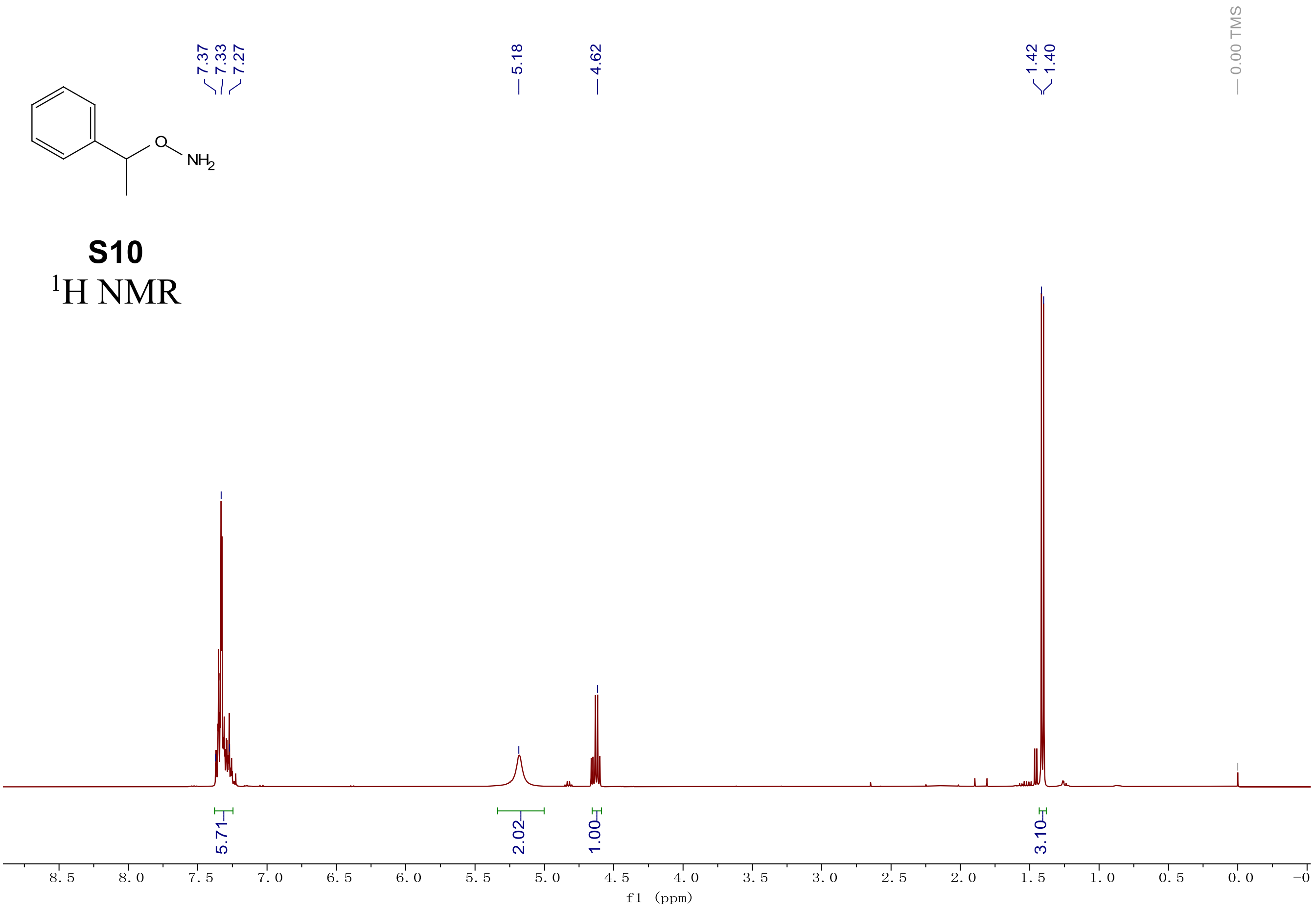

S10
^{1}H NMR

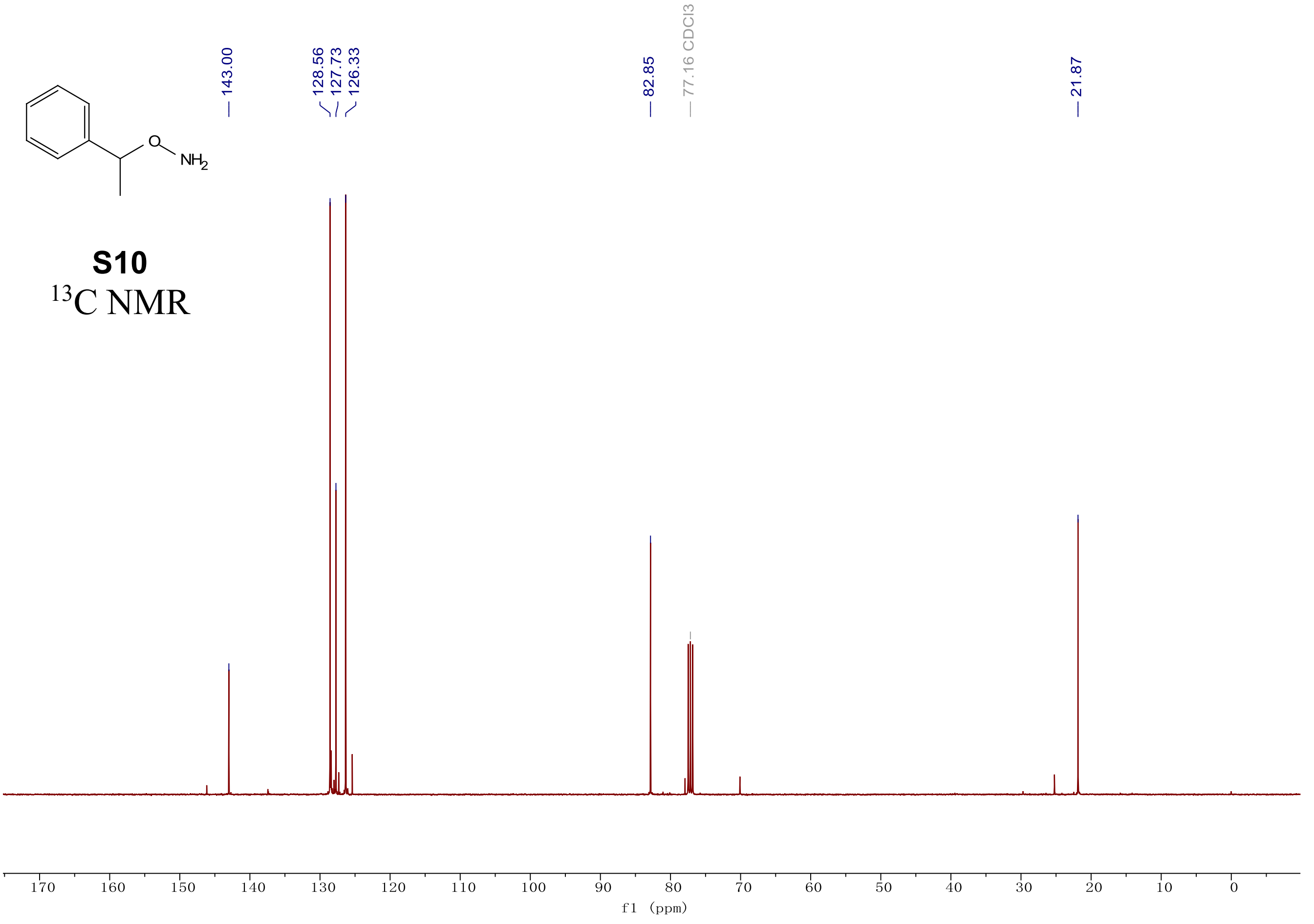

S10
^{13}C NMR

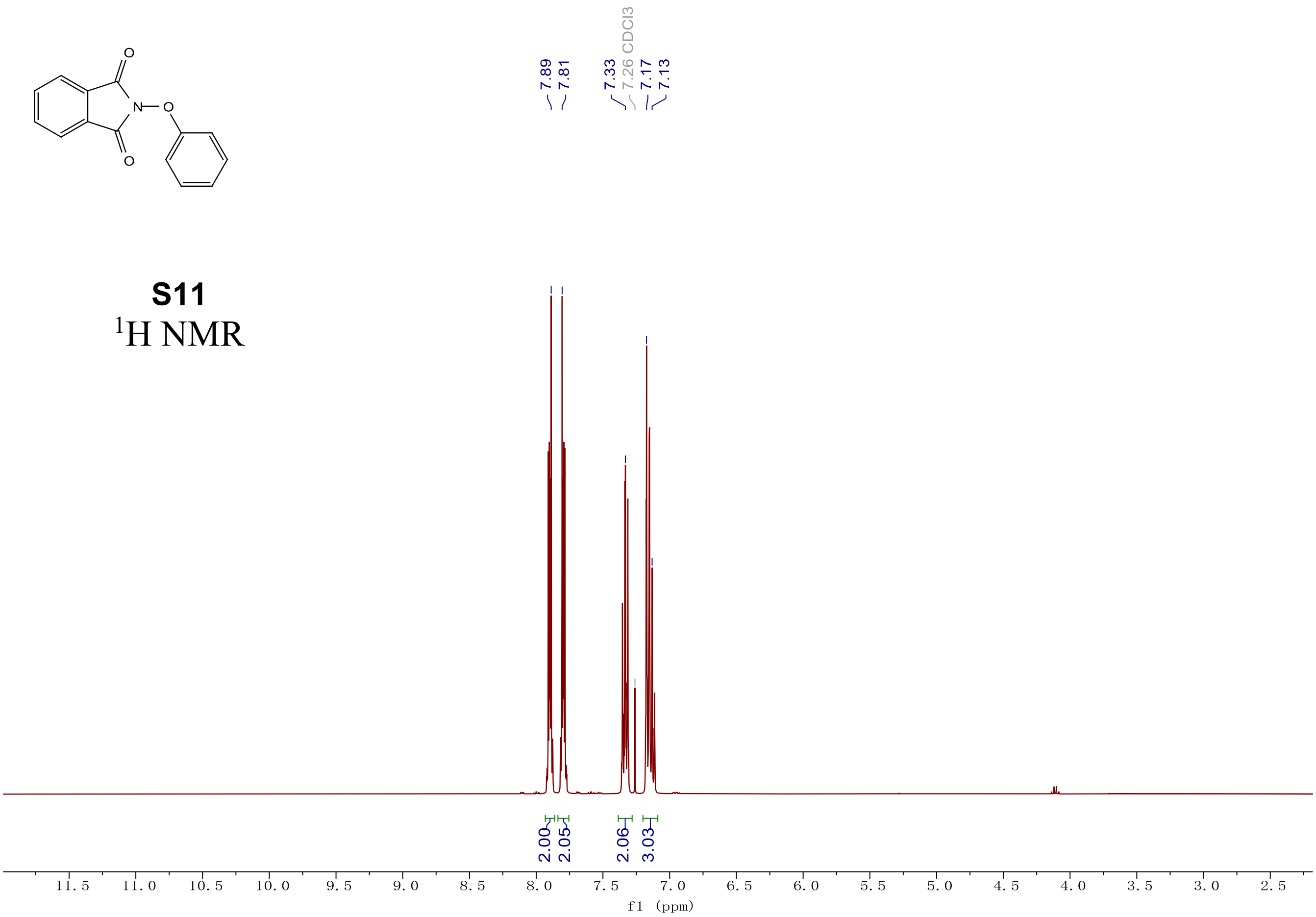

S11
^{1}H NMR
7.89
7.81
7.33
7.26 CDCl3
7.17
7.13
2.00
2.05
2.06
3.03
f1 (ppm)

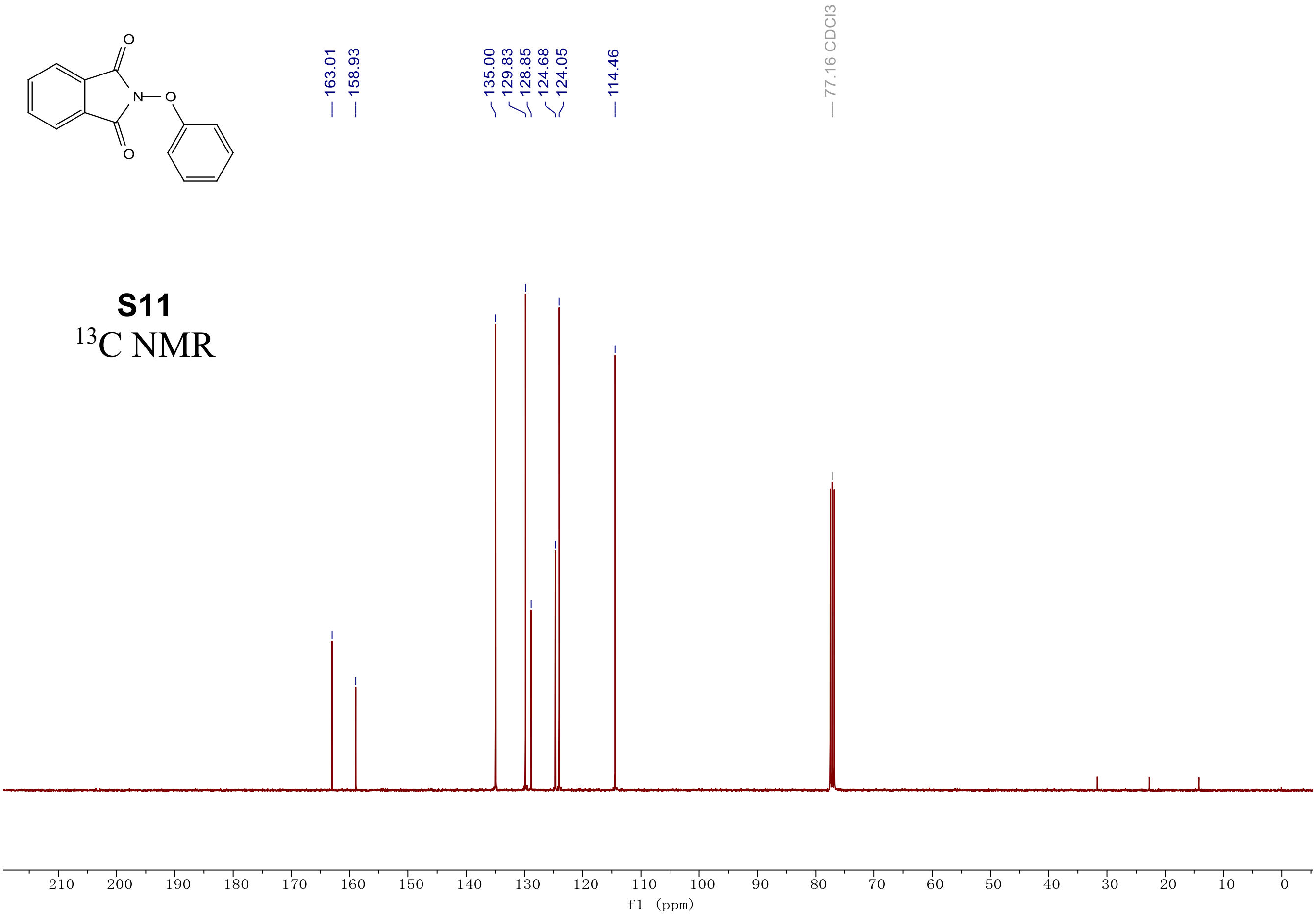

S11
^{13}C NMR
163.01
158.93
135.00
129.83
128.85
124.68
124.05
114.46
77.16 CDCl3
f1 (ppm)

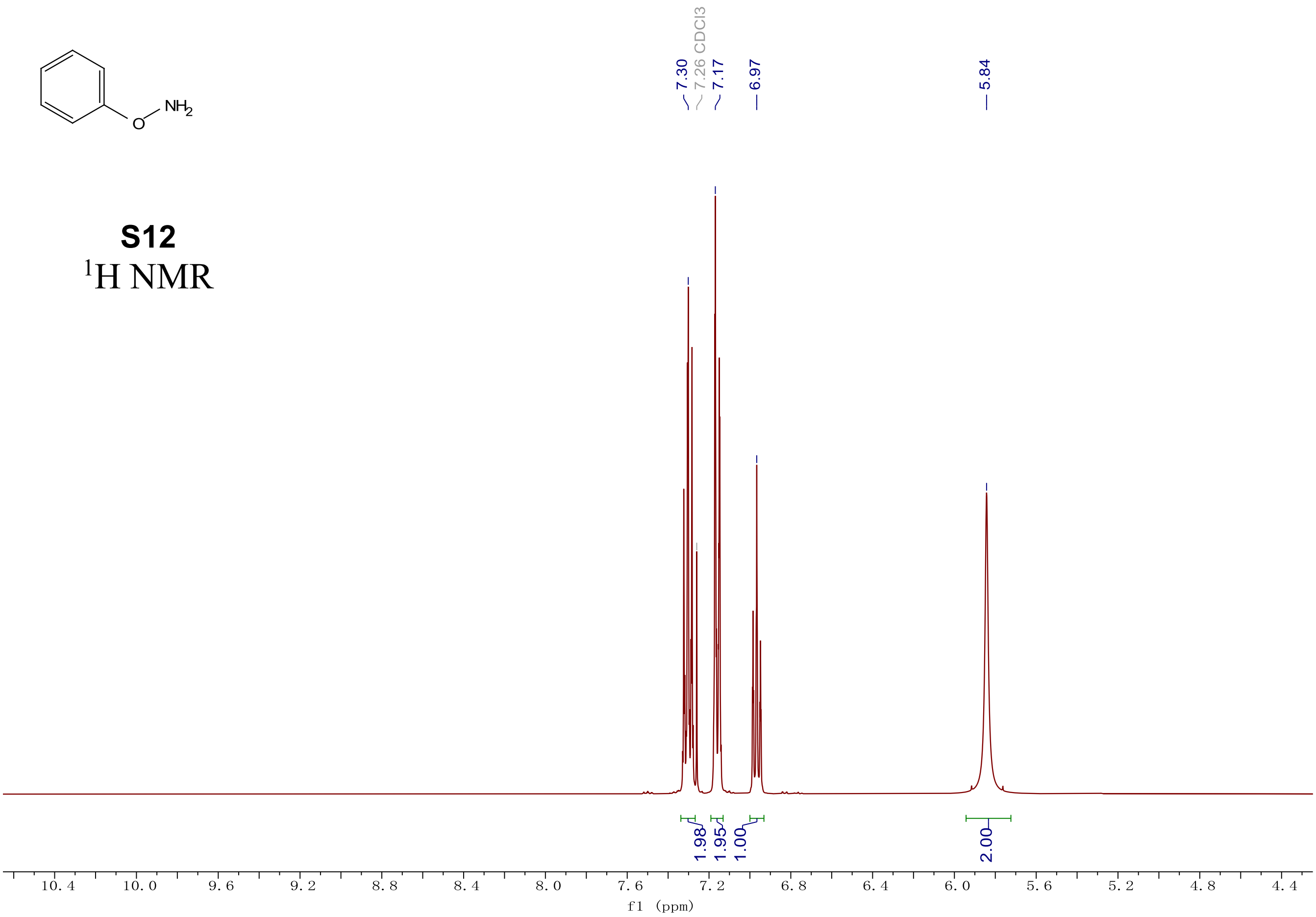
NH2
O
S12
^{1}H NMR
7.30
7.26 CDCl3
7.17
6.97
5.84
1.98
1.95
1.00
2.00
10.4 10.0 9.6 9.2 8.8 8.4 8.0 7.6 7.2 6.8 6.4 6.0 5.6 5.2 4.8 4.4
f1 (ppm)

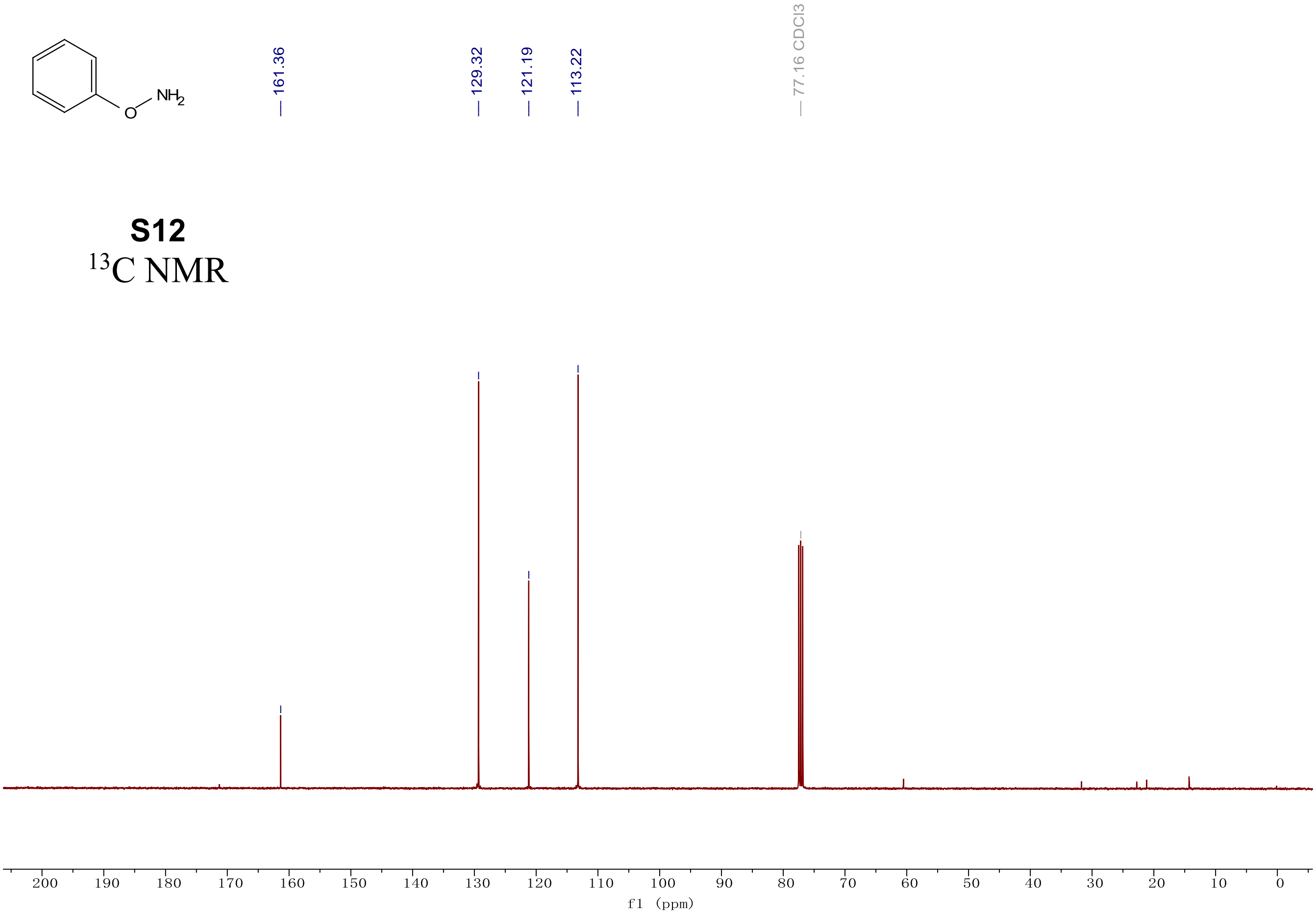
NH2
O
S12
^{13}C NMR
161.36
129.32
121.19
113.22
77.16 CDCl3
200 190 180 170 160 150 140 130 120 110 100 90 80 70 60 50 40 30 20 10 0
f1 (ppm)

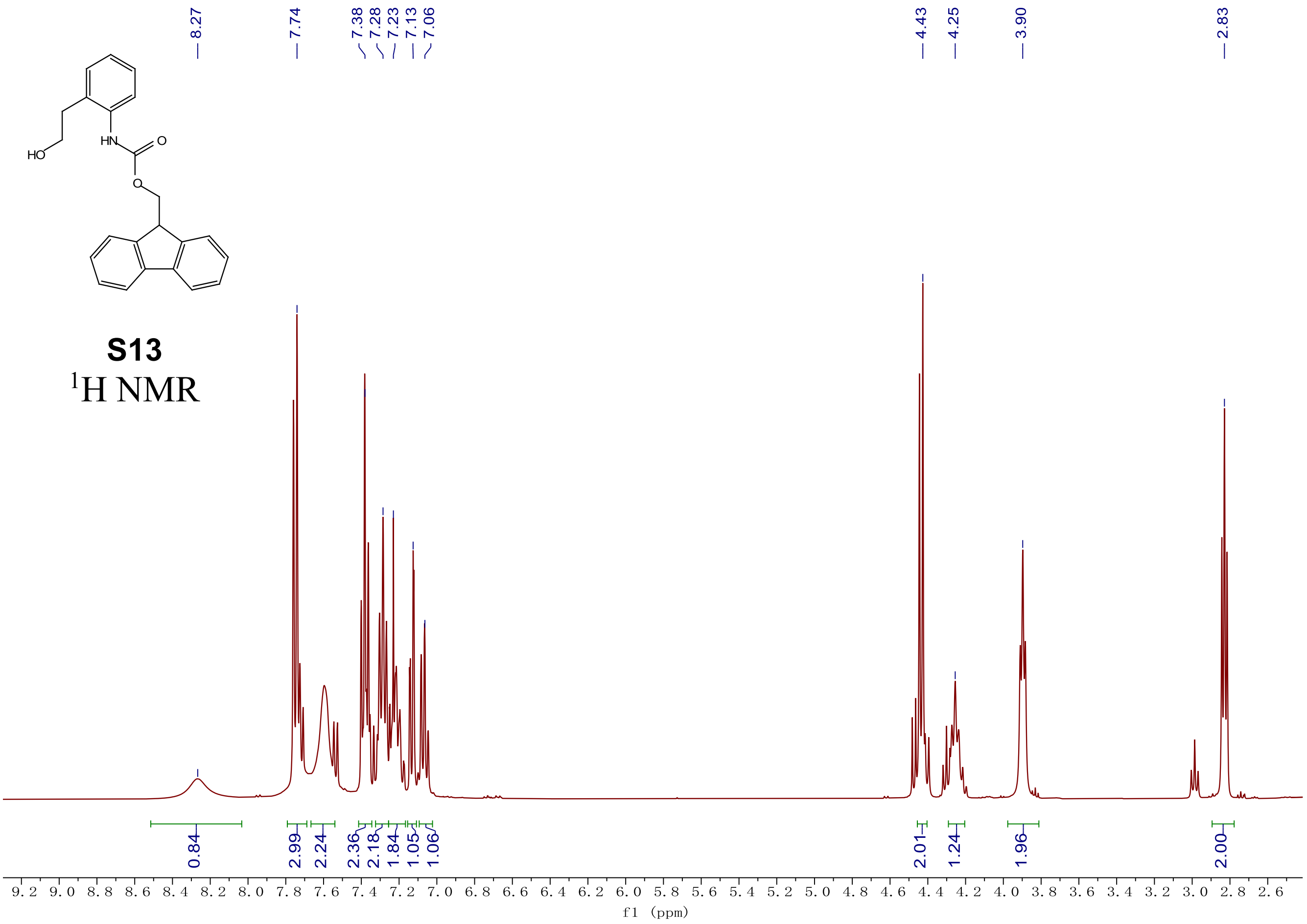
8.27
7.74
7.38
7.28
7.23
7.13
7.06
4.43
4.25
3.90
2.83
HO
HN
O
O
S13
¹H NMR
0.84
2.99
2.24
2.36
2.18
1.84
1.05
1.06
2.01
1.24
1.96
2.00
9.2 9.0 8.8 8.6 8.4 8.2 8.0 7.8 7.6 7.4 7.2 7.0 6.8 6.6 6.4 6.2 6.0 5.8 5.6 5.4 5.2 5.0 4.8 4.6 4.4 4.2 4.0 3.8 3.6 3.4 3.2 3.0 2.8 2.6
f1 (ppm)

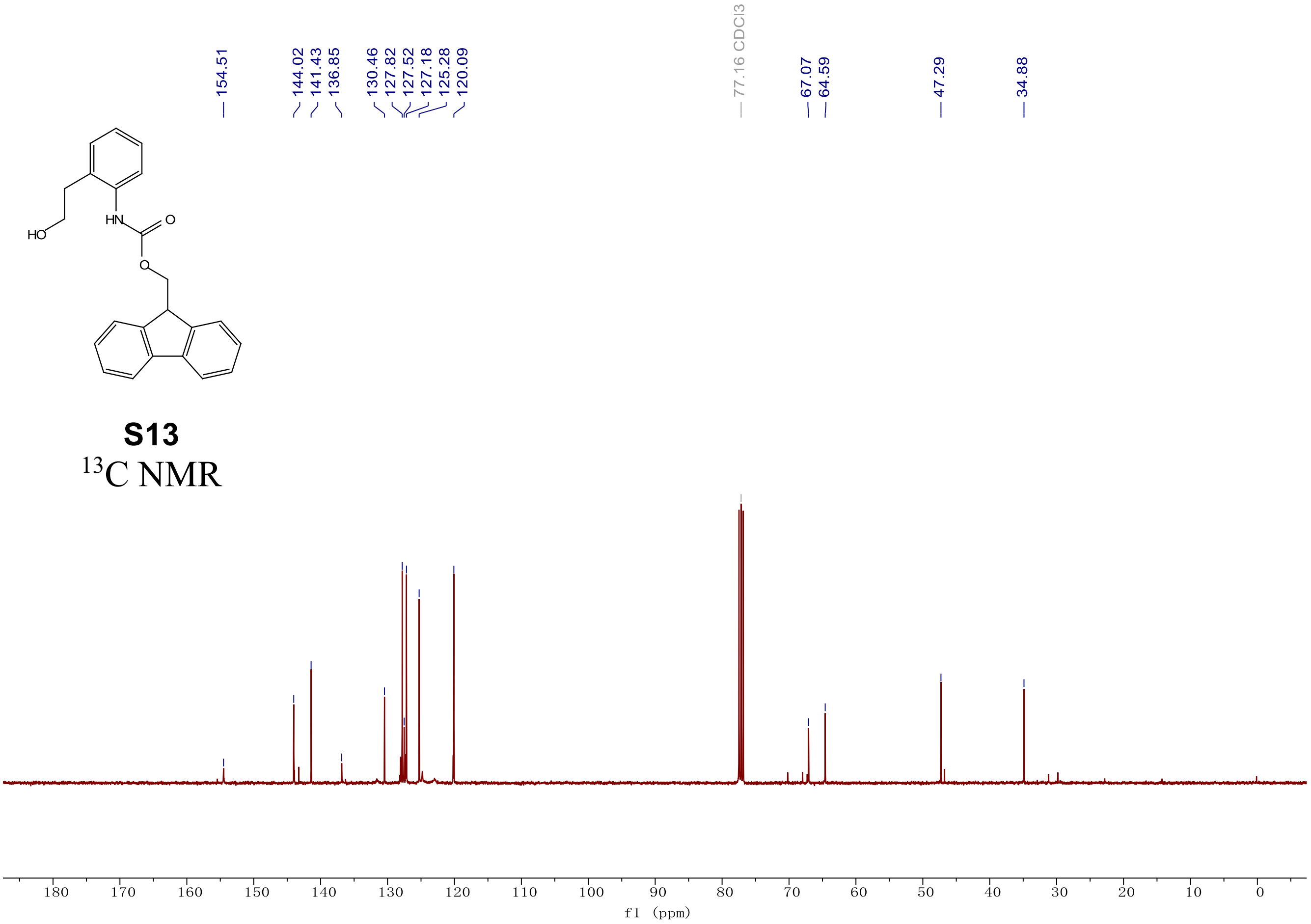
154.51
144.02
141.43
136.85
130.46
127.82
127.52
127.18
125.28
120.09
77.16 CDCl3
67.07
64.59
47.29
34.88
HO
HN
O
O
S13
¹³C NMR
180 170 160 150 140 130 120 110 100 90 80 70 60 50 40 30 20 10 0
f1 (ppm)

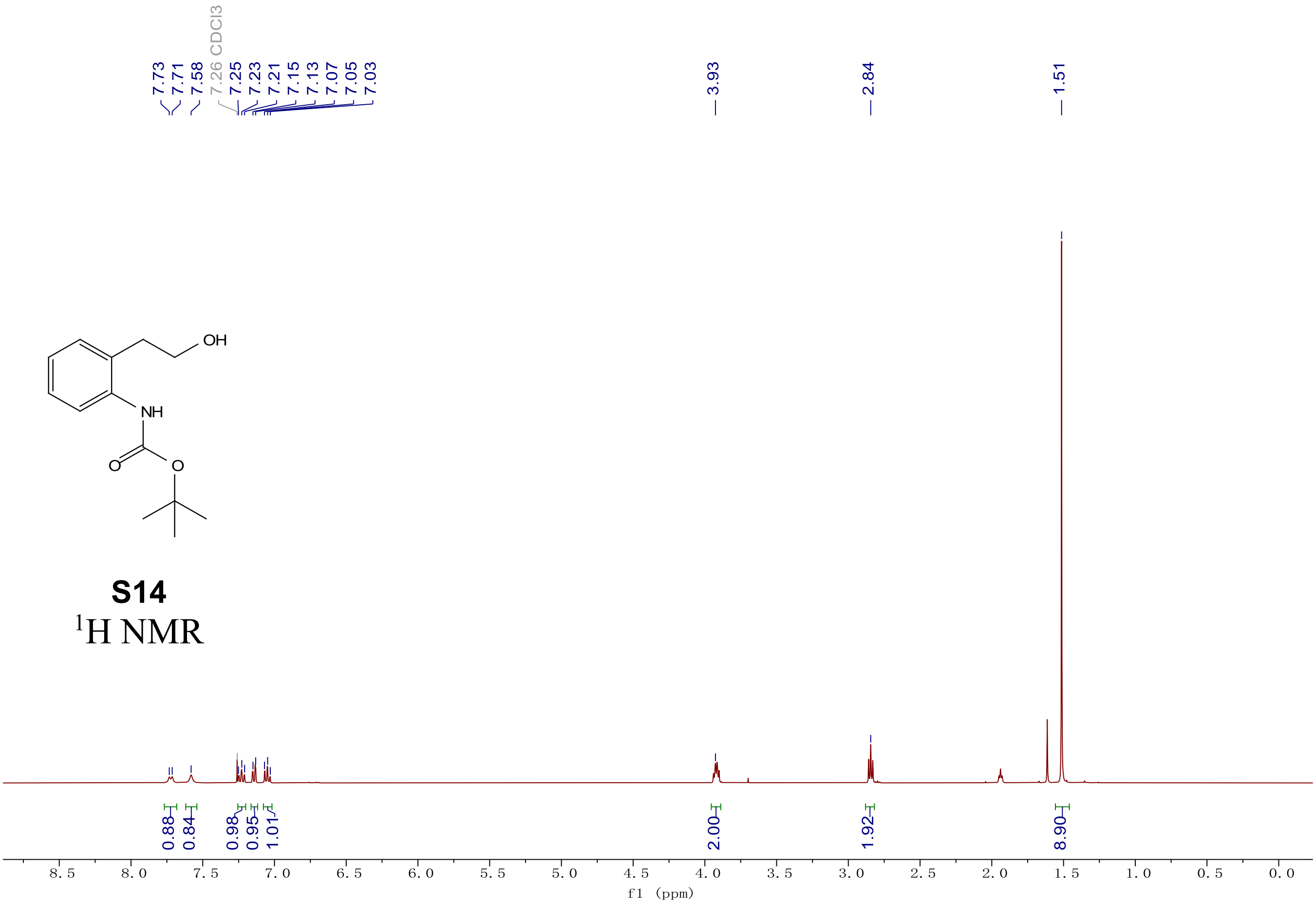

S14
^{1}H NMR

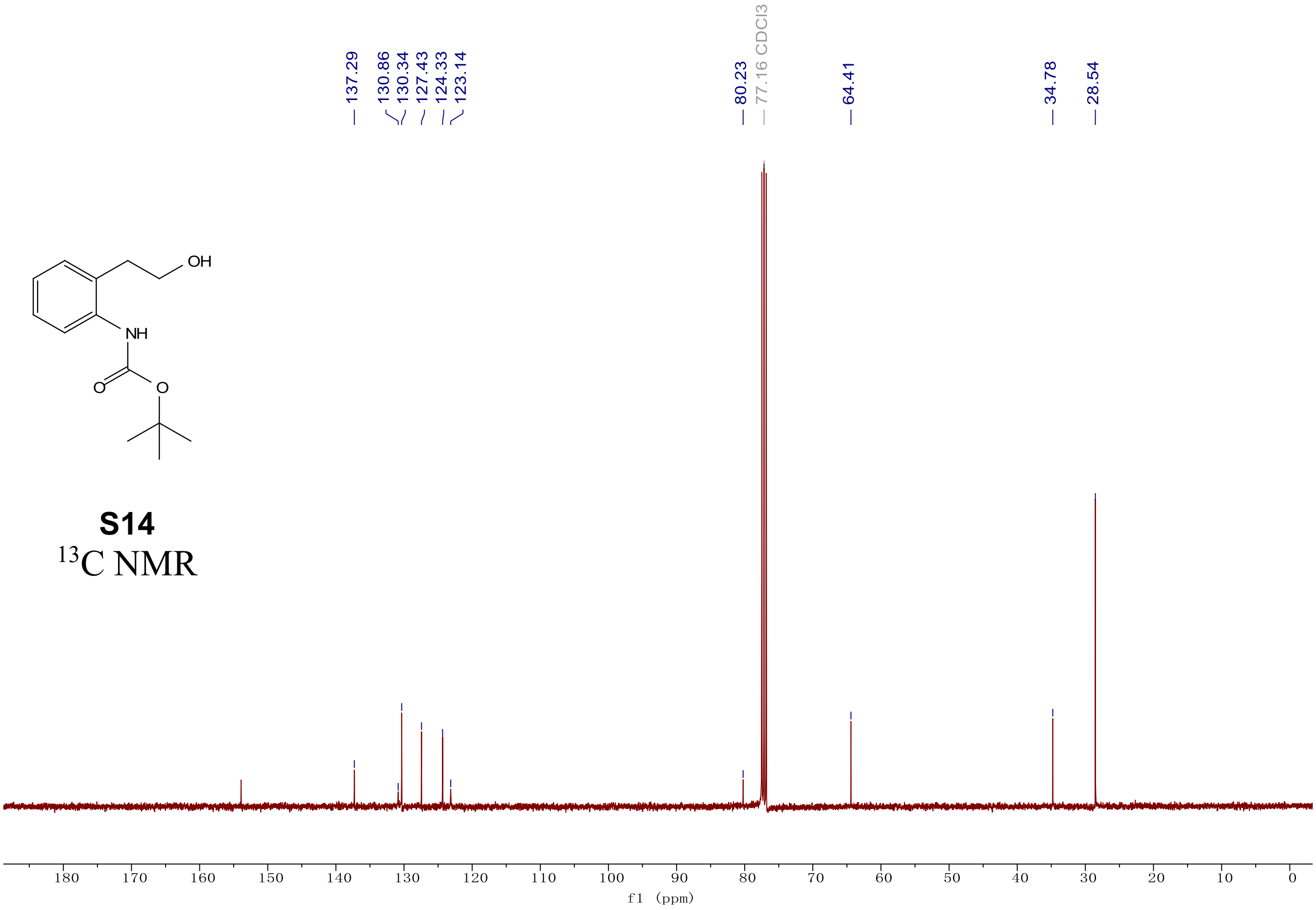

S14
^{13}C NMR

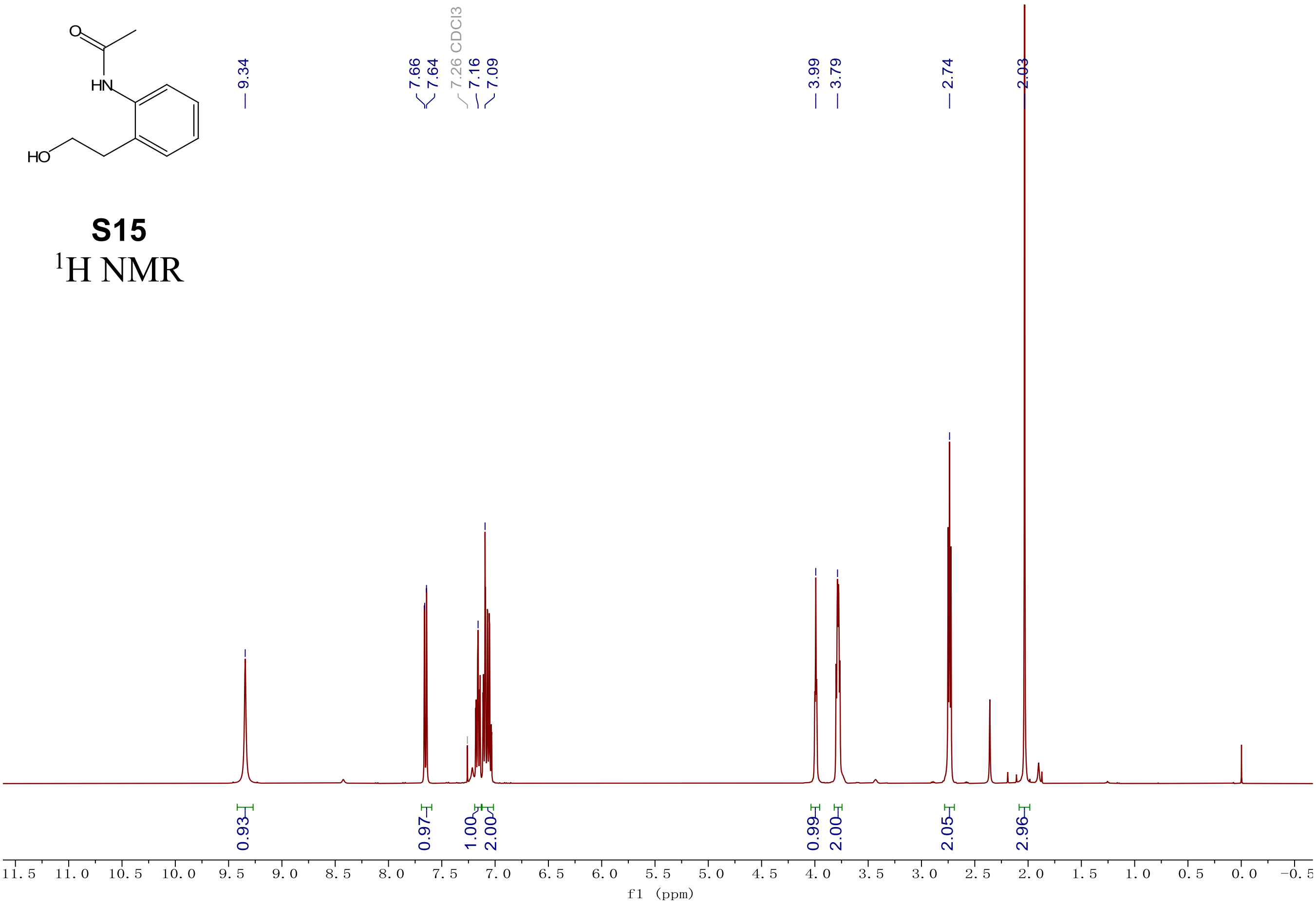

S15
^{1}H NMR

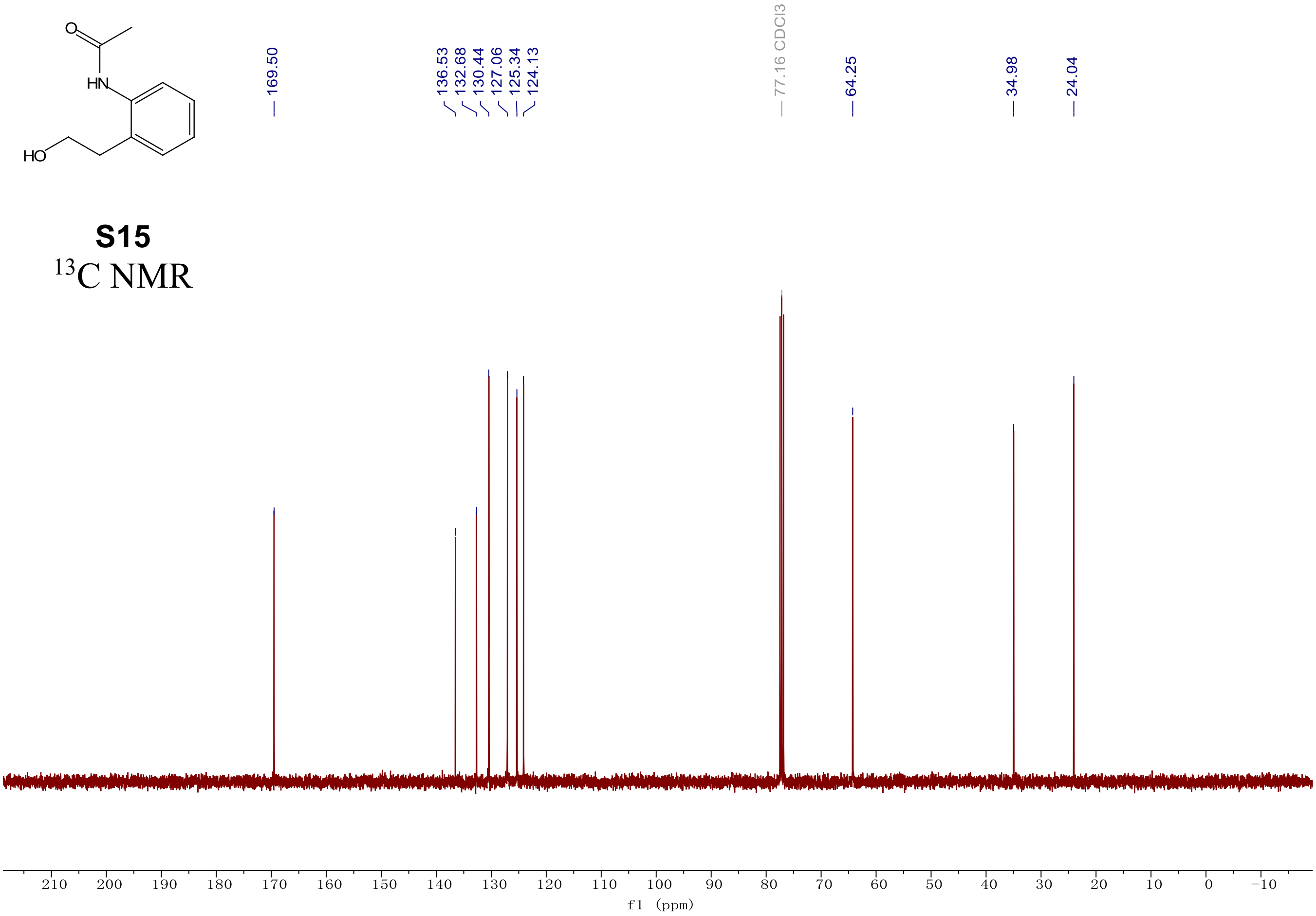

S15
^{13}C NMR

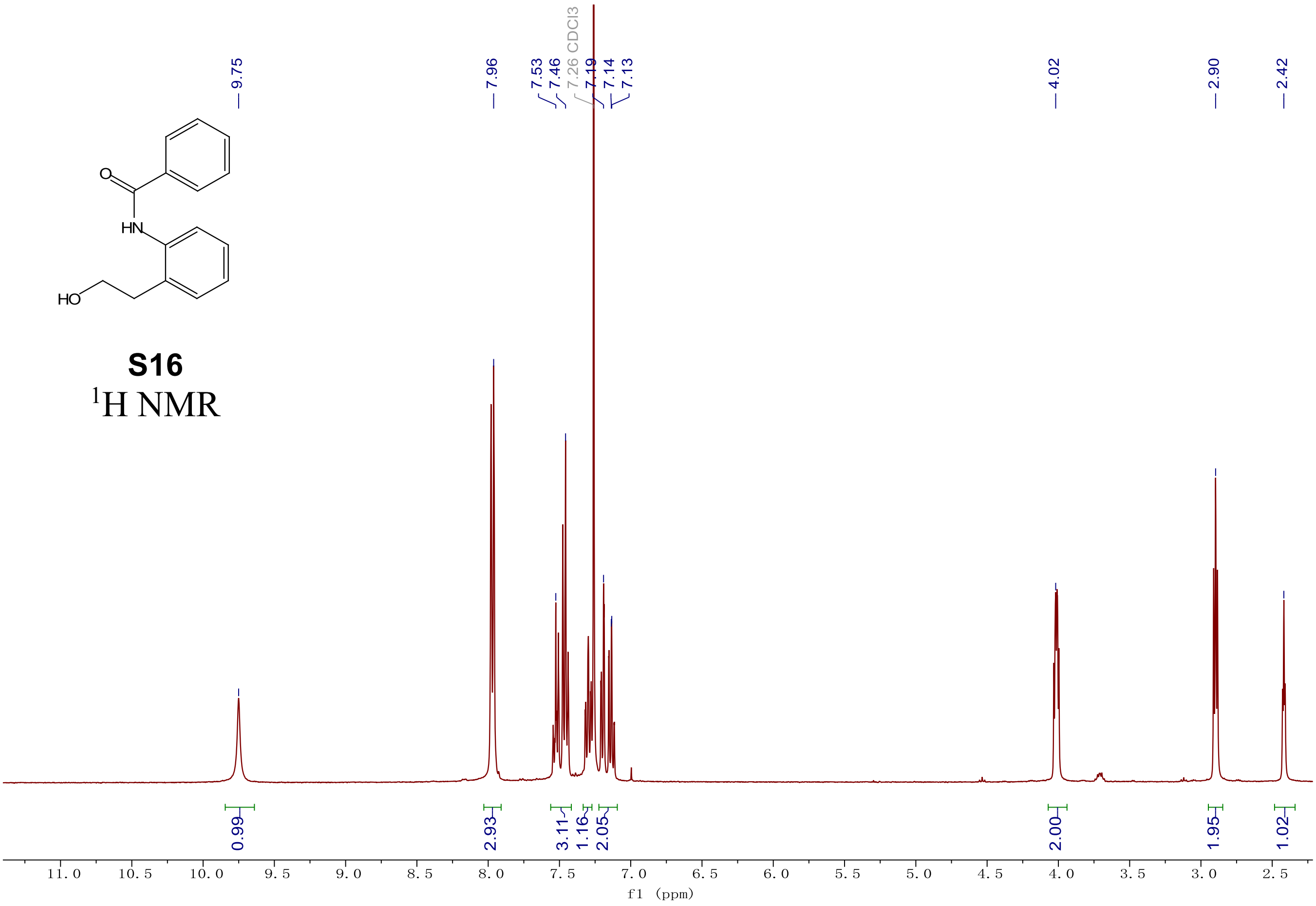

S16
^{1}H NMR

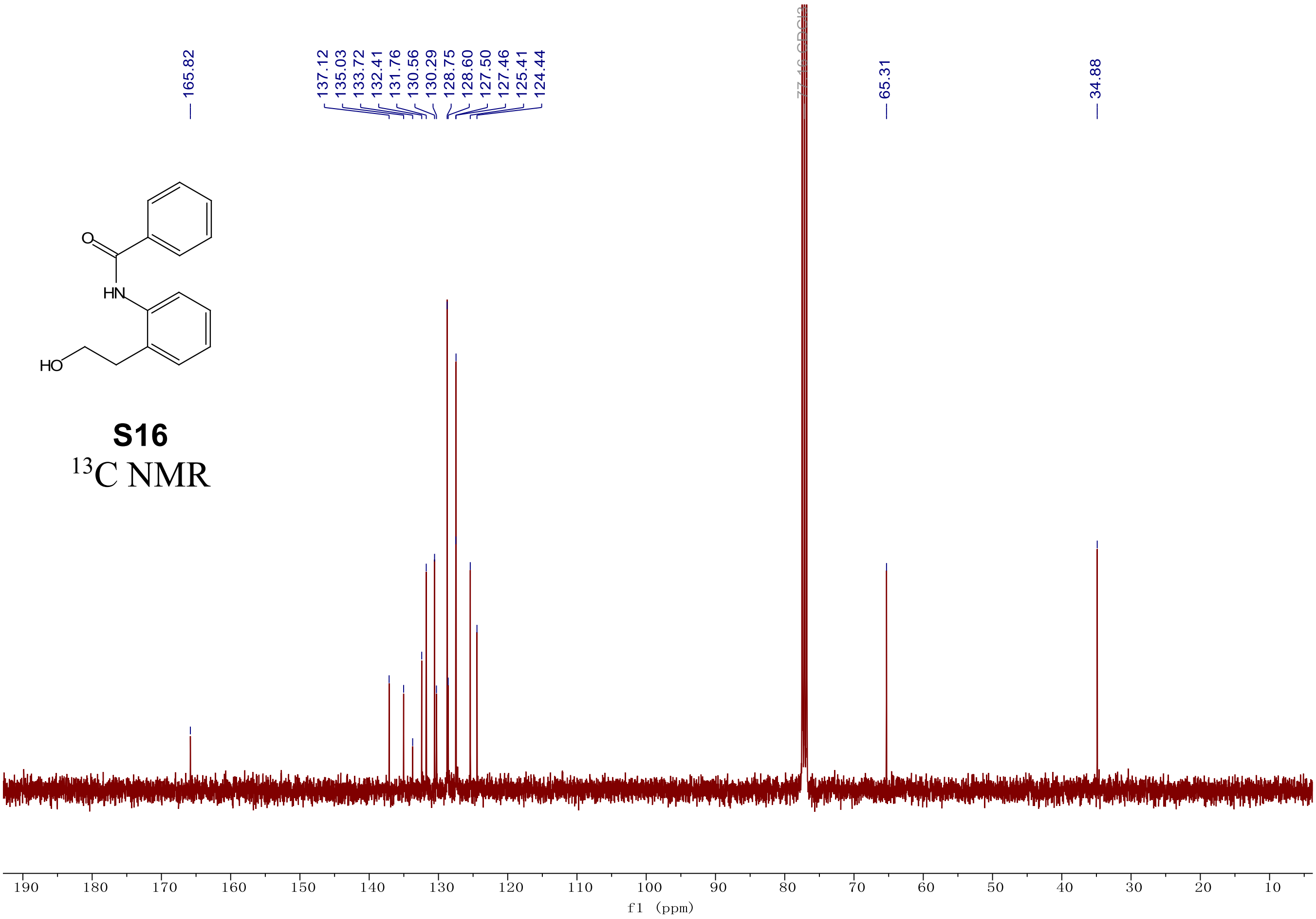

S16
^{13}C NMR

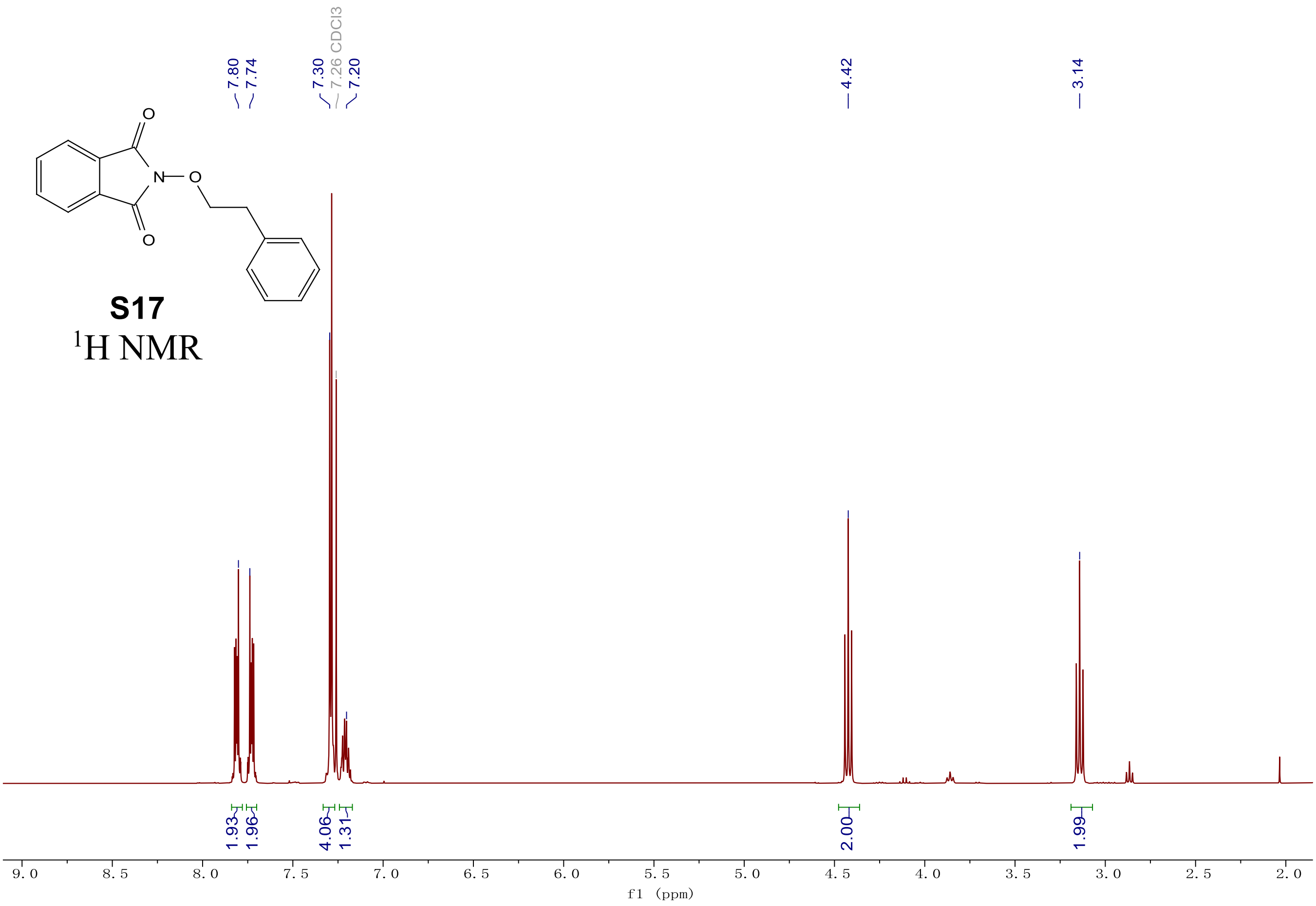

S17
^{1}H NMR
7.80
7.74
7.30
7.26 CDCl3
7.20
4.42
3.14
1.93
1.96
4.06
1.31
2.00
1.99
9.0 8.5 8.0 7.5 7.0 6.5 6.0 5.5 5.0 4.5 4.0 3.5 3.0 2.5 2.0
f1 (ppm)

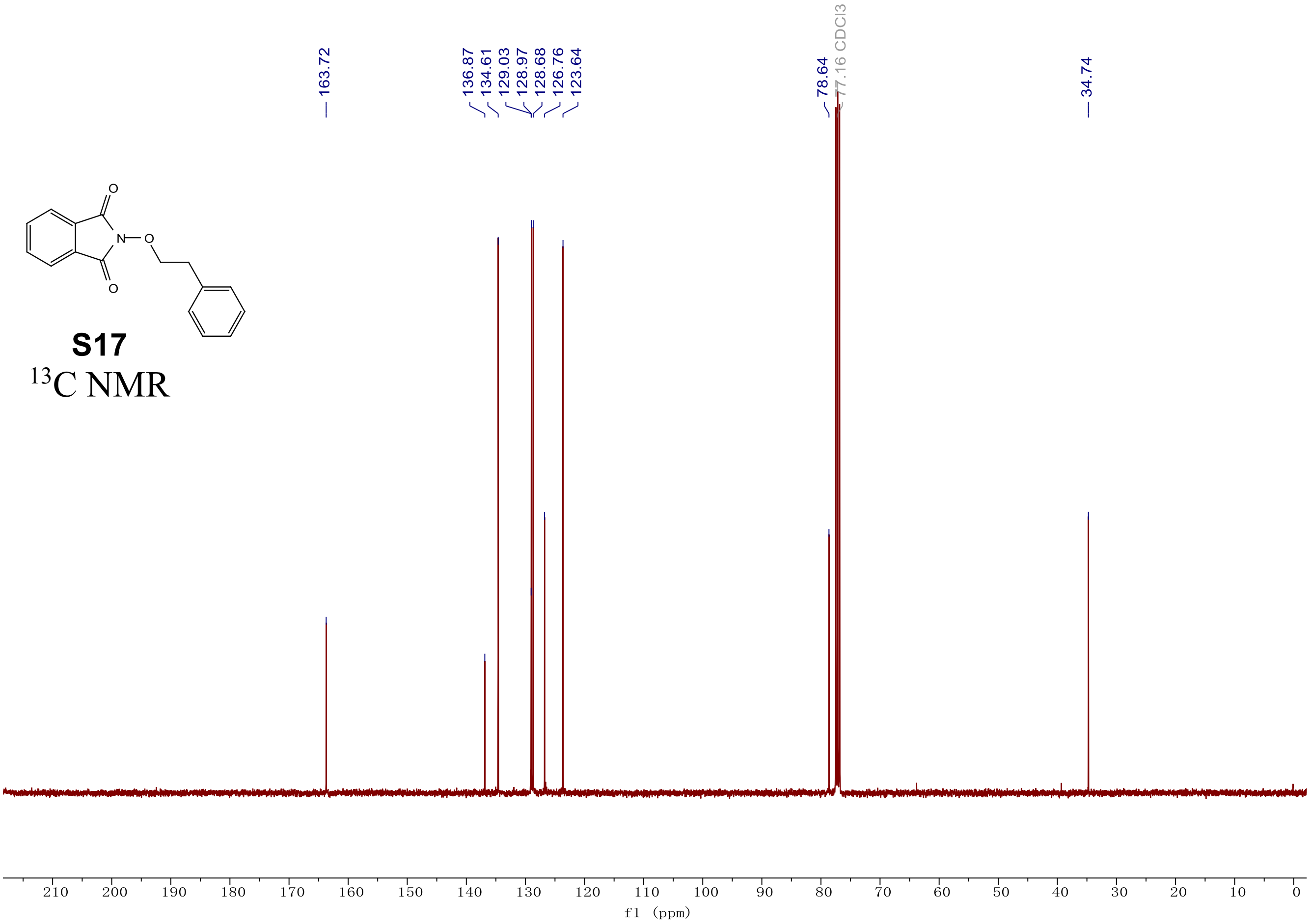

S17
^{13}C NMR
163.72
136.87
134.61
129.03
128.97
128.68
126.76
123.64
78.64
77.16 CDCl3
34.74
210 200 190 180 170 160 150 140 130 120 110 100 90 80 70 60 50 40 30 20 10 0
f1 (ppm)

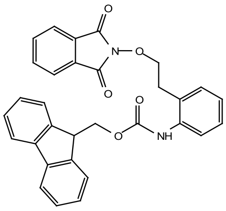

S18
^{1}H NMR

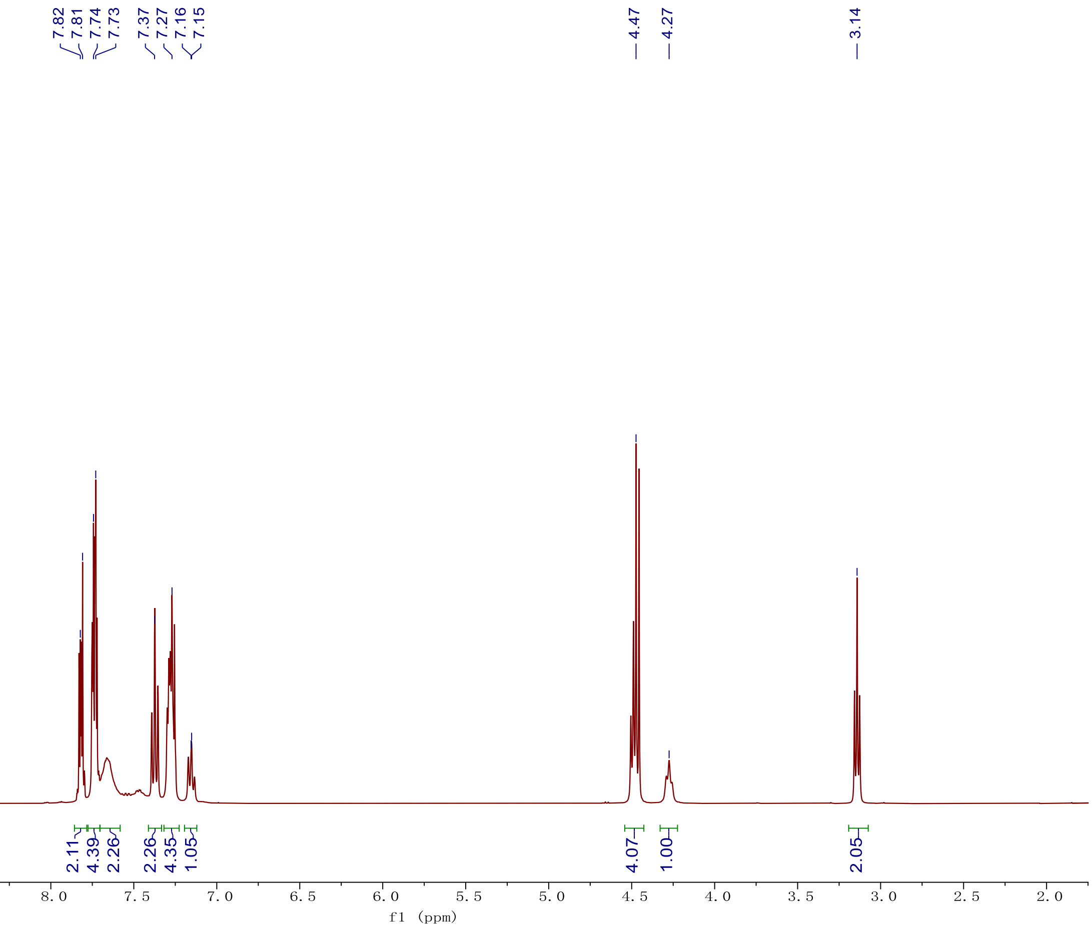

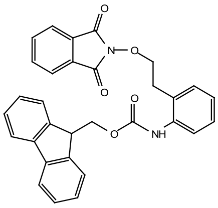

S18
^{13}C NMR

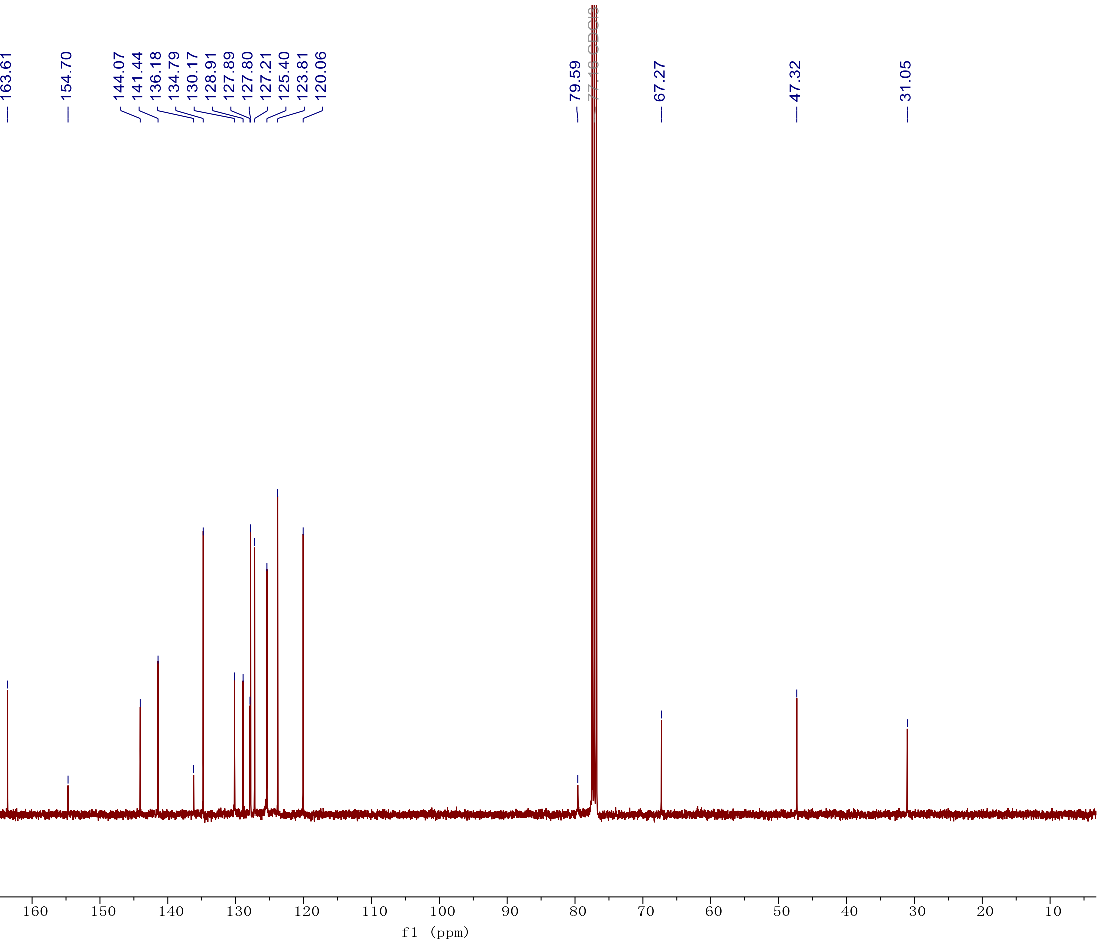

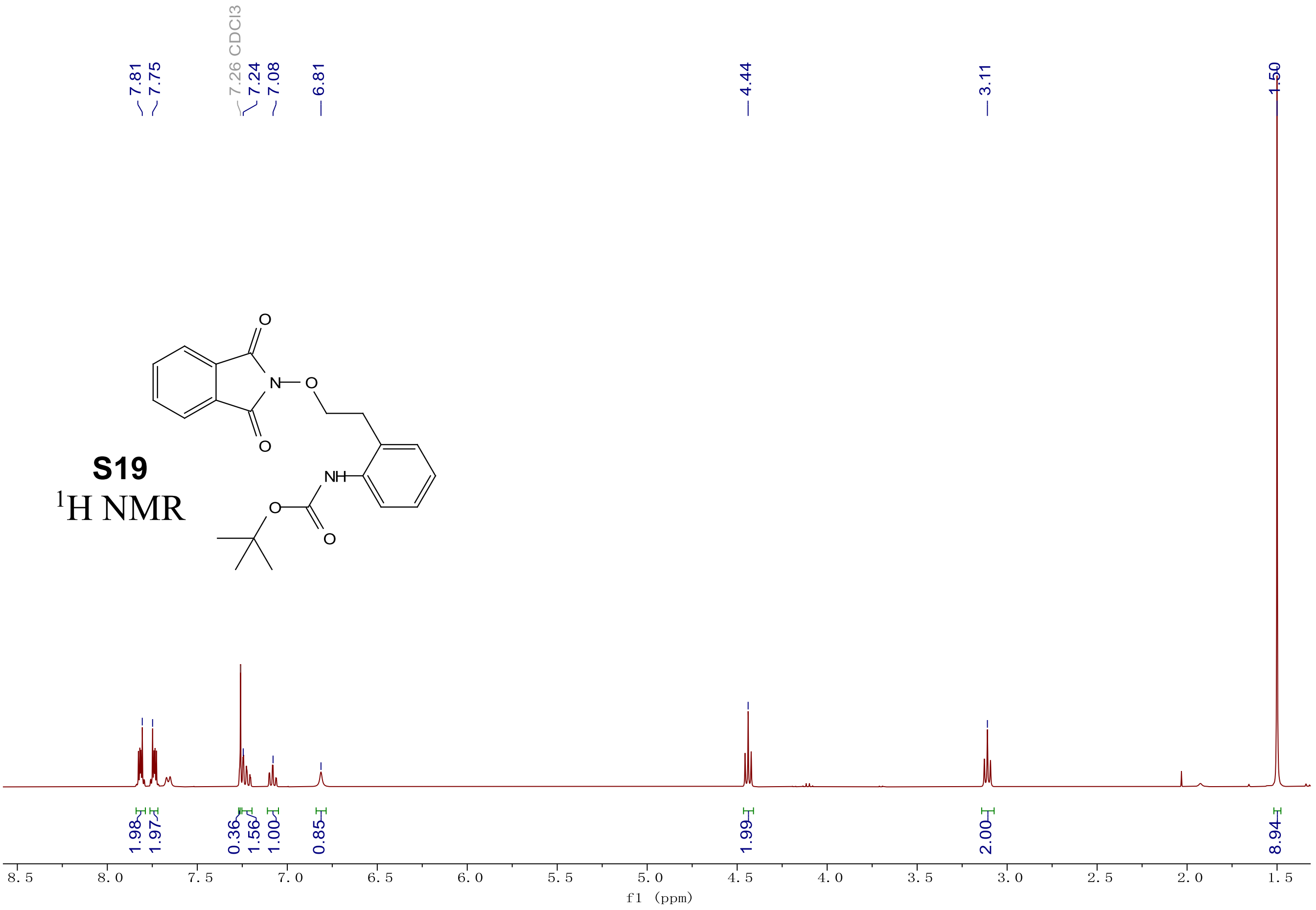
7.81
7.75
7.26 CDCl3
7.24
7.08
6.81
4.44
3.11
1.50
S19
1H NMR
1.98
1.97
0.36
1.56
1.00
0.85
1.99
2.00
8.94
f1 (ppm)

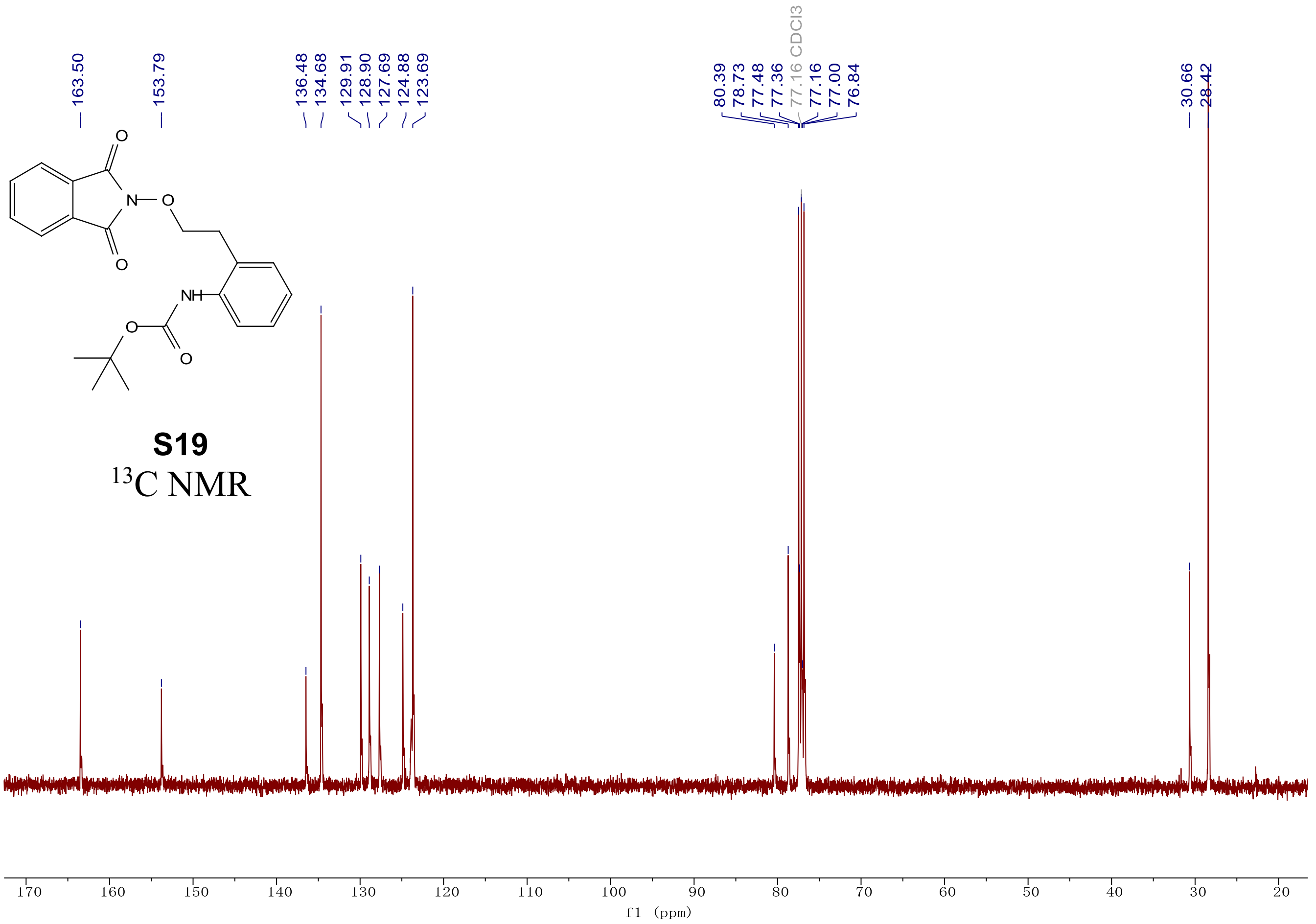
163.50
153.79
136.48
134.68
129.91
128.90
127.69
124.88
123.69
80.39
78.73
77.48
77.36
77.16 CDCl3
77.16
77.00
76.84
30.66
28.42
S19
13C NMR
f1 (ppm)

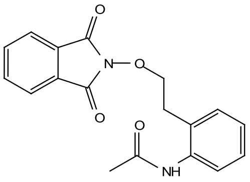

S20

^{1}H NMR

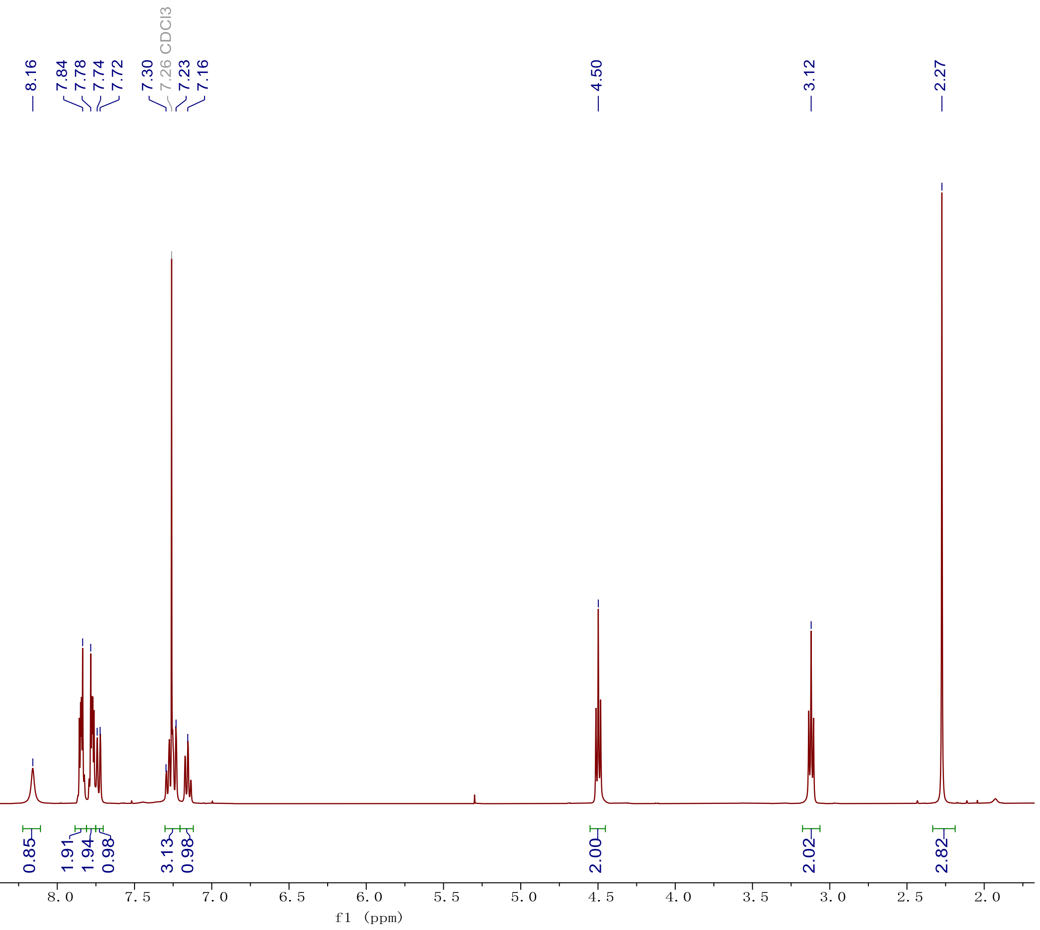

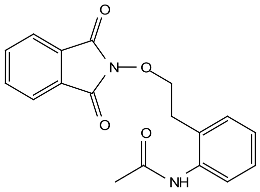

S20

^{13}C NMR

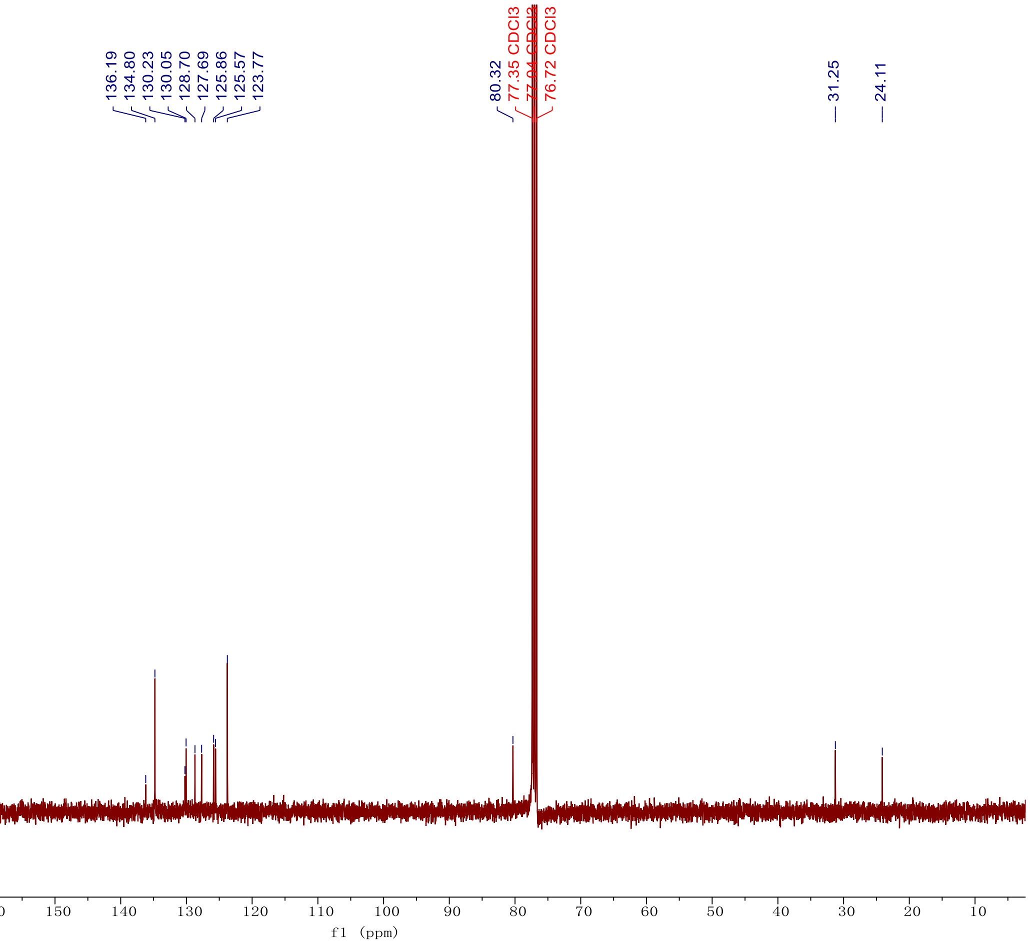

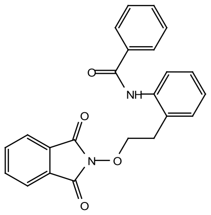

S21

^{1}H NMR

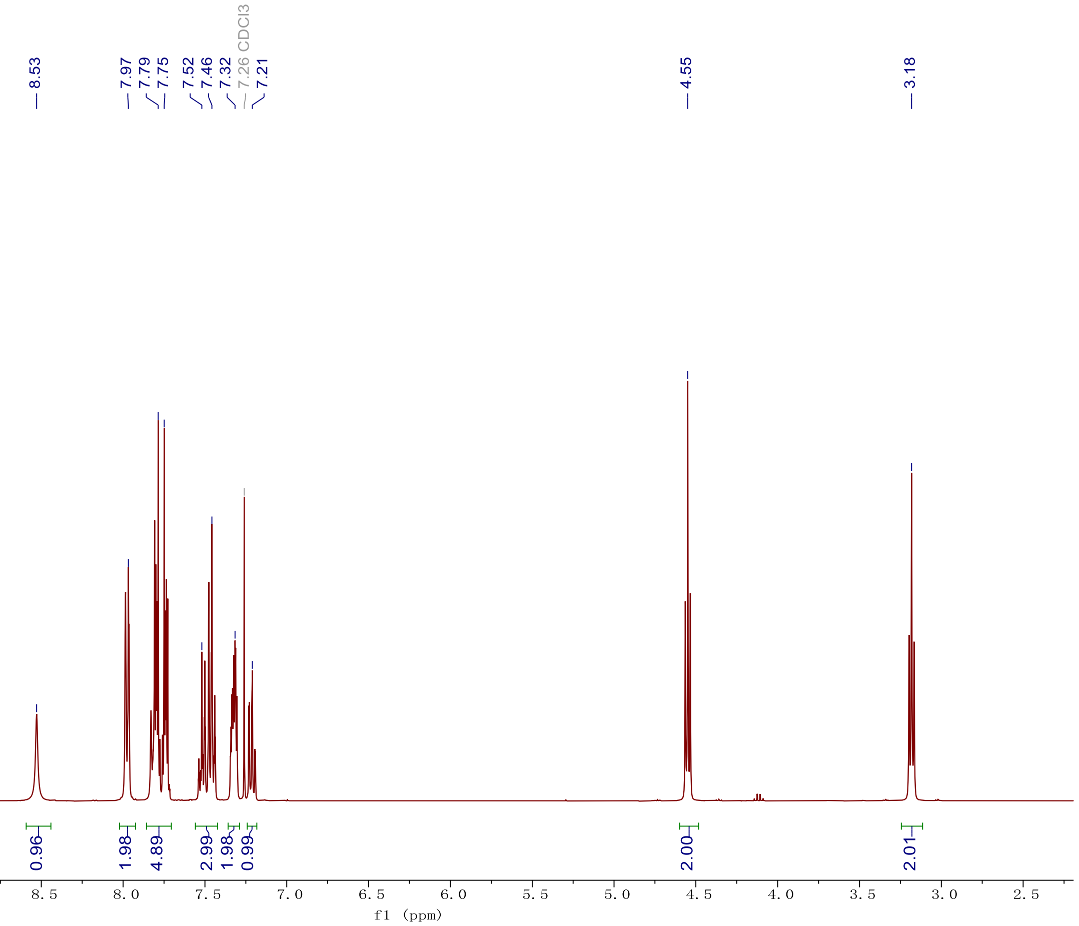

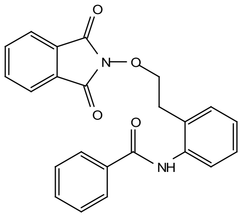

S21

^{13}C NMR

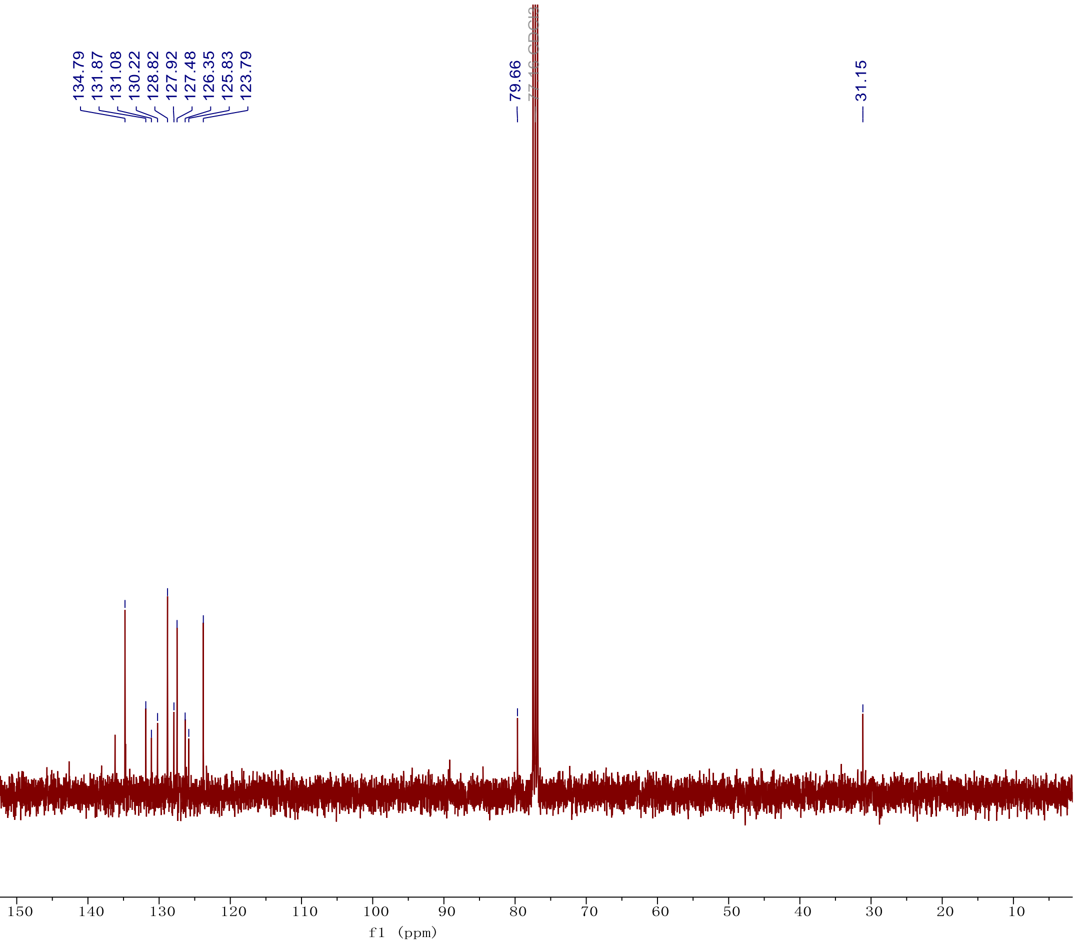

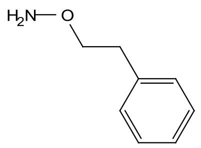

S22
^{1}H NMR

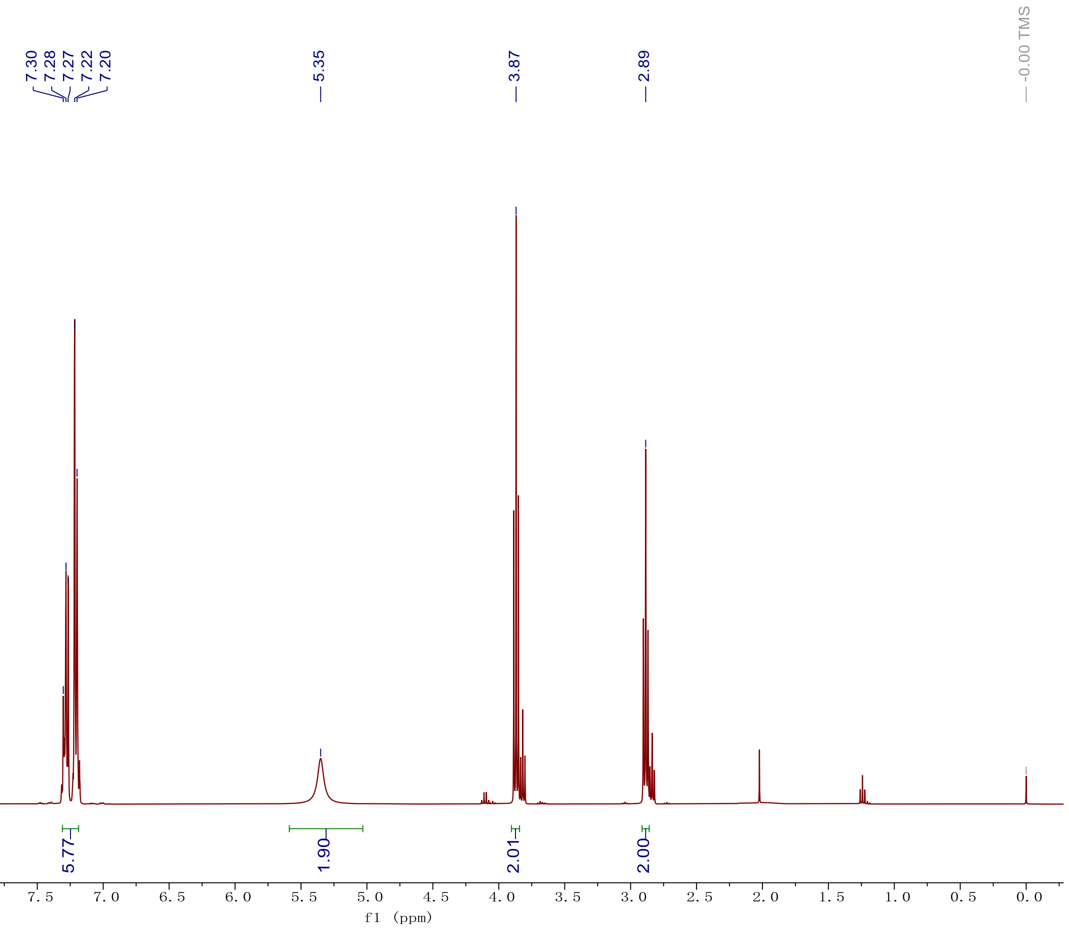

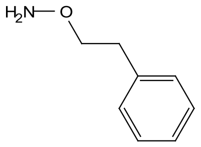

S22
^{13}C NMR

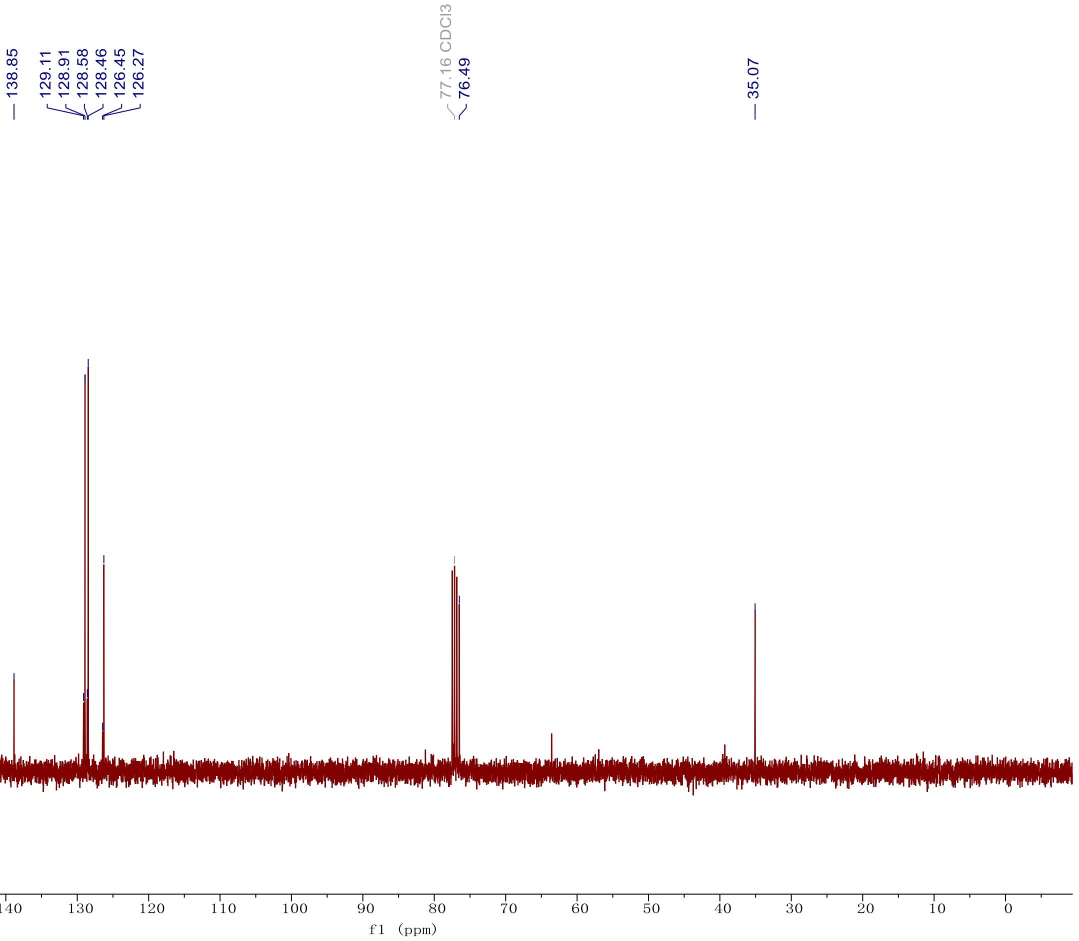

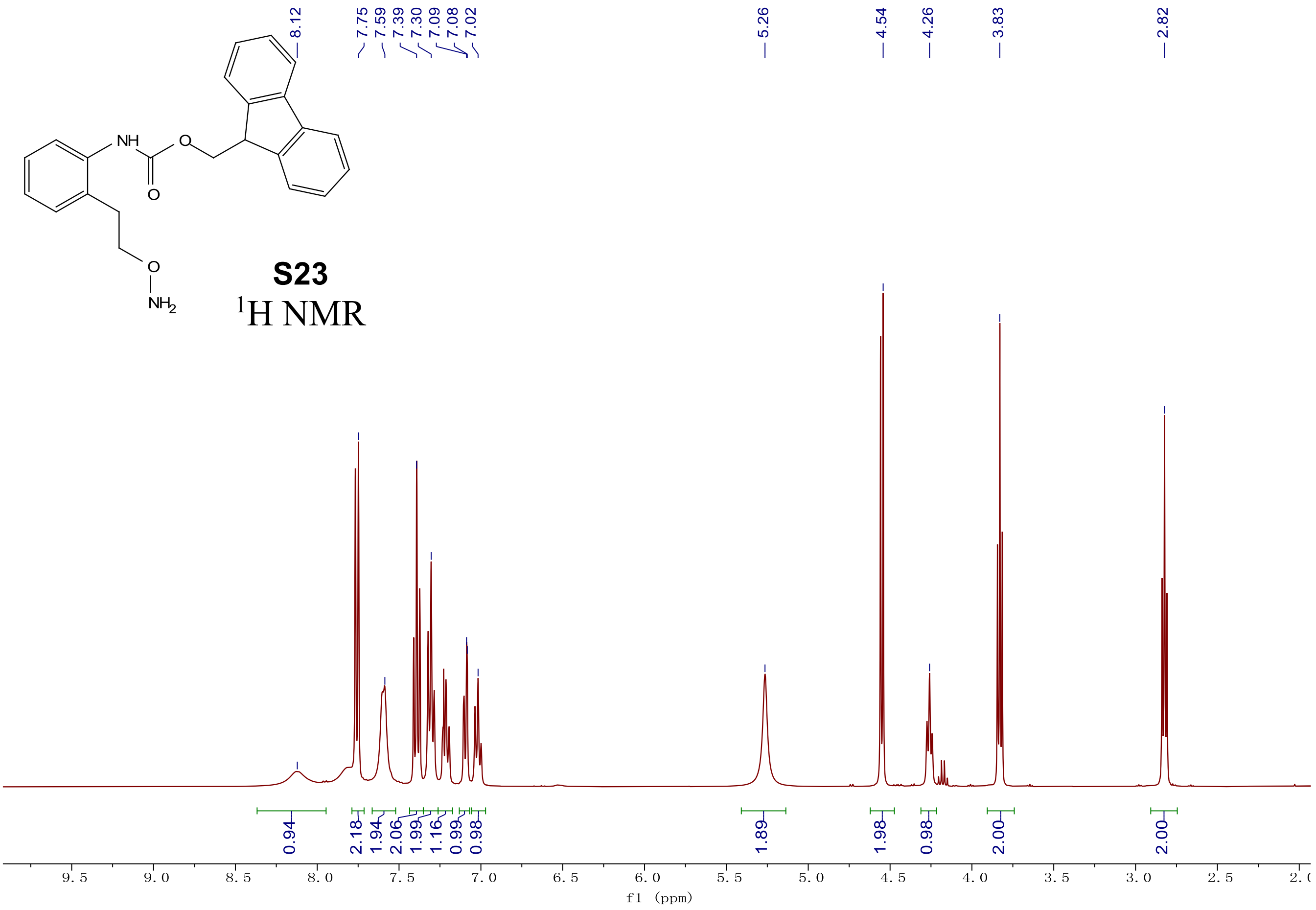

S23
^{1}H NMR

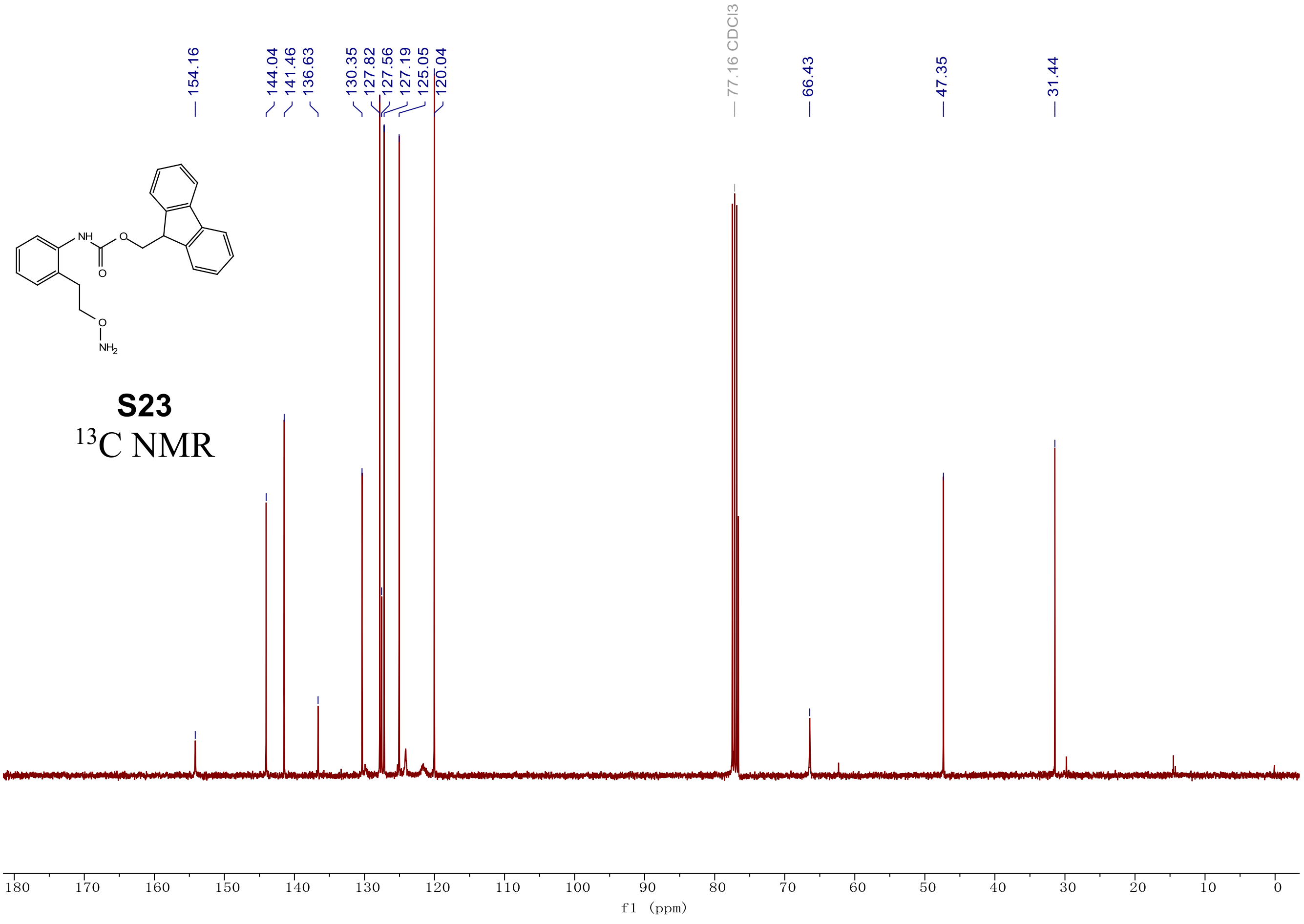

S23
^{13}C NMR

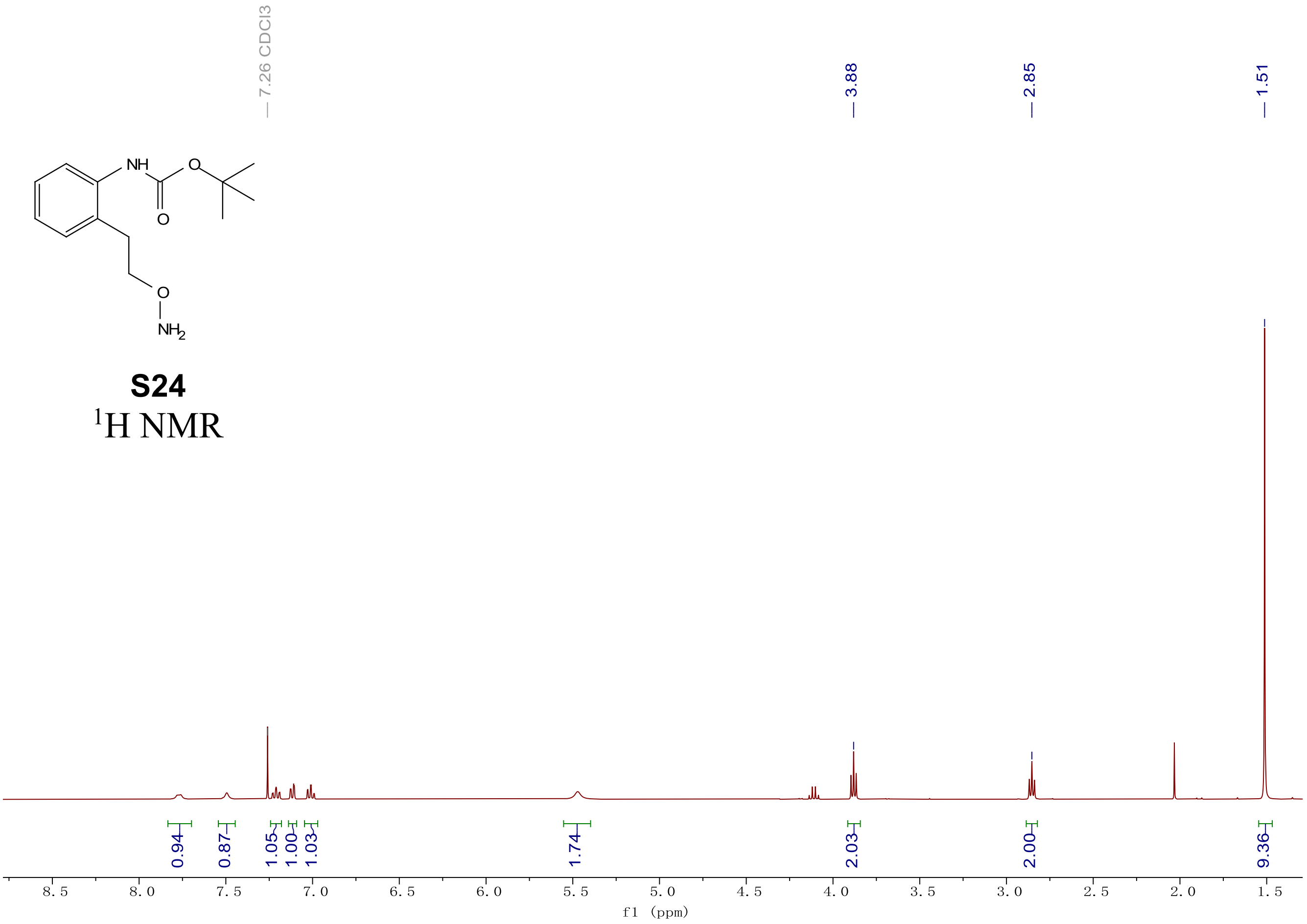

7.26 CDCl3
3.88
2.85
1.51
NH
O
O
O
NH2
S24
1H NMR
0.94
0.87
1.05
1.00
1.03
1.74
2.03
2.00
9.36
8.5
8.0
7.5
7.0
6.5
6.0
5.5
5.0
4.5
4.0
3.5
3.0
2.5
2.0
1.5
f1 (ppm)

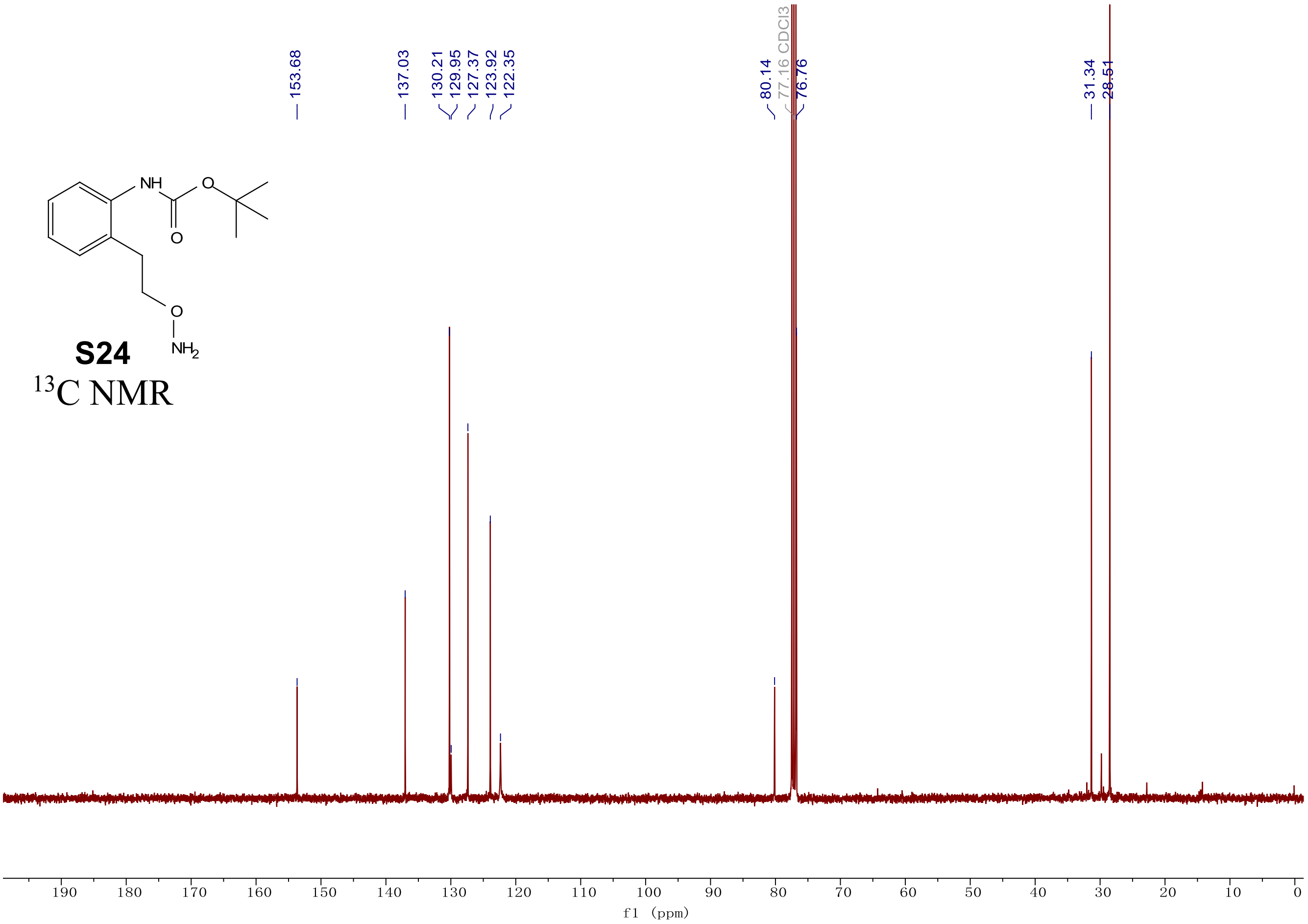

153.68
137.03
130.21
129.95
127.37
123.92
122.35
80.14
77.16 CDCl3
76.76
31.34
28.51
NH
O
O
O
NH2
S24
13C NMR
190
180
170
160
150
140
130
120
110
100
90
80
70
60
50
40
30
20
10
0
f1 (ppm)

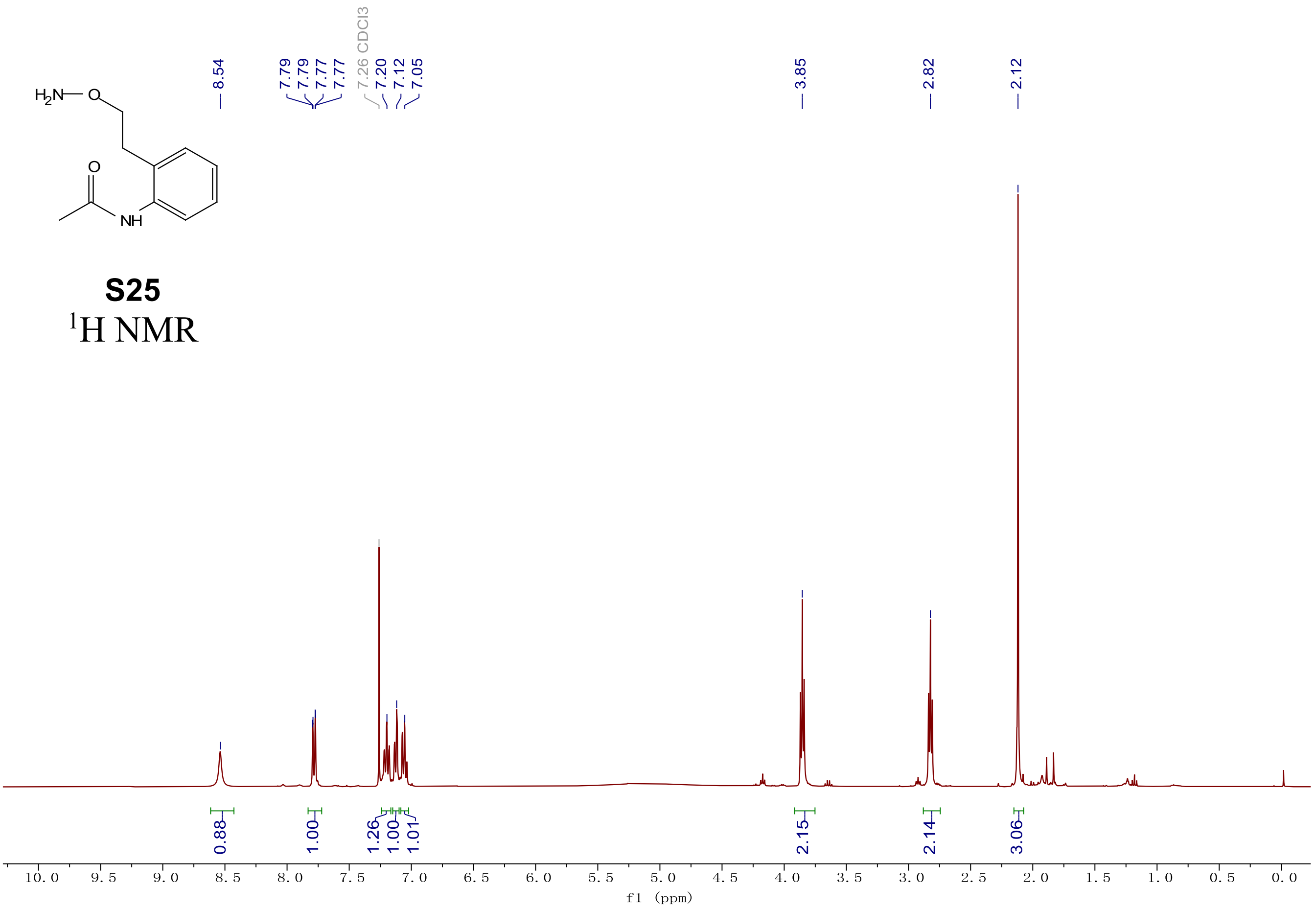
S25
1H NMR
8.54
7.79
7.79
7.77
7.77
7.26 CDCl3
7.20
7.12
7.05
3.85
2.82
2.12
0.88
1.00
1.26
1.00
1.01
2.15
2.14
3.06
f1 (ppm)

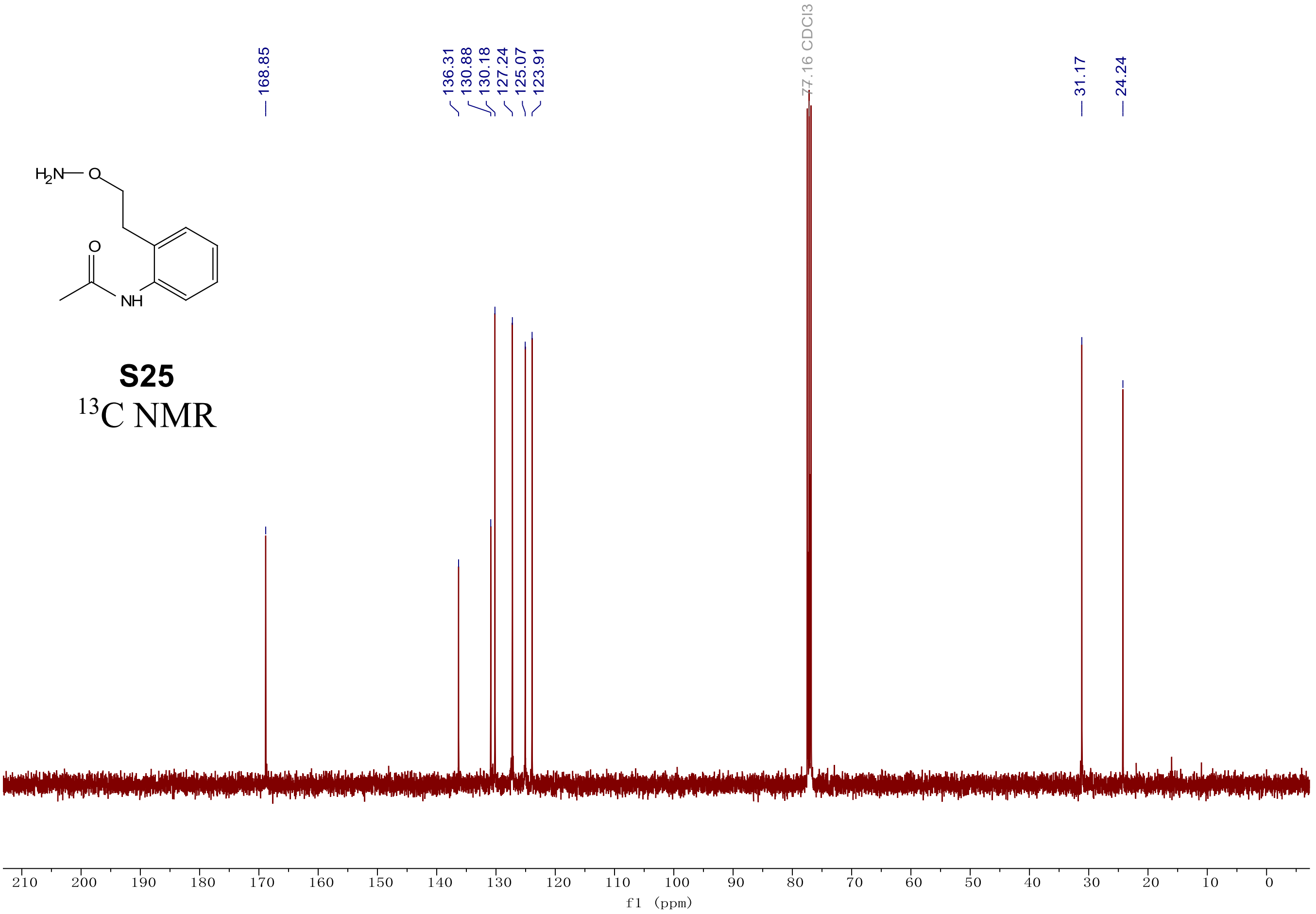
S25
13C NMR
168.85
136.31
130.88
130.18
127.24
125.07
123.91
77.16 CDCl3
31.17
24.24
f1 (ppm)

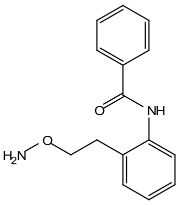

S26

^{1}H NMR

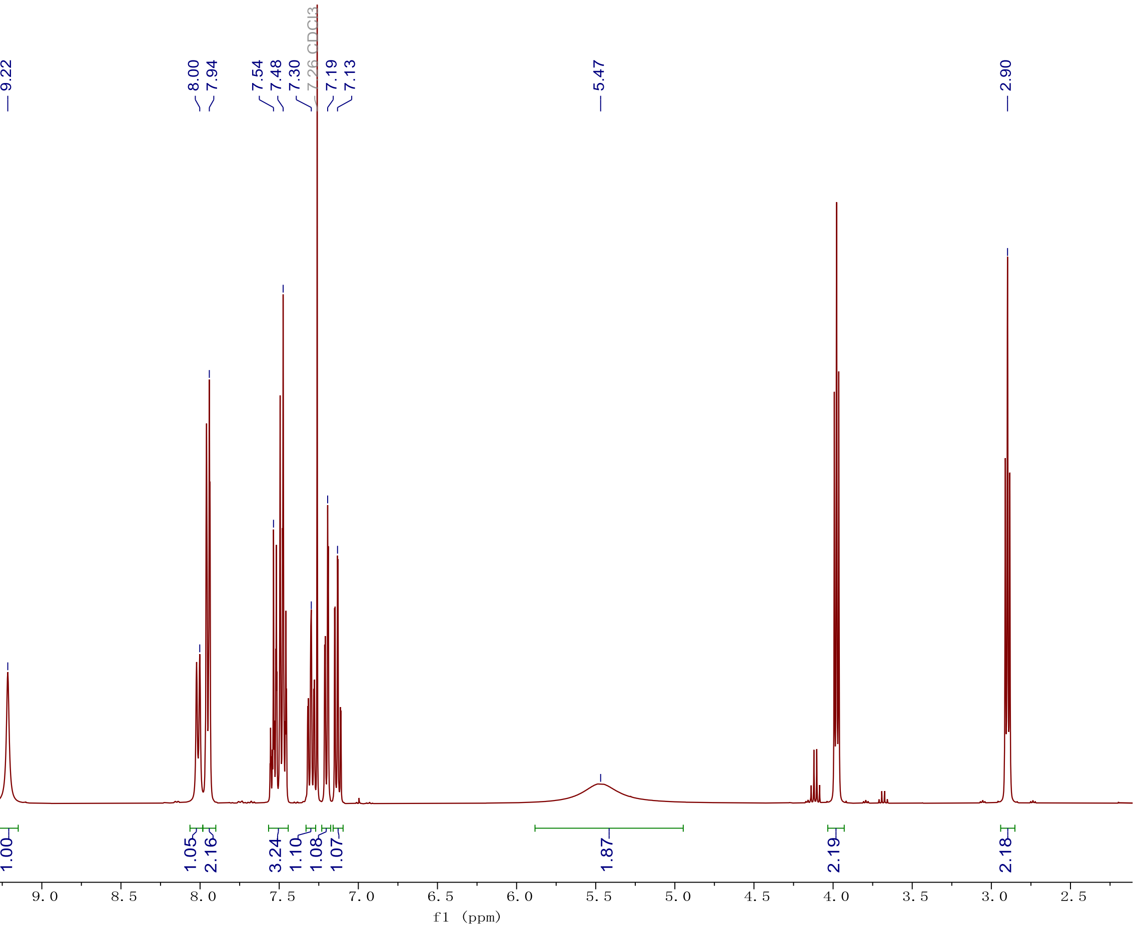

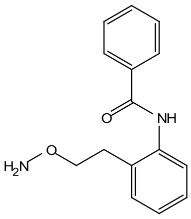

S26

^{13}C NMR

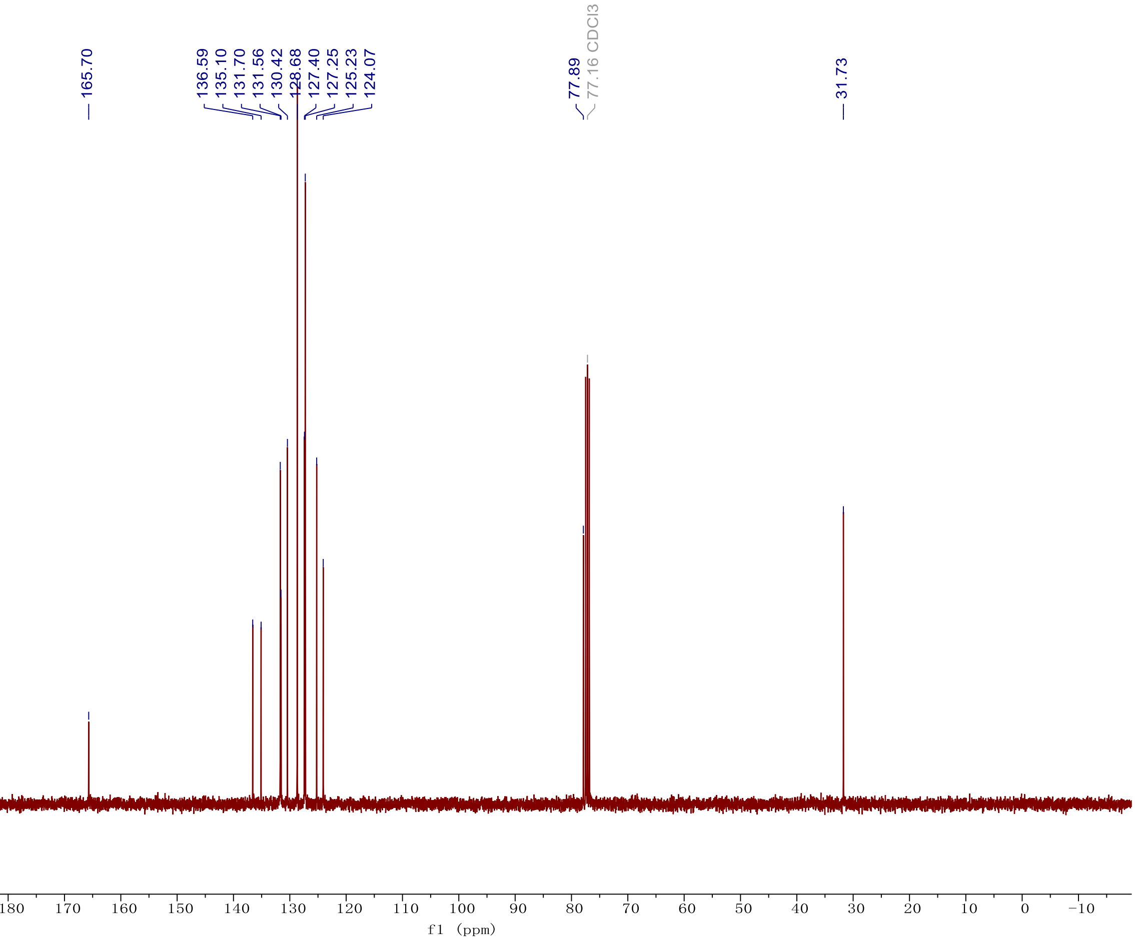

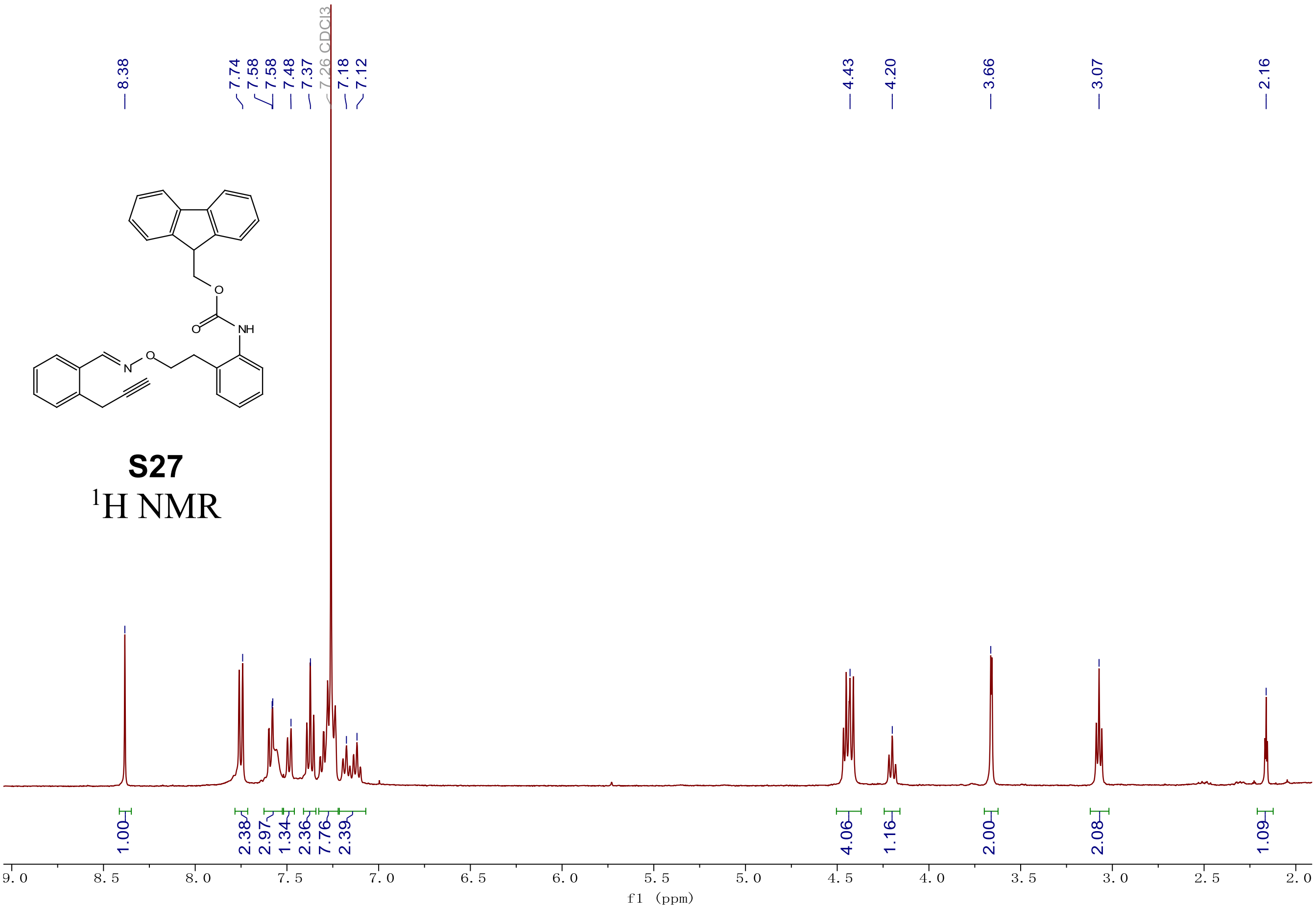
8.38
7.74
7.58
7.58
7.48
7.37
7.26 CDCl3
7.18
7.12
4.43
4.20
3.66
3.07
2.16
NH
O
N
S27
1H NMR
1.00
2.38
2.97
1.34
2.36
7.76
2.39
4.06
1.16
2.00
2.08
1.09
9.0 8.5 8.0 7.5 7.0 6.5 6.0 5.5 5.0 4.5 4.0 3.5 3.0 2.5 2.0
f1 (ppm)

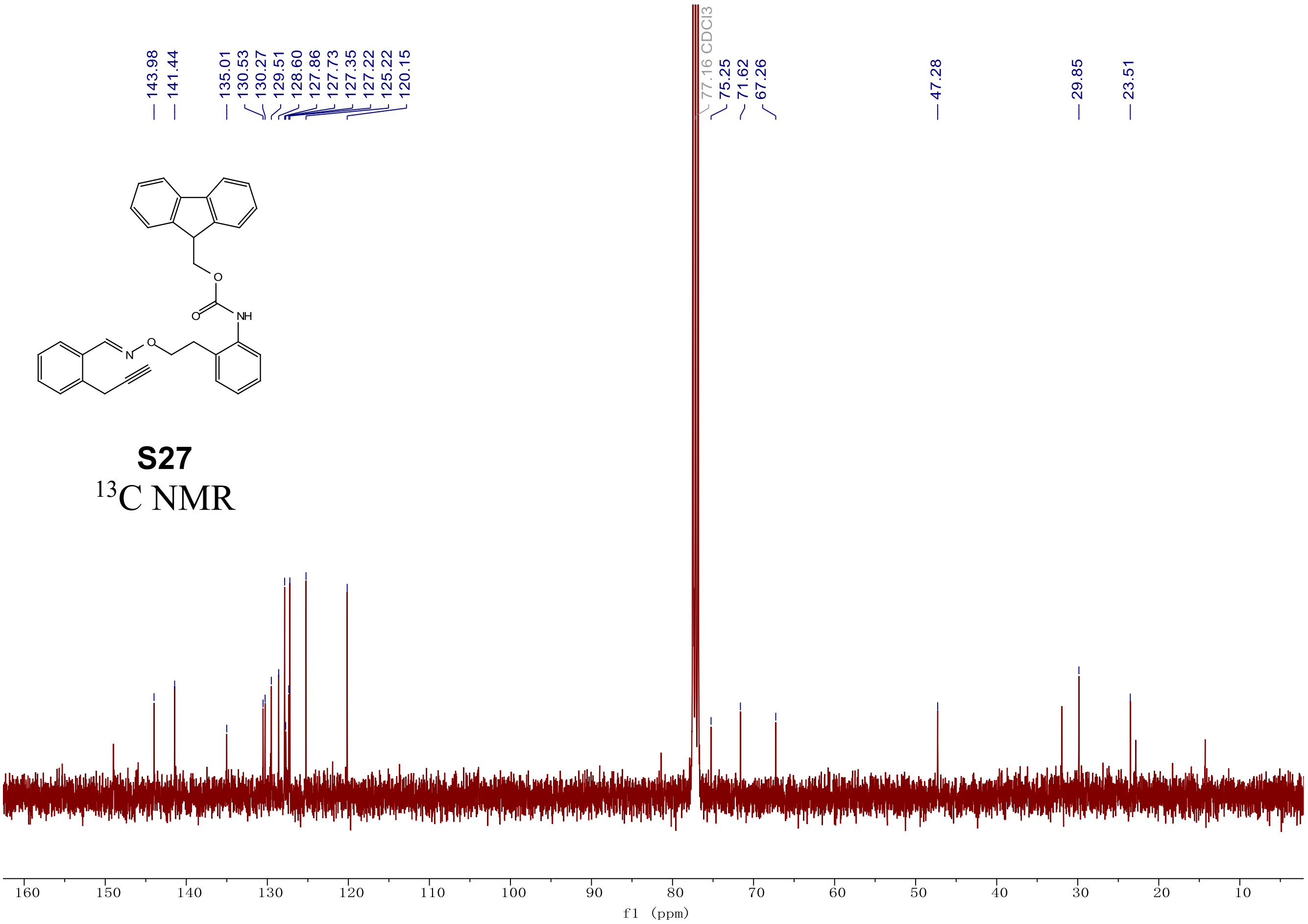
143.98
141.44
135.01
130.53
130.27
129.51
128.60
127.86
127.73
127.35
127.22
125.22
120.15
77.16 CDCl3
75.25
71.62
67.26
47.28
29.85
23.51
NH
O
N
S27
13C NMR
160 150 140 130 120 110 100 90 80 70 60 50 40 30 20 10
f1 (ppm)

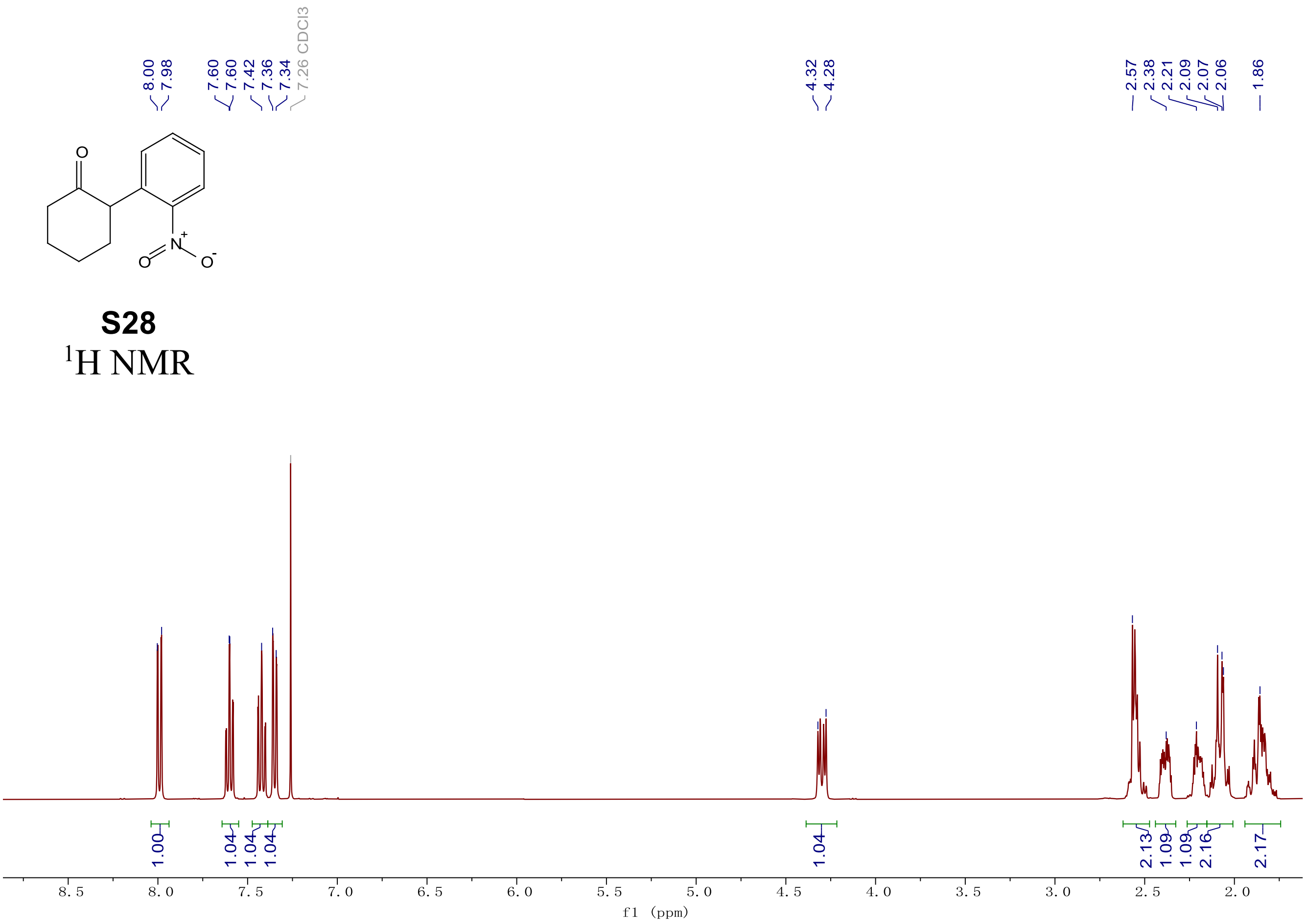

S28

^{1}H NMR

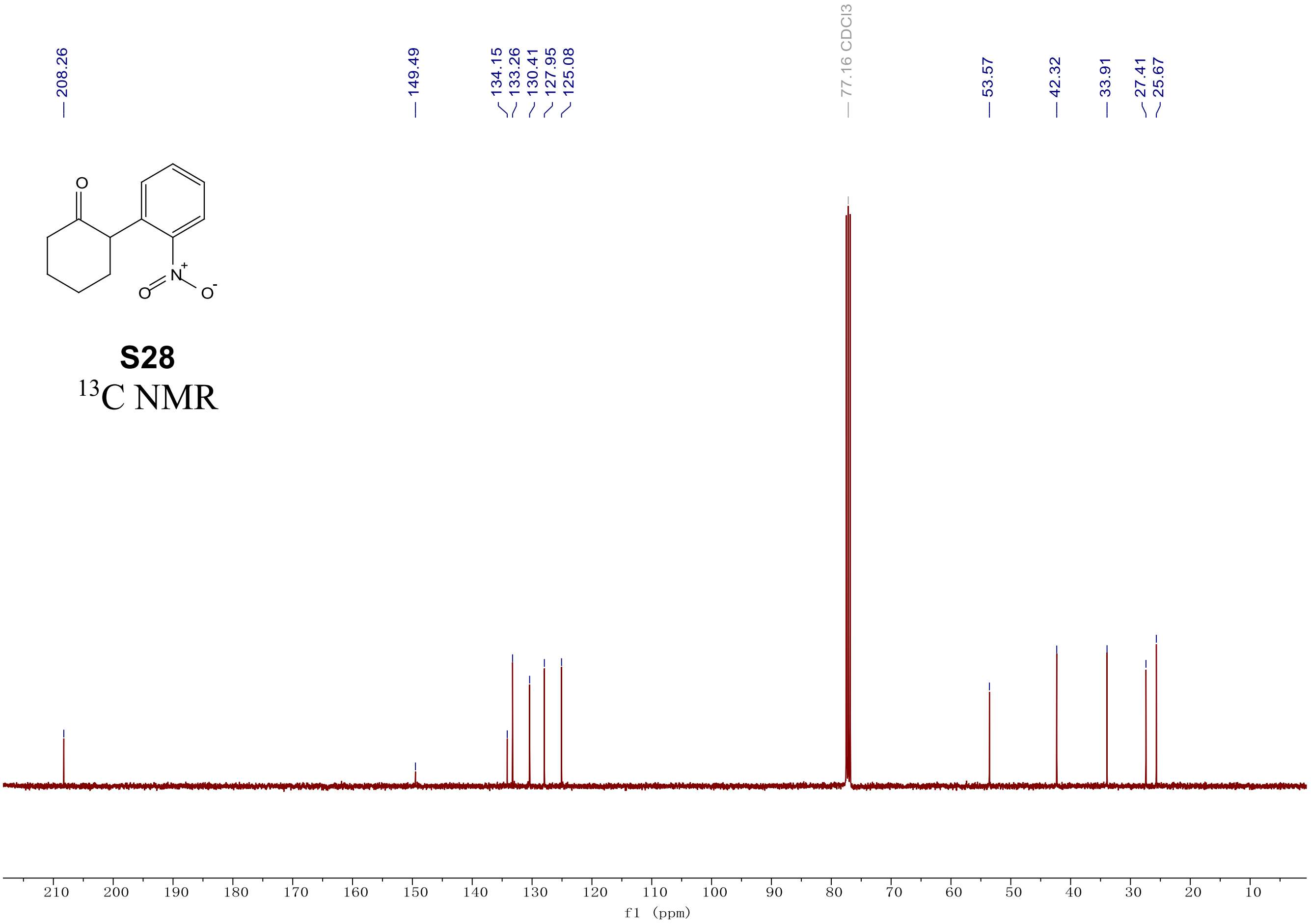

S28

^{13}C NMR

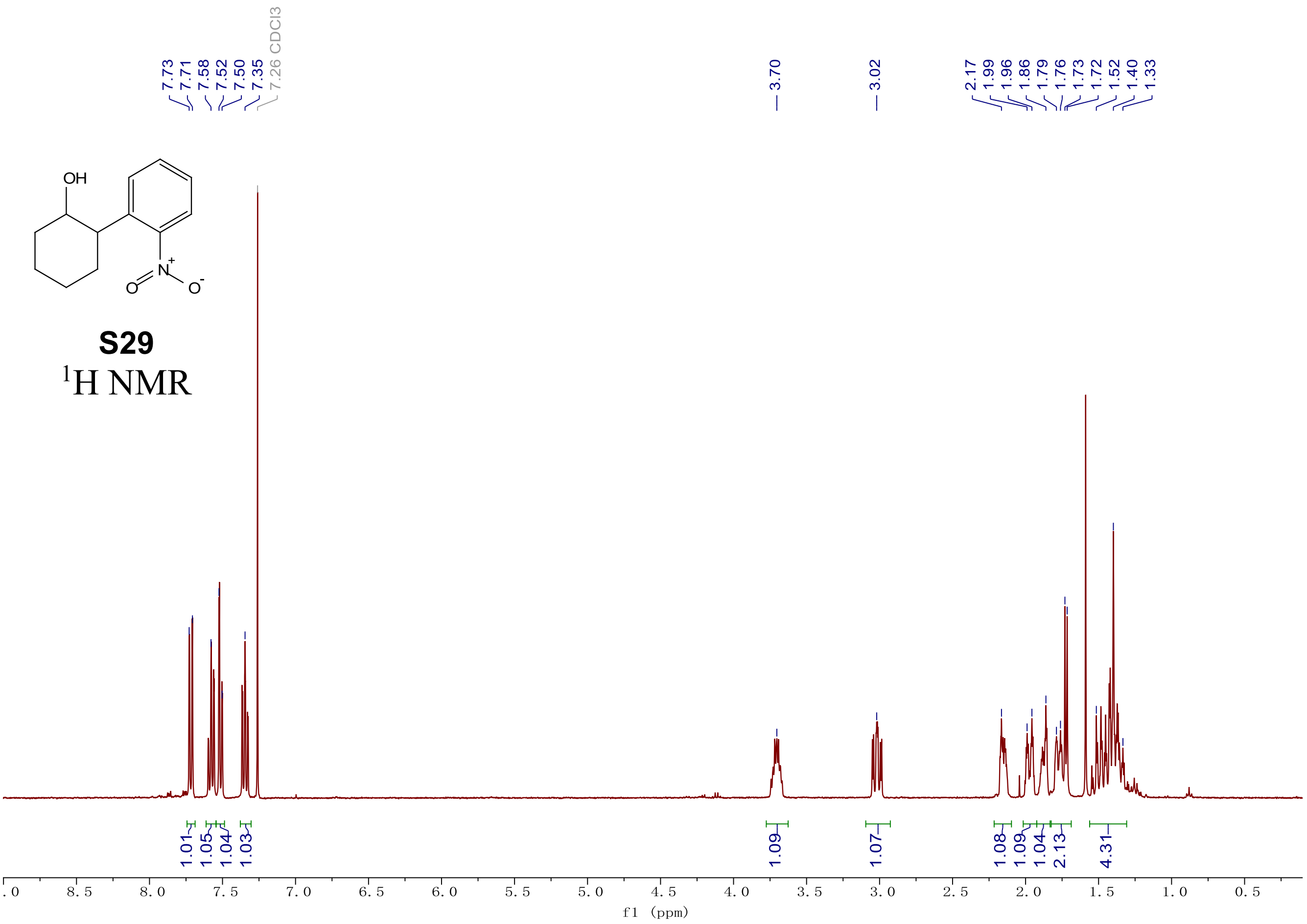
7.73
7.71
7.58
7.52
7.50
7.35
7.26 CDCl3
3.70
3.02
2.17
1.99
1.96
1.86
1.79
1.76
1.73
1.72
1.52
1.40
1.33
OH
N
O
O
S29
1H NMR
1.01
1.05
1.04
1.03
1.09
1.07
1.08
1.09
1.04
2.13
4.31
f1 (ppm)

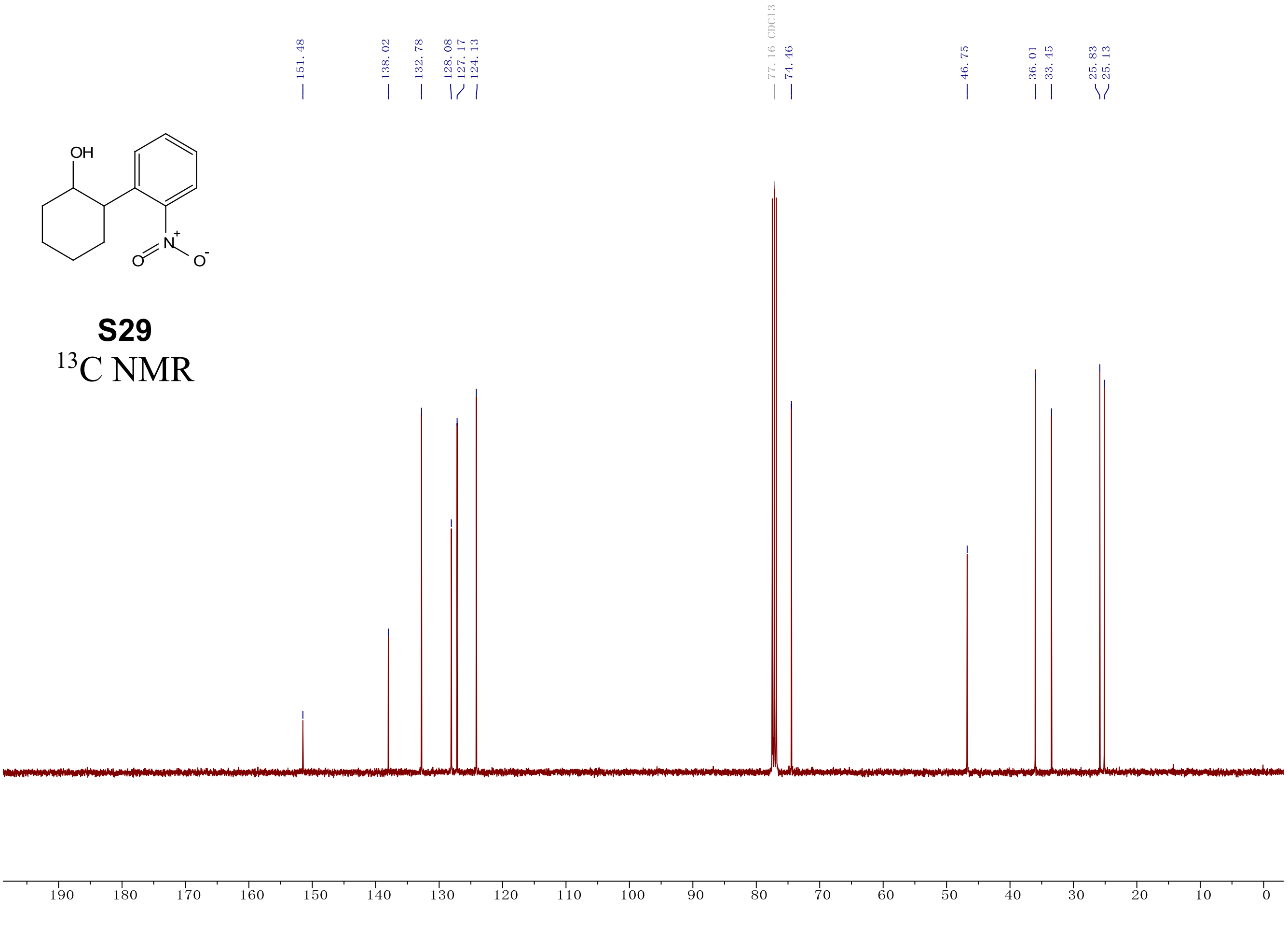
151.48
138.02
132.78
128.08
127.17
124.13
77.16 CDCl3
74.46
46.75
36.01
33.45
25.83
25.13
OH
N
O
O
S29
13C NMR

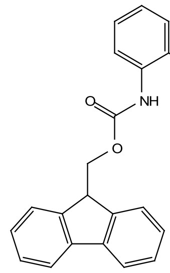

S30
^{1}H NMR

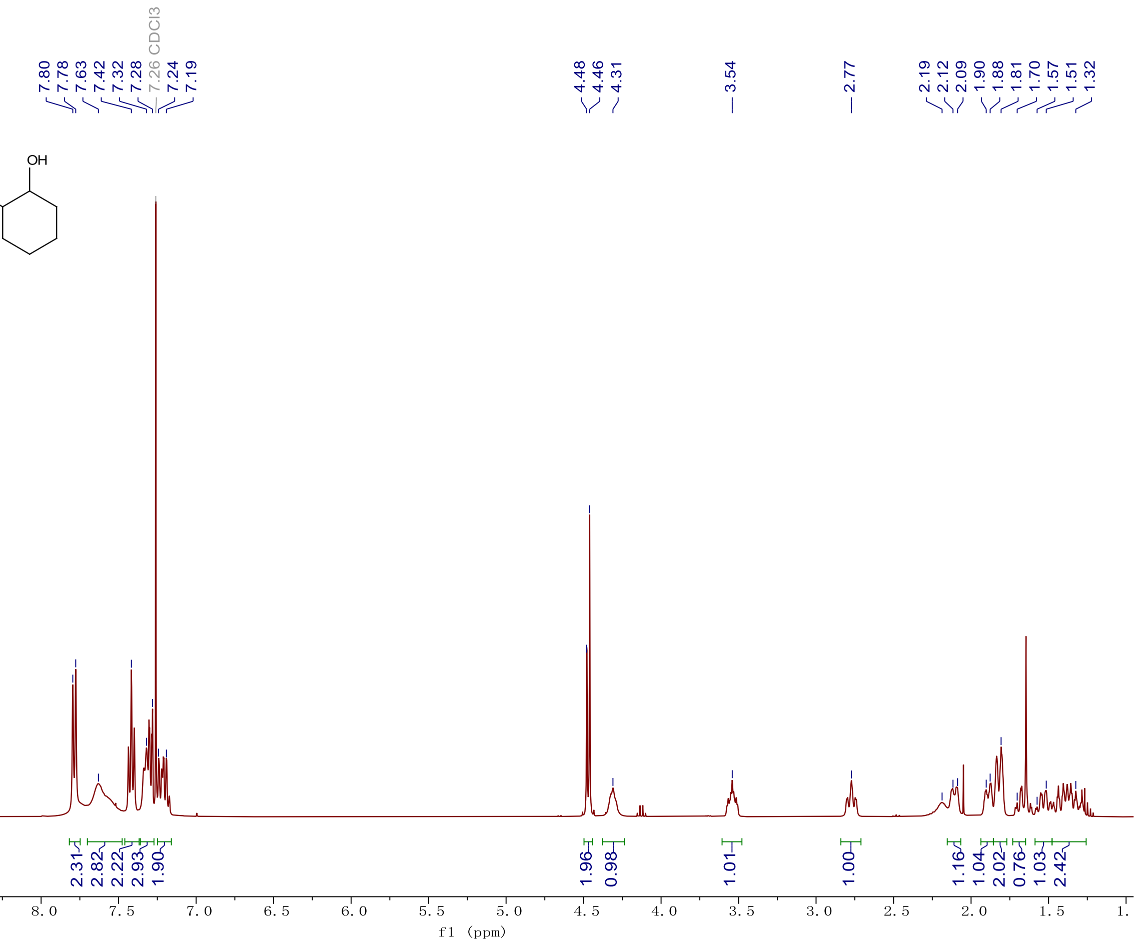

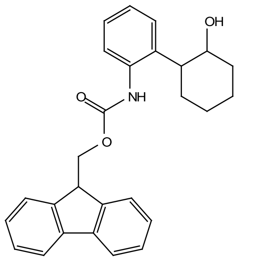

S30
^{13}C NMR

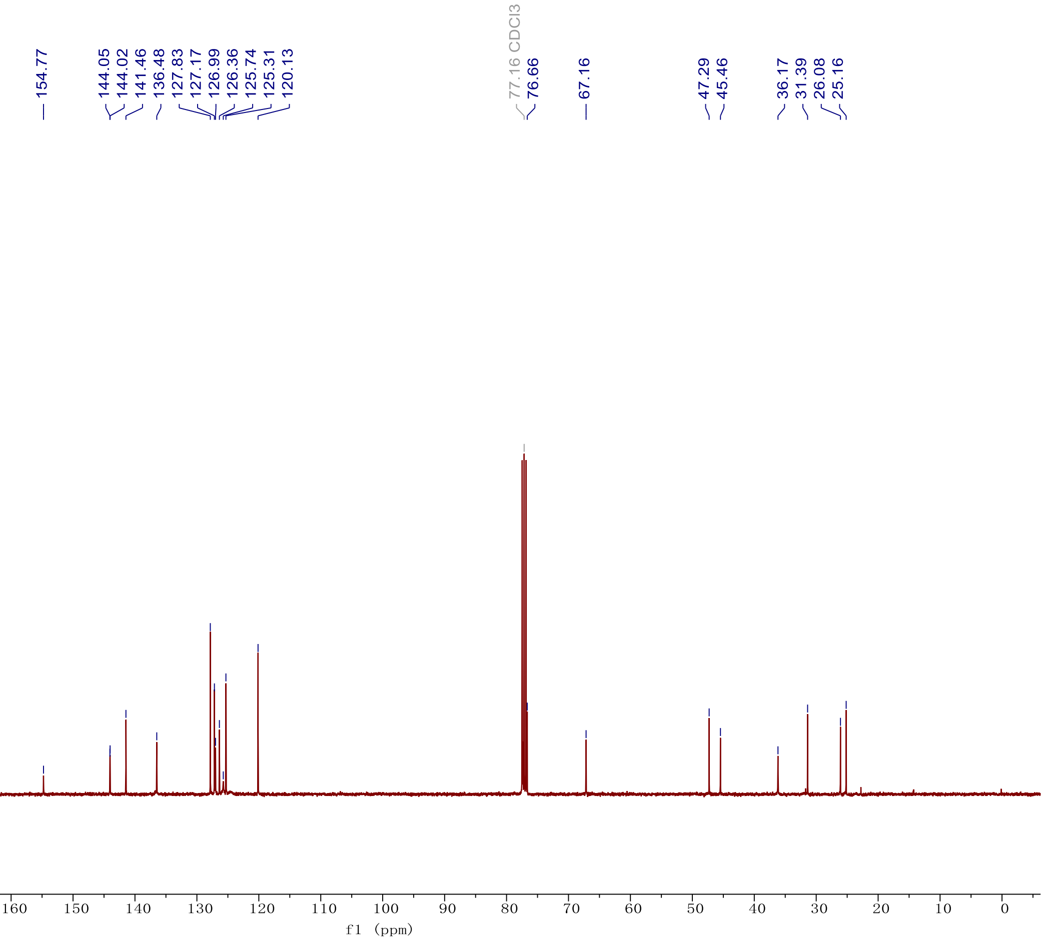

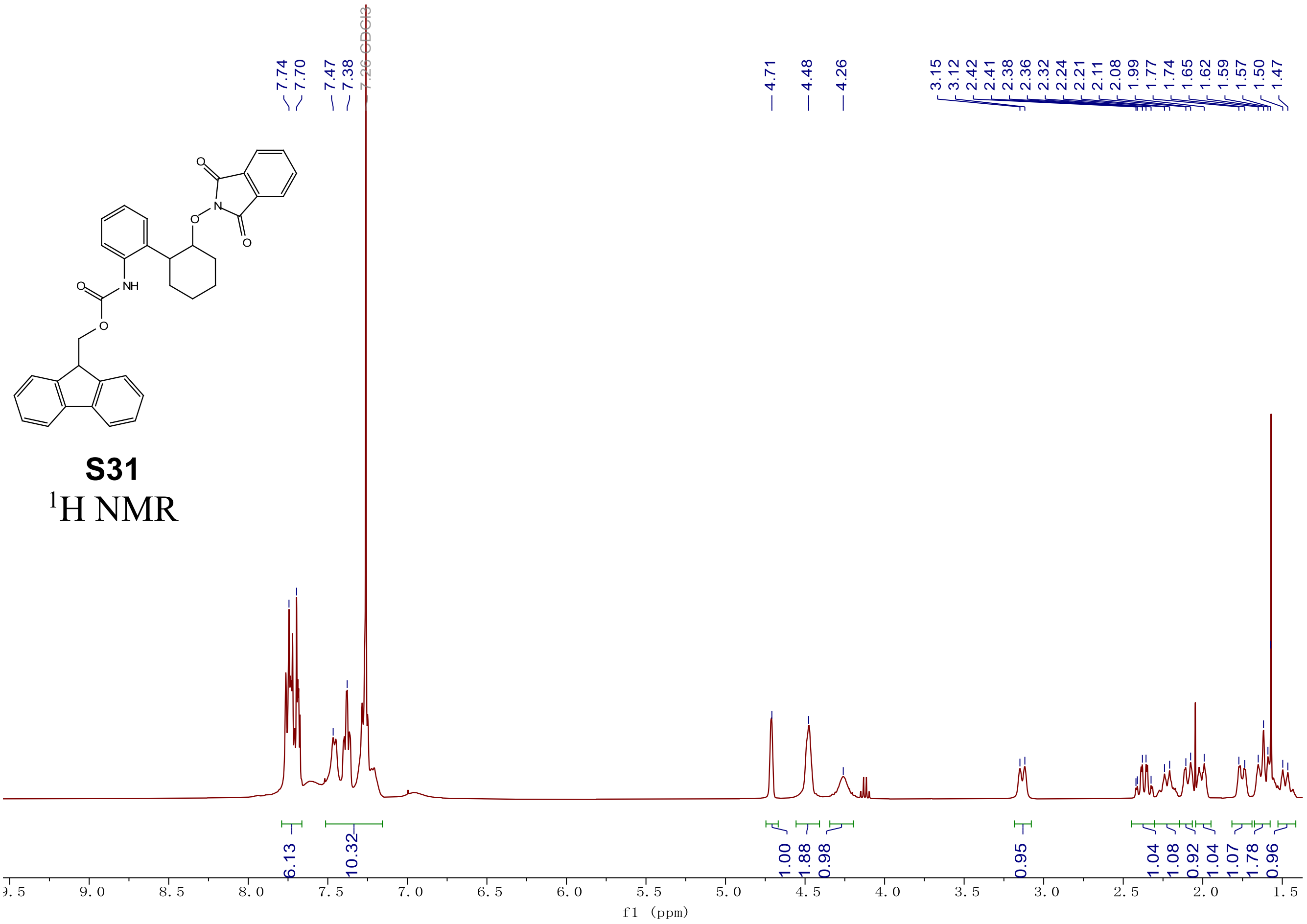

S31

^{1}H NMR

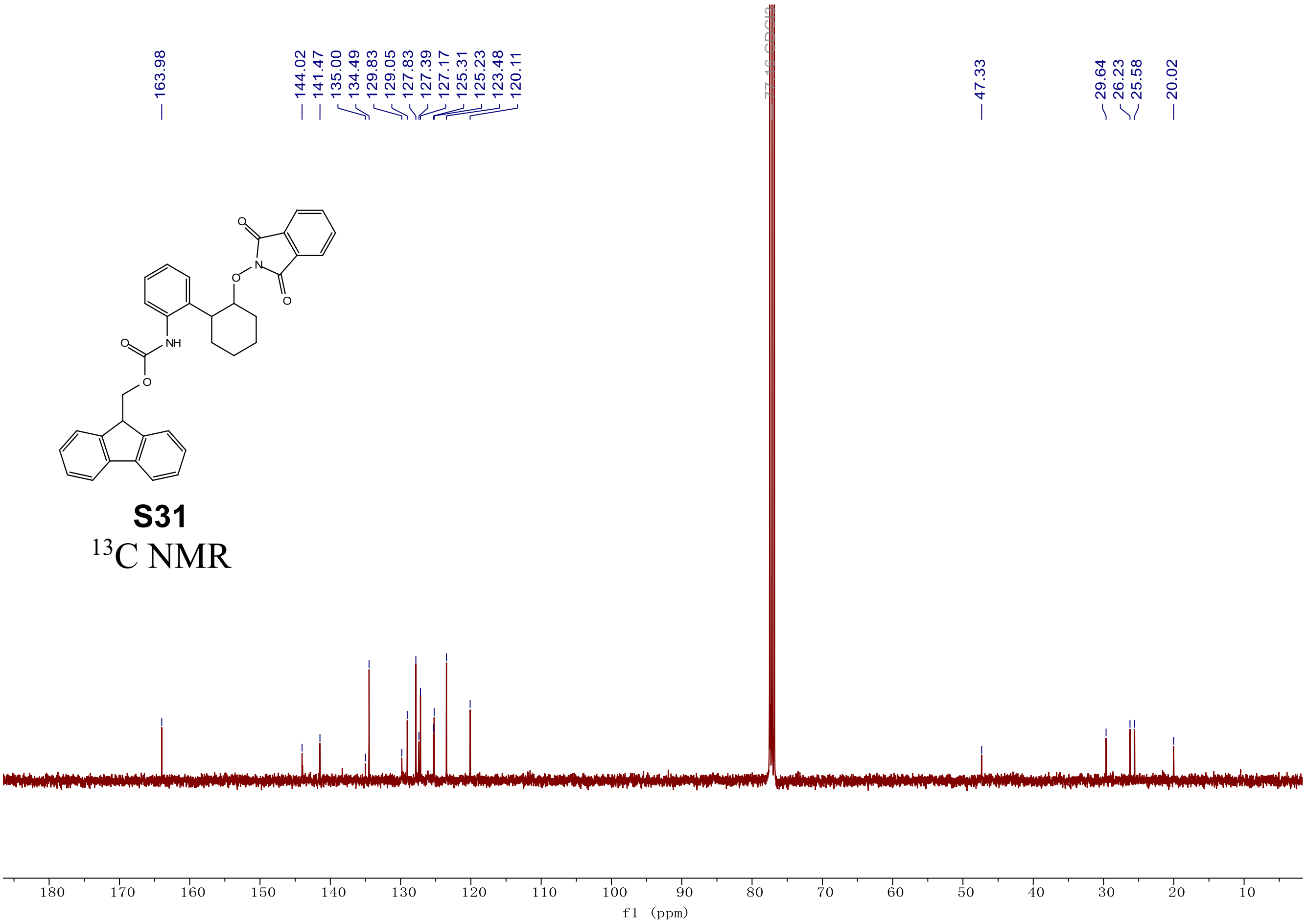

S31

^{13}C NMR

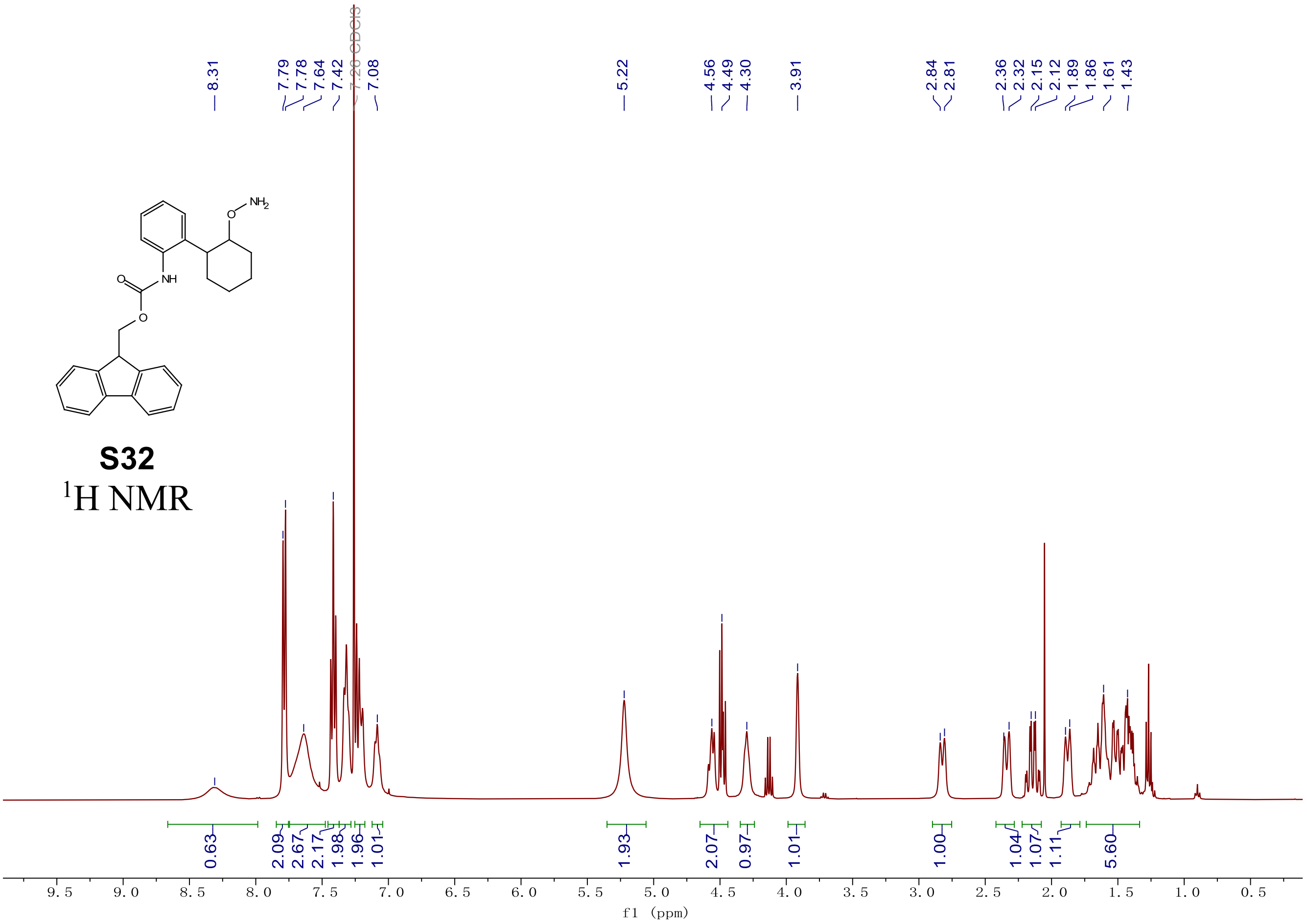
8.31
7.79
7.78
7.64
7.42
7.26 CDCl3
7.08
5.22
4.56
4.49
4.30
3.91
2.84
2.81
2.36
2.32
2.15
2.12
1.89
1.86
1.61
1.43
O
NH2
NH
O
O
S32
1H NMR
0.63
2.09
2.67
2.17
1.98
1.96
1.01
1.93
2.07
0.97
1.01
1.00
1.04
1.07
1.11
5.60
9.5
9.0
8.5
8.0
7.5
7.0
6.5
6.0
5.5
5.0
4.5
4.0
3.5
3.0
2.5
2.0
1.5
1.0
0.5
f1 (ppm)

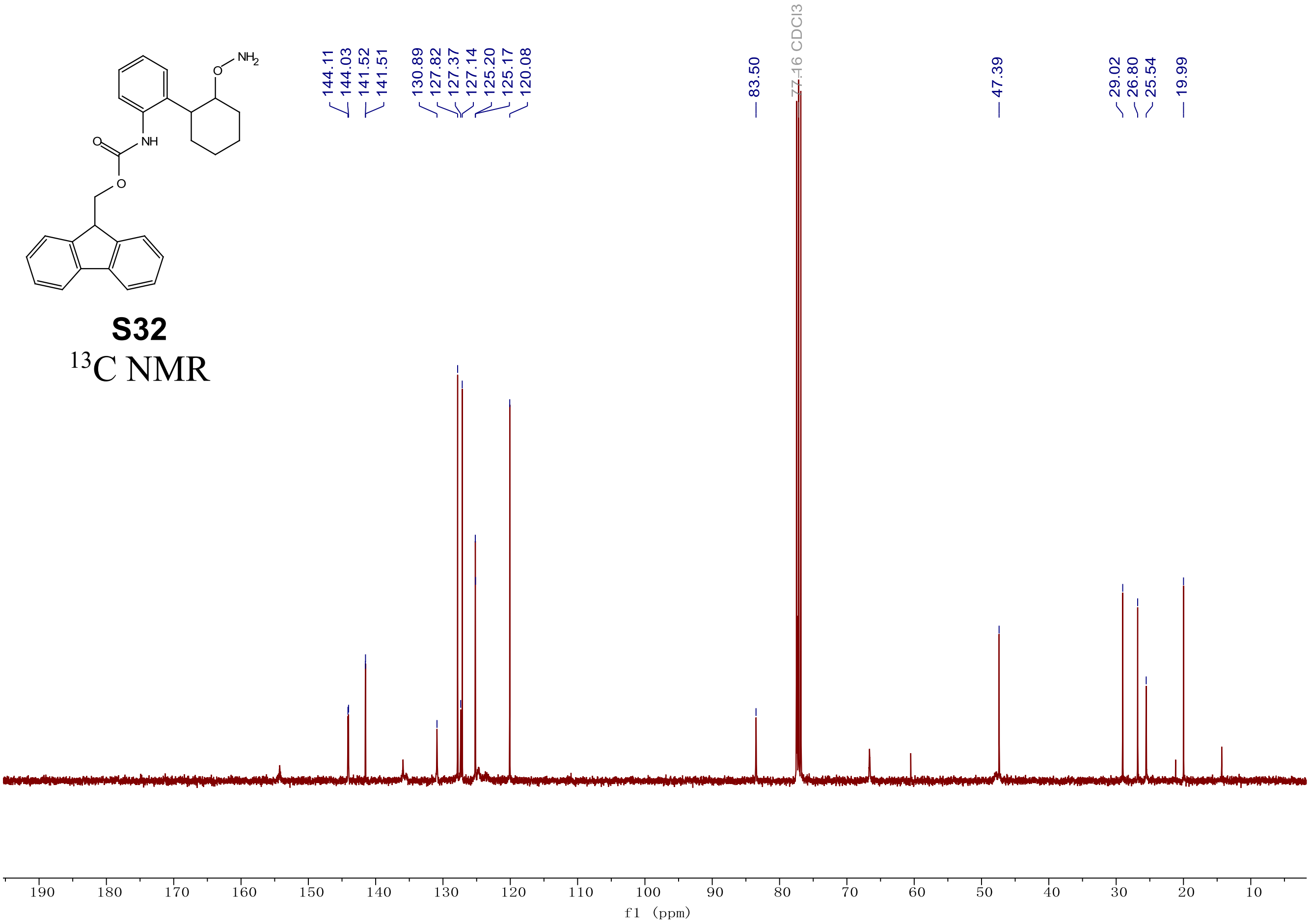
144.11
144.03
141.52
141.51
130.89
127.82
127.37
127.14
125.20
125.17
120.08
83.50
77.16 CDCl3
47.39
29.02
26.80
25.54
19.99
O
NH2
NH
O
O
S32
13C NMR
190
180
170
160
150
140
130
120
110
100
90
80
70
60
50
40
30
20
10
f1 (ppm)

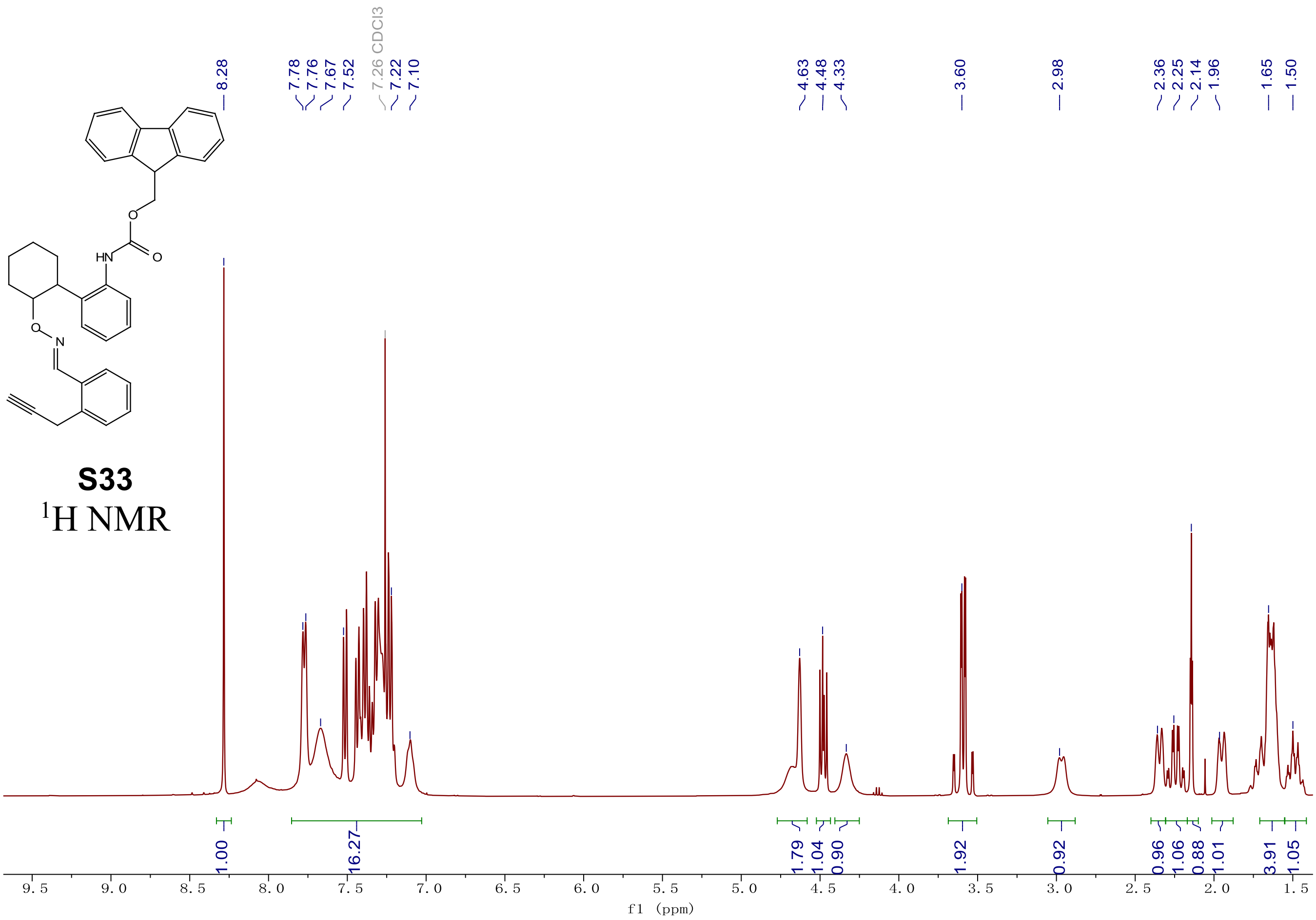

S33

^{1}H NMR

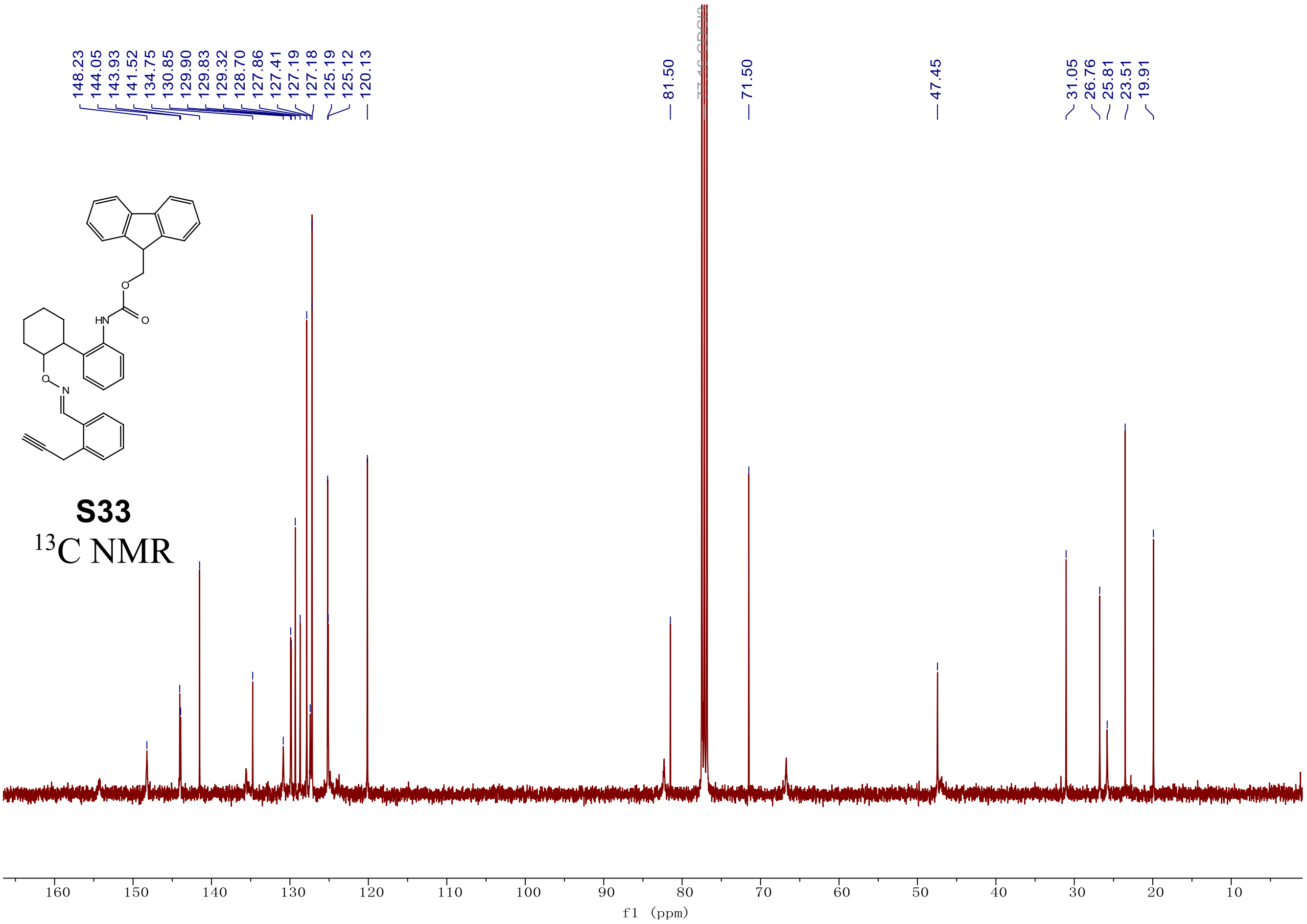

S33

^{13}C NMR

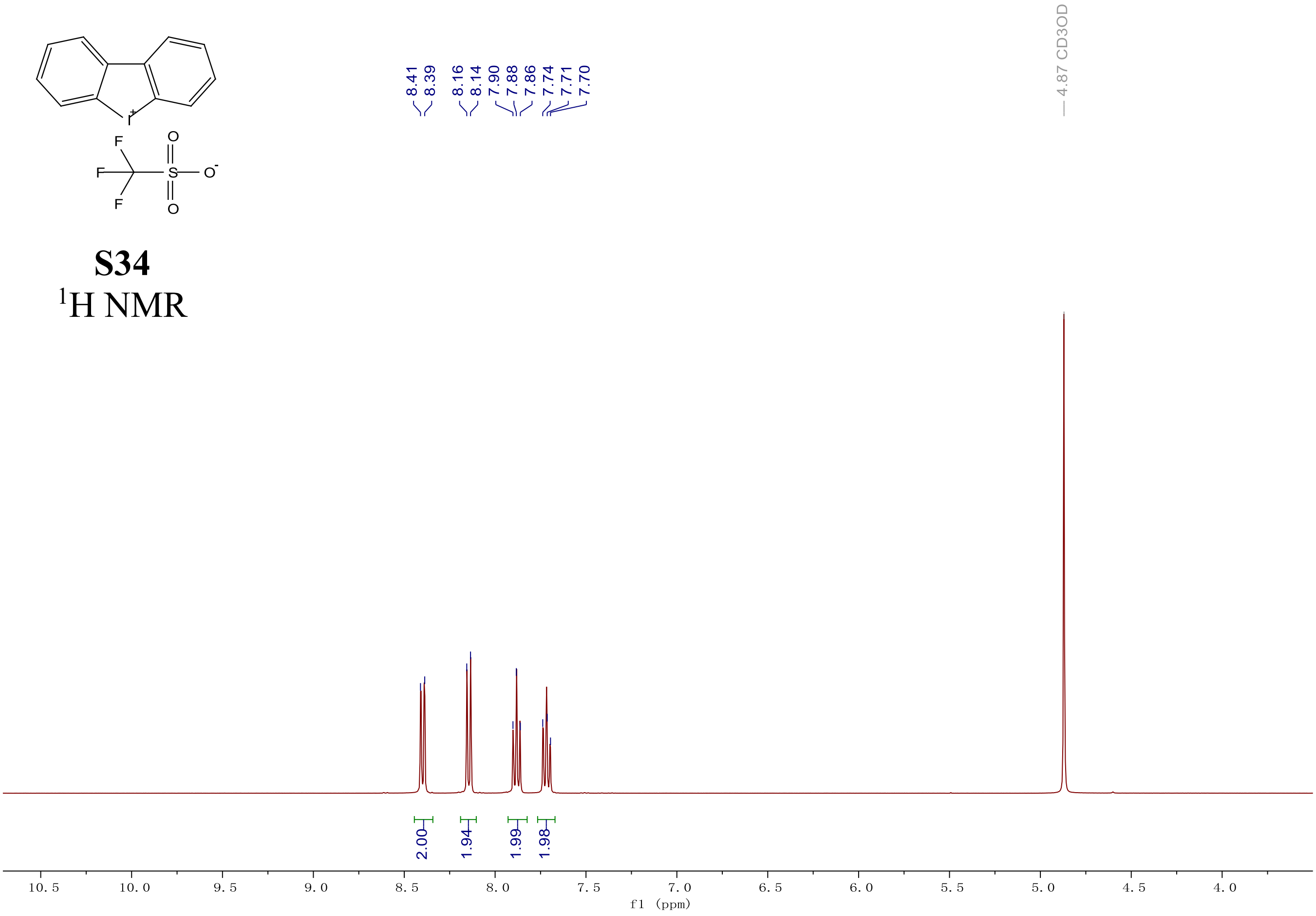
S34
1H NMR
8.41
8.39
8.16
8.14
7.90
7.88
7.86
7.74
7.71
7.70
4.87 CD3OD
2.00
1.94
1.99
1.98
f1 (ppm)

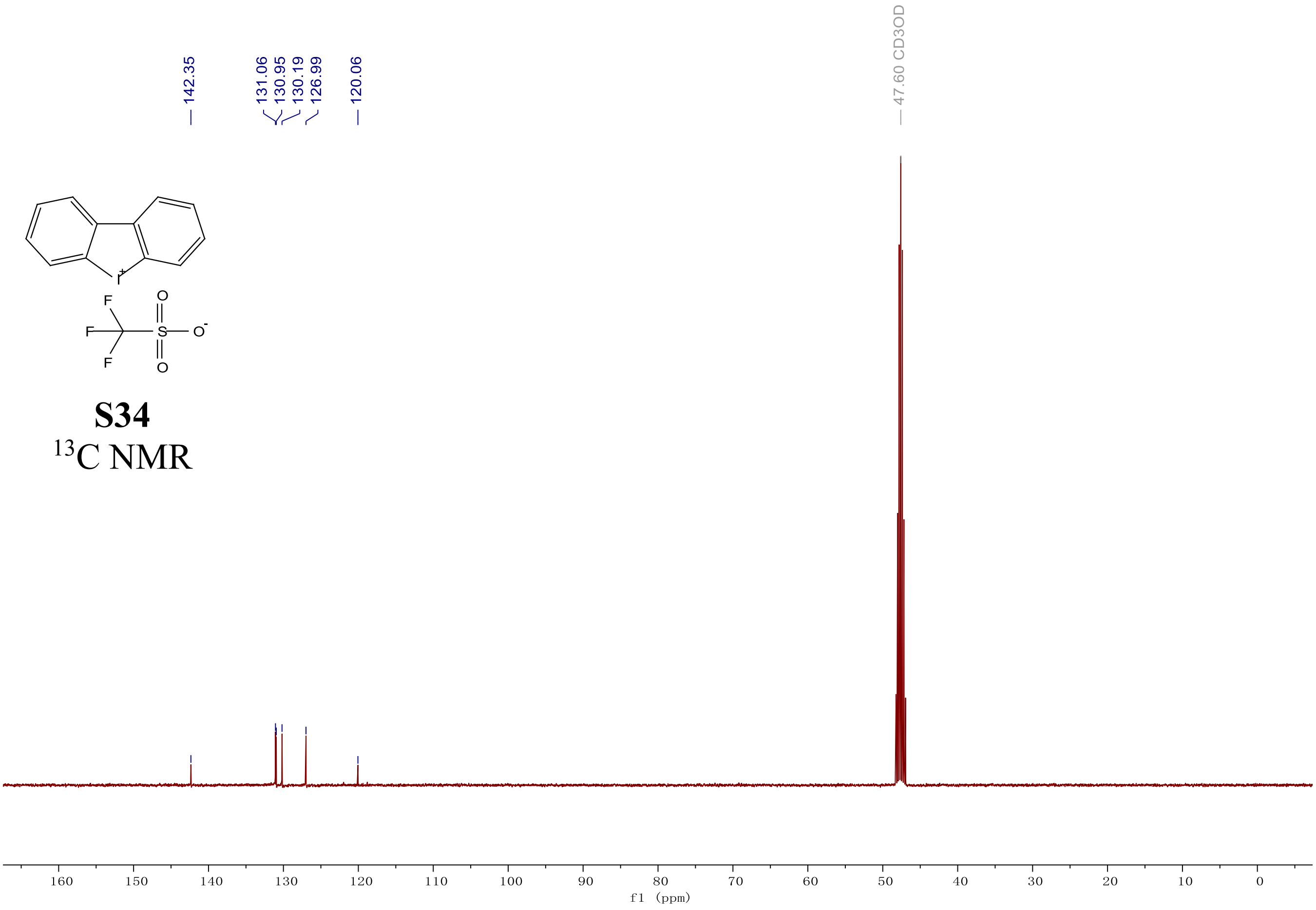
142.35
131.06
130.95
130.19
126.99
120.06
47.60 CD3OD
S34
13C NMR
f1 (ppm)

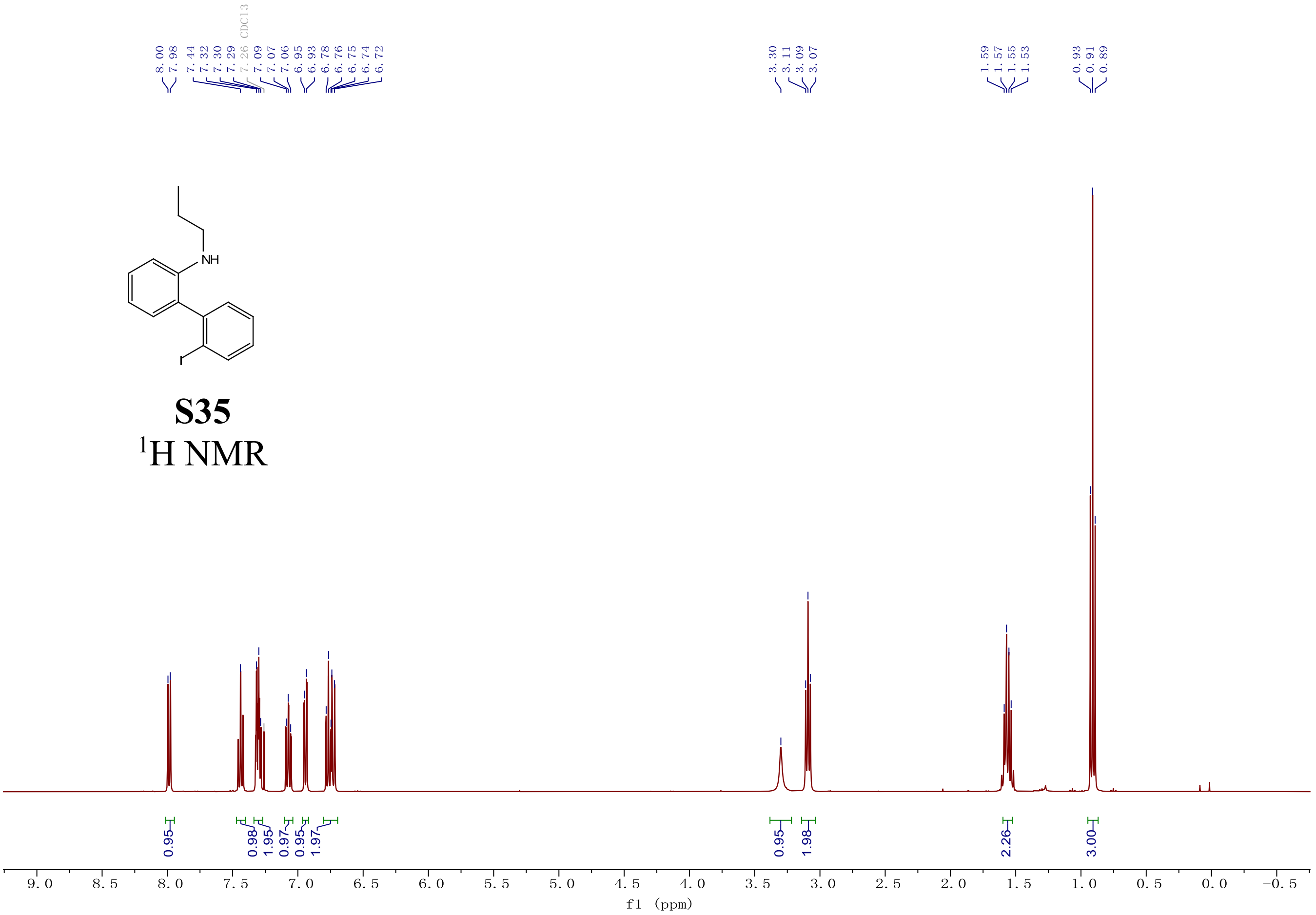
S35
1H NMR
f1 (ppm)

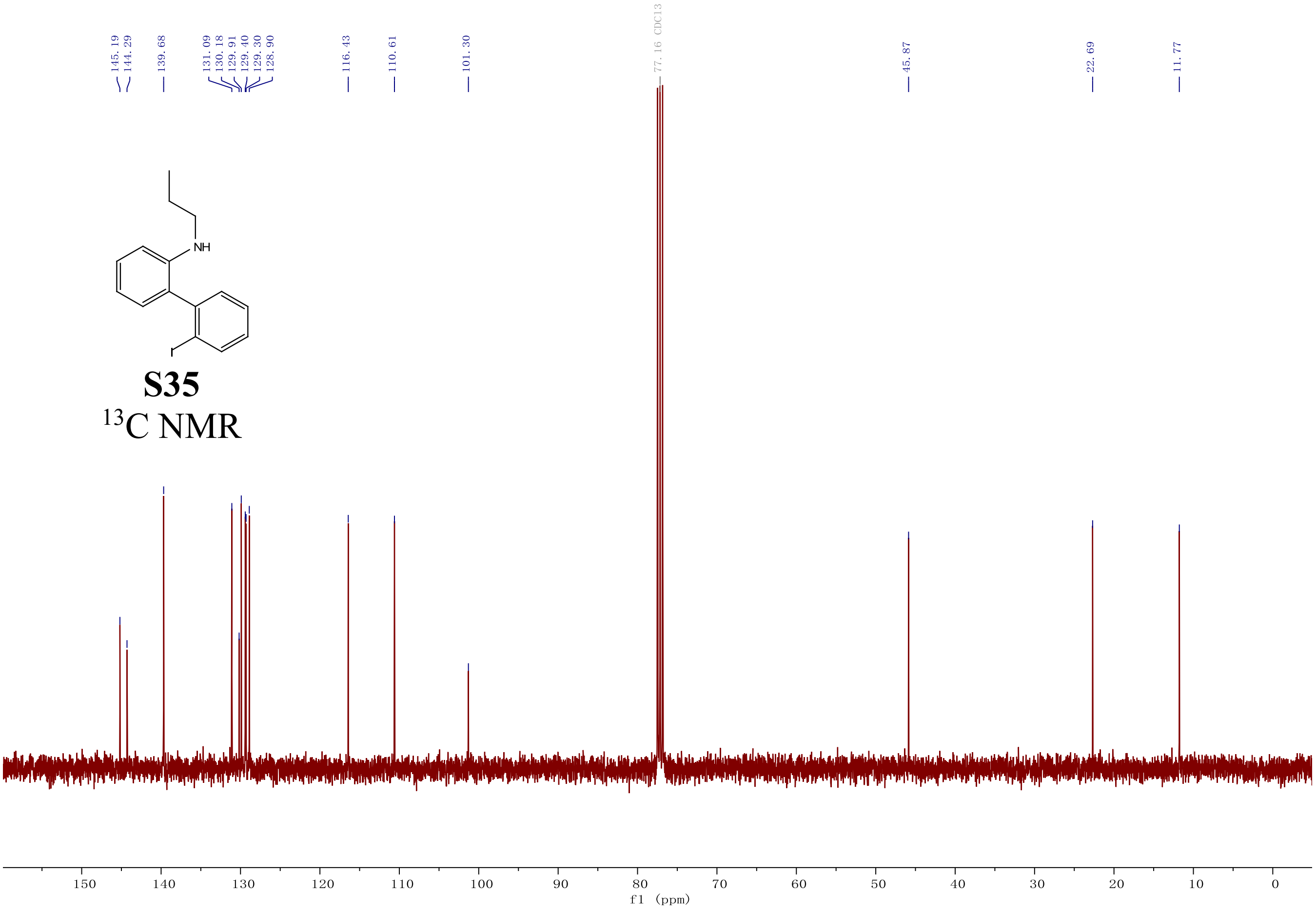
S35
13C NMR
f1 (ppm)

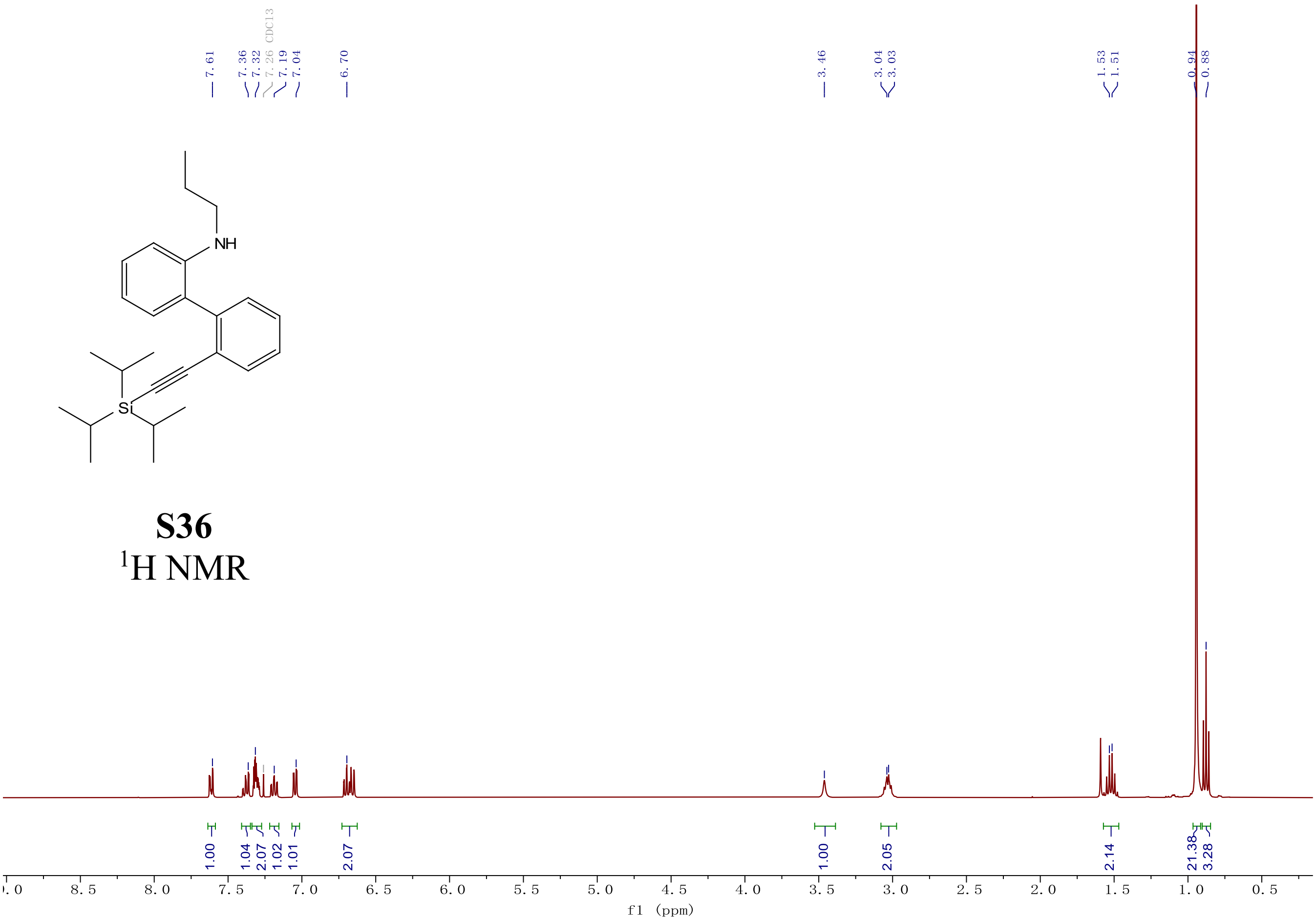

S36

^{1}H NMR

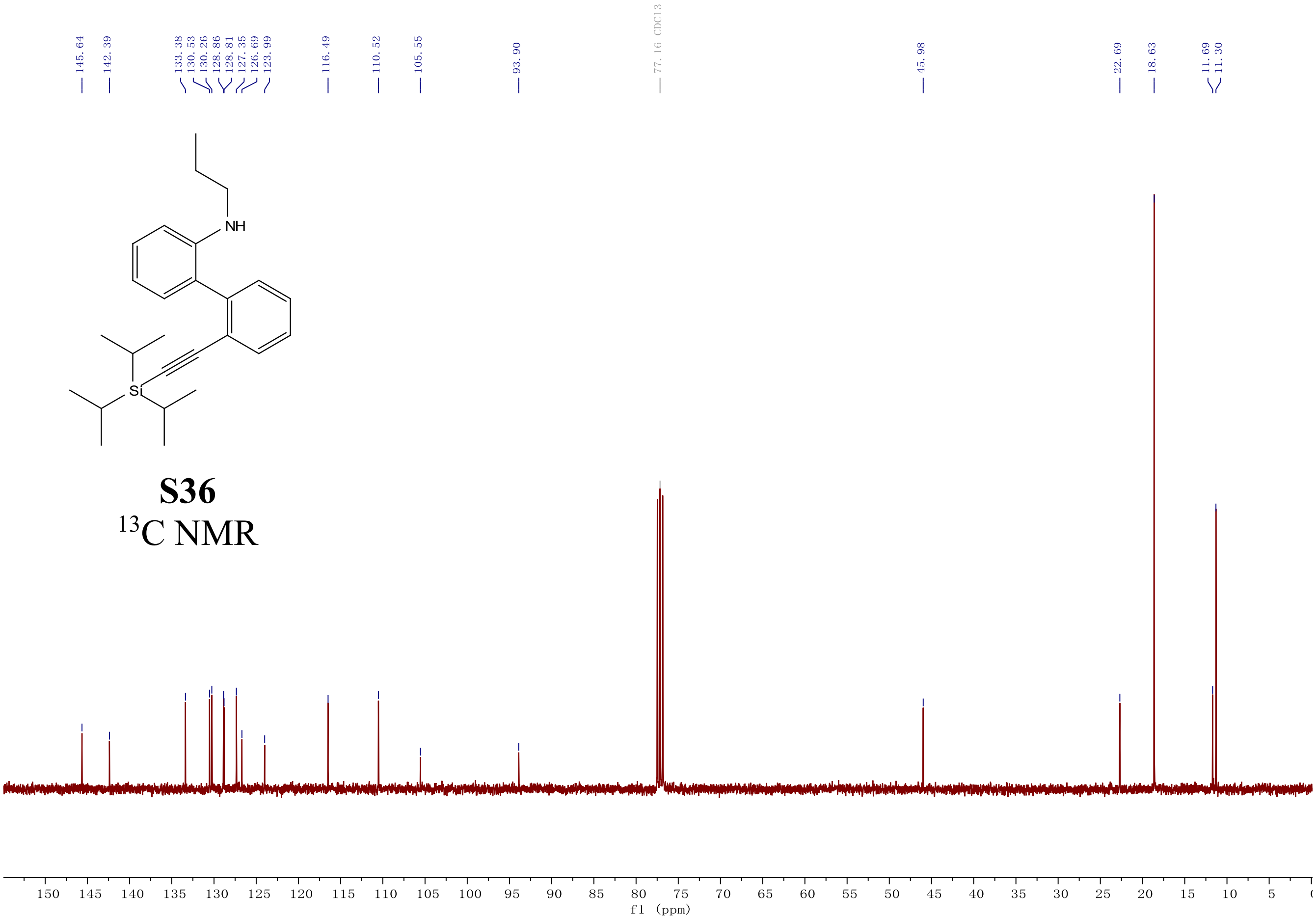

S36

^{13}C NMR

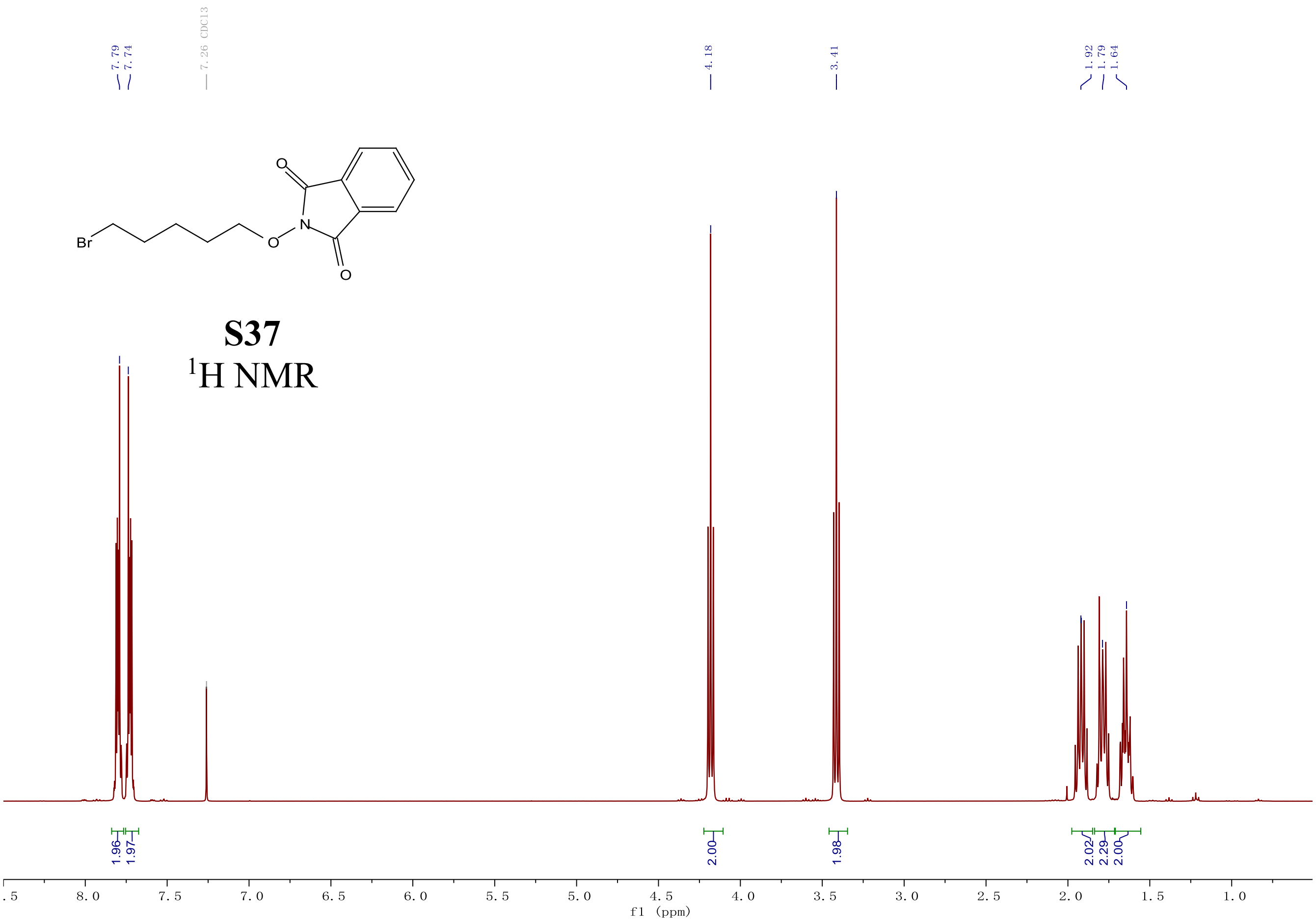
7.79
7.74
7.26 CDCl3
4.18
3.41
1.92
1.79
1.64
Br
O
N
O
O
S37
1H NMR
1.96
1.97
2.00
1.98
2.02
2.29
2.00
f1 (ppm)

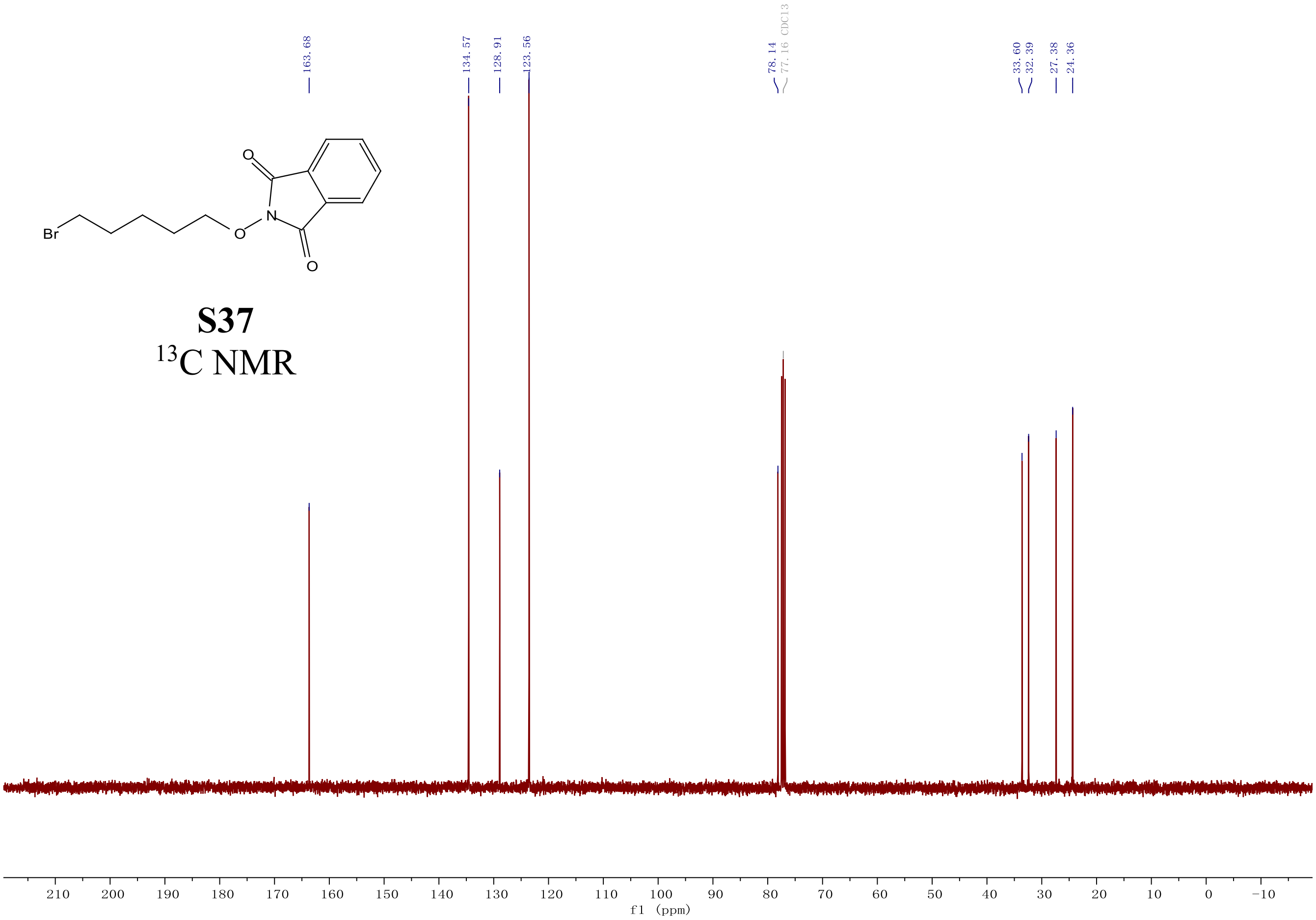
163.68
134.57
128.91
123.56
78.14
77.16 CDCl3
33.60
32.39
27.38
24.36
Br
O
N
O
O
S37
13C NMR
f1 (ppm)

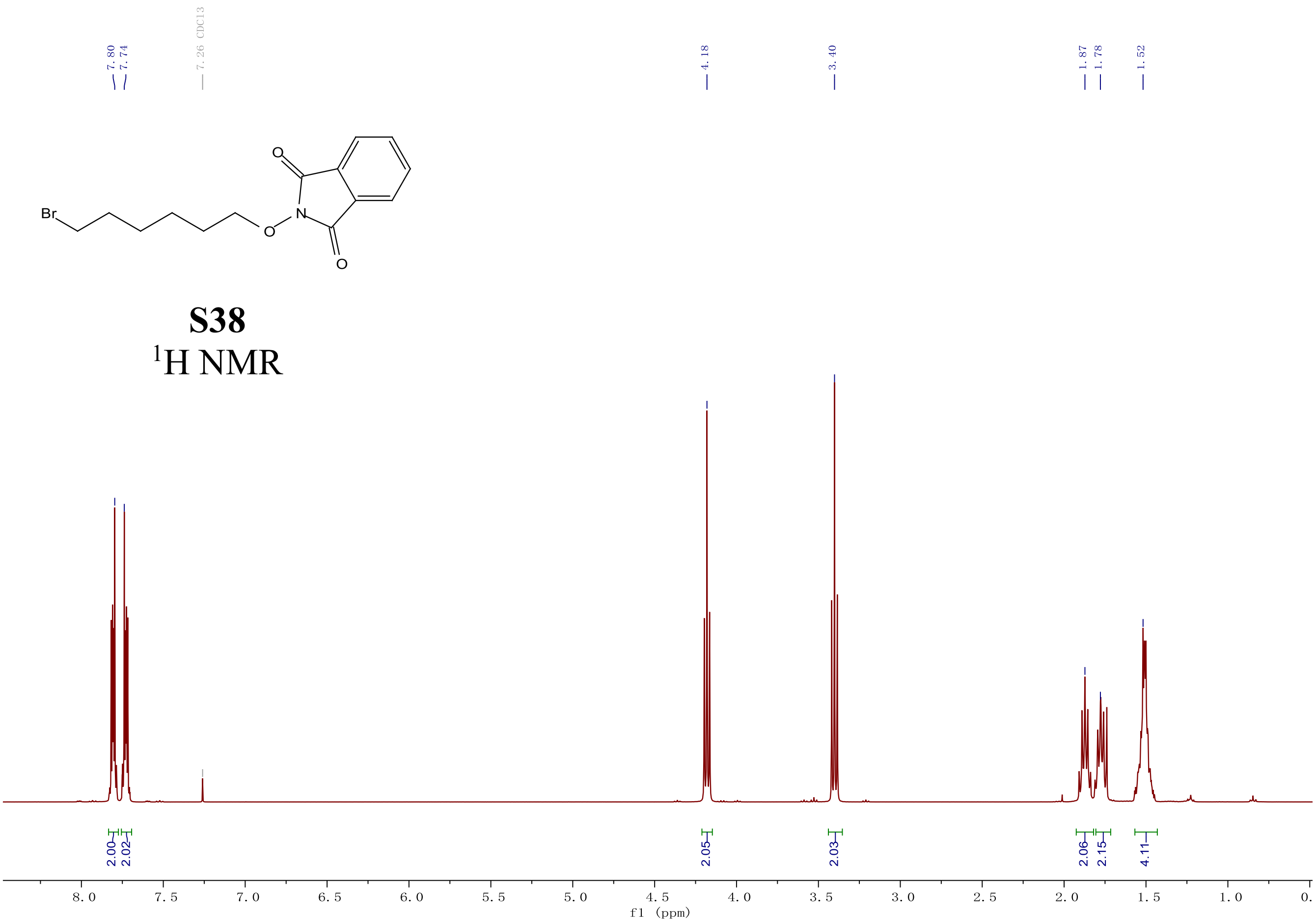
7.80
7.74
7.26 CDCl3
4.18
3.40
1.87
1.78
1.52
Br
O
N
O
O
S38
^{1}H NMR
2.00
2.02
2.05
2.03
2.06
2.15
4.11
8.0 7.5 7.0 6.5 6.0 5.5 5.0 4.5 4.0 3.5 3.0 2.5 2.0 1.5 1.0 0.
f1 (ppm)

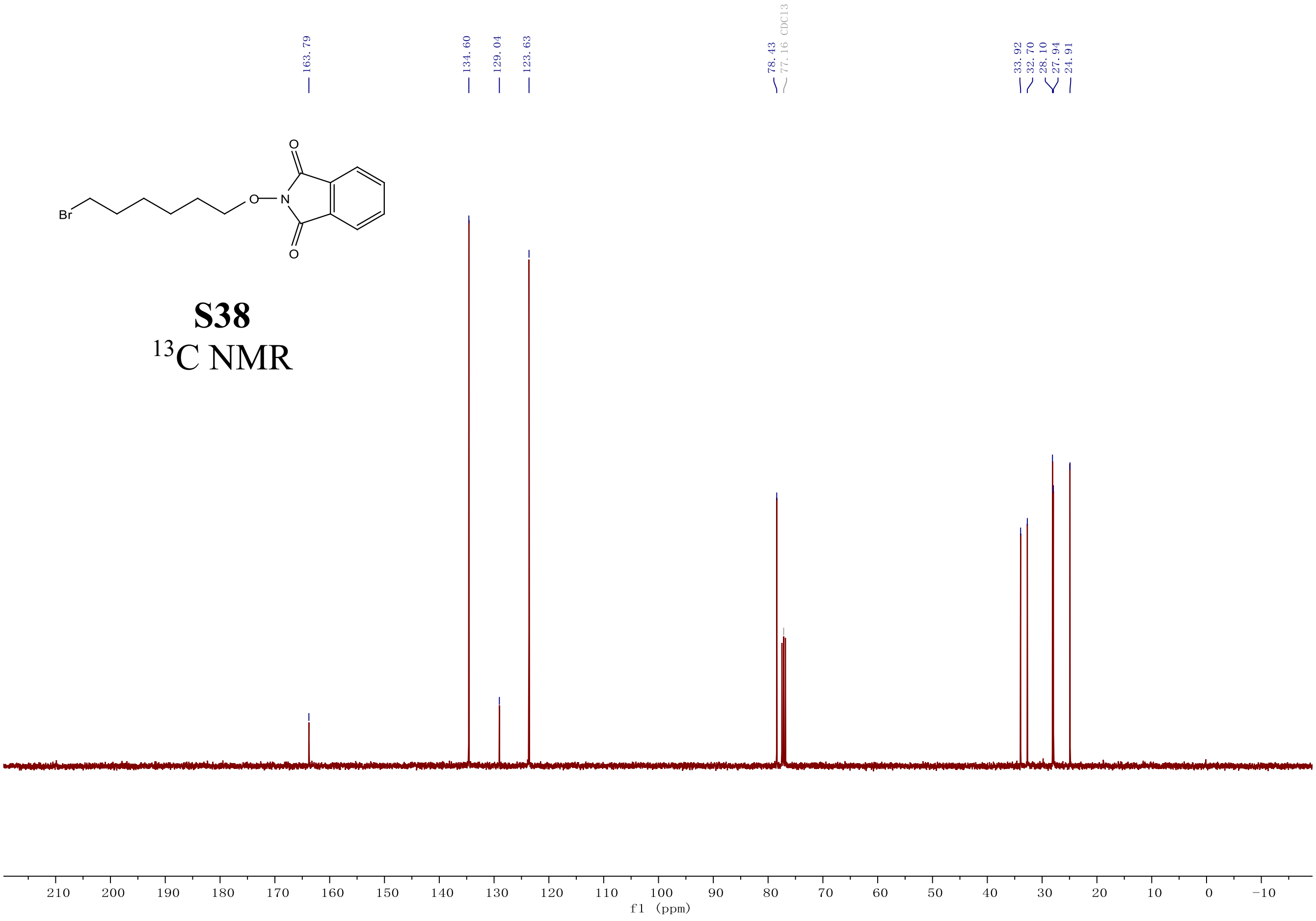
163.79
134.60
129.04
123.63
78.43
77.16 CDCl3
33.92
32.70
28.10
27.94
24.91
Br
O
N
O
O
S38
^{13}C NMR
210 200 190 180 170 160 150 140 130 120 110 100 90 80 70 60 50 40 30 20 10 0 -10
f1 (ppm)

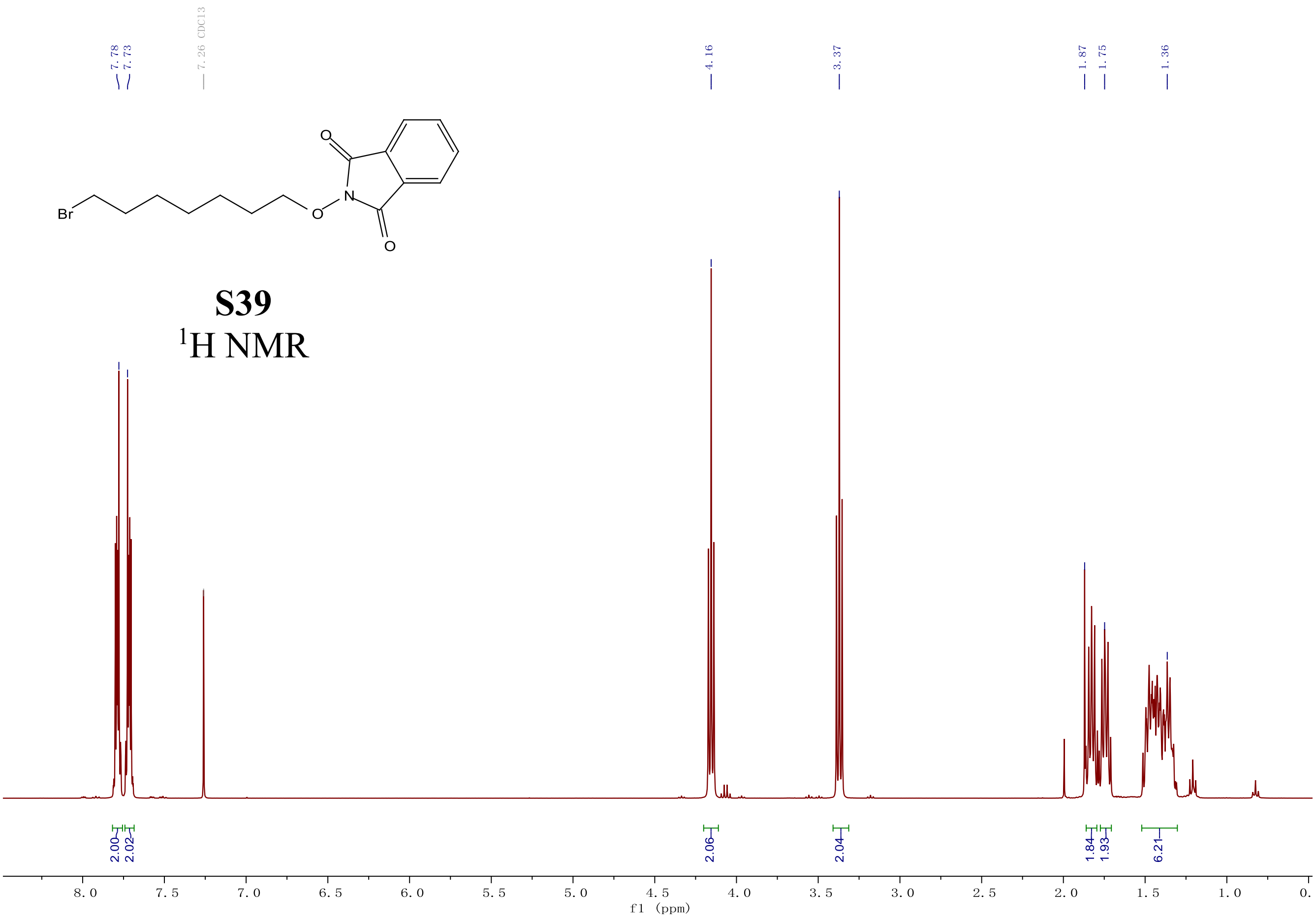
S39
1H NMR
7.78
7.73
7.26 CDCl3
4.16
3.37
1.87
1.75
1.36
2.00
2.02
2.06
2.04
1.84
1.93
6.21
f1 (ppm)

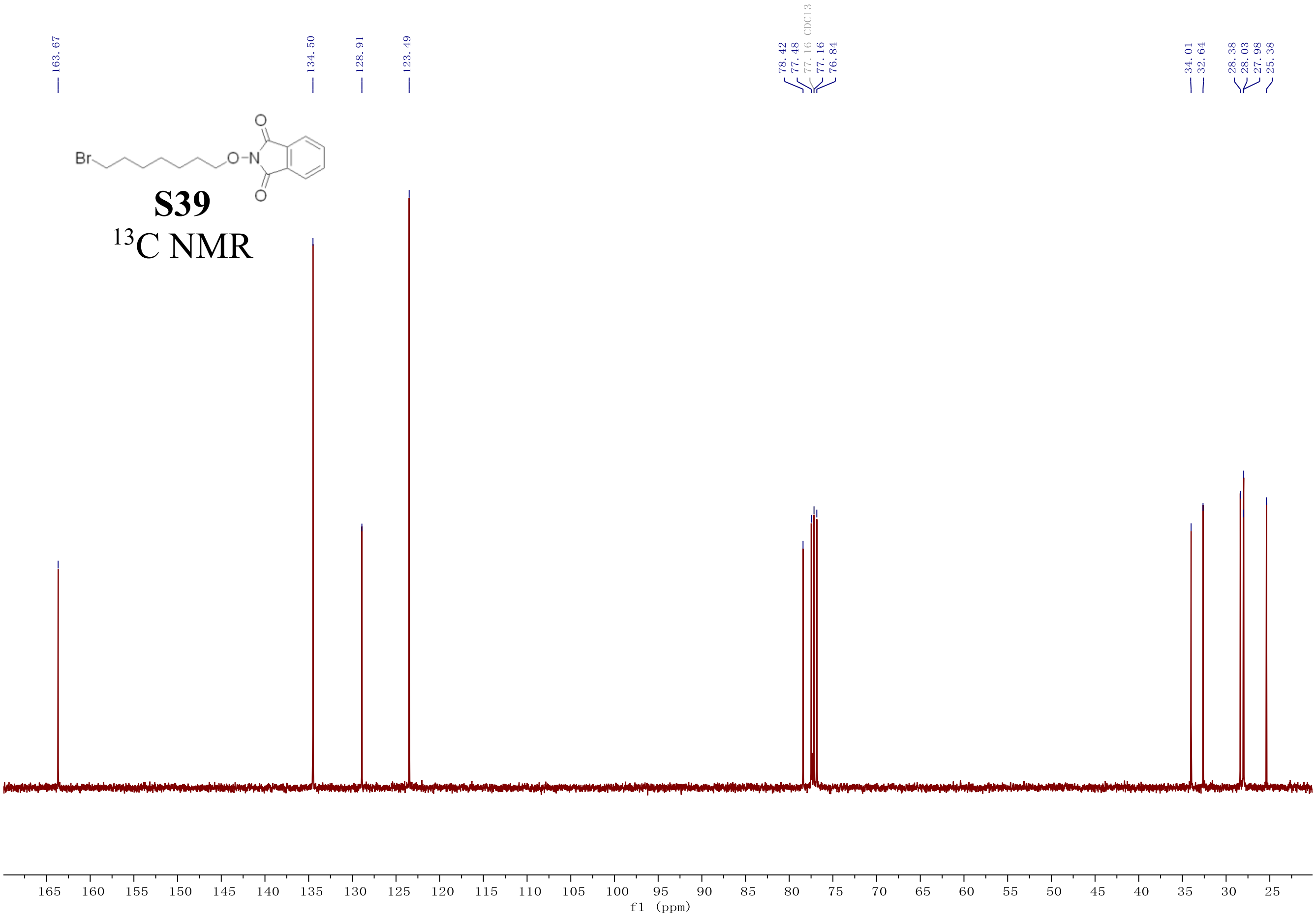
S39
13C NMR
163.67
134.50
128.91
123.49
78.42
77.48
77.16 CDCl3
77.16
76.84
34.01
32.64
28.38
28.03
27.98
25.38
f1 (ppm)

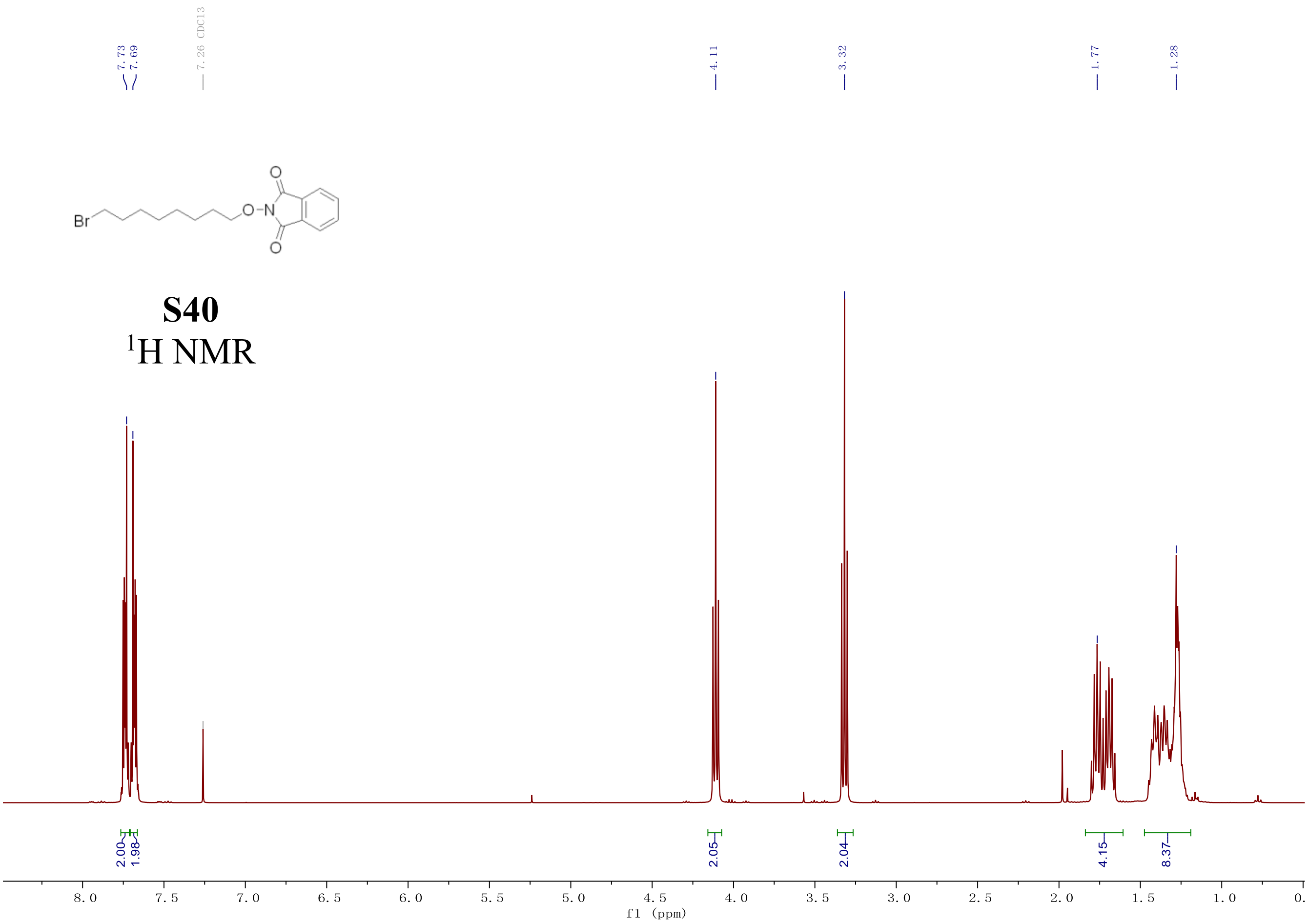
7.73
7.69
7.26 CDCl3
4.11
3.32
1.77
1.28
S40
^{1}H NMR
2.00
1.98
2.05
2.04
4.15
8.37
8.0 7.5 7.0 6.5 6.0 5.5 5.0 4.5 4.0 3.5 3.0 2.5 2.0 1.5 1.0
f1 (ppm)

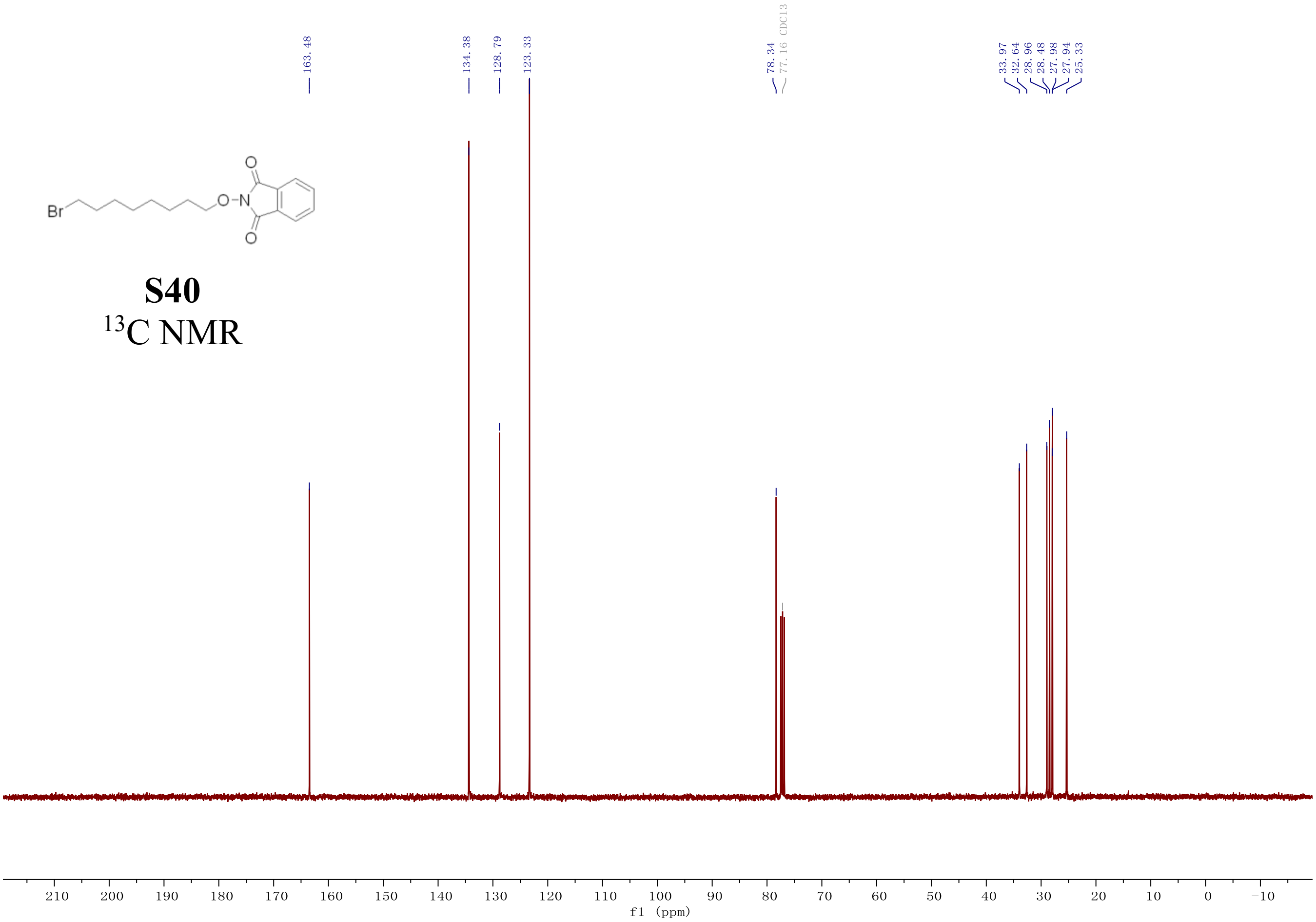
163.48
134.38
128.79
123.33
78.34
77.16 CDCl3
33.97
32.64
28.96
28.48
27.98
27.94
25.33
S40
^{13}C NMR
210 200 190 180 170 160 150 140 130 120 110 100 90 80 70 60 50 40 30 20 10 0 -10
f1 (ppm)

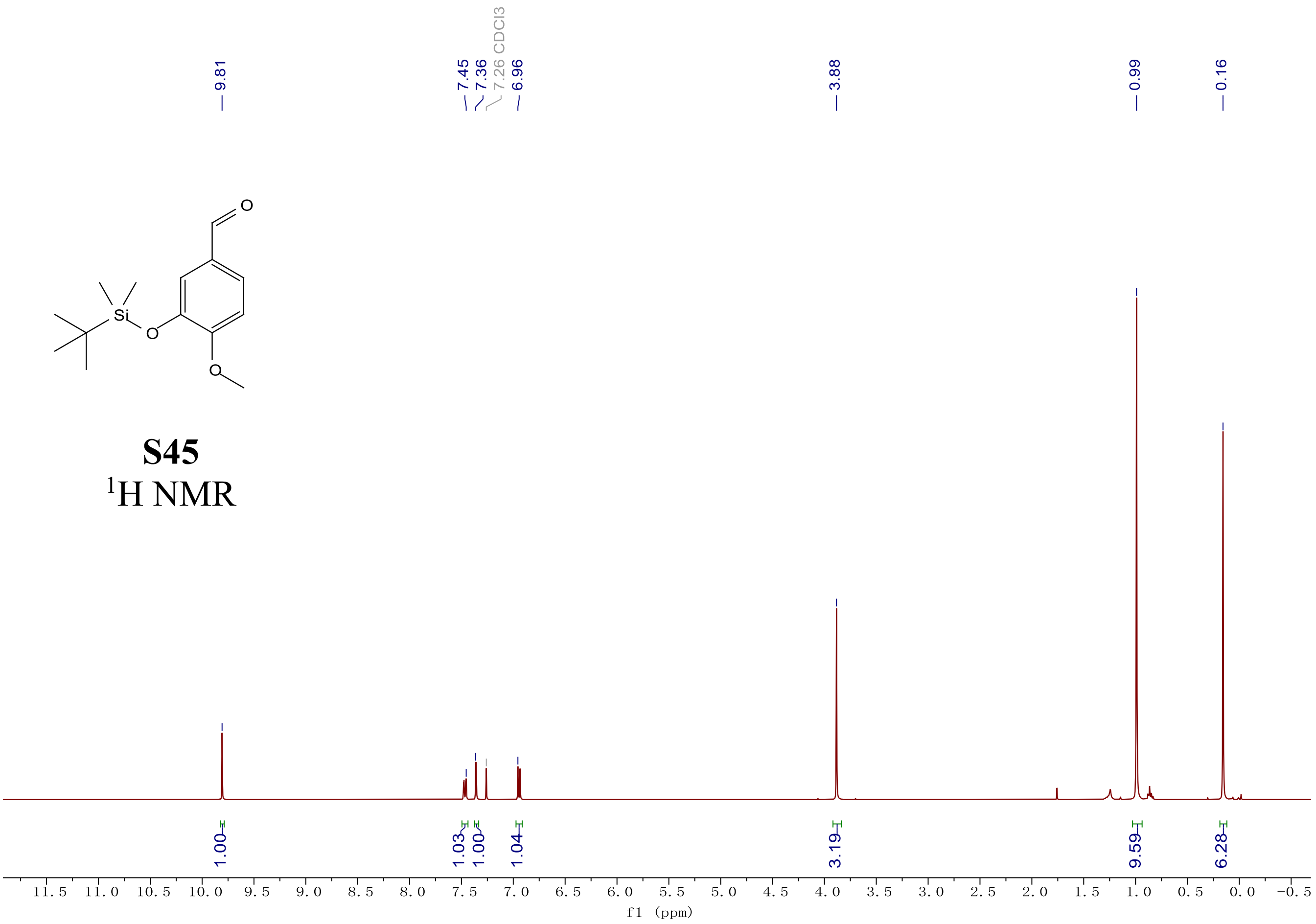

S45
^{1}H NMR
9.81
7.45
7.36
7.26 CDCl3
6.96
3.88
0.99
0.16
1.00
1.03
1.00
1.04
3.19
9.59
6.28
f1 (ppm)

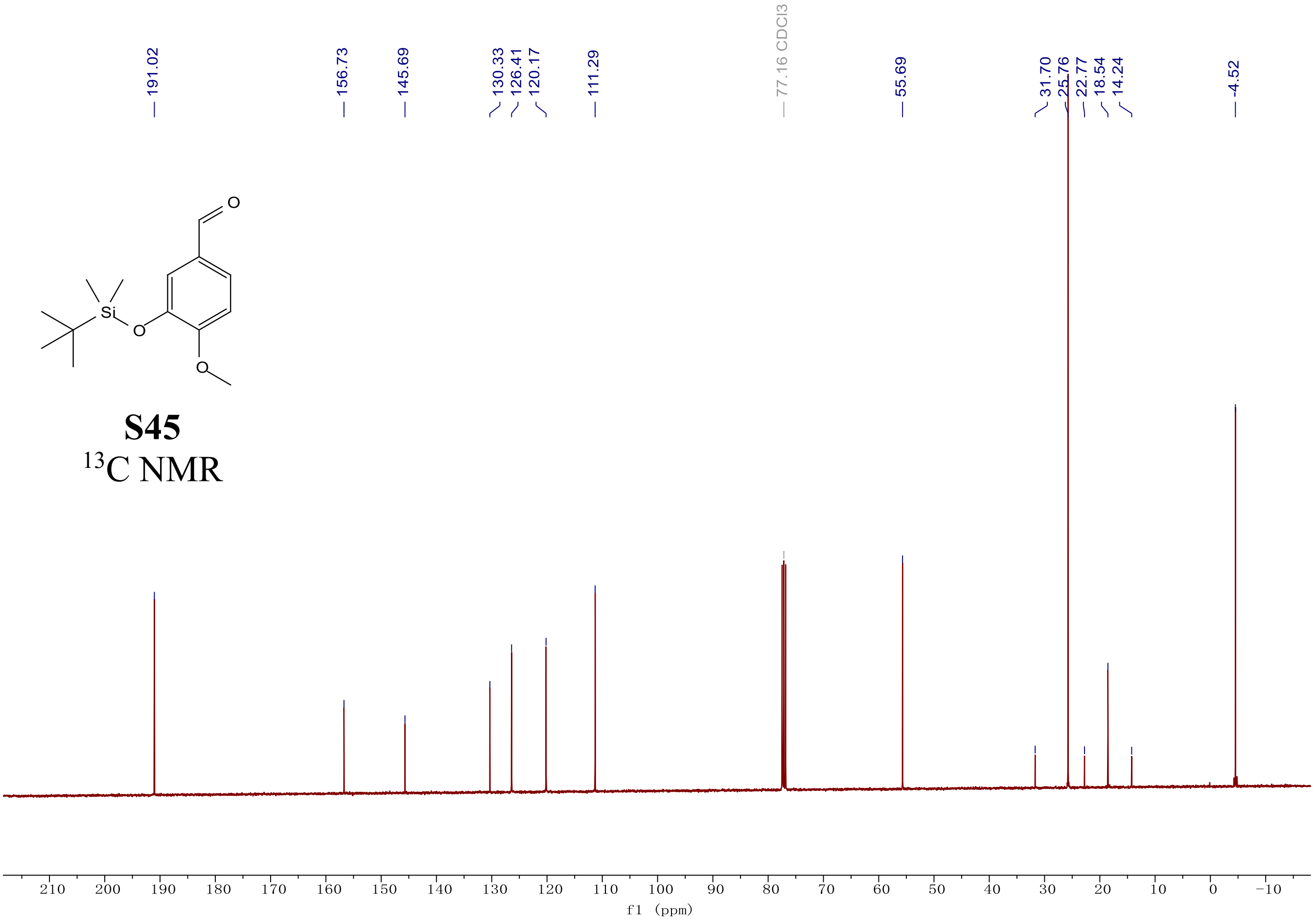

S45
^{13}C NMR
191.02
156.73
145.69
130.33
126.41
120.17
111.29
77.16 CDCl3
55.69
31.70
25.76
22.77
18.54
14.24
-4.52
f1 (ppm)

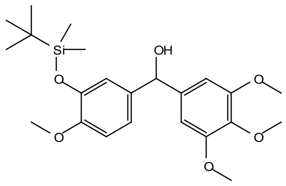

S46
^{1}H NMR

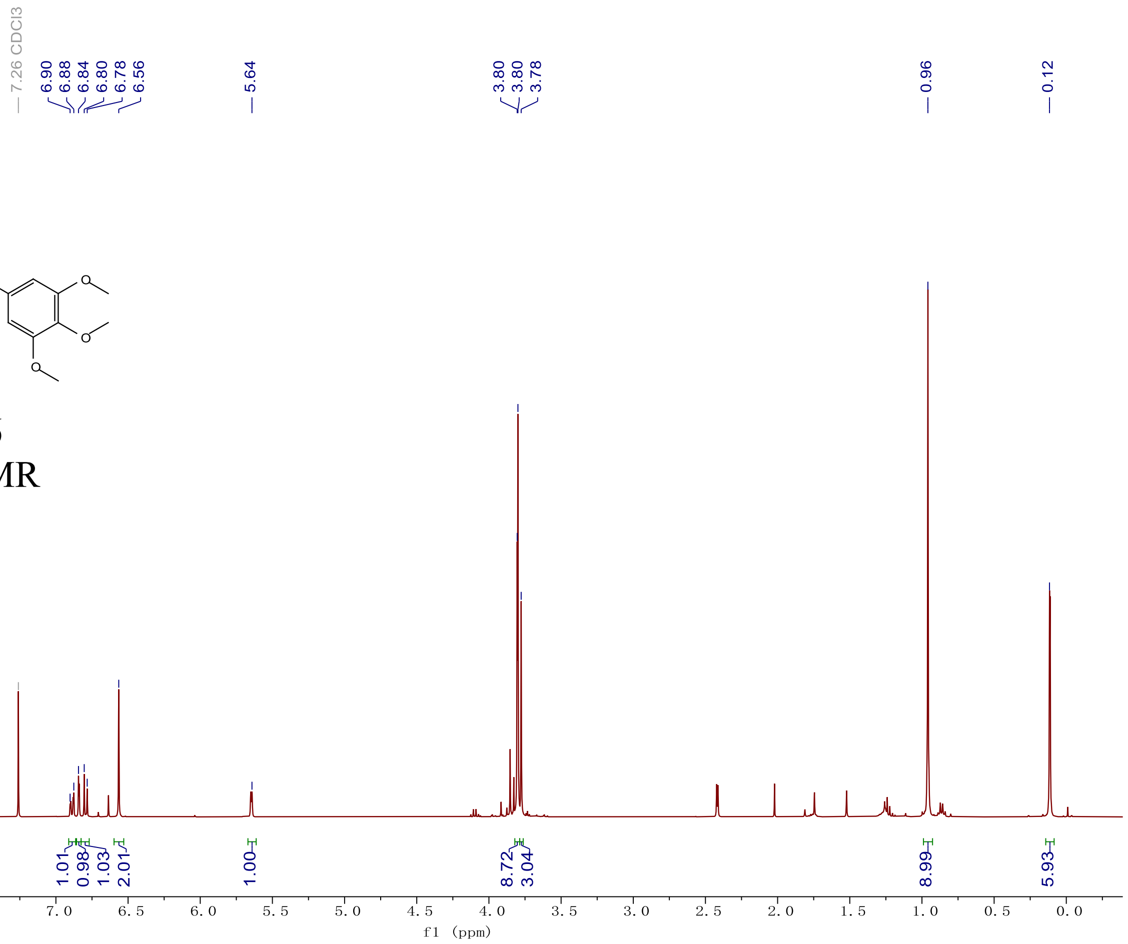

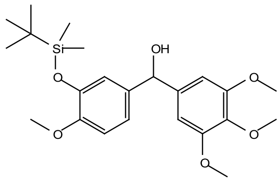

S46
^{13}C NMR

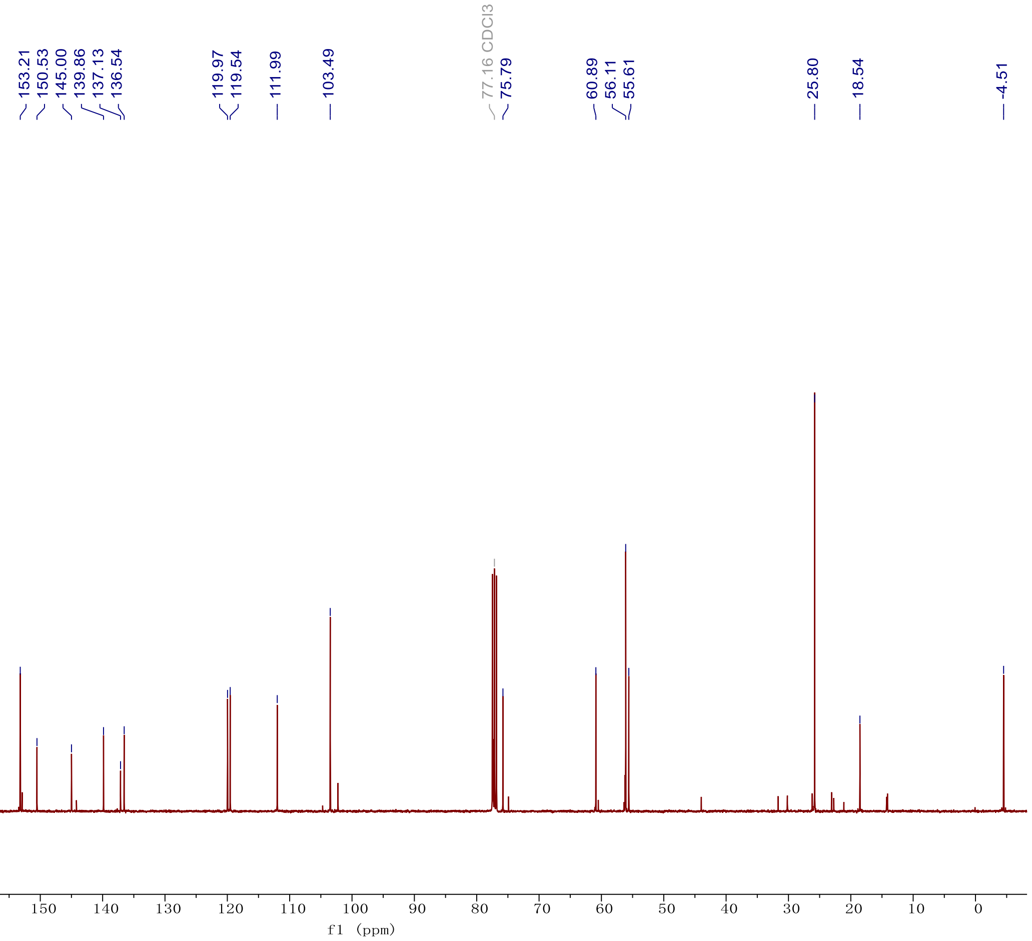

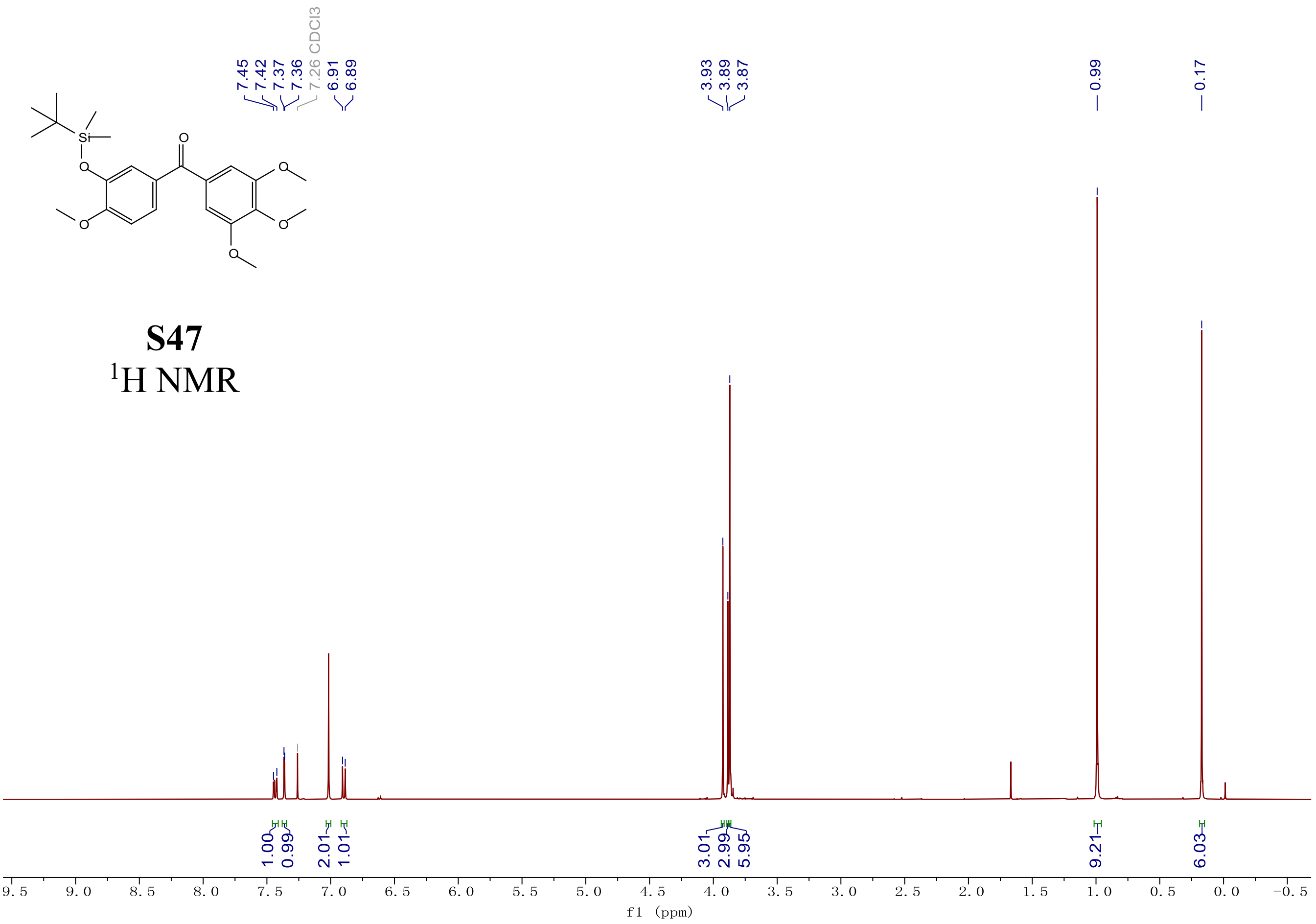

S47
^{1}H NMR
7.45
7.42
7.37
7.36
7.26 CDCl3
6.91
6.89
3.93
3.89
3.87
0.99
0.17
1.00
0.99
2.01
1.01
3.01
2.99
5.95
9.21
6.03
f1 (ppm)

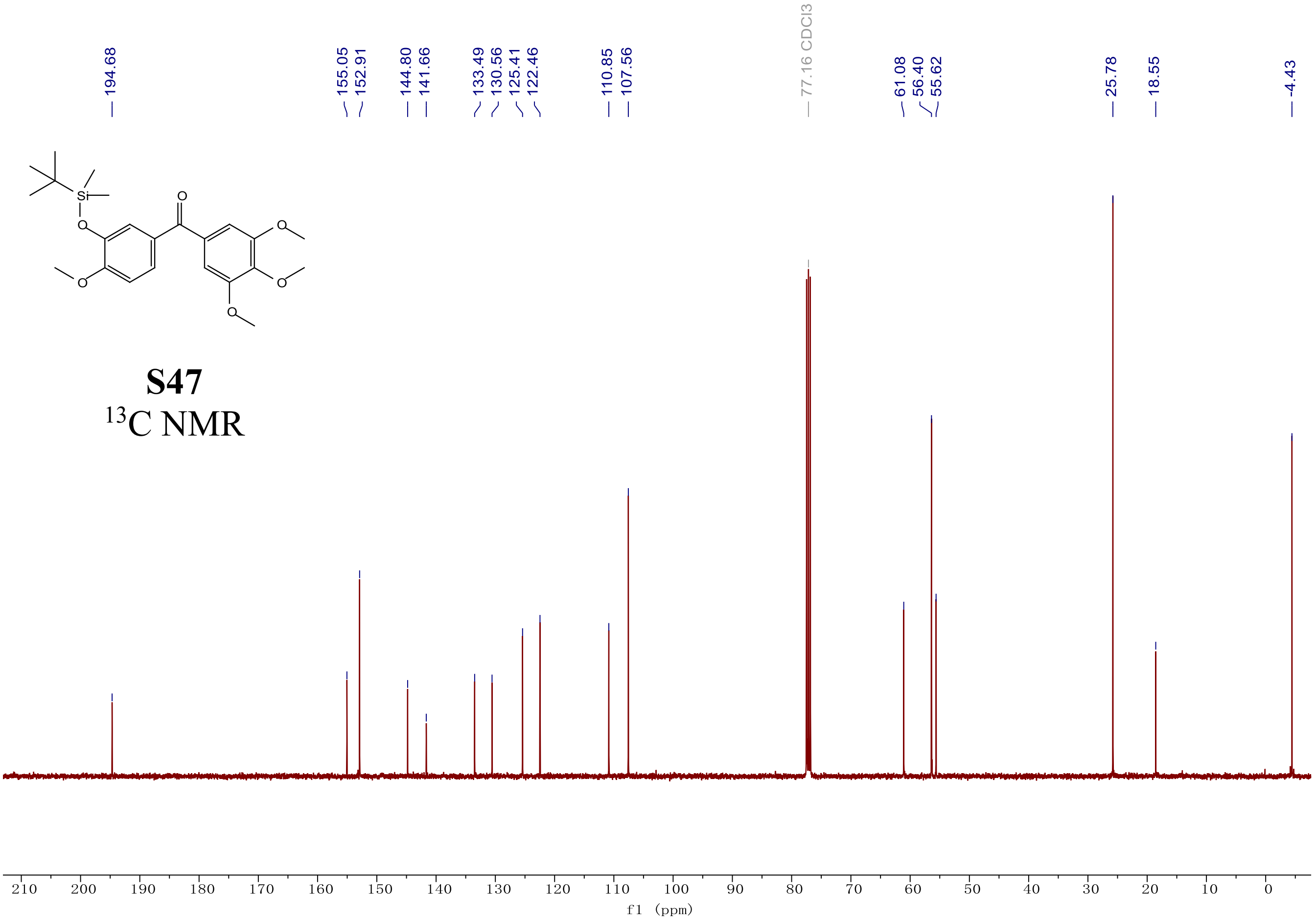

S47
^{13}C NMR
194.68
155.05
152.91
144.80
141.66
133.49
130.56
125.41
122.46
110.85
107.56
77.16 CDCl3
61.08
56.40
55.62
25.78
18.55
-4.43
f1 (ppm)

S48

^{1}H NMR

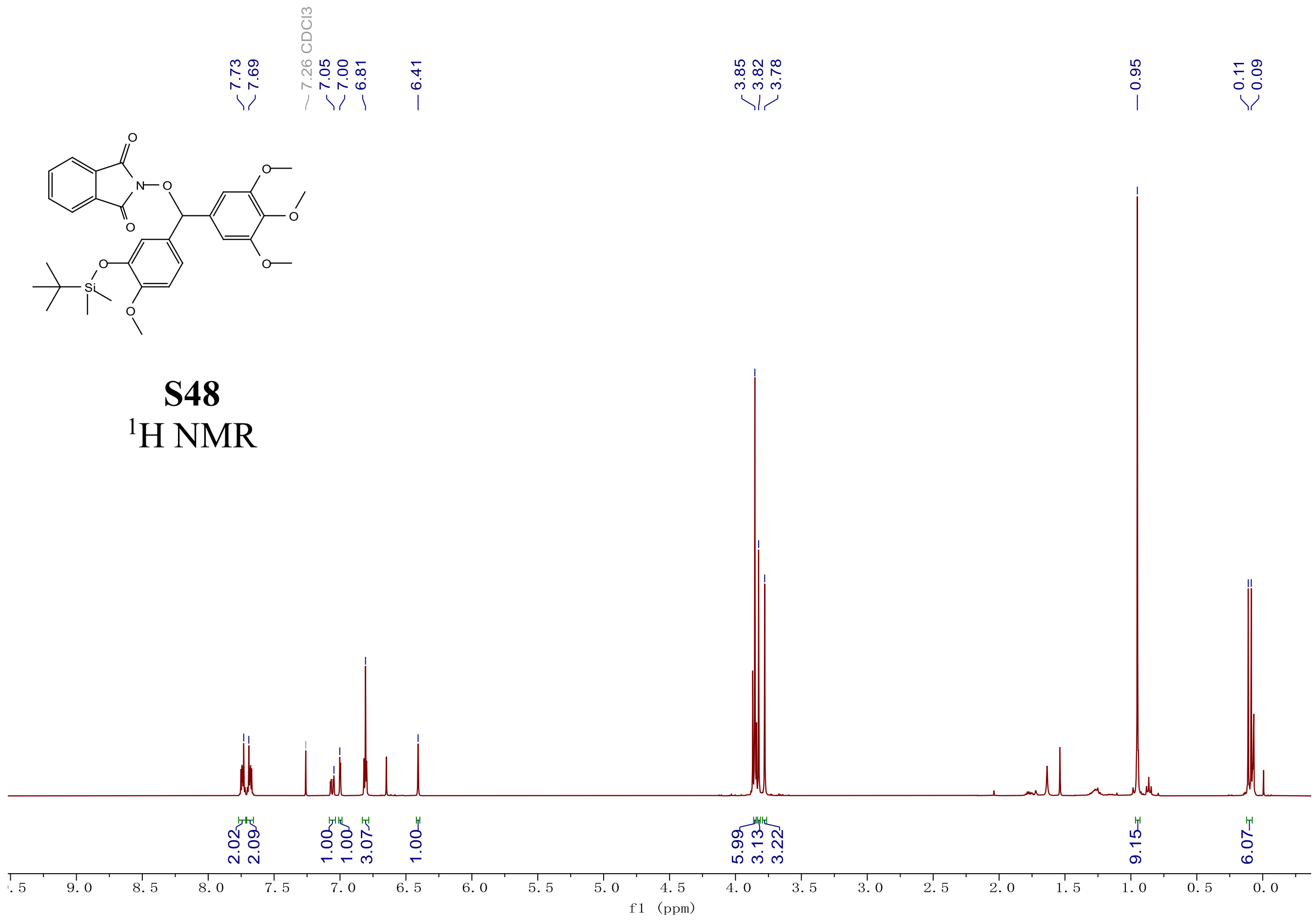

S48

^{13}C NMR

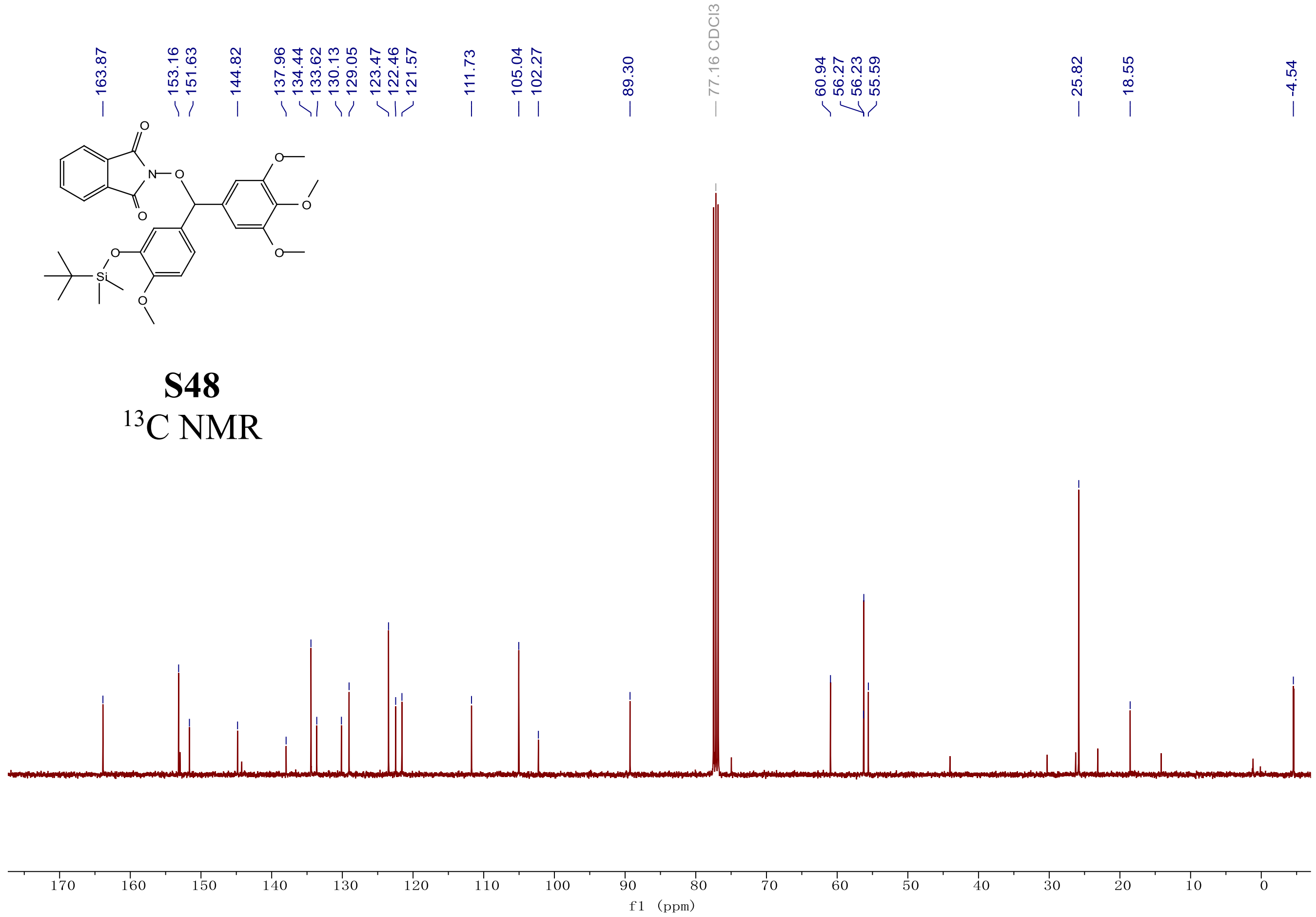

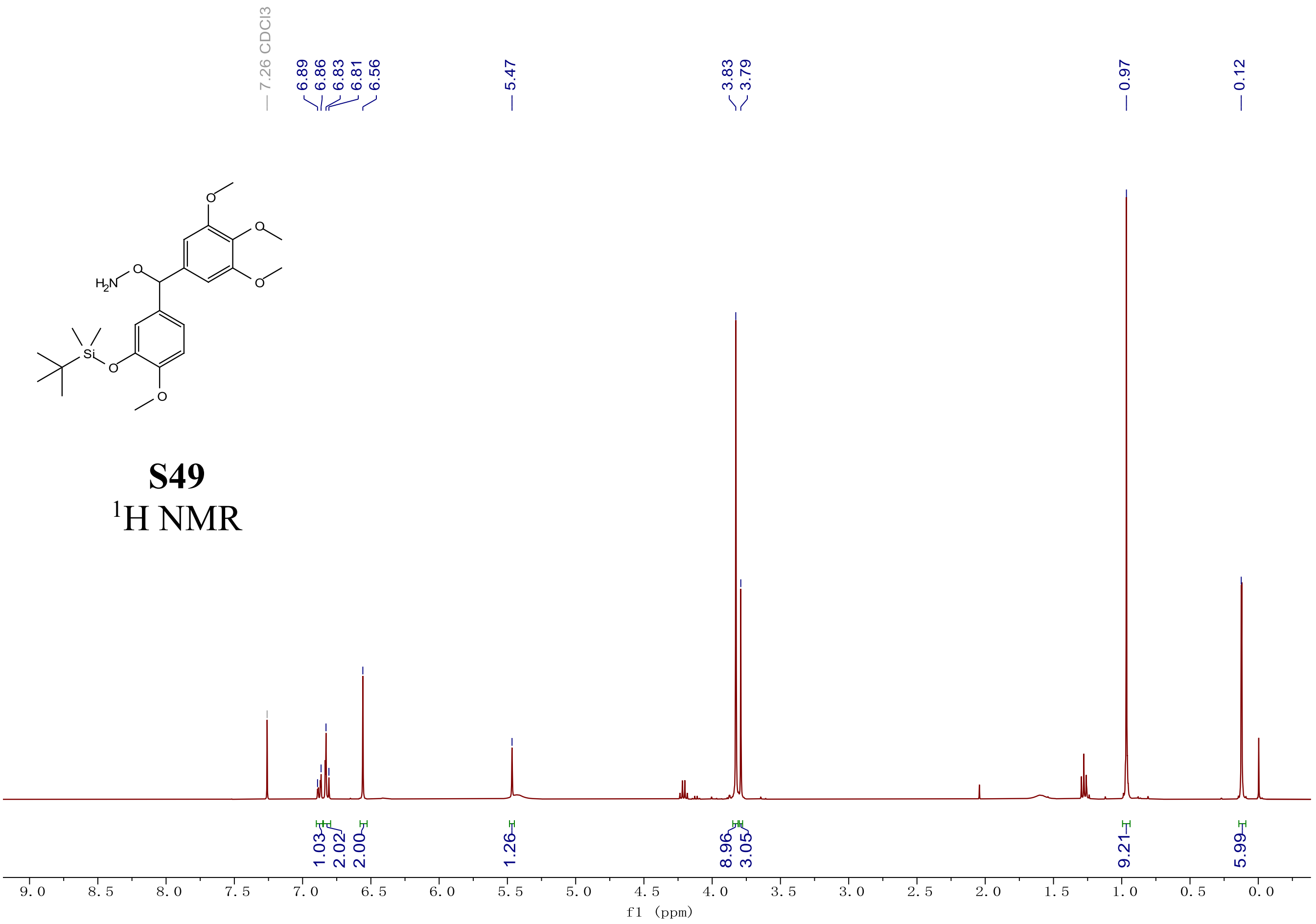

7.26 CDCl3
6.89
6.86
6.83
6.81
6.56
5.47
3.83
3.79
0.97
0.12
H2N
O
Si
S49
1H NMR
1.03
2.02
2.00
1.26
8.96
3.05
9.21
5.99
9.0 8.5 8.0 7.5 7.0 6.5 6.0 5.5 5.0 4.5 4.0 3.5 3.0 2.5 2.0 1.5 1.0 0.5 0.0
f1 (ppm)

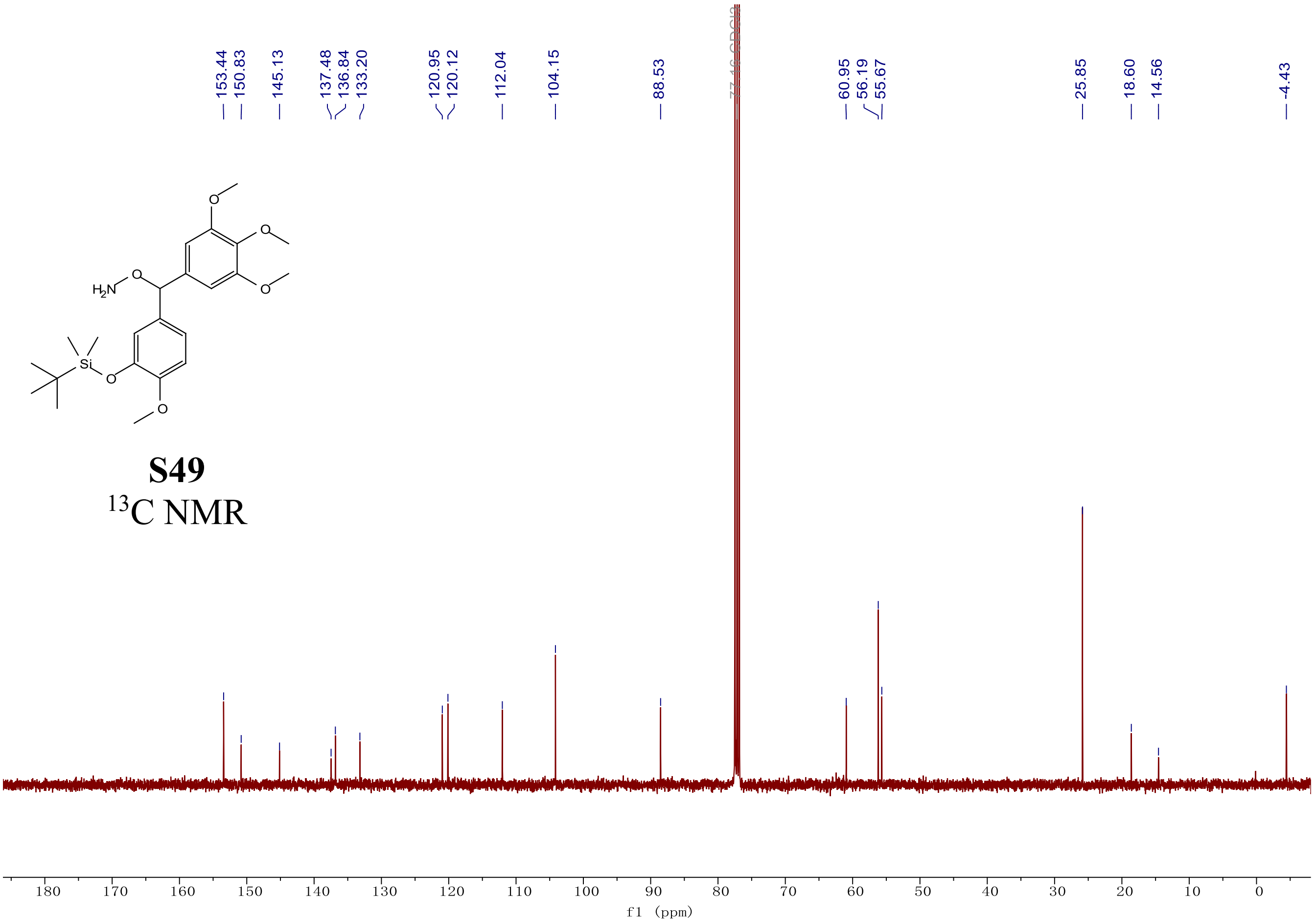

153.44
150.83
145.13
137.48
136.84
133.20
120.95
120.12
112.04
104.15
88.53
60.95
56.19
55.67
25.85
18.60
14.56
-4.43
H2N
O
Si
S49
13C NMR
180 170 160 150 140 130 120 110 100 90 80 70 60 50 40 30 20 10 0
f1 (ppm)

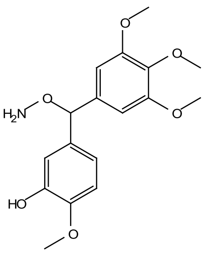

S50

^{1}H NMR

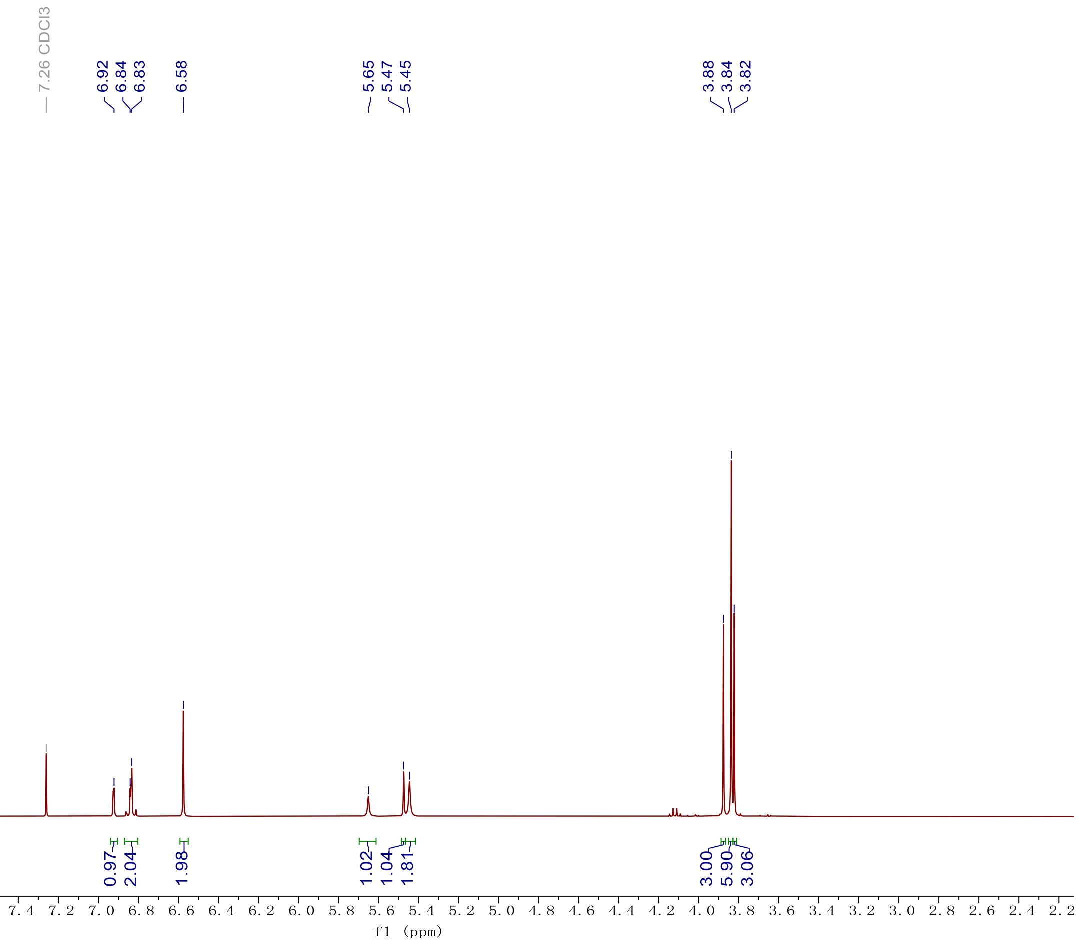

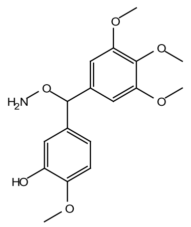

S50

^{13}C NMR

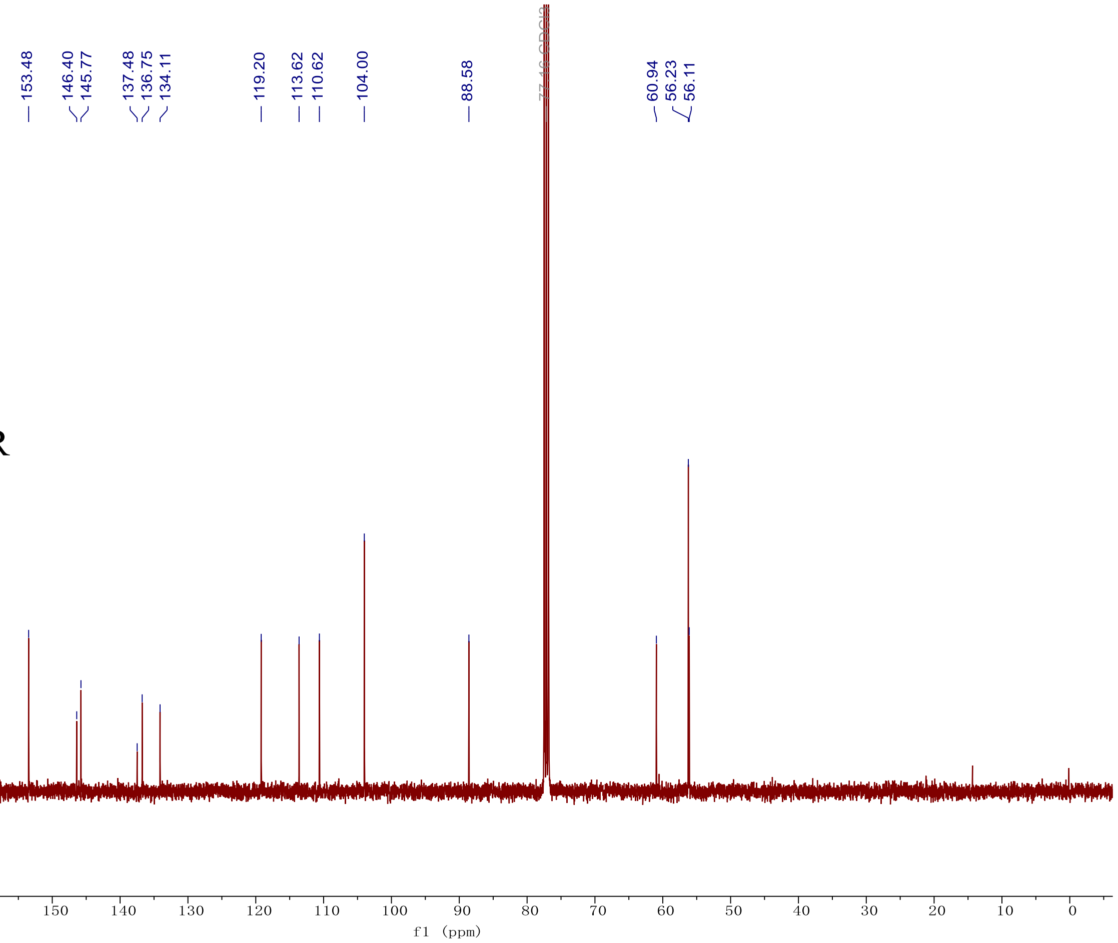

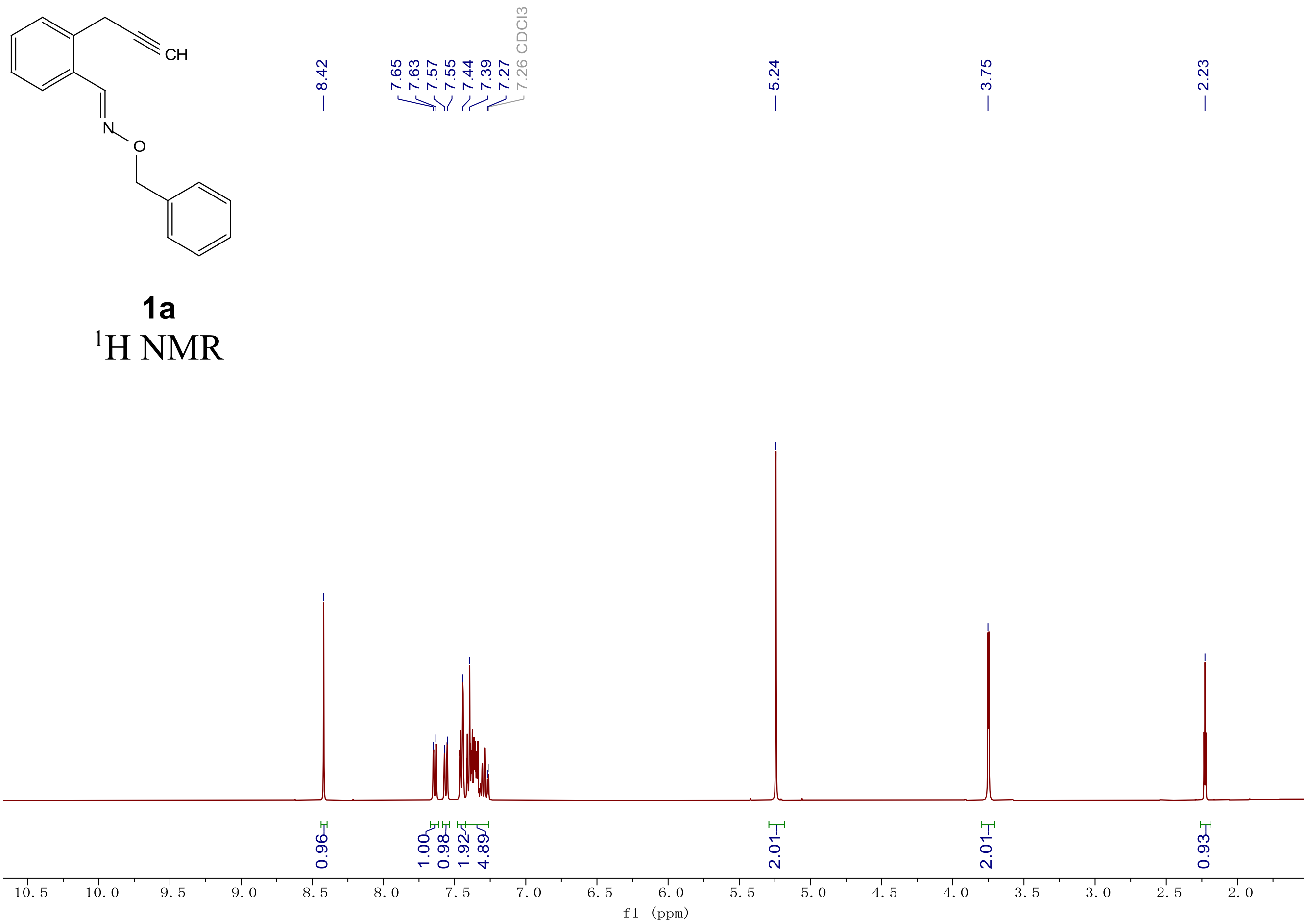

CH
1a
^{1}H NMR
8.42
7.65 7.63 7.57 7.55 7.44 7.39 7.27
7.26 CDCl3
5.24
3.75
2.23
0.96
1.00 0.98 1.92 4.89
2.01
2.01
0.93
10.5 10.0 9.5 9.0 8.5 8.0 7.5 7.0 6.5 6.0 5.5 5.0 4.5 4.0 3.5 3.0 2.5 2.0
f1 (ppm)

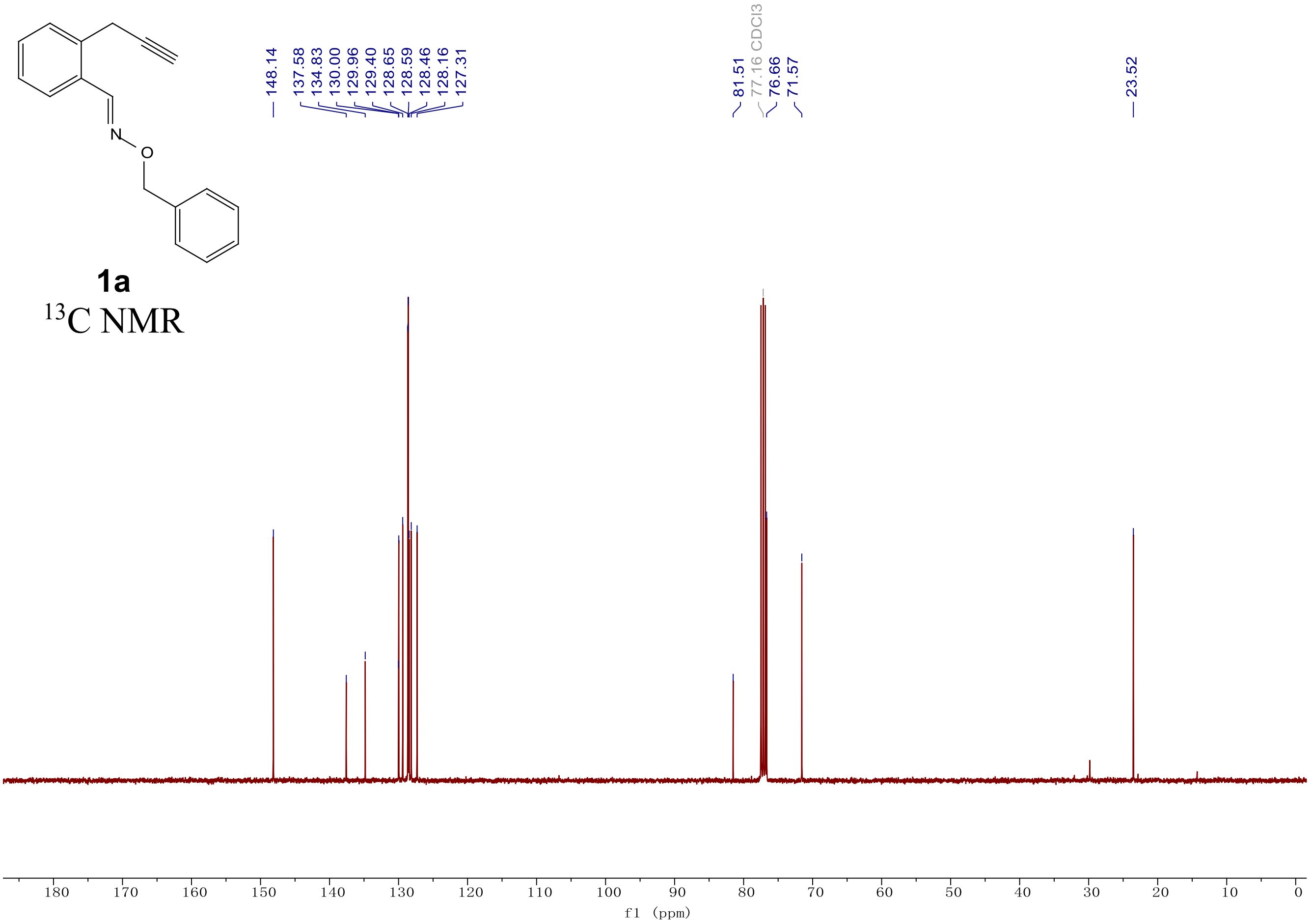

1a
^{13}C NMR
148.14
137.58 134.83 130.00 129.96 129.40 128.65 128.59 128.46 128.16 127.31
81.51
77.16 CDCl3
76.66
71.57
23.52
180 170 160 150 140 130 120 110 100 90 80 70 60 50 40 30 20 10 0
f1 (ppm)

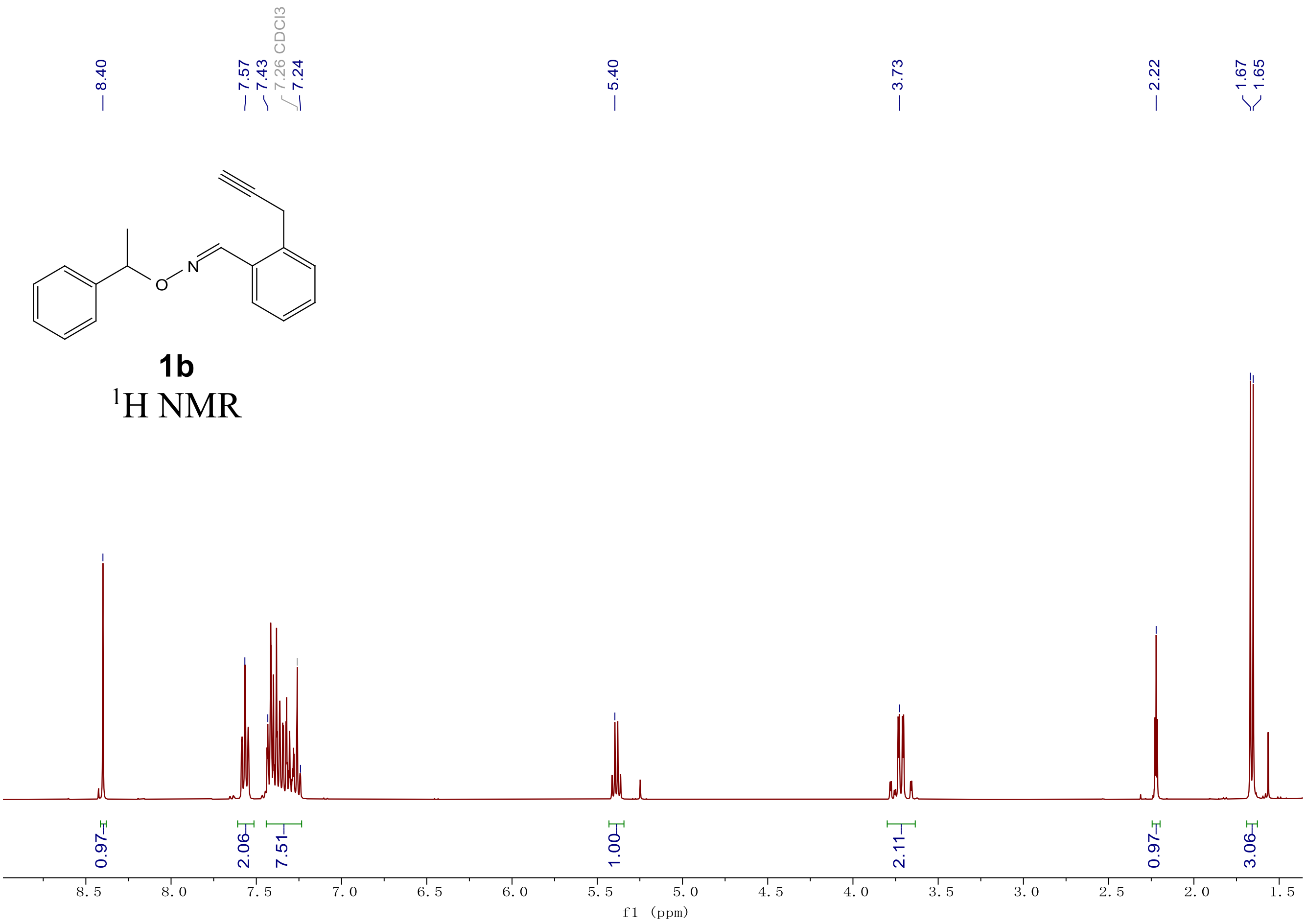
8.40
7.57
7.43
7.26 CDCl3
7.24
5.40
3.73
2.22
1.67
1.65
N
O
1b
¹H NMR
0.97
2.06
7.51
1.00
2.11
0.97
3.06
8.5
8.0
7.5
7.0
6.5
6.0
5.5
5.0
4.5
4.0
3.5
3.0
2.5
2.0
1.5
f1 (ppm)

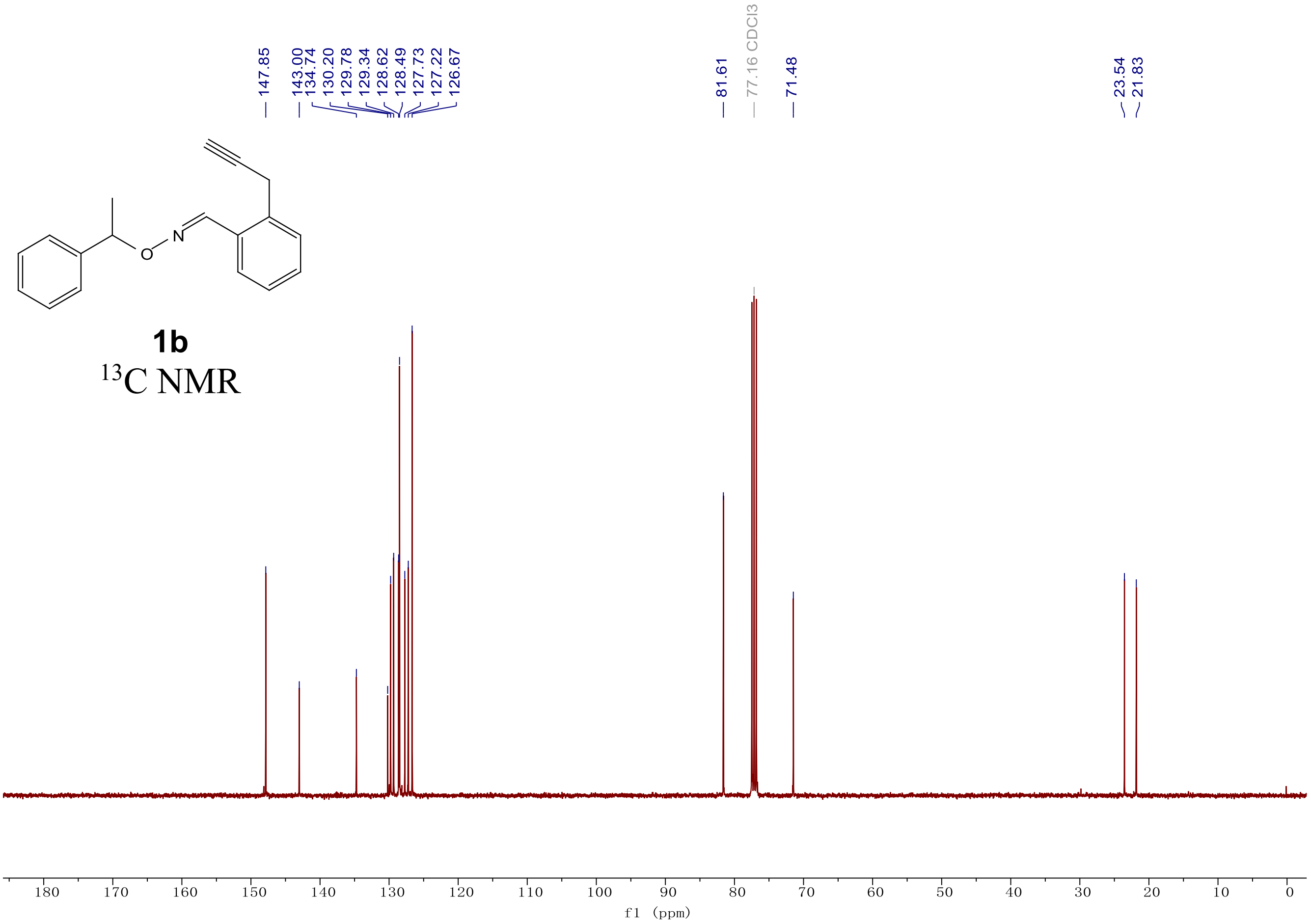
147.85
143.00
134.74
130.20
129.78
129.34
128.62
128.49
127.73
127.22
126.67
81.61
77.16 CDCl3
71.48
23.54
21.83
N
O
1b
¹³C NMR
180
170
160
150
140
130
120
110
100
90
80
70
60
50
40
30
20
10
0
f1 (ppm)

1c
^{1}H NMR

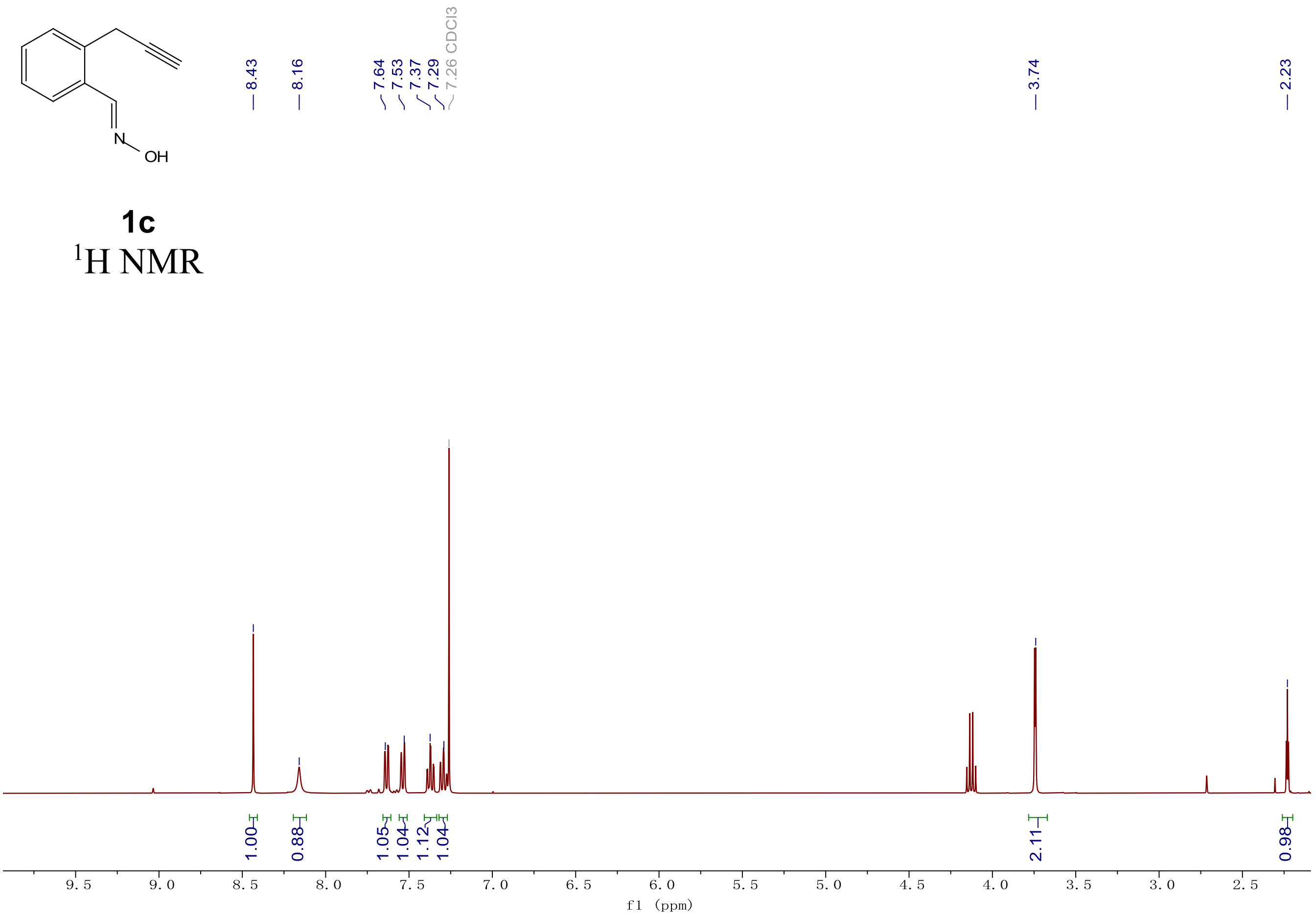

1c
^{13}C NMR

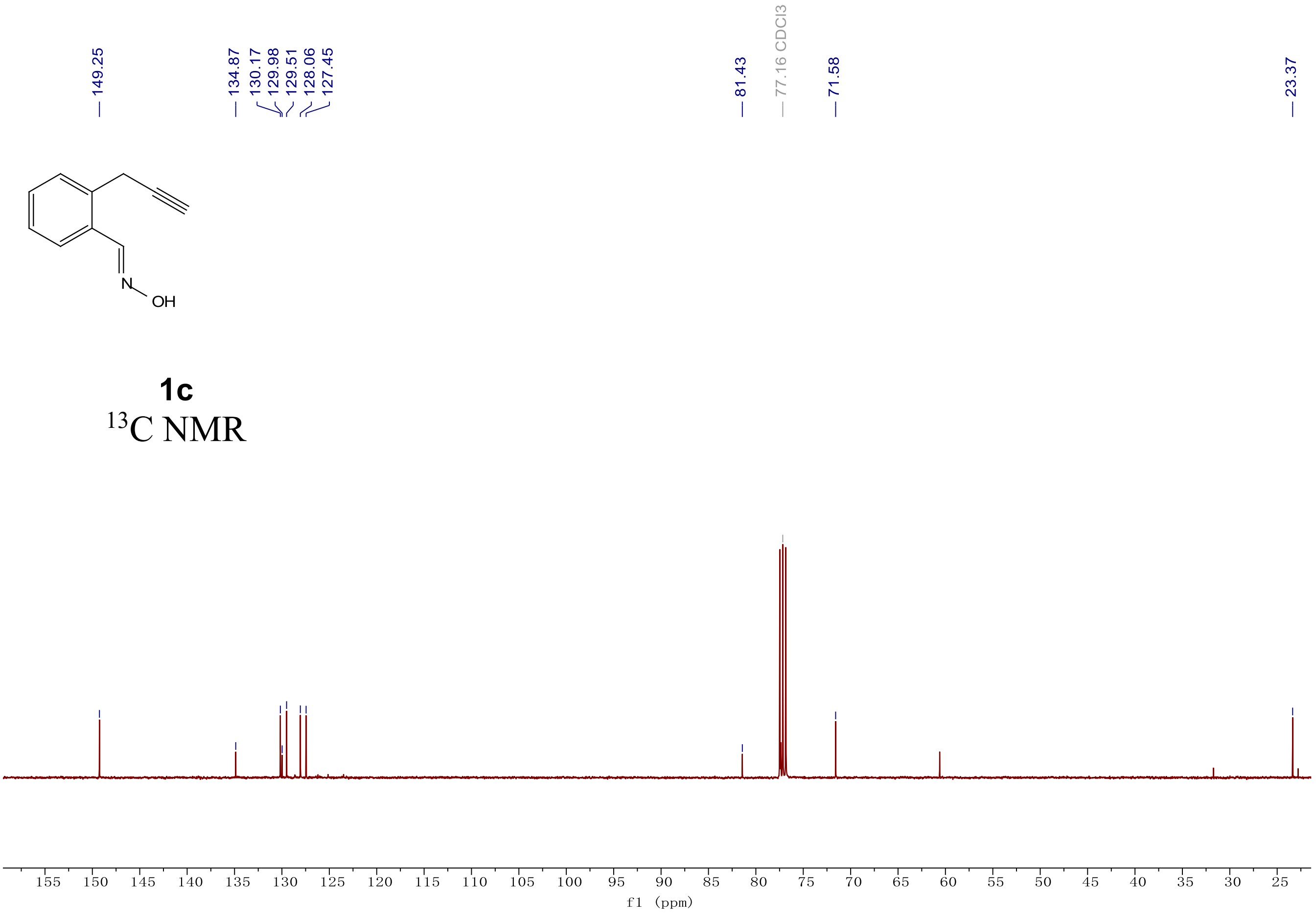

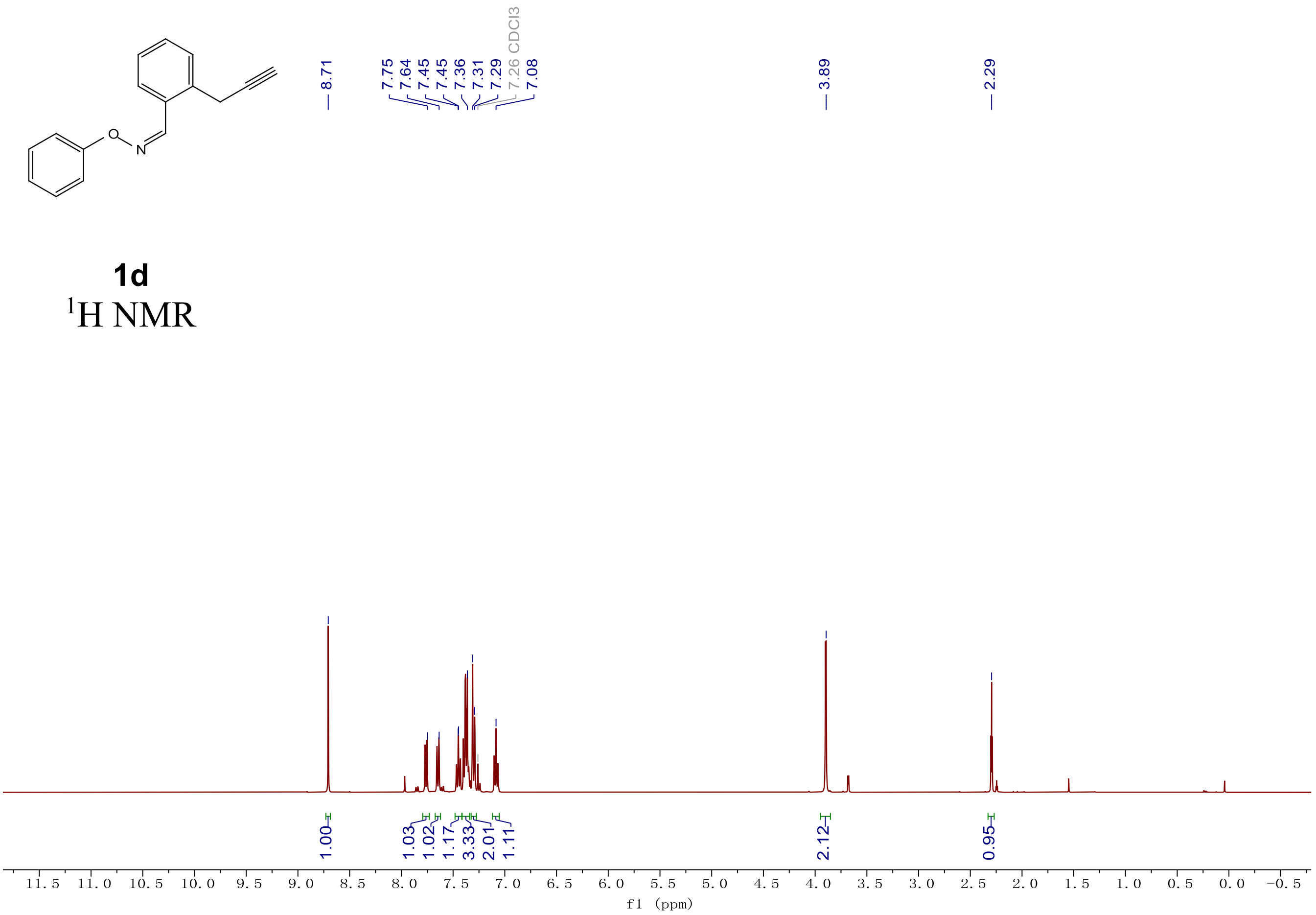

1d
^{1}H NMR

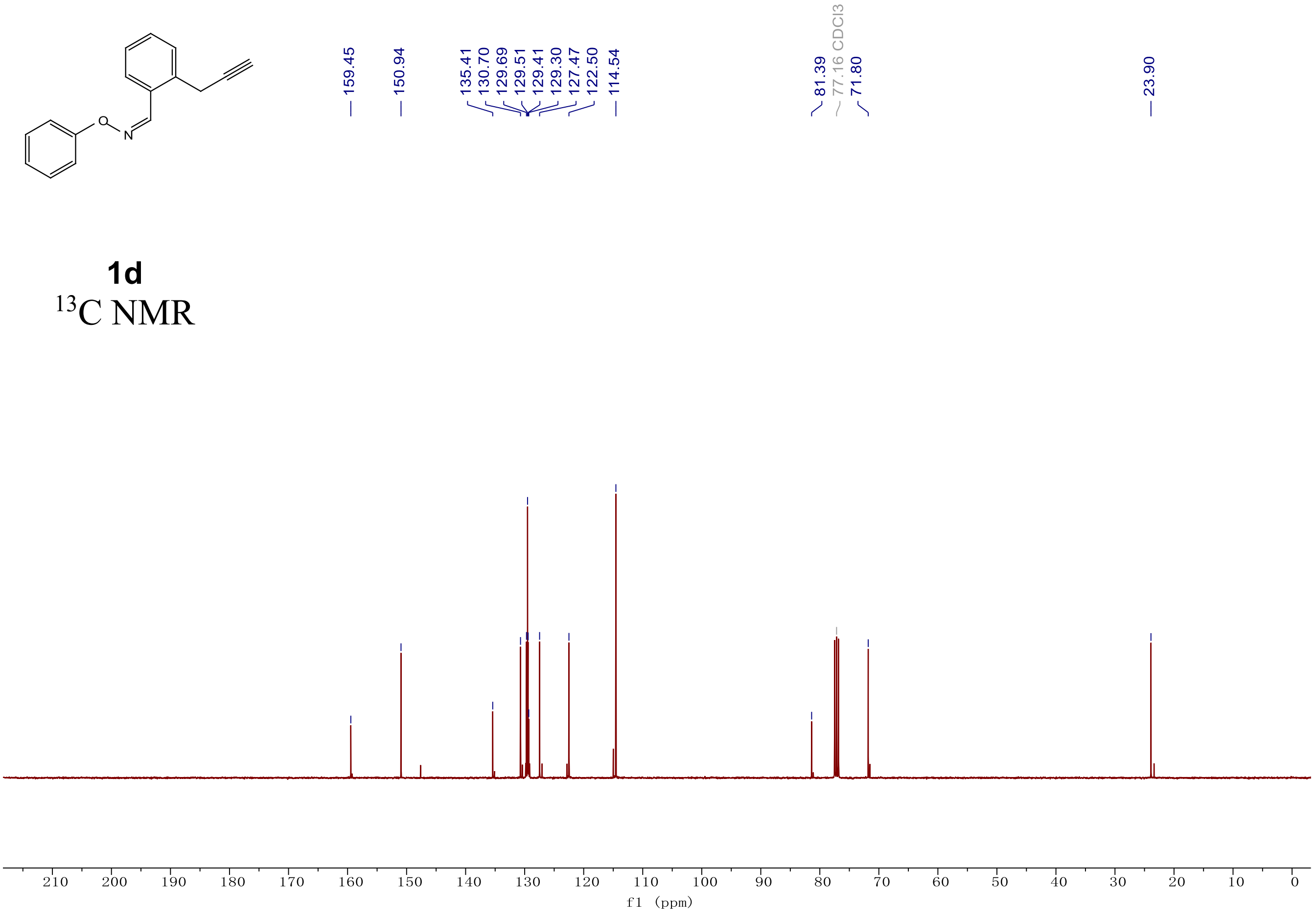

1d
^{13}C NMR

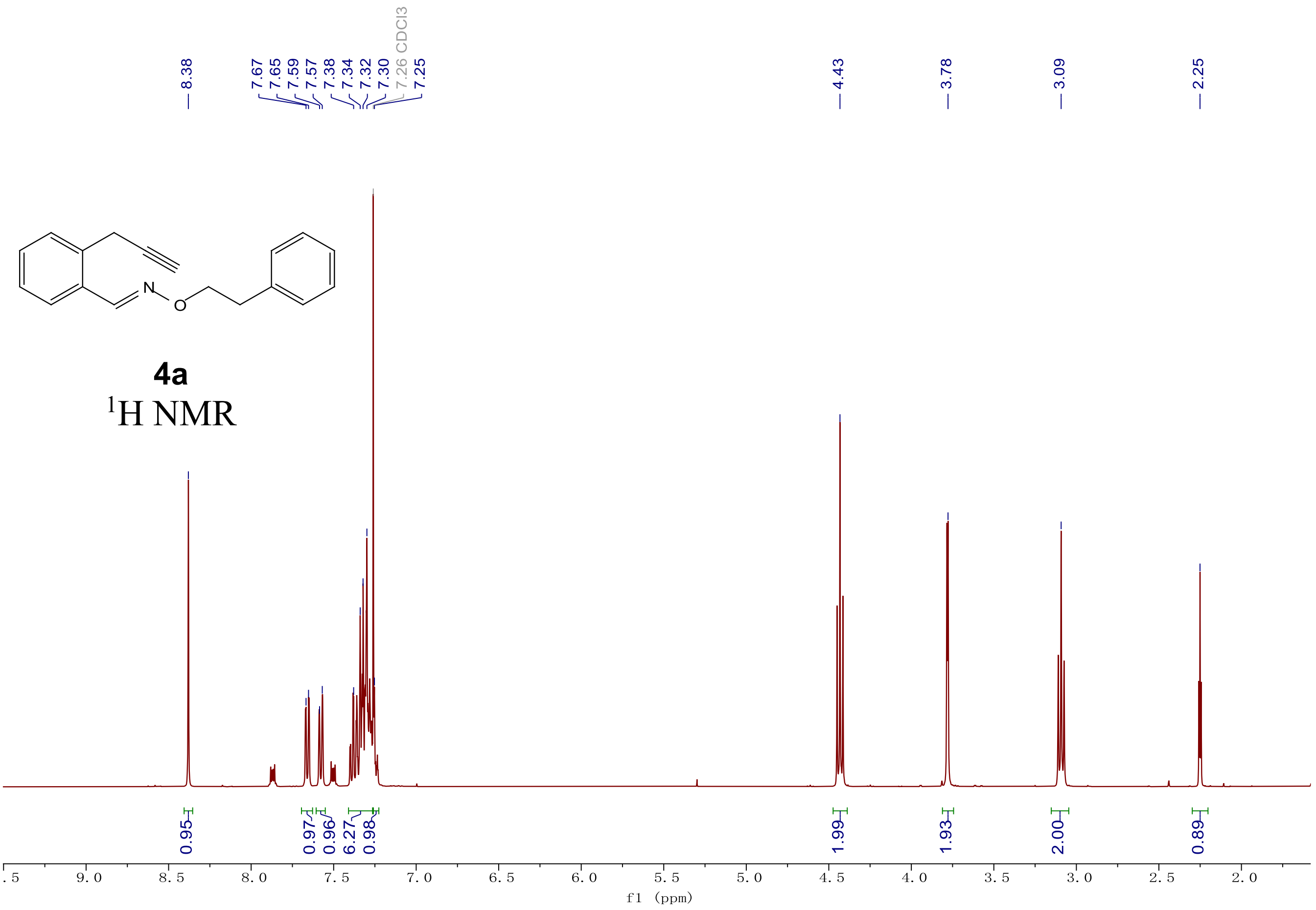

4a

^{1}H NMR

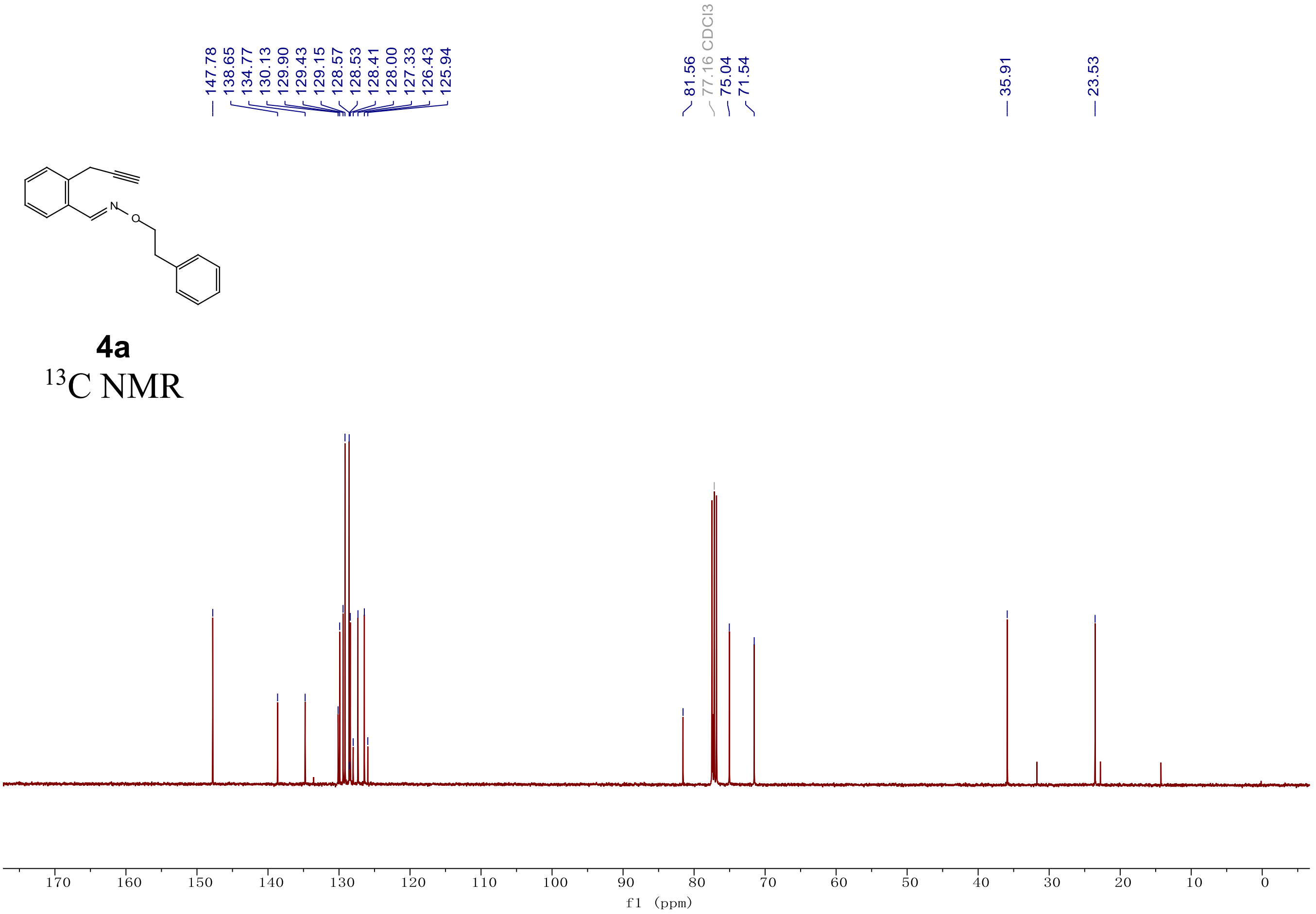

4a

^{13}C NMR

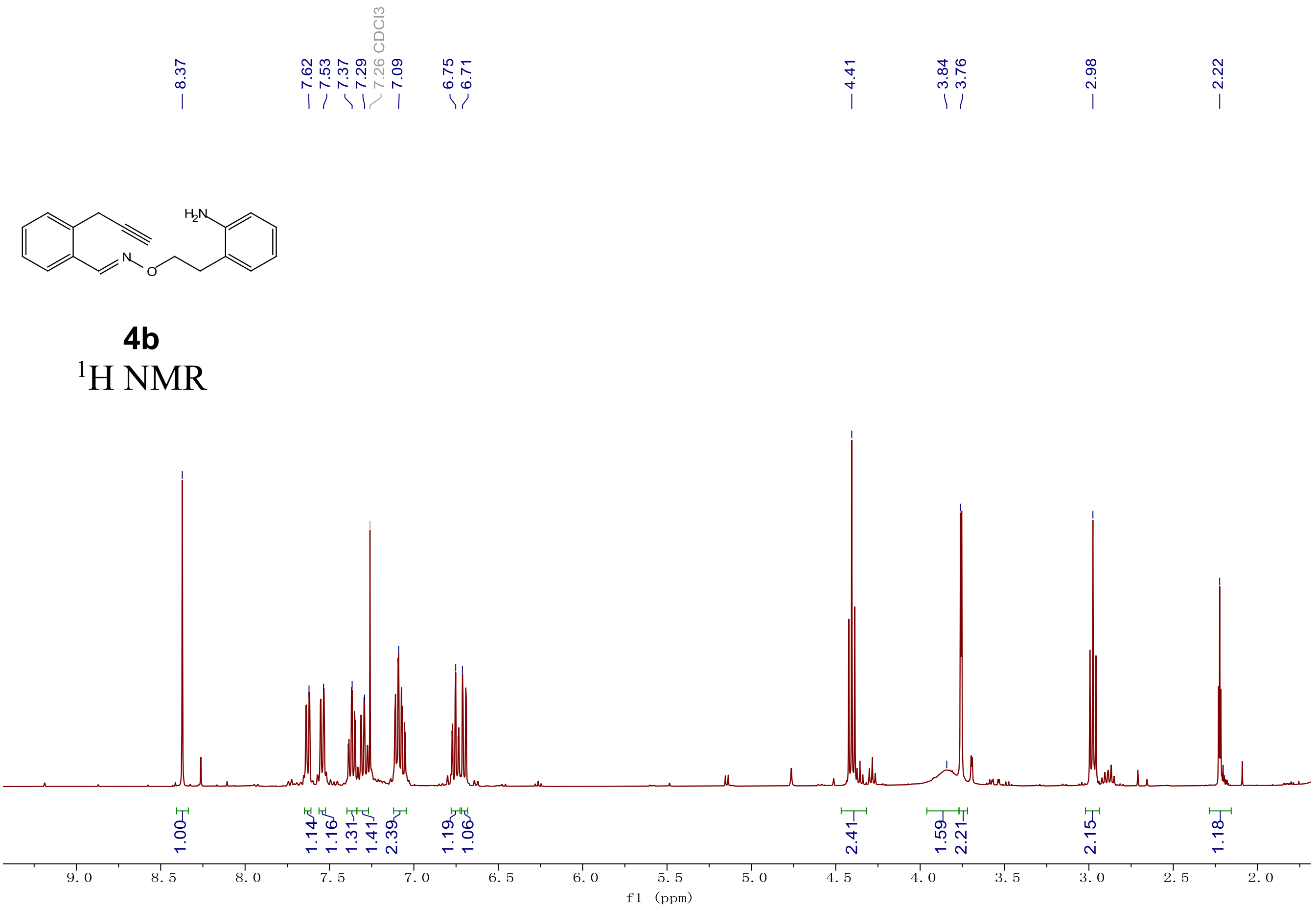

4b
^{1}H NMR

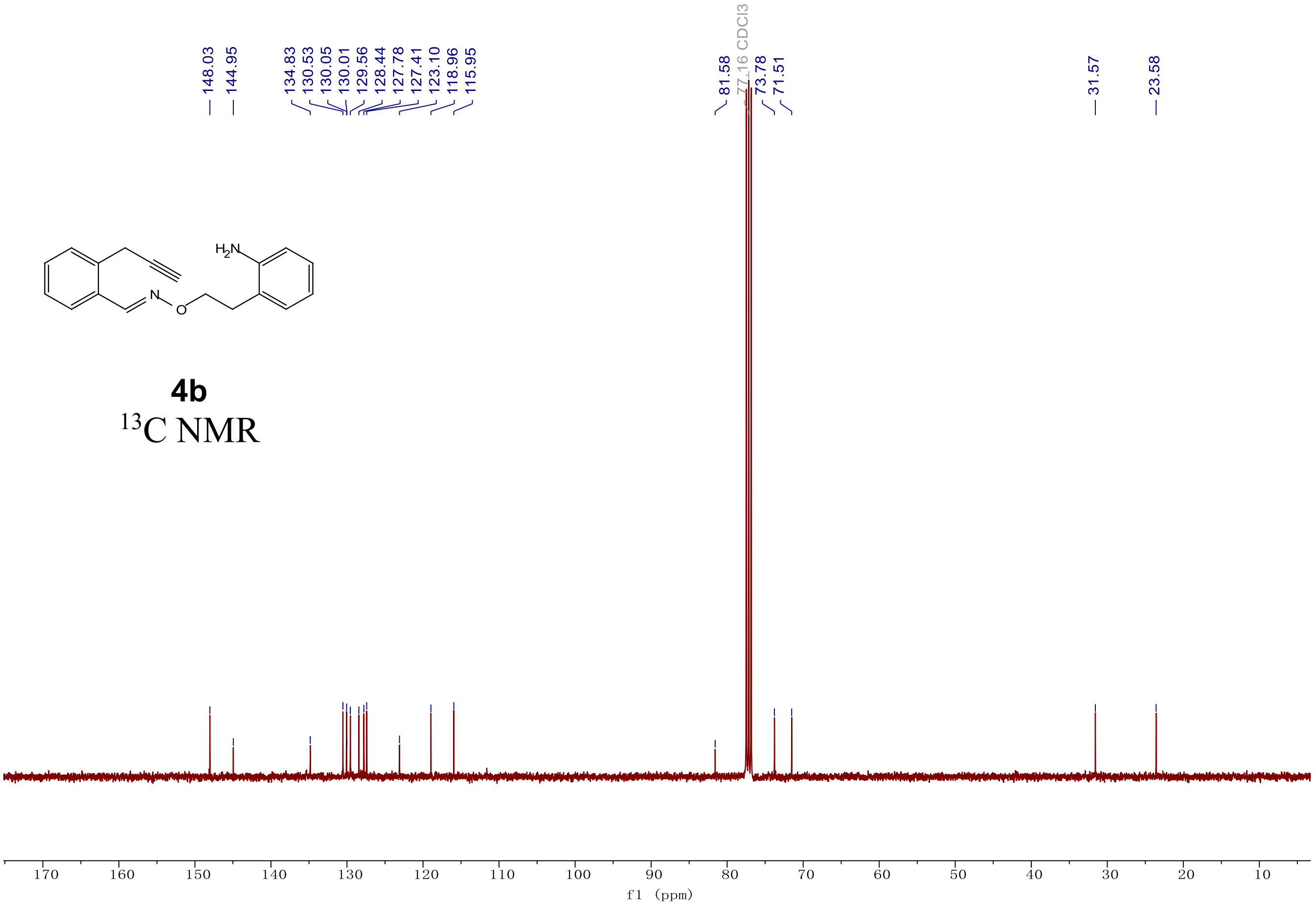

4b
^{13}C NMR

4c
^{1}H NMR

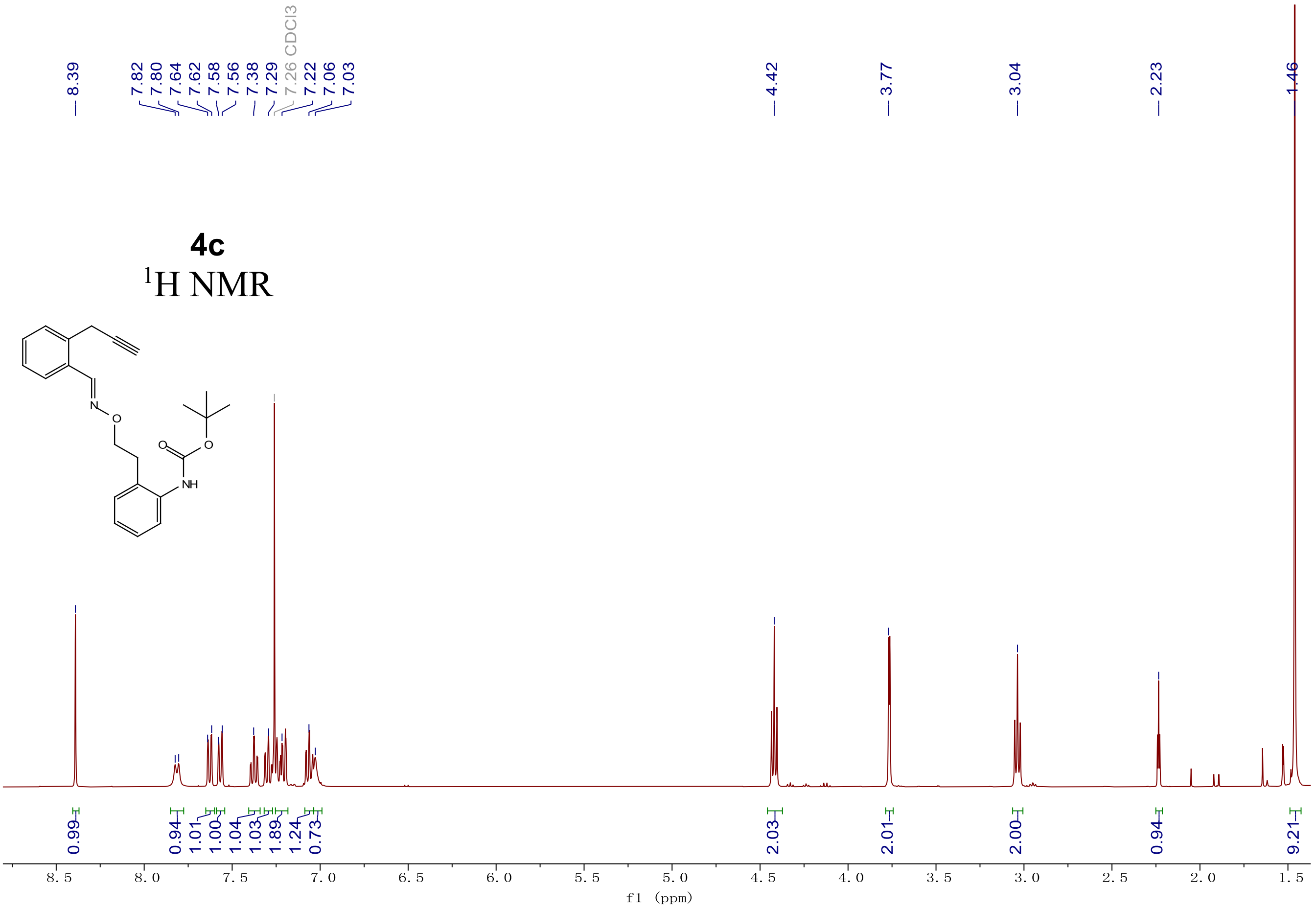

4c
^{13}C NMR

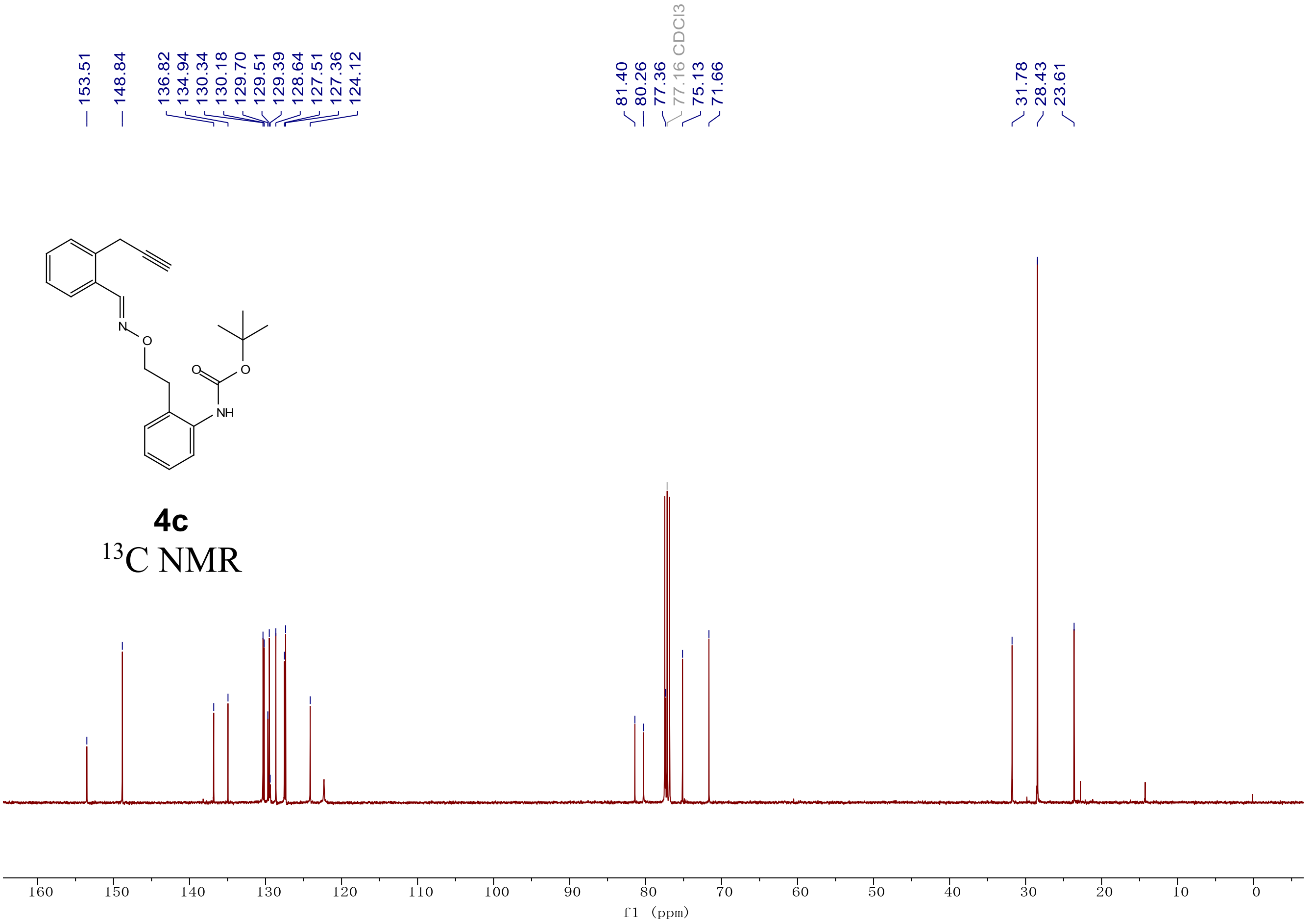

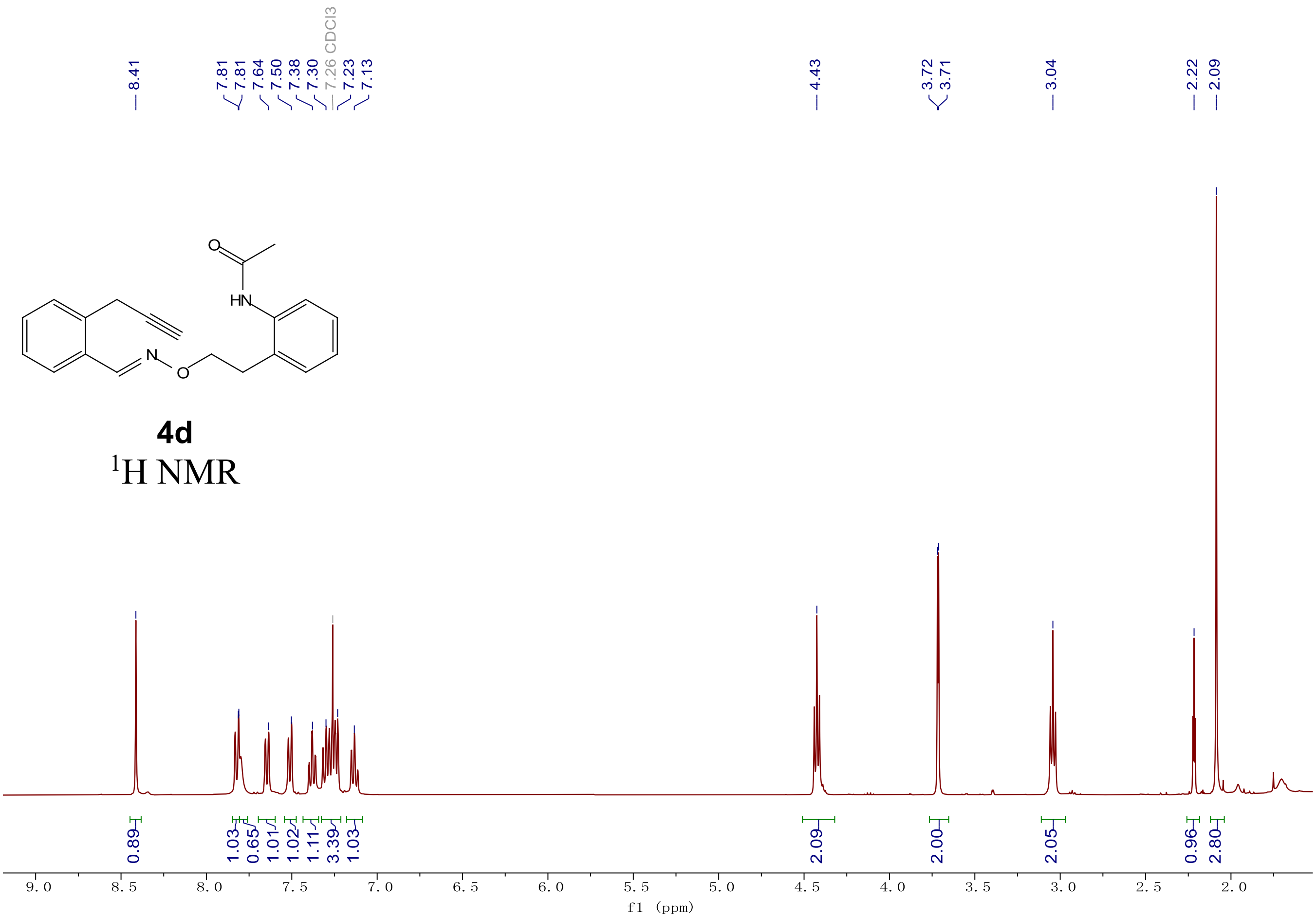
8.41
7.81
7.81
7.64
7.50
7.38
7.30
7.26 CDCl3
7.23
7.13
4.43
3.72
3.71
3.04
2.22
2.09
O
HN
N
O
4d
1H NMR
0.89
1.03
0.65
1.01
1.02
1.11
3.39
1.03
2.09
2.00
2.05
0.96
2.80
9.0
8.5
8.0
7.5
7.0
6.5
6.0
5.5
5.0
4.5
4.0
3.5
3.0
2.5
2.0
f1 (ppm)

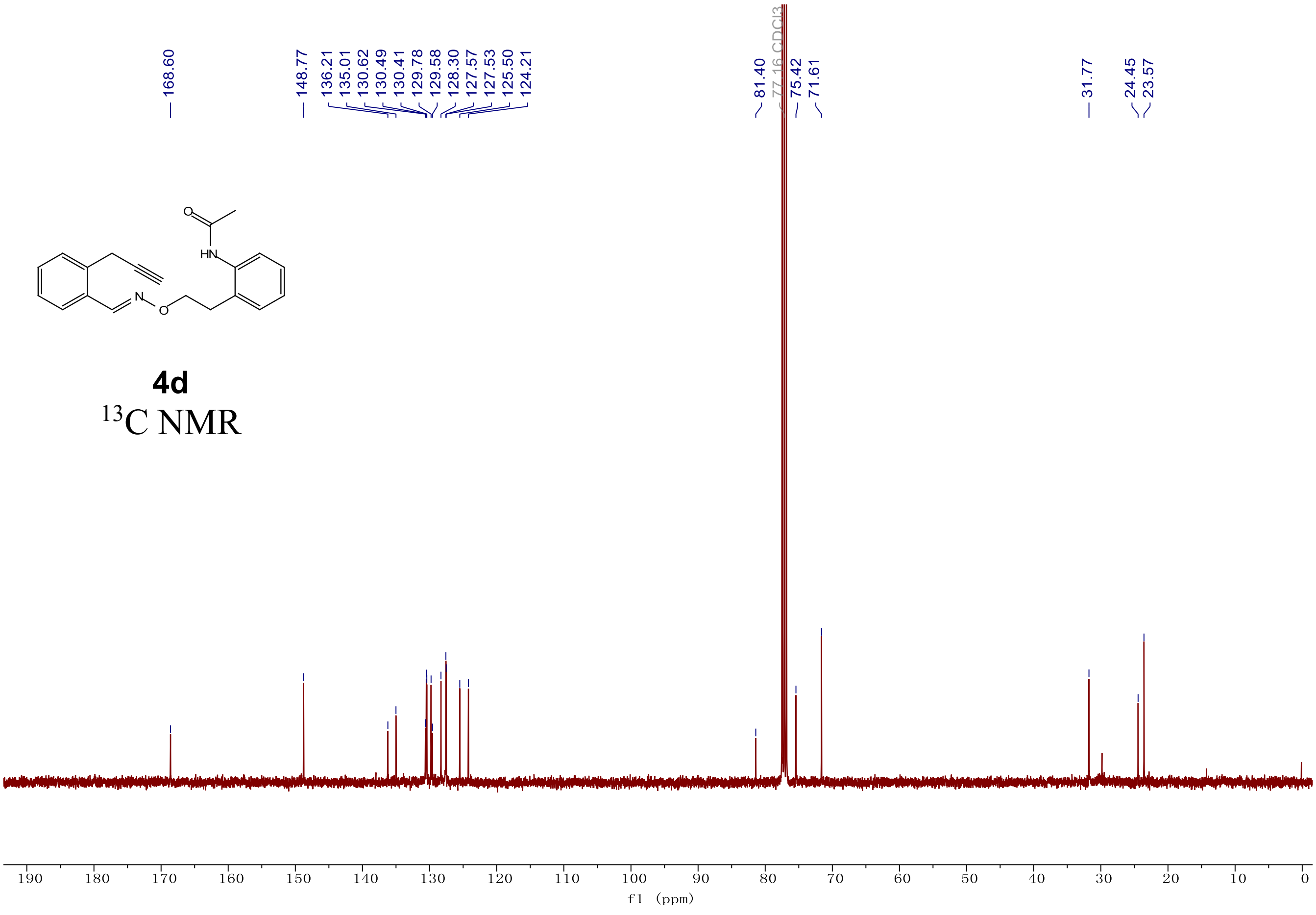
168.60
148.77
136.21
135.01
130.62
130.49
130.41
129.78
129.58
128.30
127.57
127.53
125.50
124.21
81.40
77.16 CDCl3
75.42
71.61
31.77
24.45
23.57
O
HN
N
O
4d
13C NMR
190
180
170
160
150
140
130
120
110
100
90
80
70
60
50
40
30
20
10
0
f1 (ppm)

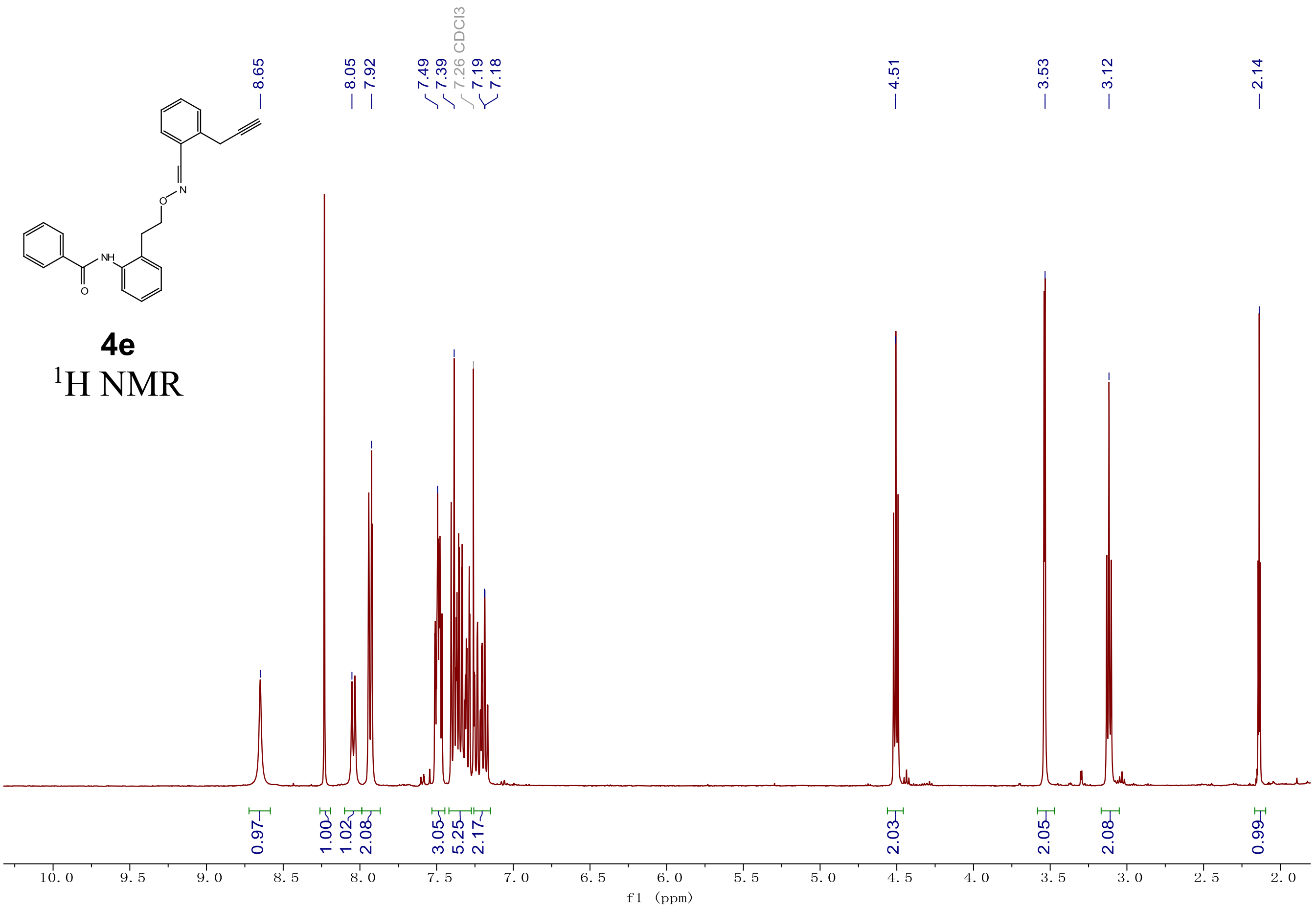

4e

^{1}H NMR

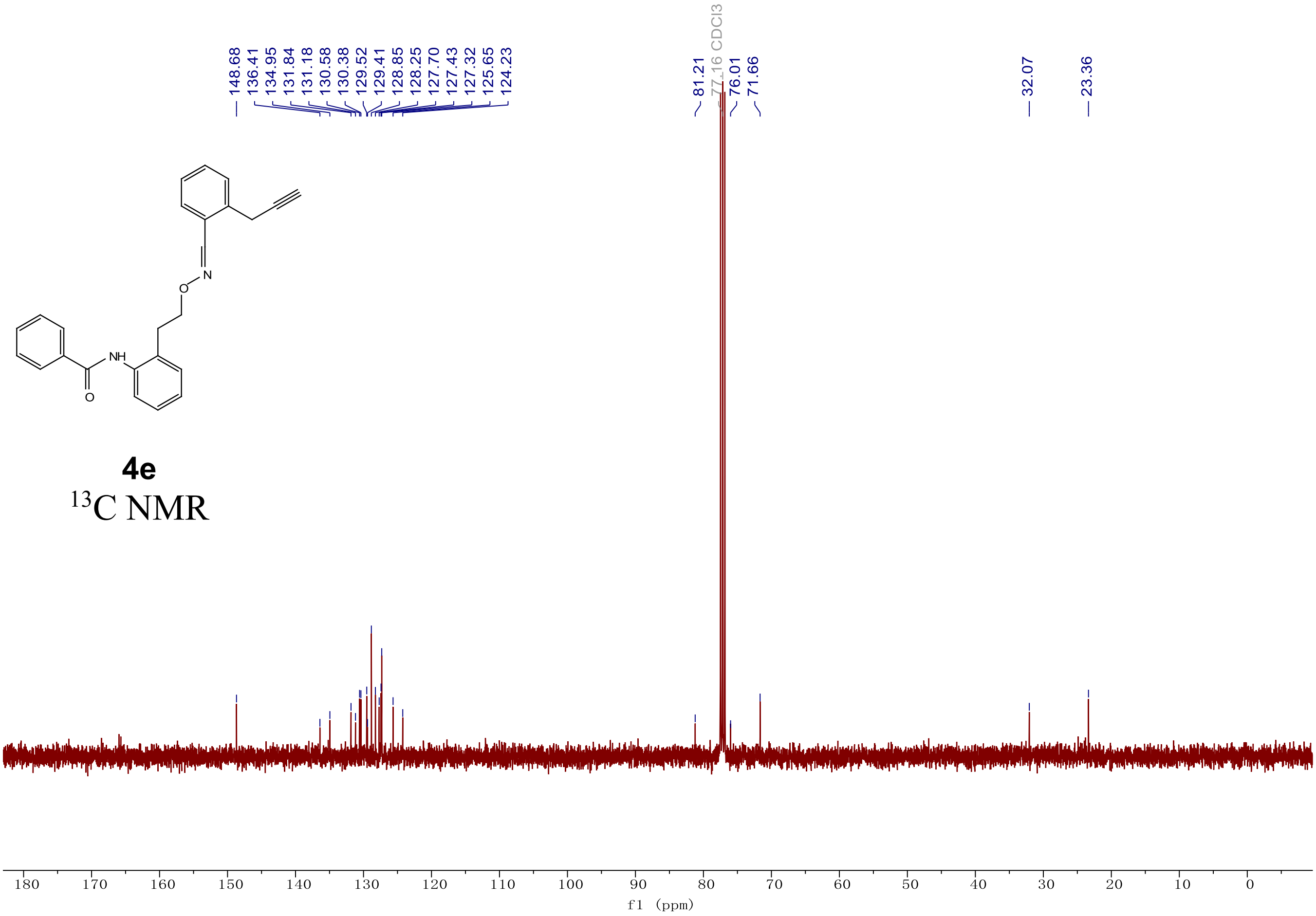

4e

^{13}C NMR

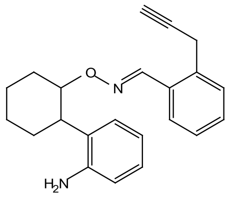

4f

^{1}H NMR

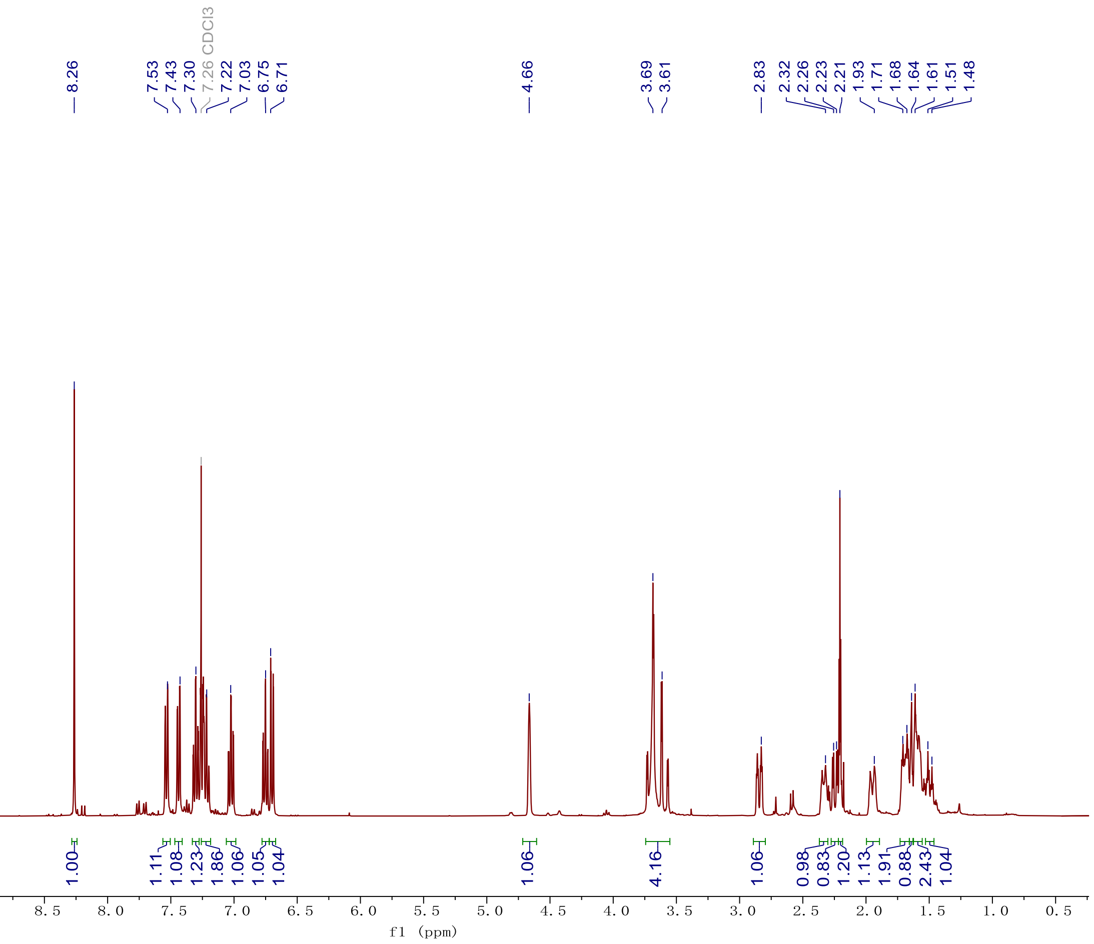

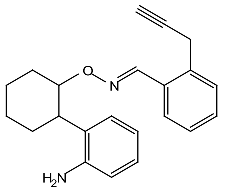

4f

^{13}C NMR

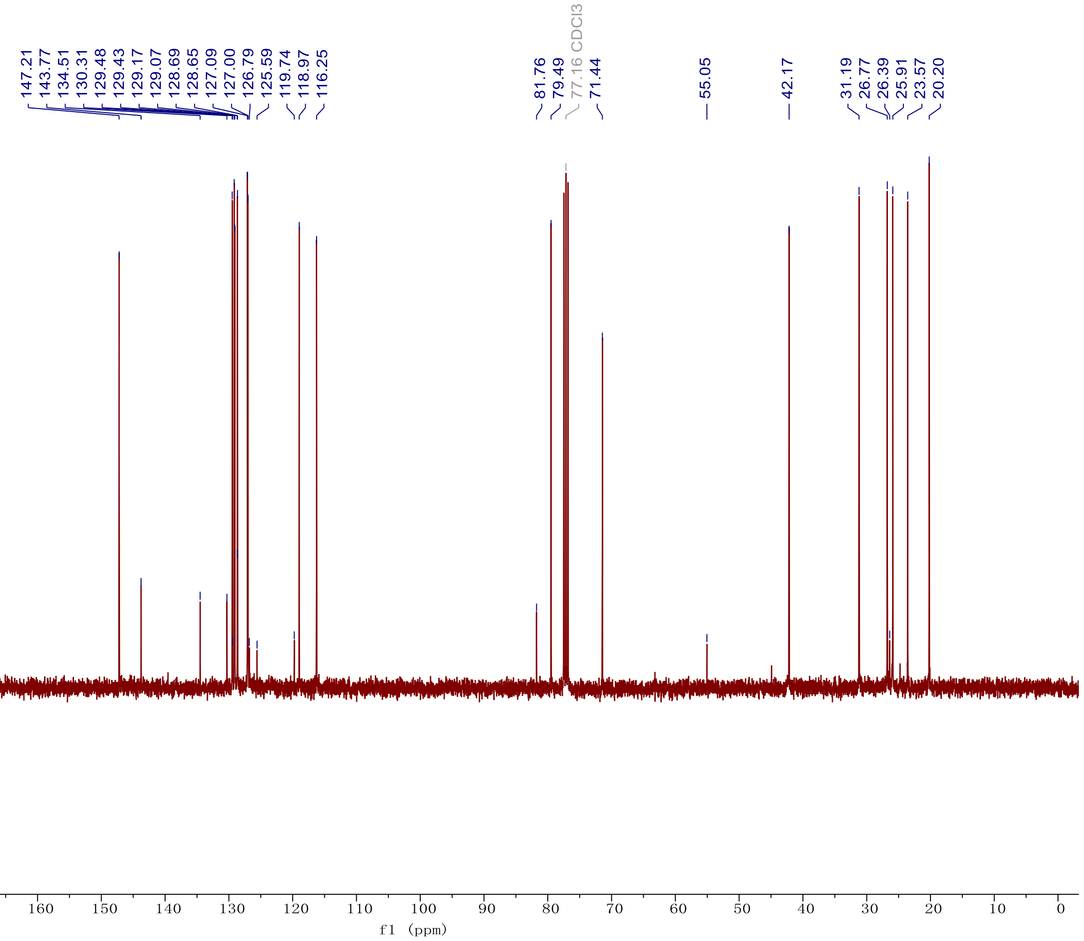

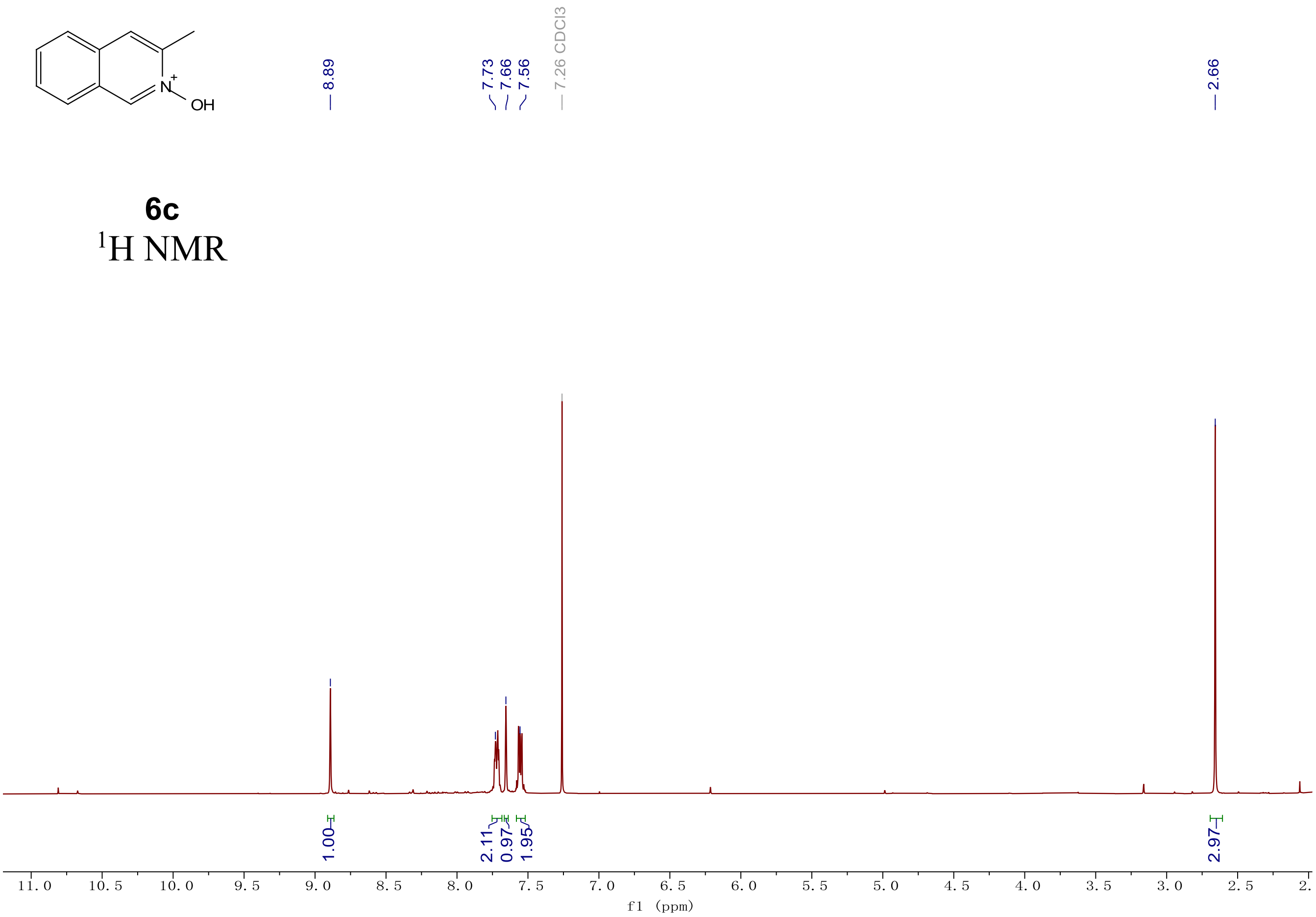

6c
^{1}H NMR

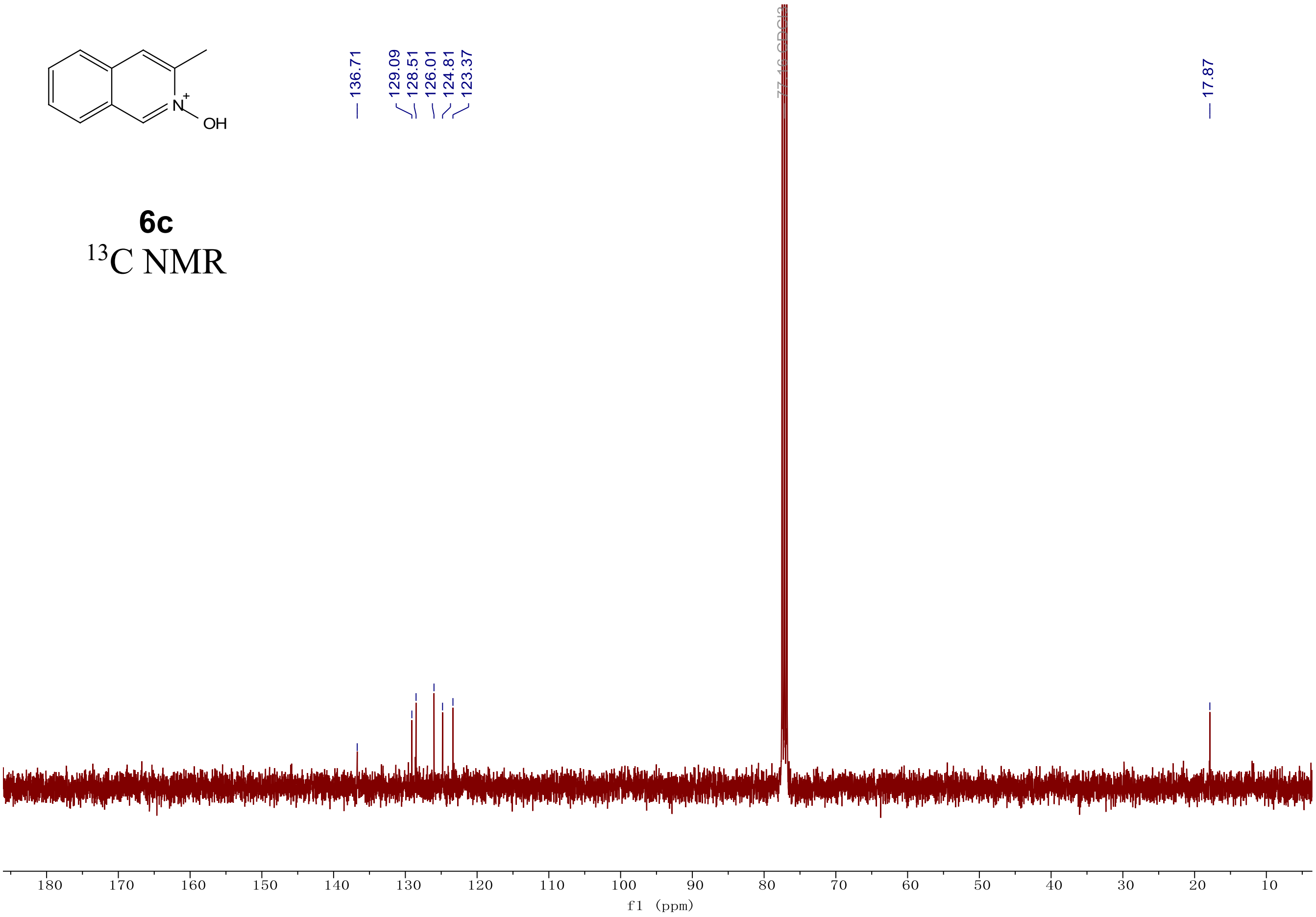

6c
^{13}C NMR

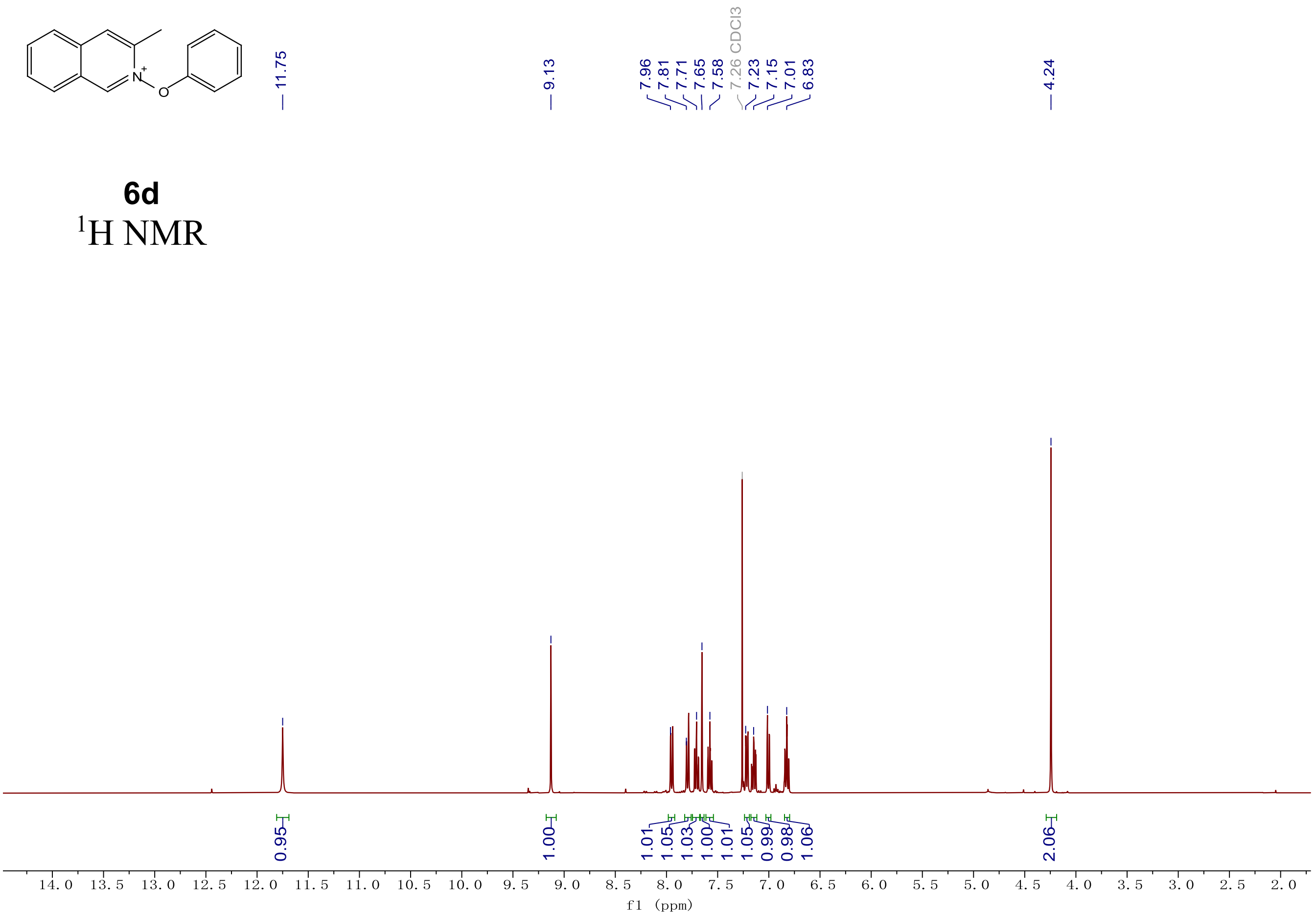

6d
^{1}H NMR

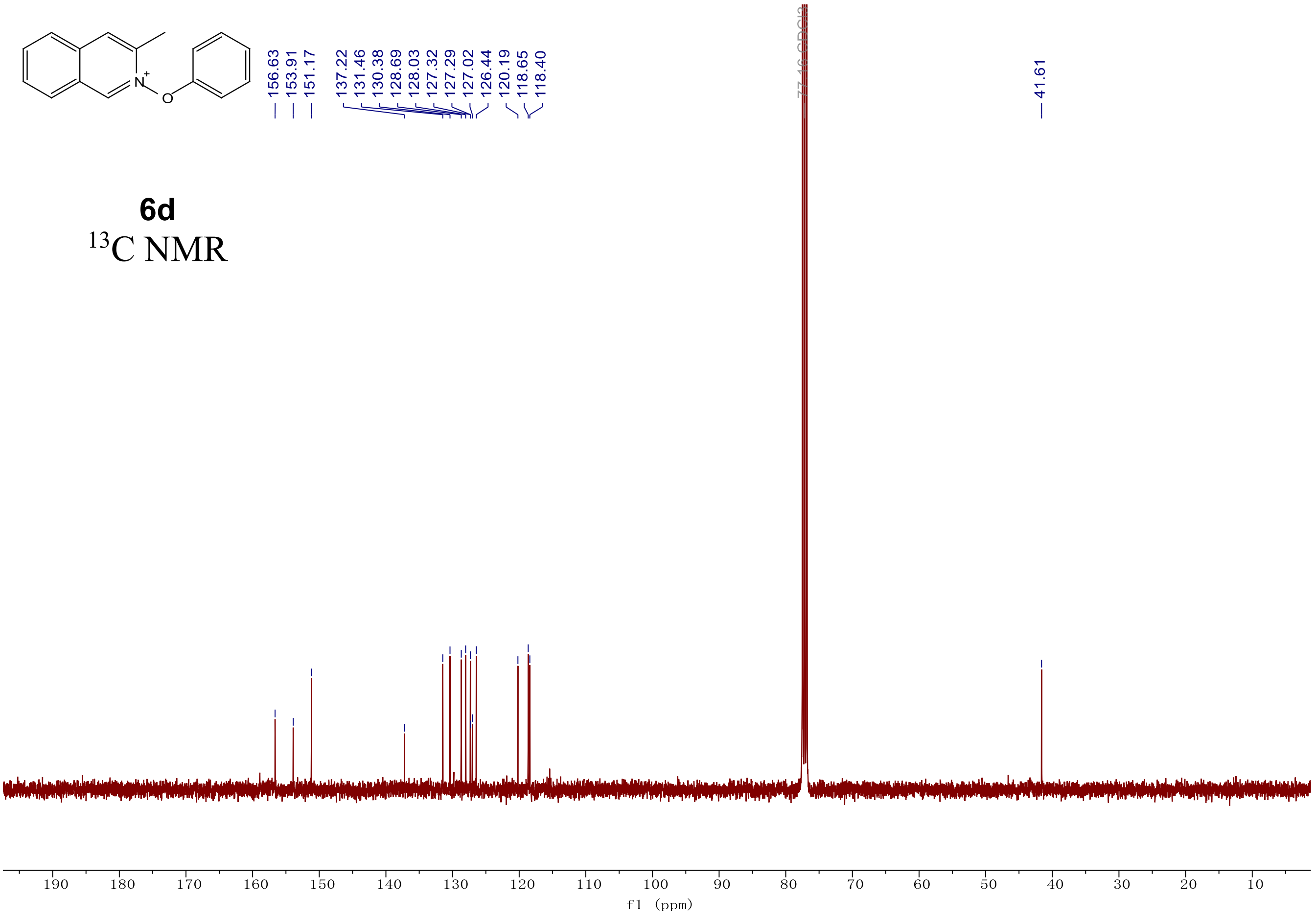

6d
^{13}C NMR

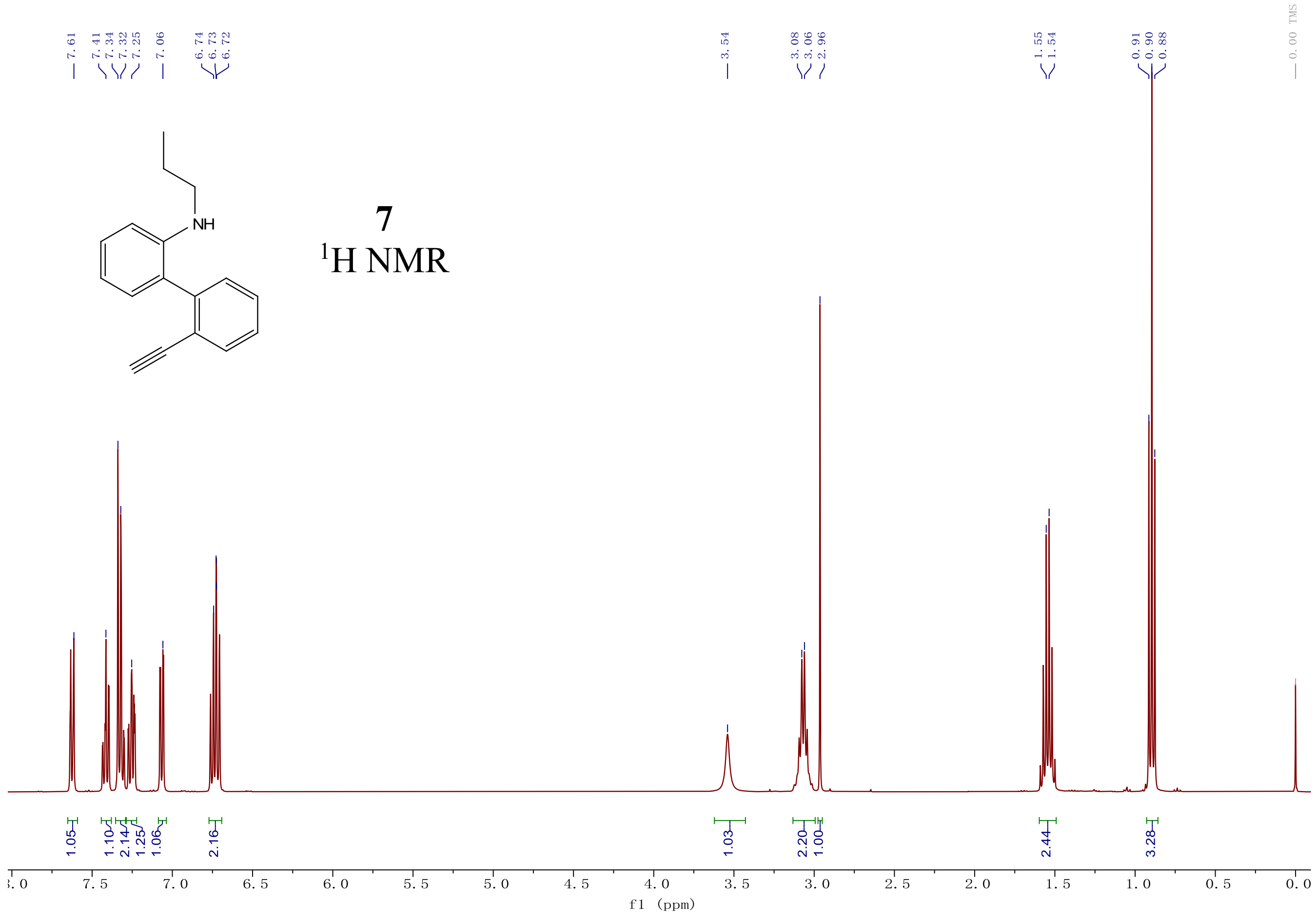
7
1H NMR
NH
7.61
7.41
7.34
7.32
7.25
7.06
6.74
6.73
6.72
3.54
3.08
3.06
2.96
1.55
1.54
0.91
0.90
0.88
0.00 TMS
1.05
1.10
2.14
1.25
1.06
2.16
1.03
2.20
1.00
2.44
3.28
f1 (ppm)

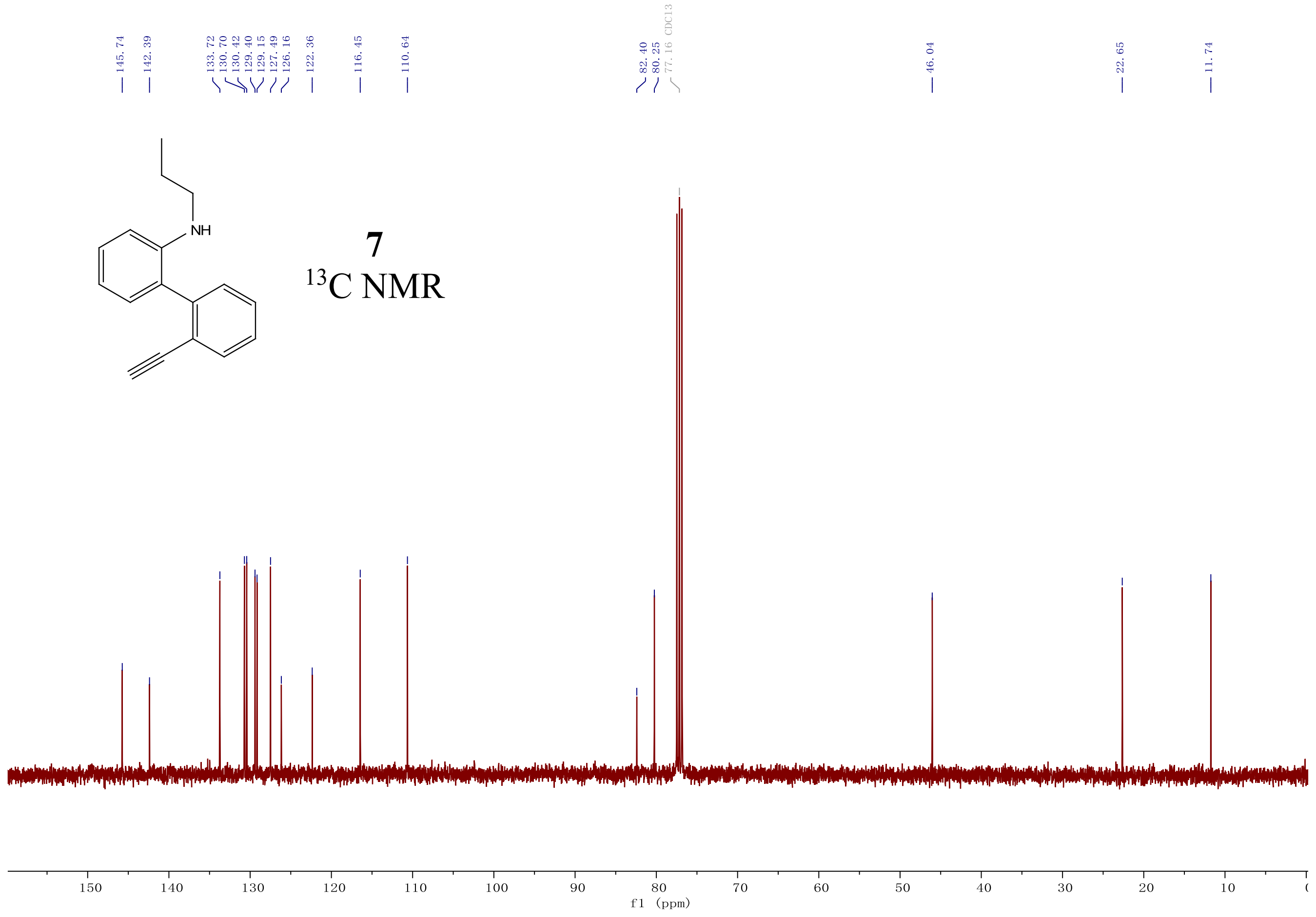
7
13C NMR
NH
145.74
142.39
133.72
130.70
130.42
129.40
129.15
127.49
126.16
122.36
116.45
110.64
82.40
80.25
77.16 CDCl3
46.04
22.65
11.74
f1 (ppm)

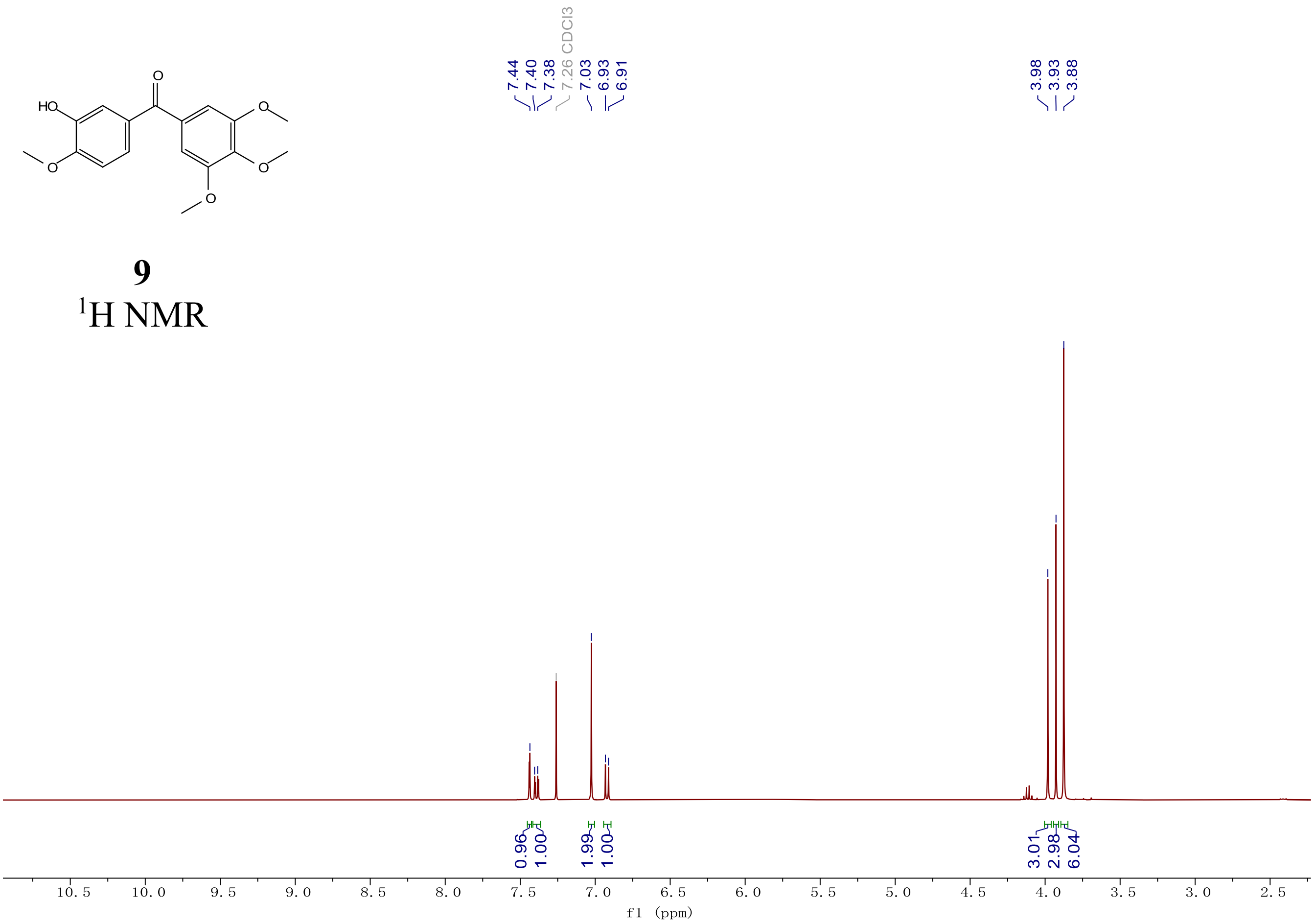

9

^{1}H NMR

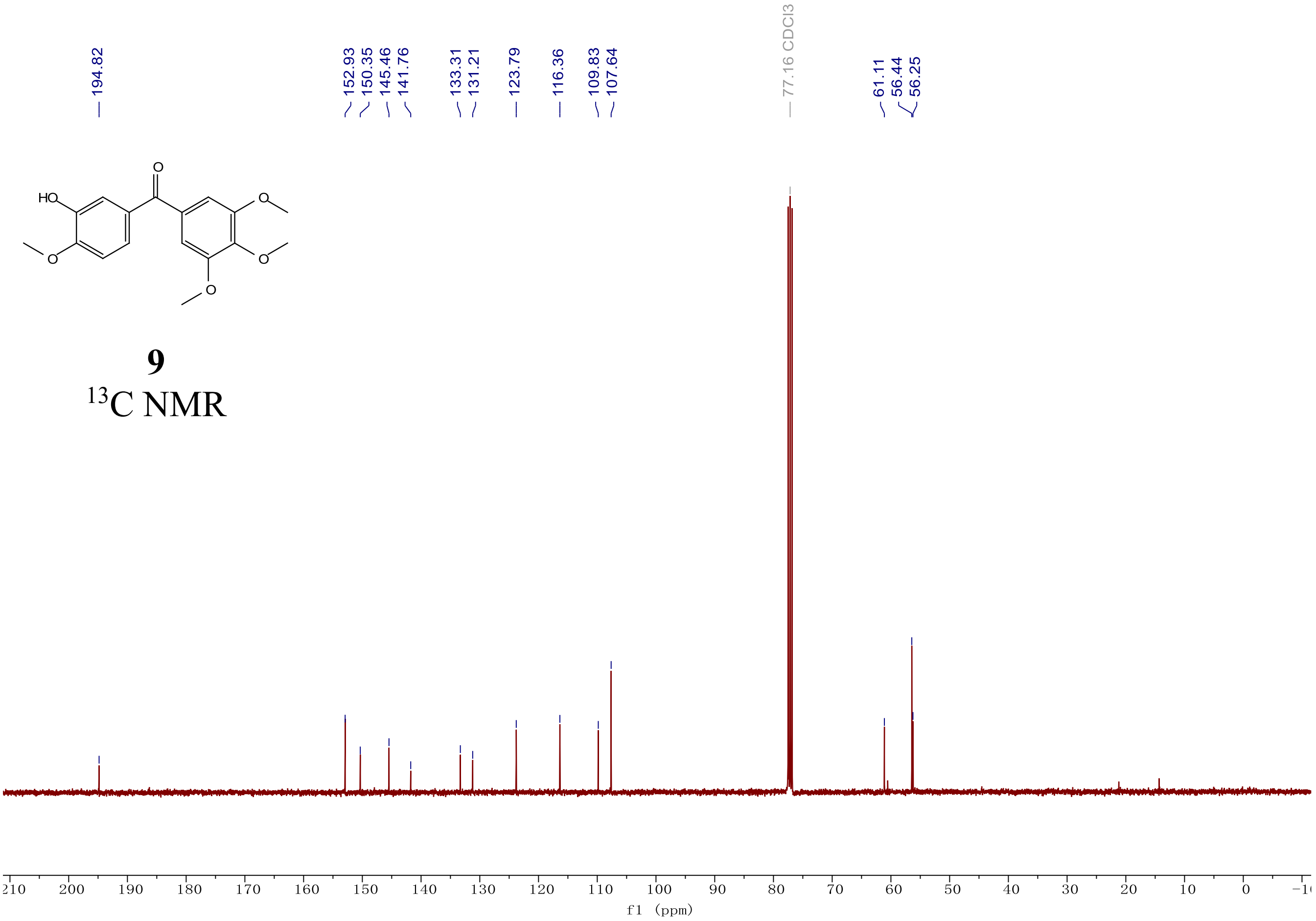

9

^{13}C NMR

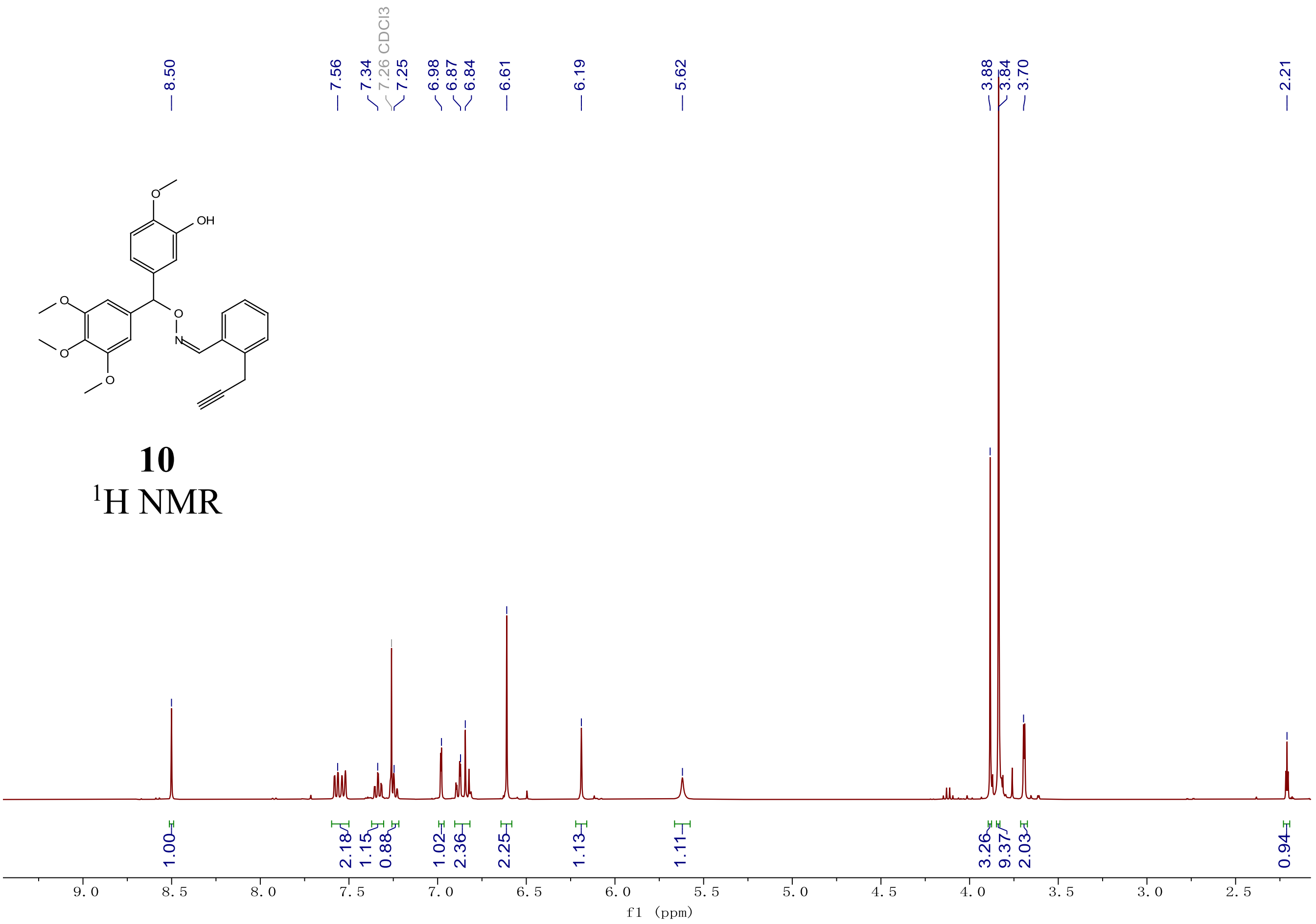

10
^{1}H NMR

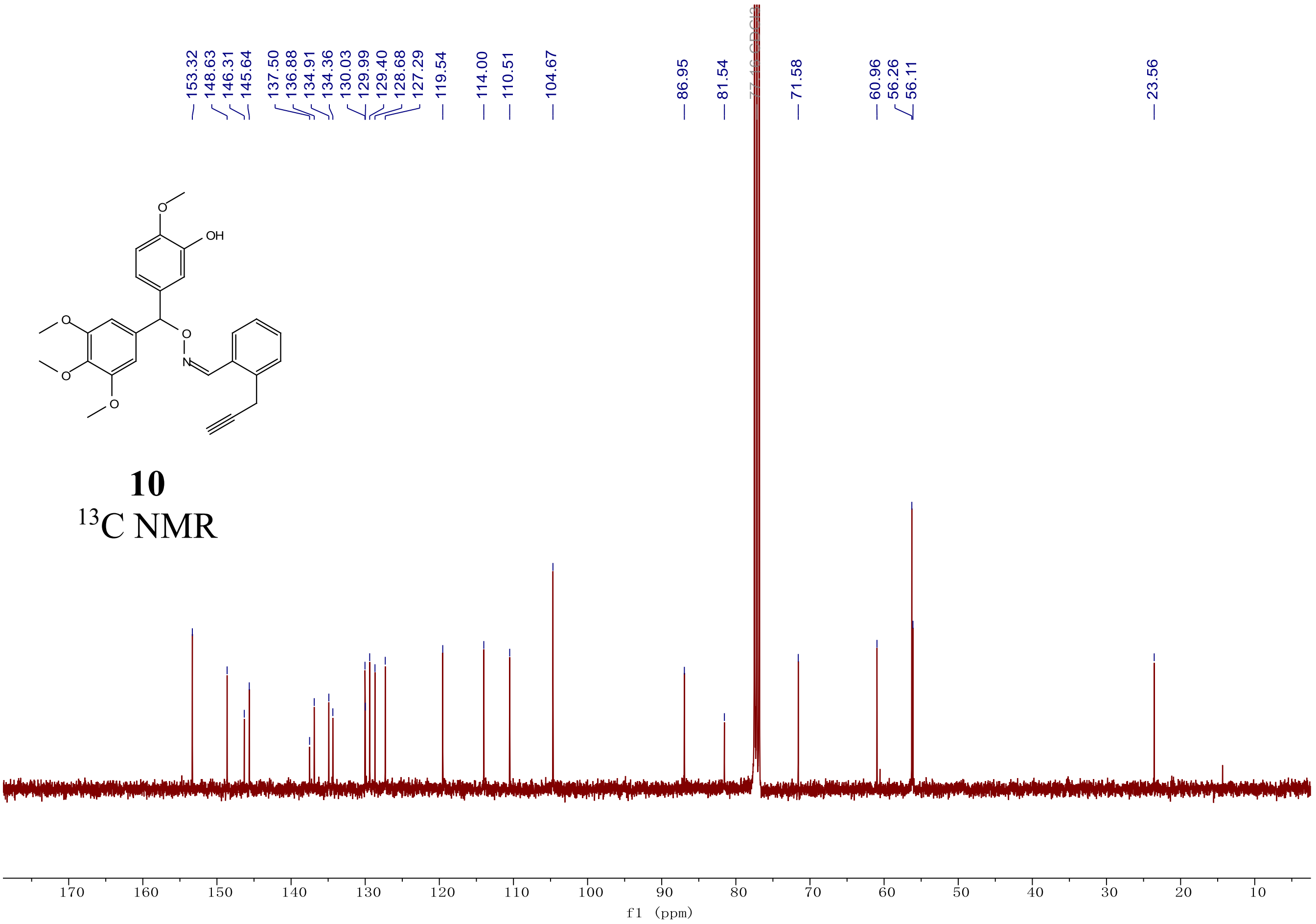

10
^{13}C NMR

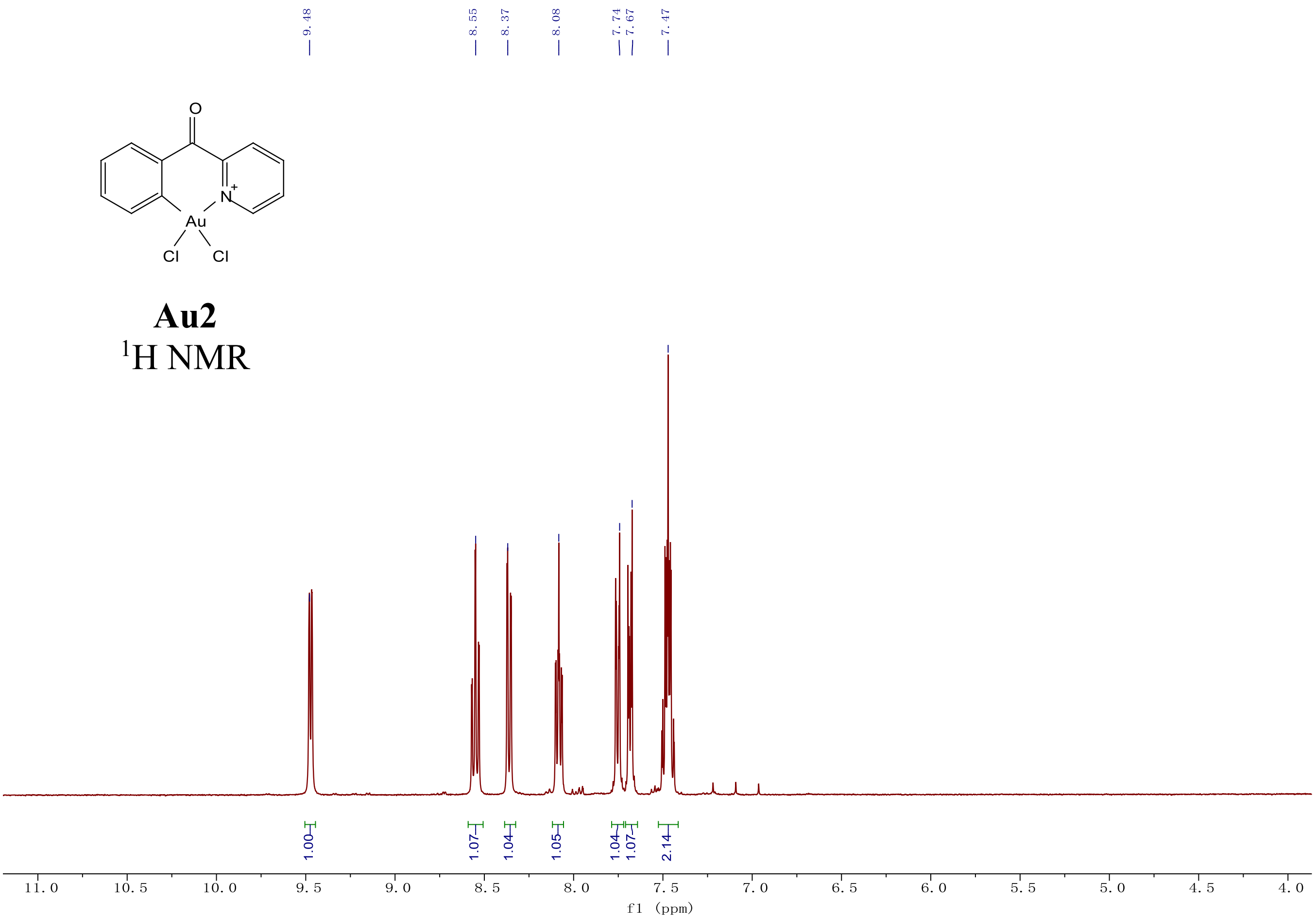

Au2
1H NMR
9.48
8.55
8.37
8.08
7.74
7.67
7.47
1.00
1.07
1.04
1.05
1.04
1.07
2.14
11.0 10.5 10.0 9.5 9.0 8.5 8.0 7.5 7.0 6.5 6.0 5.5 5.0 4.5 4.0
f1 (ppm)
O
N+
Au
Cl Cl

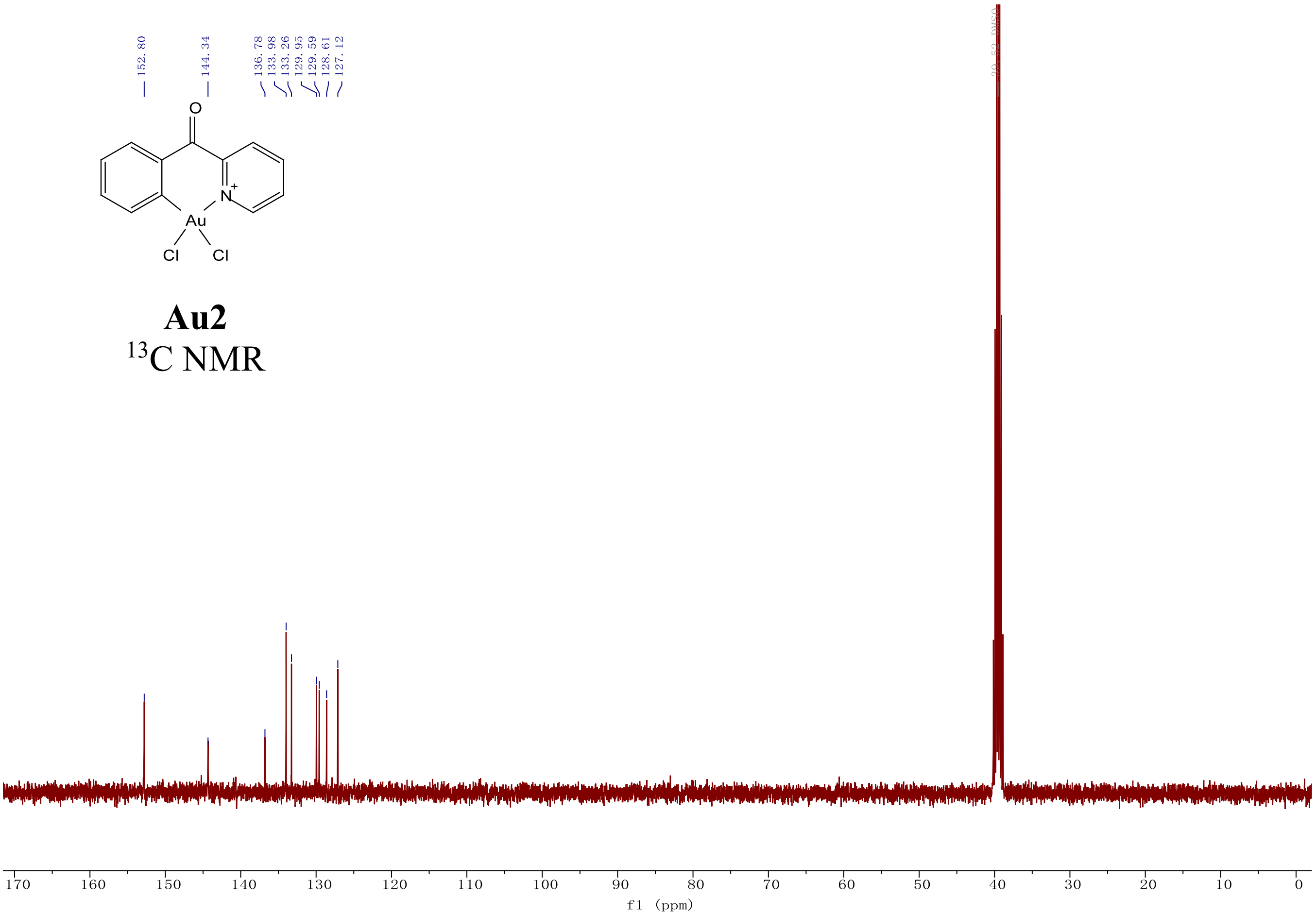

Au2
13C NMR
152.80
144.34
136.78
133.98
133.26
129.95
129.59
128.61
127.12
170 160 150 140 130 120 110 100 90 80 70 60 50 40 30 20 10 0
f1 (ppm)
O
N+
Au
Cl Cl

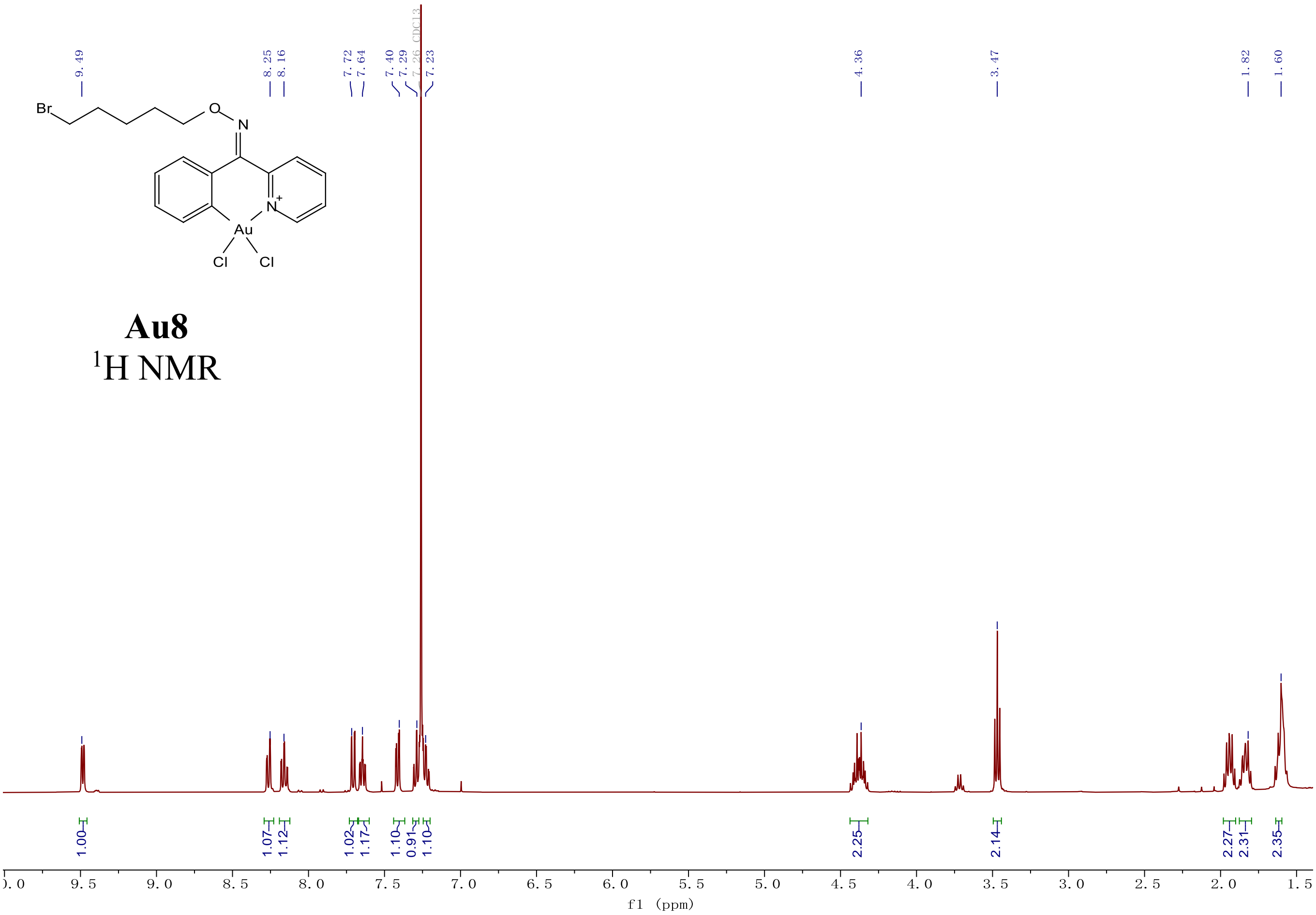

Au8

^{1}H NMR

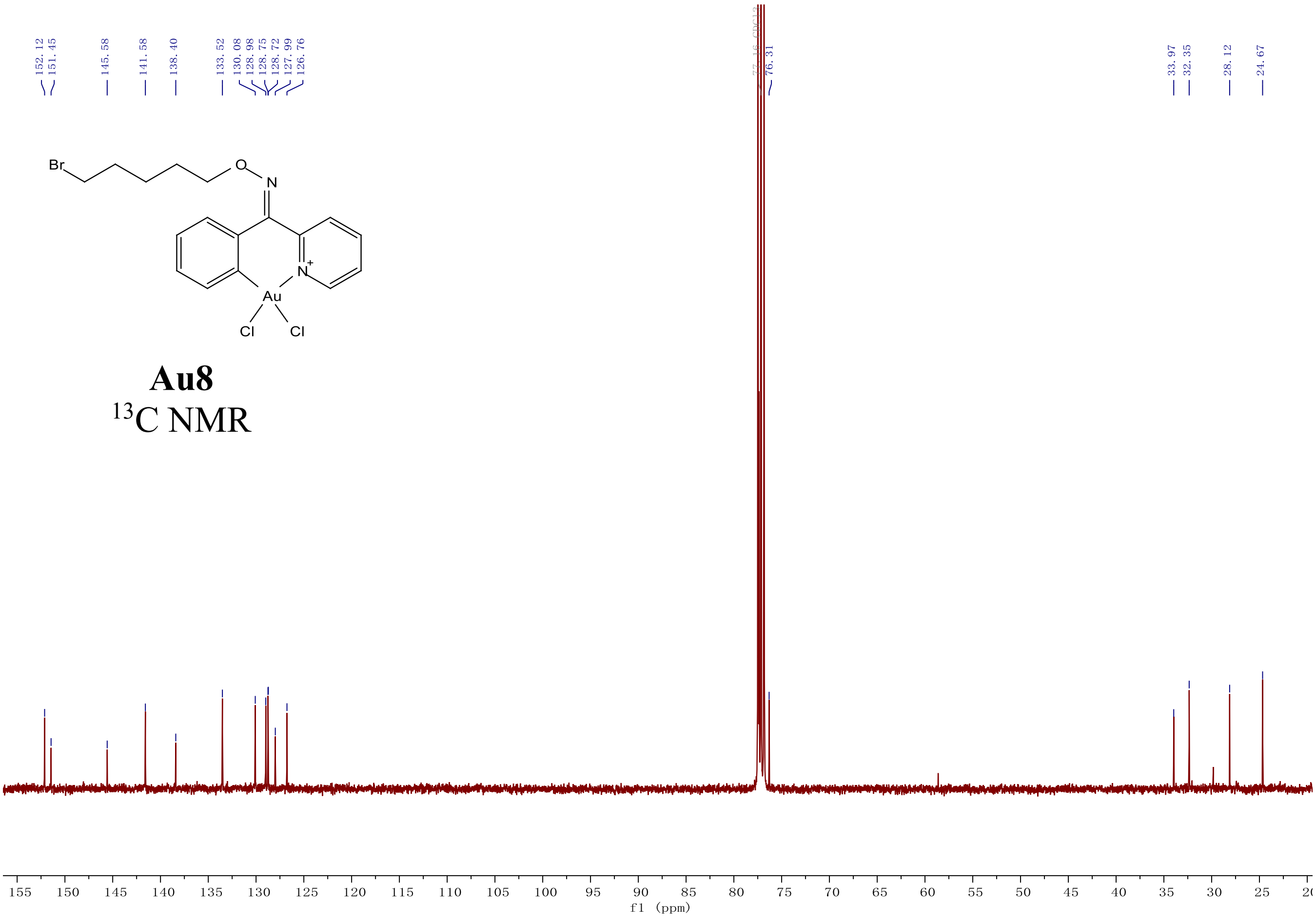

Au8

^{13}C NMR

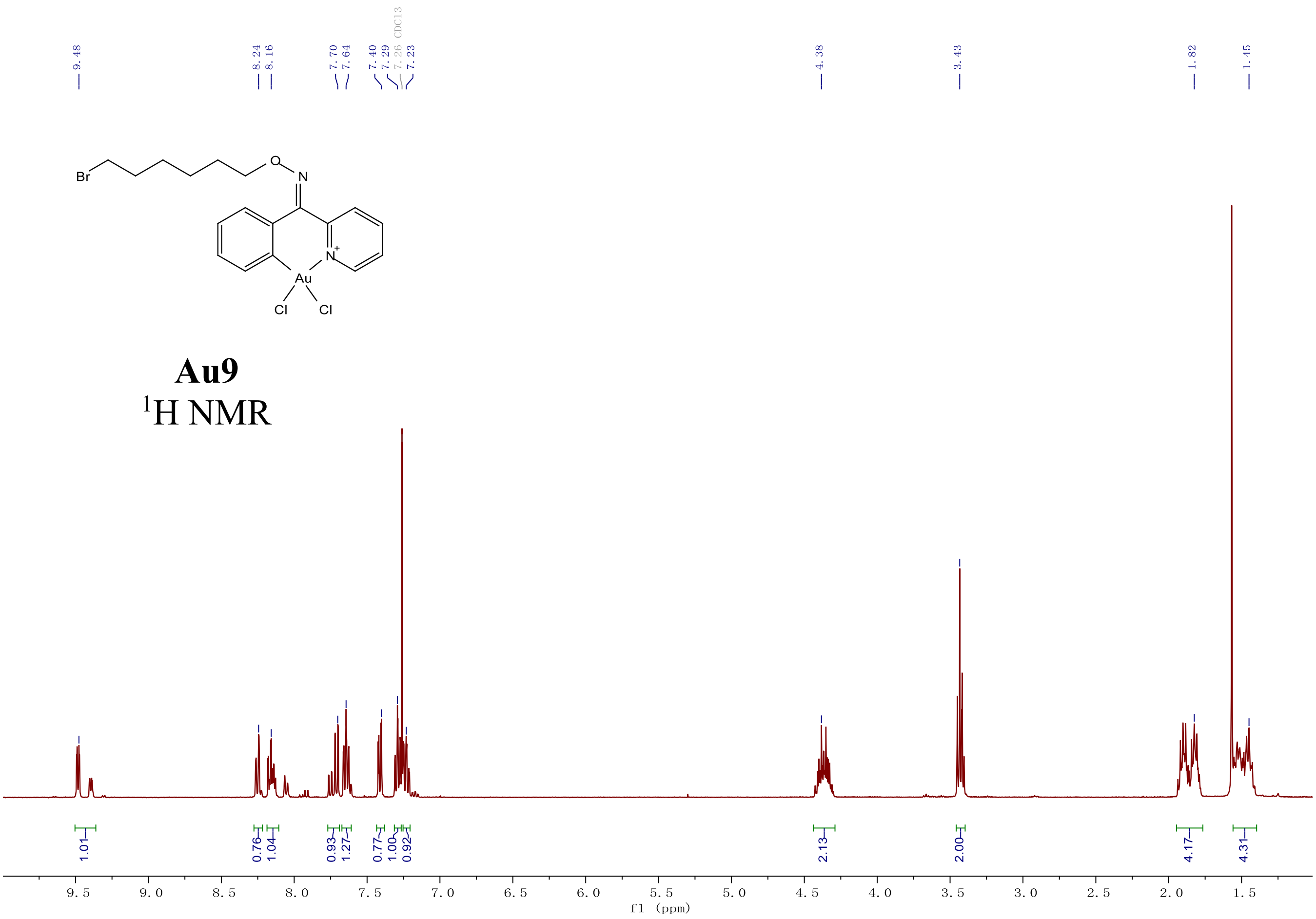
9.48
8.24
8.16
7.70
7.61
7.40
7.29
7.26 CDCl3
7.23
4.38
3.43
1.82
1.45
Br
O
N
N+
Au
Cl
Cl
Au9
1H NMR
1.01
0.76
1.04
0.93
1.27
0.77
1.00
0.92
2.13
2.00
4.17
4.31
f1 (ppm)

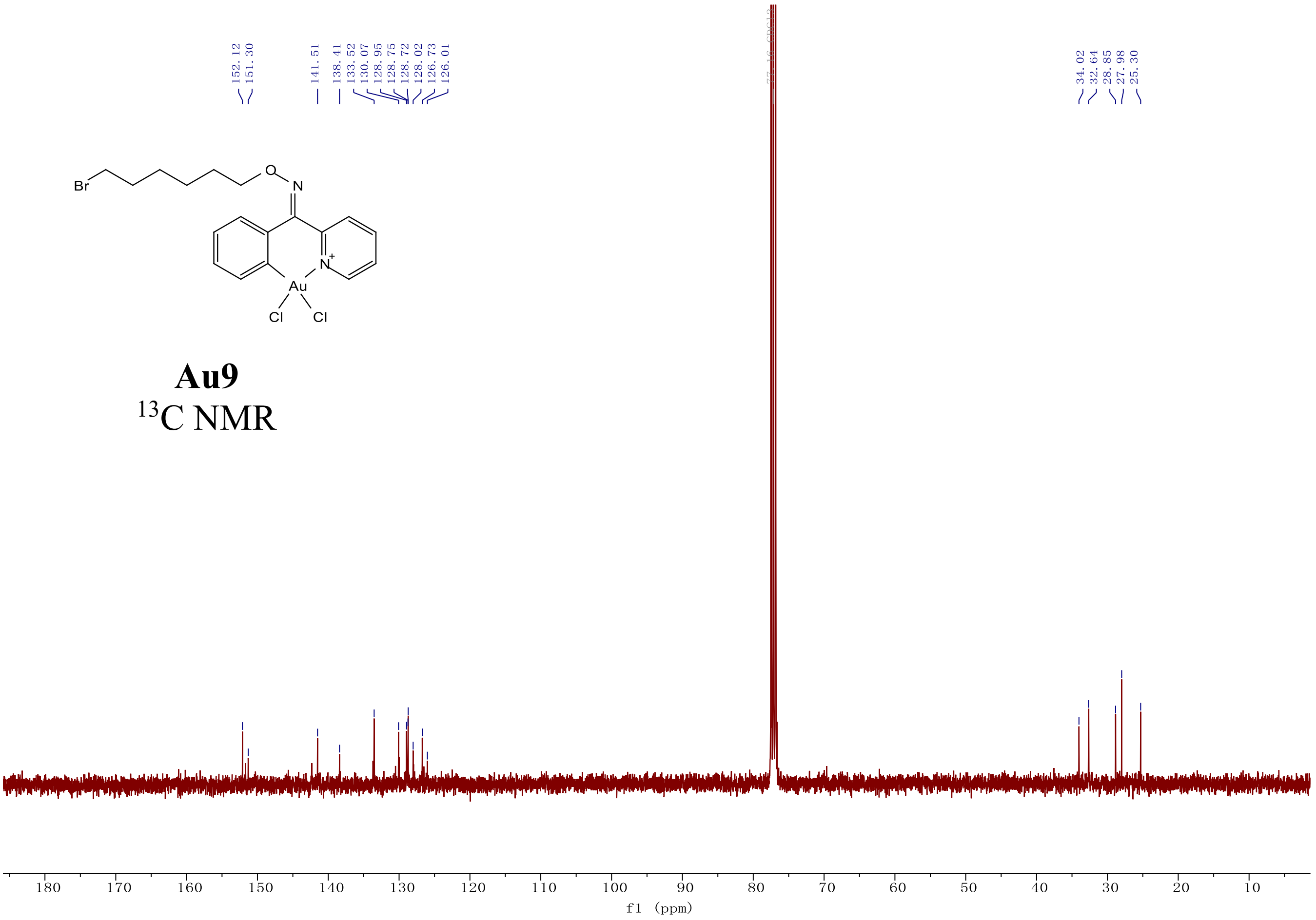
152.12
151.30
141.51
138.41
133.52
130.07
128.95
128.75
128.72
128.02
126.73
126.01
34.02
32.64
28.85
27.98
25.30
Br
O
N
N+
Au
Cl
Cl
Au9
13C NMR
f1 (ppm)

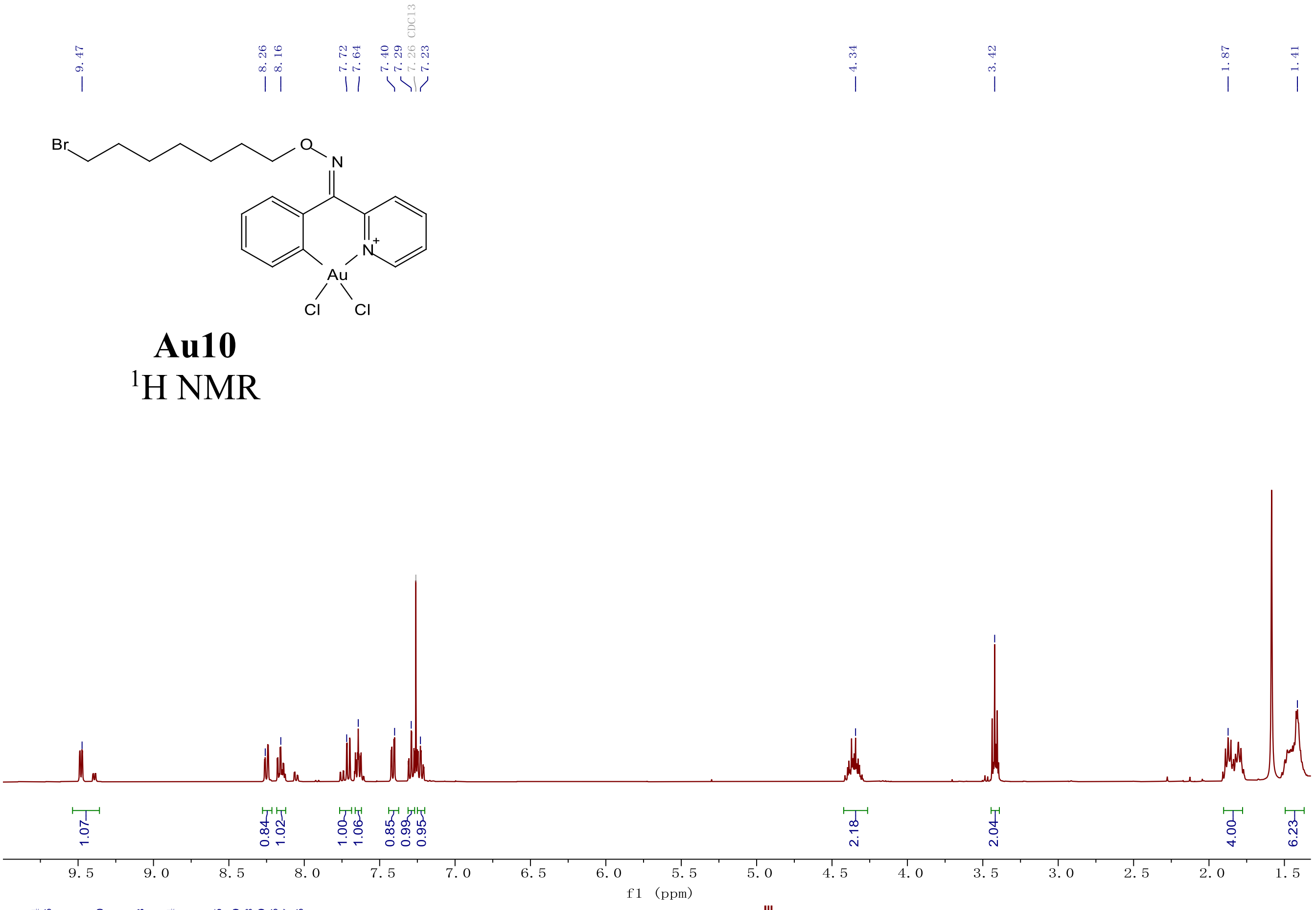

Au10

^{1}H NMR

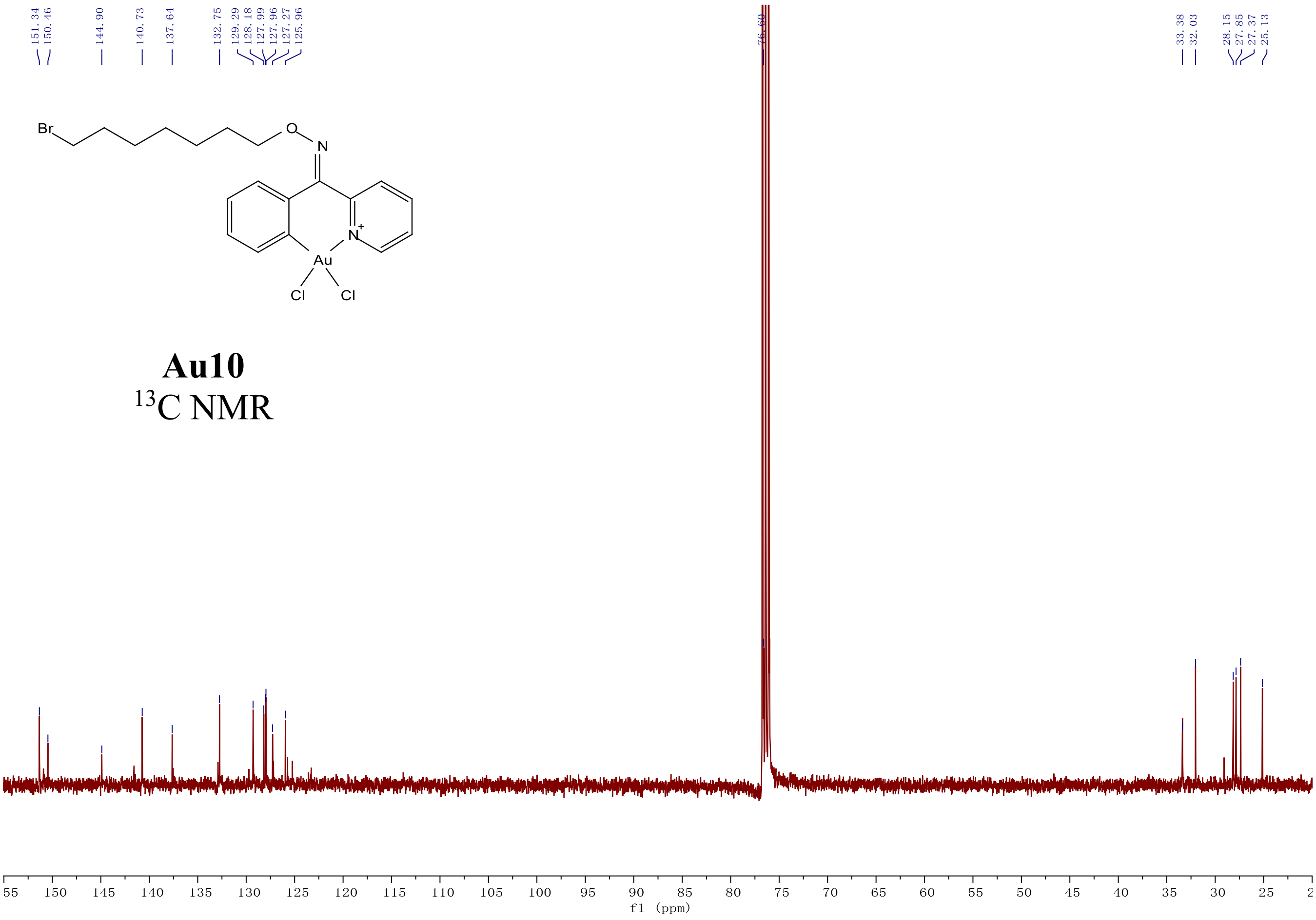

Au10

^{13}C NMR

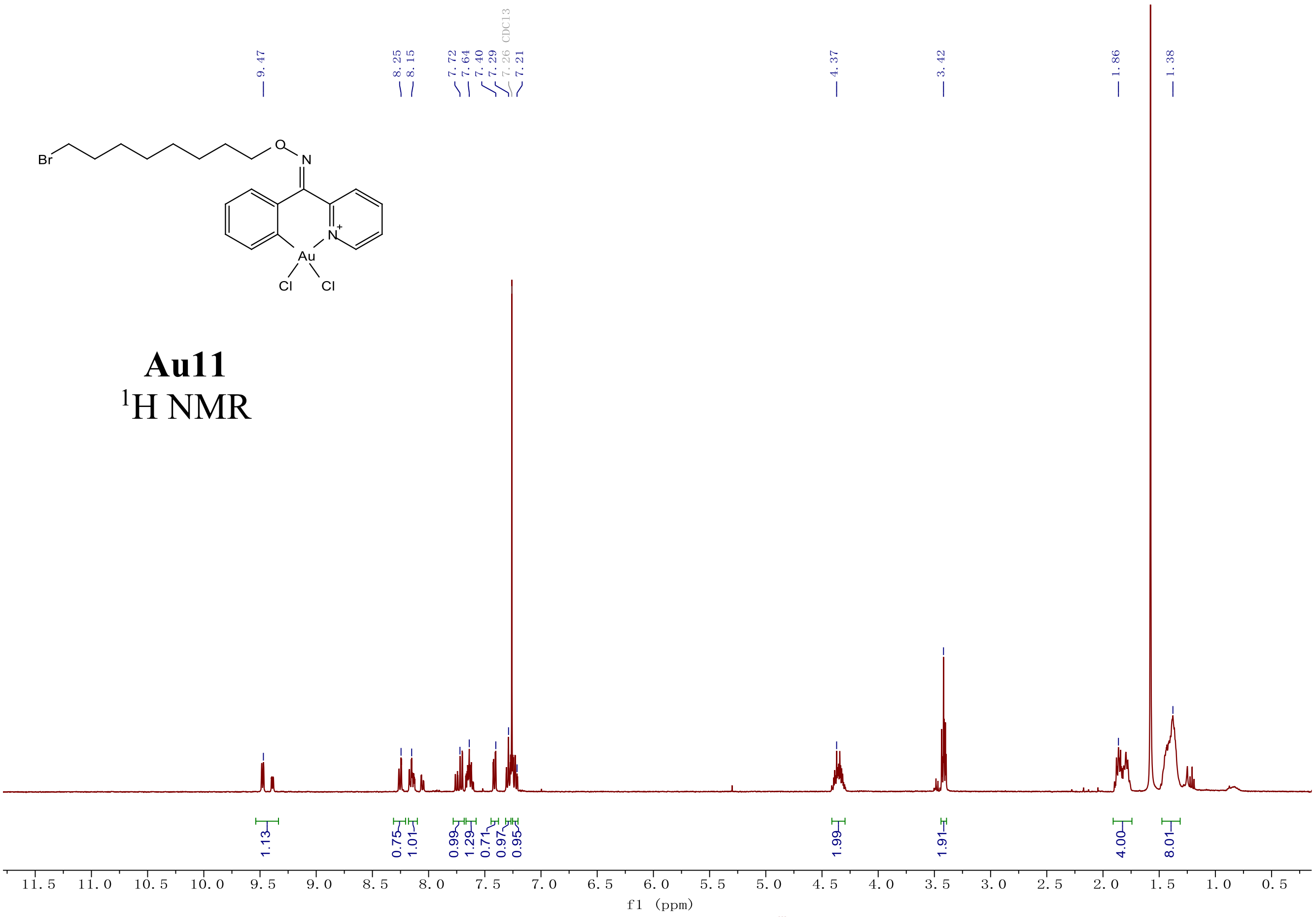
Au11
1H NMR
Br
O
N
N+
Au
Cl
Cl
9.47
8.25
8.15
7.72
7.64
7.40
7.29
7.26 CDCl3
7.21
4.37
3.42
1.86
1.38
1.13
0.75
1.01
0.99
1.29
0.71
0.97
0.95
1.99
1.91
4.00
8.01
f1 (ppm)

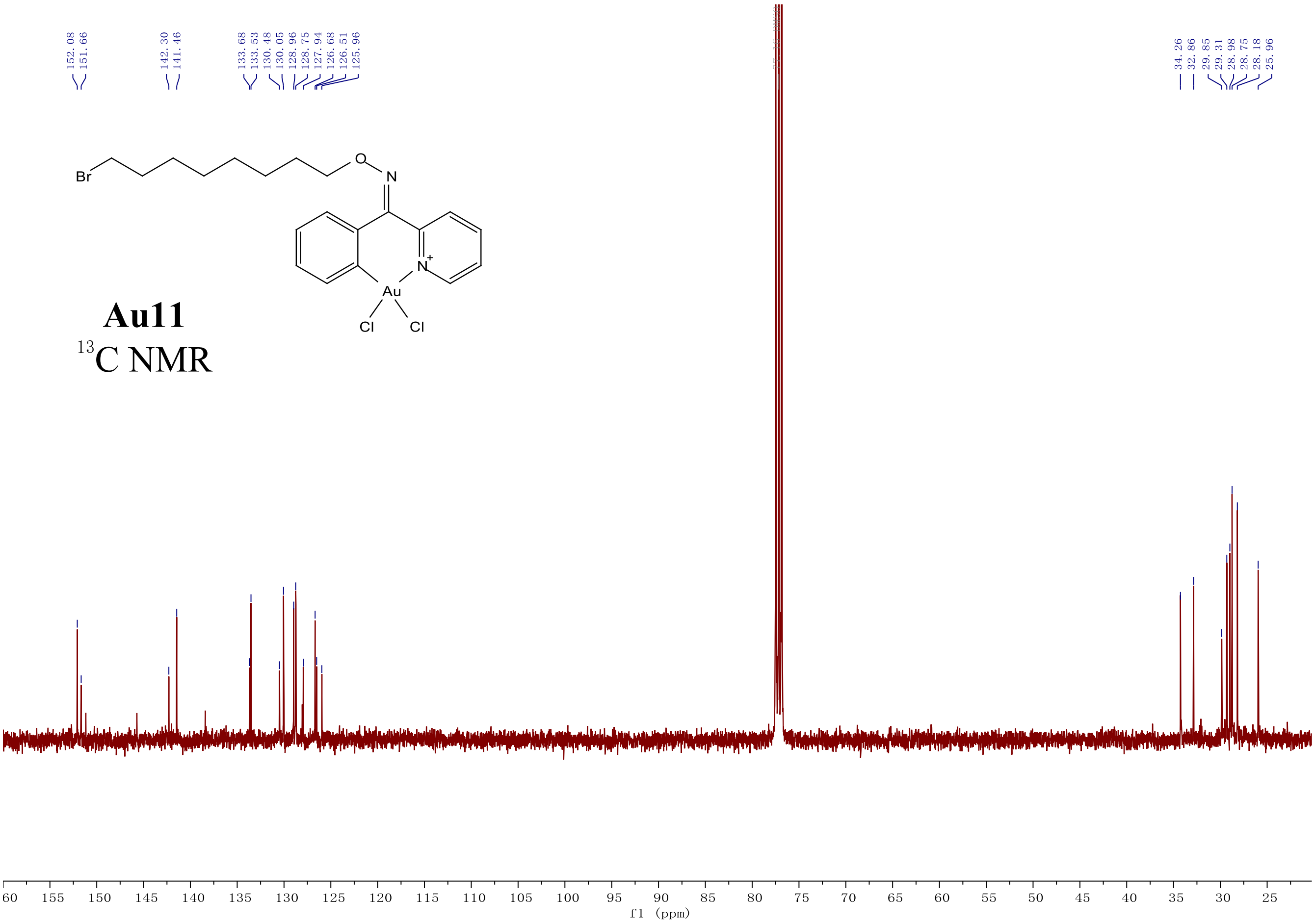
Au11
13C NMR
Br
O
N
N+
Au
Cl
Cl
152.08
151.66
142.30
141.46
133.68
133.53
130.48
130.05
128.96
128.75
127.94
126.68
126.51
125.96
34.26
32.86
29.85
29.31
28.98
28.75
28.18
25.96
f1 (ppm)

7. References

(1) Ravindranath, P. A.; Forli, S.; Goodsell, D. S.; Olson, A. J.; Sanner, M. F. AutoDockFR: Advances in Protein-Ligand Docking with Explicitly Specified Binding Site Flexibility. *PLOS Computational Biology* **2015**, *11* (12), e1004586.

(2) Ravindranath, P. A.; Sanner, M. F. AutoSite: an automated approach for pseudo-ligands prediction-from ligand-binding sites identification to predicting key ligand atoms. *Bioinformatics* **2016**, *32* (20), 3142-3149.

(3) Zhang, Y.; Forli, S.; Omelchenko, A.; Sanner, M. F. AutoGridFR: Improvements on AutoDock Affinity Maps and Associated Software Tools. *Journal of Computational Chemistry* **2019**, *40* (32), 2882-2886.

(4) Trott, O.; Olson, A. J. AutoDock Vina: Improving the speed and accuracy of docking with a new scoring function, efficient optimization, and multithreading. *Journal of Computational Chemistry* **2010**, *31* (2), 455-461.

(5) Morris, G. M.; Huey, R.; Lindstrom, W.; Sanner, M. F.; Belew, R. K.; Goodsell, D. S.; Olson, A. J. AutoDock4 and AutoDockTools4: Automated docking with selective receptor flexibility. *Journal of Computational Chemistry* **2009**, *30* (16), 2785-2791.
